# Behavioral Phenotyping of an Improved Mouse Model of Phelan–McDermid Syndrome with a Complete Deletion of the *Shank3* Gene

**DOI:** 10.1523/ENEURO.0046-18.2018

**Published:** 2018-10-05

**Authors:** Elodie Drapeau, Mohammed Riad, Yuji Kajiwara, Joseph D. Buxbaum

**Affiliations:** 1 Seaver Autism Center for Research and Treatment, Icahn School of Medicine at Mount Sinai, New York, NY 10029; 2 Department of Psychiatry, Icahn School of Medicine at Mount Sinai, New York, NY 10029; 3 Department of Genetics and Genomic Sciences, Icahn School of Medicine at Mount Sinai, New York, NY 10029; 4 Department of Neuroscience, Icahn School of Medicine at Mount Sinai, New York, NY 10029; 5 Mindich Child Health and Development Institute, Icahn School of Medicine at Mount Sinai, New York, NY 10029; 6 Friedman Brain Institute, Icahn School of Medicine at Mount Sinai, New York, NY 10029

**Keywords:** 22q13, autism spectrum disorder, behavior, mouse model, Phelan–McDermid syndrome, Shank3

## Abstract

Phelan–McDermid syndrome (PMS) is a rare genetic disorder in which one copy of the *SHANK3* gene is missing or mutated, leading to a global developmental delay, intellectual disability (ID), and autism. Multiple intragenic promoters and alternatively spliced exons are responsible for the formation of numerous isoforms. Many genetically-modified mouse models of PMS have been generated but most disrupt only some of the isoforms. In contrast, the vast majority of known *SHANK3* mutations found in patients involve deletions that disrupt all isoforms. Here, we report the production and thorough behavioral characterization of a new mouse model in which all *Shank3* isoforms are disrupted. Domains and tasks examined in adults included measures of general health, neurological reflexes, motor abilities, sensory reactivity, social behavior, repetitive behaviors, cognition and behavioral inflexibility, and anxiety. Our mice are more severely affected than previously published models. While the deficits were typically more pronounced in homozygotes, an intermediate phenotype was observed for heterozygotes in many paradigms. As in other *Shank3* mouse models, stereotypies, including increased grooming, were observed. Additionally, sensory alterations were detected in both neonatal and adult mice, and motor behavior was strongly altered, especially in the open field and rotarod locomotor tests. While social behaviors measured with the three-chambered social approach and male-female interaction tests were not strongly impacted, Shank3-deficient mice displayed a strong escape behavior and avoidance of inanimate objects in novel object recognition, repetitive novel object contact, marble burying, and nest building tasks, indicating increased novelty-induced anxiety. Similarly, increased freezing was observed during fear conditioning training and amygdala-dependent cued retrieval. Finally, deficits were observed in both initial training and reversal in the Barnes maze and in contextual fear testing, which are memory tasks involving hippocampal-prefrontal circuits. In contrast, working memory in the Y-maze spontaneous alternation test was not altered. This new mouse model of PMS, engineered to most closely represent human mutations, recapitulates core symptoms of PMS providing improvements for both construct and face validity, compared to previous models.

## Significance Statement

Phelan–McDermid syndrome (PMS), caused by happloinsufficiency of *Shank3*, is a severe and complex neurodevelopmental disorder. This study investigates the behavioral consequences of a disruption of all *Shank3* isoforms in neonatal and adult mice using a detailed battery of tests tailored to investigate core symptoms and usual comorbidities of PMS. We found that our new model is more severely affected than previously published mouse models with only partial deletions of *Shank3* and more closely recapitulates symptoms of PMS, thus providing improvements for both construct and face validity. Our results highlight the significance of using a mouse model with a complete deletion of *Shank3* for studying mechanisms underlying autism spectrum disorder (ASD) and PMS, carrying preclinical studies and testing test novel therapeutic approaches.

## Introduction

Phelan–McDermid syndrome (PMS) is a rare and complex neurodevelopmental disorder that manifests with global developmental delay, mild dysmorphic features, motor deficits, variable degrees of intellectual disability (ID), and absent or delayed speech. Additionally, autism spectrum disorder (ASD), epilepsy, attention deficits, and recurrent medical comorbidities are common in patients with PMS (Phelan and McDermid, 2012; Betancur and Buxbaum, 2013; Soorya et al., 2013; Sarasua et al., 2014a). Recent studies show that PMS is emerging as one of the most frequent and penetrant monogenic causes of autism and ID (Sykes et al., 2009; Betancur and Buxbaum, 2013; Soorya et al., 2013; Leblond et al., 2014).

Despite overlapping etiologies between patients, there is a tremendous heterogeneity in the expression and severity of the phenotype (Cusmano-Ozog et al., 2007; Dhar et al., 2010; Phelan and Betancur, 2011; Soorya et al., 2013). This is no doubt in part due to the complex nature of in the genetic etiology of PMS (De Rubeis et al., 2018). While a large body of data indicates that haploinsufficiency of *SHANK3* is the key contributor for the neurobehavioral manifestations of PMS, it can be caused by a variety of genetic rearrangements including unbalanced translocations, ring chromosome 22, terminal deletions (ranging from deletions of just *SHANK3* to large deletions of up to 9 Mb), and interstitial deletions or point mutation within the *SHANK3* gene (Durand et al., 2007; Moessner et al., 2007; Sykes et al., 2009; Bonaglia et al., 2011; Phelan and McDermid, 2012; Soorya et al., 2013; Leblond et al., 2014; De Rubeis et al., 2018).

Genotype-phenotype analyses have shown positive correlations between the size of the deletion and the number and/or severity of some phenotypes (Luciani et al., 2003; Dhar et al., 2010; Bonaglia et al., 2011; Soorya et al., 2013; Sarasua et al., 2014b). However, findings on specific clinical variables have not been consistent across studies. Importantly, it has become clear that indels or point mutations that impact *SHANK3* alone can lead to all of the neurobehavioral phenotypes of PMS (De Rubeis et al., 2018). The *SHANK3* gene has multiple promoters and is alternatively spliced and the number of *Shank3* isoforms can be extensive (Maunakea et al., 2010; Benthani et al., 2015). Some *de novo* microdeletions or mutations of *SHANK3* can therefore affect some but not other *SHANK3* isoforms. The genetic heterogeneity of PMS underscores the importance of studying a wide range of mutations and deletions. SHANK3 (ProSAP2) is a major scaffolding protein that forms a key structural part of the postsynaptic density of excitatory glutamatergic synapses. SHANK3 contains multiple protein-protein interaction domains that each mediates specific protein-protein interactions at synapses. Moreover, the expression and alternative splicing of Shank3 isoforms or even their subcellular distribution has been shown to be cell-type specific, activity dependent as well as regionally and developmentally regulated (Wang et al., 2014) raising the possibility that differing SHANK3 isoforms may play distinct roles in synaptic developmental and function and hence may make distinct contributions to the pathobiology of PMS.

More than a dozen isoform-specific *Shank3* mouse models have been independently generated (Table 1). As expected, these models shared some similarities but also showed significant differences in molecular, synaptic, and behavioral phenotypes. Depending on the targeted exons, alterations have been reported in motor functions, social interactions, ultrasonic vocalizations, repetitive grooming, cognitive functions, and anxiety. However, very high variability has been observed regarding the presence or the intensity of such impairments across several types of *Shank3*-deficient models or even across different cohorts of the same model. These models are based on exonic deletions that have not been reported in human and do not reflect the vast majority of known PMS cases, which are caused by deletions affecting all *SHANK3* isoforms. There was therefore an urgent need to develop an animal model with broader construct validity for PMS to fully understand the consequences of a complete deletion of *SHANK3* across the range of behavioral phenotypes, which we achieved through a deletion of exons 4-22.

**Table 1.** Summary of existing mouse models of PMS

		Strategy	Targetedexons	Domains	Expressed isoforms										Original publication	Other publications	Synonyms	Provider	Repository	Catalog
					**a**	**a(e10-12s)**	**b**	**b(e10-12s)**	**a/b(e11-12s)**	**c**	**d**	**e**	**e-1**	**f**						
1	Deletion	Ubiquitous CMV-Cre/loxP-mediated excision	Exons 4-9	Ankyrin	−	−	−	−	−	+	+	+	+	+	Bozdagi et al. (2010)	Yang et al. (2012); Bozdagi et al. (2013); Drapeau et al. (2014)	Shank3Δ ex4-9; B6(Cg)-Shank3tm1.2Bux/J	Joseph D. Buxbaum	JAX	#017890
2	Deletion	Homologous recombination (replacement of exon 4-9 by NEO cassette)	Exons 4-9	Ankyrin	−	−	−	−	−	+	+	+	+	+	Wang et al. (2011)	Bariselli et al. (2017)	Shank3e4-9; B6.129S7-Shank3tm1Yhj/J	Yong-Hui Jiang	JAX	#017442
3	Deletion	Ubiquitous MV-Cre/loxP-mediated excision	Exons 4-9	Ankyrin	−	−	−	−	−	+	+	+	+	+	Jaramillo et al. (2016)			Craig M. Powell	NA	NA
4	Deletion	Homologous recombination (replacement of exon 4-7 by NEO cassette)	Exons 4-7	Last three ankyrin repeats	−	−	−	−	−	+	+	+	+	+	Peça et al. (2011)		Shank3A	Guoping Feng	NA	NA
5	Deletion	Ubiquitous MV-Cre/loxP-mediated excision	Exon 9	Last ankyrin repeat	−	−	−	−	−	+	+	+	+	+	Lee et al. (2015)		Shank3 (Δ9)	Eunjoon Kim	NA	NA
6	Deletion	Homologous recombination (introduction of stop codon in exon 11)	Exon 11	SH3	−	+	−	+	+	−	+	+	+	+	Schmeisser et al. (2012)	Vicidomini et al. (2017); Reim et al. (2017)	Shank3αβ, Shank3Δ11	Tobias M. Boeckers	NA	NA
7	stop codon	Insertion of Neo-Stop cassette in intron 12	Exon 13	PDZ	−	−	−	−	+	+	−	+	+	+	Jaramillo et al. (2017)		Shank3E13	Craig M. Powell	NA	NA
8	Deletion	Homologous recombination (replacement of exon 13-16 by NEO cassette)	Exons 13-16	PDZ	−	−	−	−	+	−	−	+	+	+	Peça et al. (2011)	Luo et al. (2017); Copping et al. (2017)	Shank3B; B6.129-Shank3tm2Gfng/J	Guoping Feng	JAX	#017688
9	inducible Deletion	Homologous recombination (inversion of exons 13-16 and flanking with FLEx cassette) + crossing with CAGGS-CreER mice for tamoxifen rescue	Exons 13-16	PDZ	−(+)	−(+)	−(+)	−(+)	+	−(+)	−(+)	+	+	+	Mei et al. (2016)		Shank3fx/fx and Shank3fx/fx:CAGGS-CreER; STOCK Shank3tm5.1Gfng/J; B6.129(Cg)-Shank3tm5.1Gfng/J	Guoping Feng	JAX	#028800
10	Deletion	Ubiquitous CMV-Cre/loxP mediated excision	Exon 21	PRO	−	−	+	+	+	−	−	−	+	−	Bangash et al. (2011) (retracted)	Cope et al. (2016)	Shank3ΔC (Shank3Δ ex21); B6.129S6(Cg)-Shank3tm1.1Pfw/J; B6.Cg-Shank3tm1.1Pfw/J; STOCK Shank3tm1.1Pfw/J	Paul Worley	JAX	#018398
11	Deletion	Ubiquitous CMV-Cre/loxP-mediated excision	Exon 21	PRO	−	−	+	+	+	−	−	−	+	−	Kouser et al. (2013)	Kloth et al. (2015); Duffney et al. (2015); Bidinosti et al. (2016); Li et al. (2017)	Shank3ΔC/ΔC	Craig M. Powell	NA	NA
12	inducible point insertion	Insertion of a floxed mutated exon 21 followed by a transcriptional stop (Neo-stop) cassette + crossing with B6.Cg-Tg(CAG-cre/Esr1*)5Amc/J for tamoxifen rescue	Exon 21	PRO	−	−	+	+	+	−	−	−	+	−	Speed et al. (2015)		Shank3G/G and Reversible-Shank3GCre+	Craig M. Powell	NA	NA
13	point insertion	Homologous recombination (G insertion at position 3680 causing a frameshift and premature stop codon)	Exon 21	PRO	−(+)	−(+)	+	+	+	−(+)	−(+)	−(+)	+	−(+)	Zhou et al. (2016)		Shank3*G3680 knock-in; STOCK Shank3tm3.1Gfng/J	Guoping Feng	JAX	#028778
14	point mutation	Homologous recombination (R1117X nonsense mutation)	Exon 21	PRO	−	−	+	+	+	−	−	−	+	−	Zhou et al. (2016)		Shank3*R1117X knock-in; STOCK Shank3tm4.1Gfng/J	Guoping Feng	JAX	#028779
15	Deletion	Ubiquitous CMV-Cre/loxP-mediated excision	Exons 4-22	ANK, SH3, PDZ, PRO, SAM	−	−	−	−	−	−	−	−	−	−	Wang et al. (2016)	Han et al. (2016)	Shank3Δe4–22	Yong-Hui Jiang	NA	NA
16	over-expression	EGFP–Shank3 BAC transgenic mice	Full gene		++	++	++	++	++	++	++	++	++	++	Han et al. (2013)		Tg(Shank3-EGFP)1Hzo; B6.FVB-Tg(Shank3-EGFP)1Hzo/J	Huda Y Zoghbi	JAX	#024033

1-5: targeted deletions in the ankyrin repeat domain. Δ4-7: deletion of exons 4 to 7; Δ4-9: deletion of exons 4 to 9; Δ9: deletion of exon 9. 6: targeted deletion in the SH3 (Src Homology 3) domain. Δ11: deletion of exon 11. 7-9: targeted deletions in the PDZ (PSD95/Discs large/zona-occludens-1) domain. Δ13: deletion of exon 13; Δ13-16: deletion of exon 13 to 16). 10-14: targeted deletions of point mutations in the proline-rich domain. Δ21: deletion of exon 21. 15: deletion of all functional domains. Δ4-22: deletions of exons 4 to 22. 16: overexpression of the full Shank3 gene.

Interestingly, as our work was progressing, a completely independent mouse model, similarly targeting exons 4-22, was reported (Wang et al., 2016b). These mice highlight cortico-striatal circuit abnormalities and demonstrate a behavioral phenotype that resemble features of PMS. We therefore decided to conduct a comprehensive and behavioral evaluation of our mouse model evaluating many more phenotypes relevant to PMS and ASD. Critically, our findings complement and supplement the observations made by the Jiang group with many results clearly confirmed across two independent laboratories, as well as unique analyses in each study.

## Materials and Methods

### Generation of inbred strains of *Shank^Δ4-22^*-deficient animals

**Figure 1.**
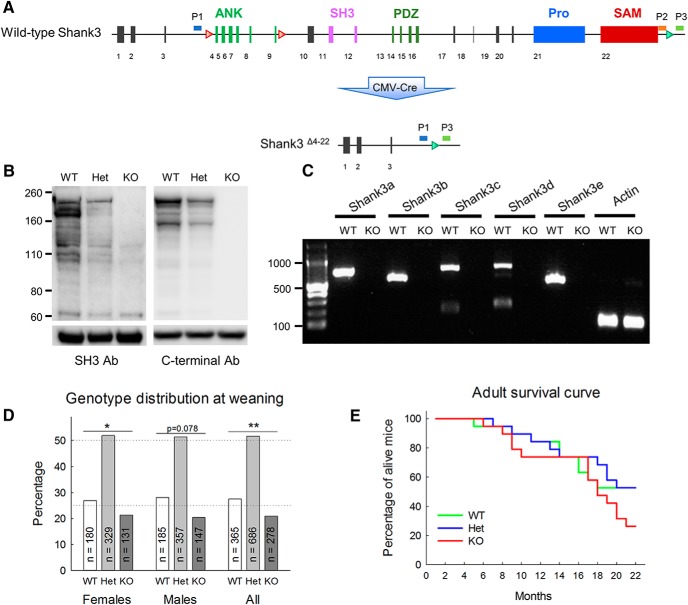
Generation and validation of a knock-out mice with a complete deletion of Shank3. ***A***, Schematic design for generation of a *Shank3^Δ4-22^* complete knock-out mouse using a Cre-loxP strategy. Bruce4 C57BL/6 embryonic stem cells from a previously generated mouse with two LoxP site located upstream exon 4 and downstream exon 9 (top, red triangles) were retargeted to insert an additional LoxP site 155 pb downstream of exon 22 (green triangle). Floxed mice were crossed with CMV-Cre mice to generate ubiquitous deletion of exons 4–22 (bottom). ANK, ankyrin repeats; SH3, Src homology 3 domain; PDZ, PSD/Dlg1/zo-1 domain; Pro, proline-rich domain; SAM, sterile α-motif domain. The positions of the PCR primers (P1, P2, P3) for genotyping are indicated. ***B***, Expression of Shank3 in PSD fractions. PSD fractions from wild-type, heterozygous, and homozygous mice were subjected to immunoblotting with either the N367/62 anti-Shank3 antibody directed against an epitope in the SH3 domain or the H160 C-terminal antibody. Immunoblots show that all Shank3 protein bands are absent in KO brains. The migration of molecular weight markers is shown on the left (in kilodaltons) and an immunoblot for βIII-tubulin as a loading control is shown below. Original full scans of immunoblots are displayed in Extended Data Figure 1-1. ***C***, RT-PCR analysis for specific Shank3 transcripts in *Shank3^Δ4-22^* mice. Brain-derived mRNAs from wild-type and homozygous mice were subjected to RT-PCR targeting different isoforms. All transcripts were absent in *Shank3^Δ4-22^* homozygous mice. ***D***, Distribution of genotype. A deficit in the number of *Shank3^Δ4-22^* knockout mice was observed at the time of weaning. ***E***, Survival curve of *Shank3^Δ4-22^* wild-type, heterozygous and homozygous mice between 2 and 22 months. WT, wild-type mice; Het, heterozygous mice; KO, homozygous knockout mice. *: *p* < 0.05, **: *p* < 0.1.

All animal procedures were approved by the Institutional Animal Care and Use Committee of the Icahn School of Medicine at Mount Sinai. A *Shank3^Δ4-22^* mouse line with a complete disruption of the *Shank3* gene was generated at Ozgene by retargeting Bruce4 C57BL/6 embryonic stem cells from a previously published mouse. A third loxP site was inserted immediately downstream of exon 22 in addition of the 2 pre-existing loxP sites flanking exons 4 and 9 (Fig. 1*A*). To generate the mice used in the present study, the floxed allele was excised by breeding with a CMV-Cre transgenic line (Tg(CMV-cre)1Cgn, The Jackson Laboratory, #006054) resulting in a deletion of exons 4-22 and therefore a constitutive disruption of all the Shank3 murine isoforms. Both the floxed and deleted mouse strains are available at The Jackson Laboratory Repository (Shank3^Δ4-22^ floxed strain: JAX Stock No. 032158; Shank3^Δ4-22^ deleted strain: JAX Stock No. 032169; http://jaxmice.jax.org/query).

10.1523/ENEURO.0046-18.2018.f1-1 Extended Data Figure 1-1 **Validation of a knockout mice with a complete deletion of Shank3** (A) Genotyping of Shank3^∆4-22^ mice by PCR. The P1-P3 primer pair produced a 490 bp band identifying the ∆4-22 allele, while the P2-P3 primer pair amplified the 390 bp product from the wild-type allele. (B) Original full scans of immunoblots related to Figure 1. WT, wild-type mice; Het, heterozygous mice; KO, homozygous knockout mice. Download Figure 1-1, TIF file.

The colony was maintained on a pure C57BL/6Tac background (Taconic). Heterozygous mice were mated to generate litters consisting of three genotypes, wild-type (WT), heterozygote (Het), and knock-out (KO). Mice were weaned at 21 d of age, and at least one littermate from each genotype were group housed in standard plastic cages of three to five littermates per cage. Standard rodent chow and tap water were available ad libitum. The colony room was maintained on a 12/12 h light/dark cycle with lights on at 6 A.M. at a constant temperature of 21–22°C and 55% humidity. All animal procedures were performed in accordance with the Icahn School of Medicine at Mount Sinai animal care committee’s regulations.

### Genotyping

The confirmation of the deletions of all Shank3 isoforms was performed by RT-PCR. All the animals included in this study were genotyped using tail samples collected at the time of weaning. Additionally, the genotype of all the adult animals was confirmed using a supplementary biopsy at the end of the behavioral testing. Mouse tail snips were collected by dissecting 0.2 cm of tail between postnatal days 15 and 21. Tails were digested, genomic DNA isolated and purified using the QIAGEN DNAeasy kit (QIAGEN) according to the manufacturer’s instructions. After the extraction, 2.0 μl of DNA in buffer containing ∼250–400 μg of DNA was amplified by PCR using standard PCR methods and a combination of three primers designed inside and outside the deleted region to identify both the wild-type and Δe4-22 alleles (Fig. 1; Extended Data Fig. 1-1; P1-KO: TGAGACCAGAGTTGTTAGGATTTG, P2-WT: AGATGGCTCAGCCAGGTAAG, P3-Common AGATGGCTCAGCCAGGTAAG). The P1-P3 primer pair produced a 490-bp band identifying the Δe4-22 allele, while the P2-P3 primer pair amplified a 390-bp band from the wild-type allele. Denaturing, annealing, and extension steps were performed using 94°C for 3 min, 35 cycles of 94°C for 30 s, 62°C for 45 s, 45°C for 30 s, and for 1 cycle 72°C for 4 min. The PCR products were run on a 1.5% agarose gel and stained with ethidium bromide.

### Immunoblotting

Postsynaptic density (PSD) fractions were prepared as follows. Hemibrains of wild-type, heterozygous, and homozygous *Shank3^Δ4-22^* mice were homogenized in 2-[4-(2-hydroxyethyl)piperazin-1-yl]ethanesulfonic acid (HEPES)-A containing 4 mM HEPES, pH 7.4, 0.32 M sucrose, and Protease Inhibitor Cocktail and PhoSTOP Phosphatase Inhibitor Cocktail (both from Roche). Nuclear fractions were precipitated by centrifuging twice at 700 × *g* for 15 min, and the resulting supernatants were further centrifuged at 21,000 × *g* for 15 min. The precipitates were resuspended in HEPES-B containing 4 mM HEPES, pH 7.4, Protease Inhibitor Cocktail, and PhoSTOP Phosphatase Inhibitor Cocktail, homogenized, and rotated at 4°C for 1 h. The lysates were centrifuged at 32,000 × *g* for 20 min and washed twice with HEPES-C containing 50 mM HEPES, pH 7.4, 0.5% Triton X-100, Protease Inhibitor Cocktail, and PhoSTOP Phosphatase Inhibitor Cocktail. Finally, PSD fractions were resuspended in HEPES-C containing 1.8% sodium dodecyl sulfate (SDS) and 2.5 M urea. Fifty micrograms of PSD fraction was loaded to 4–12% SDS-PAGE (PAGE gel, Invitrogen), transferred to polyvinylidene fluoride membrane and immunoblotted with either the N367/62 anti-Shank3 antibody directed against an epitope in the SH3 domain (UC Davis/NIH NeuroMab Facility) or the H160 anti-Shank3 antibody directed against amino acids 1431–1590 mapping near the C terminus of isoform 2 of Shank 3 (sc-30193, Santa Cruz Biotechnology). For βIII-tubulin, the membrane was stripped and immunoblotted with an anti-βIII-tubulin antibody (Abcam).

### RT-PCR isoform analysis

Total RNA from hemibrains of wild-type and homozygous *Shank3^Δ4-22^* mice was isolated using the TRIzol method (Invitrogen, ThermoFisher Scientific). Reverse transcription was performed with SuperScript III first-strand synthesis system (Invitrogen, ThermoFisher Scientific). DNA was amplified by PCR using standard PCR methods and the following primers ass described previously (Wang et al., 2014). Shank3a forward: ACGAAGTGCCTGCGTCTGGAC, Shank3a reverse: CTCTTGCCAACCATTCTCATCAGTG; Shank3b forward: GTAGCCACCTCTTGCTCACAT, Shank3b reverse: TTGCCAACCATTCTCATCAGT; Shank3c forward: CTTCTTCACTGGCAATCCTTG, Shank3c reverse: CAGTGTAGTGGCGGAAGAGAC; Shank3d forward: AGGGTCACGACTGTTTCTTAGC, Shank3d reverse: TGTGGGTGTAAACTCCTCAATG; Shank3e forward: GTACCTGGGTCTGGGTGCTTTA, Shank3e reverse: AACTGCCAGGATCTCATCCA.

### Behavioral overview

Multiple cohorts were used for behavioral testing. The first cohort consisted of 54 newborn mice (14 WT, 30 Het and 10 KO) from 10 independent litters. The second cohort consisted of 57 newborn mice (16 WT, 32 Het, and nine KO) from nine independent litters. Cohorts 3 (30 adult male mice, 11 WT, 10 Het, and nine KO) and 4 (27 adult male mice, 11 WT, 10 Het, and nine KO) were tested between 3 and 10 months of age according to the schedule described in Table 2. In each adult cohort, all mice were born within two weeks of each other, and generally only one triplet came from any given individual litter of mice. Behavioral experiments were conducted between 9 A.M. and 5 P.M. during the light phase of the 12/12 h light/dark cycle in dedicated testing sound-attenuated rooms. Mice were brought to the front room of the testing area at least half an hour before the start of experiments. All three genotypes were tested on the same day in randomized order by two investigators who were blind to the genotypes. Behavioral tests were conducted in the order and at the ages indicated in Table 2 and included developmental milestones, cage observation, neurologic and motor reflexes, open field, elevated zero-maze, Y-maze, beam walking, grip strength, gait analysis, rotarod, three-chambered social interaction task, nest building, novel object recognition, fear conditioning, pre-pulse inhibition, tail flick, olfactory habituation/dishabituation, buried food, social transmission of food preference, marble burying, four-object repetitive novel object contact task, male-female social interaction, and Barnes maze. Behavioral results are not described in the order they were tested in an effort to ease presentation and interpretation of the data.

### Newborn development

The physical, sensory and motor developmental milestones of neonates were assessed between postnatal days 1 and 21 using a battery of tests adapted from the Fox scale (Fox, 1965; Heyser, 2004). As we had previously observed, a higher rate of postnatal mortality on the first litter, only dams that already had one litter were used for this experiment. To control for litter and avoid nutritional effects the litter size was homogenized and limited to six pups per dam by reducing larger litters and adding excess pups to smaller litters on the morning of postnatal day 1 where and when possible. At this time, pups were identified by paw tattoo using a nontoxic animal tattoo ink (Animal Identification & Marking Systems Inc) inserted subcutaneously through a 30-gauge hypodermic needle tip into the center of the paw. Individual pups were removed from the litter and placed on cotton pads in a heated cage under a heating lamp throughout the testing. Each subject was tested at approximately the same time of day. For all the timed tests, a 30-s cutoff was used and nonresponding animal received a score of 30 s. Most responses were considered positive only after they had been observed for two consecutive days.

The physical development was measured by following the weight (postnatal day 1 to 21), eye opening (postnatal days 9 to 20), tooth eruption (postnatal days 7 to 18), the ear development (postnatal day 1 to 9), and the fur development (postnatal days 1 to 14) using the following scales. Eye opening, per eye: 0 = eye fully closed, 1 = eye partially opened, 2 = eye full opened, tooth eruption, scored separately for bottom and top incisors: 0 = incisors not visible, 1 = incisors visible but not erupted, 2 = incisors fully erupted. Ear development, per ear: 0 = ear bud not detached from the pinna, 1 = ear flap detached from the pinna, ear fully developed on the back of the ear). Fur development: 1 = bright red, 2 = nude, pink, 3 = nude, gray, 4 = gray, fuzzy on back and shoulder, 5 = black hair on back, gray fuzzy belly, 6 = body fully covered.

Sensory development was assessed using cliff aversion (postnatal days 2 to 14), auditory startle (postnatal days 6 to 18), rooting reflex (postnatal days 2 to 10), ear twitch (postnatal days 7 to 15), and forelimb grasp (postnatal days 4 to 14) using the following measures. For cliff aversion, the subject was placed on the edge of a Plexiglas platform with a 30-cm cliff with its nose and forefeet over the edge. The latency to move away from the edge was recorded. Auditory startle was measured in response to an 80-dB click 30 cm above the mouse and was considered present when the pup moved immediately after the presentation of the auditory stimulus. For the rooting reflex, the side of the pup’s face were bilaterally stimulated with two cotton swabs. The reflex was considered present when the pup crawled forwards pushing the head during the stimulation. For the ear twitch, the ear of the pup was stimulated with the tip of a cotton swab that was previously pulled to form a filament. Both ears were successively stimulated and the test was considered positive when the pup turned its head or jumped in response to the stimulation. The forelimb reflex was tested by gently stimulated the front paws with the loop of a small bended metallic wire. Each front paw was scored separately as follow: 0 = no response to stimulation, 1 = paw folding in response to the stimulation, 2 = paw grasping the wire in response to the stimulation, 3 = grasp strong enough to hold for at least 1 s when the wire was lifted up.

Motor development was studied using surface righting (postnatal days 2 to 13), negative geotaxis (postnatal days 2 to 14), air righting (postnatal days 8 to 20), open field crossing (postnatal days 8 to 20), and rod suspension (P11–P20) using the following criteria. The surface righting was measured by the time for pups placed on their back to fully turn with all four paws on the ground. For negative geotaxis, pups were placed head down on a mesh covered plan that was slanted at a 45° angle, and the latency to either roll down, stay, or turn and move up the slope was recorded. For the air righting, the pup was dropped upside down at a height of 30 cm over a padded surface. Subjects received a score of 2 if they successfully righted themselves during the fall, 1 if they landed on the side and 0 if they did no turn. The open field crossing was measured by the time to exit a 13 cm in diameter circle when place on the center of the circle. For the rod suspension, the pups were gently grabbed by the trunk, brought up close to a 3-mm wooden rod 30 cm above a padded surface and released once they grabbed the rod with their front paws. The latency to stay suspended was recorded.

### Physical factors, gross appearance, and spontaneous activity

Adult animals were handled daily for one week before starting behavioral testing and general health, weight (grams), length (centimeters), physical factors, gross appearance, and spontaneous activity were recording during handling using the following scales.

#### Physical factor and gross appearance

Coat appearance: 0 = ungroomed, 1 = partially groomed, 2 = semi-groomed, 3 = groomed. Skin color (pinna and footpads): 0 = pink, 1= purple, 2 = other. Whisker barbering: 0 = normal, 1 = abnormally shortened. Patches of missing fur on face or body: 0 = none, 1 = some, 2 = extensive. Wounding: 0 = none, 1 = signs of previous wounding, 2 = slight wounds present, 3 = moderate wounds present, 4 = extensive wounds present. Body tone when both sides of the mouse are compressed between thumb and index finger: 0 = flaccid, no return of cavity to normal, 1 = slight resistance, 2 = extreme resistance. Palpebral closure: 0 = eyes wide open, 1 = eyes half open, 2 = eyes closed. Spontaneous piloerection: 0 = none, 1 - coat standing on end.

Spontaneous general activity in a 1000-ml jar and after transfer in a regular home cage for 5 min each. Body position: 0 = completely flat, 1 = lying on side, 2 = lying prone, 3 = sitting or standing, 4 = rearing on hind legs, 5 = repeated vertical leaping. Spontaneous activity: 0 = none, resting, 1 = casual scratch, groom, slow movement, 2 = vigorous scratch, groom, moderate movement, 3 = vigorous, rapid/dart movement, 4 = extremely vigorous, rapid/dart movement. Respiration rate: 0 = gasping, irregular, 1 = slow, shallow, 2 = normal, 3 = hyperventilation. Tremor: 0 = none, 1 = mild, 2 = marked. Urination: 0 = none, 1 = little, 3 = moderate amount, 4 = extensive. Defecation: number of fecal boli. Transfer arousal: 0 = coma, 1 = prolonged freeze, then slight movement, 2 = brief freeze, then active movement, 3 = no freeze, stretch attends, 4 = no freeze, immediate movement (manic). Gait: 0 = normal, 1 = fluid but abnormal, 2 = slow and halting, 3 = limited movement only, 4 = incapacity. Pelvic elevation: 0 = markedly flattened, 1 = barely touches, 2 = normal (3 mm elevation), 3 = elevated (more than 3 mm elevation). Tail elevation: 0 = dragging, 1 = horizontally extended, 2 = <30° elevation, 3 = 30–60° elevation, 4 = 60–90° elevation.

### Motor testing

#### Gait analysis

Motor coordination and gait patterns was observed as the subject was allowed to run the length of an elevated runway (dimensions: 152 cm long × 10 cm wide) lined with white paper (Carter et al., 2001). After three training runs, the subject’s paws were coated in nontoxic paint (different colors for hind and front paws) to record paw prints on two consecutive runs. The record displaying the clearest prints and most consistent gait for analysis of 50 cm was chosen to measure sway (mean distance between left and right paws), stride (mean distance between same side front and hind paws) and diagonal stance (mean distance between diagonally opposed front and hind paws).

#### Open field

Mice were tested in an open field (45 × 45 cm) virtually divided into central and peripheral regions. Animal activity was recorded by video tracking (Noldus Ethovision). Each mouse was allowed to explore the apparatus for 60 min. The distance traveled, the number of rears and revolutions, the number of grooming bouts and cumulative grooming time, the number of head shaking or twitches, the number of entries in the center, and the time spent in the central and peripheral regions were recorded. Measures were recorded in 10-min intervals.

#### Rotarod

Motor coordination, endurance and learning was assessed in the Rotarod test (Omnitech Electronics Inc). Mice were placed on an elevated accelerating rod (3 cm in diameter) for three trials per day on two consecutive days. Each trial lasted for a maximum of 5 min, during which the Rotarod underwent a linear acceleration from 4 to 40 rpm. A 20-min interval was used between trials to avoid fatigue. Animals were scored for their latency to fall.

#### Beam walking

Subtle deficits in fine motor coordination and balance that might not be detected by other motor tests were assessed by the beam walking assay in which the mouse had to walk across an elevated horizontal wood beam (100 cm long, 1 m above bedding) to a safe dark box (Carter et al., 2001). Subjects were placed near one end in bright light, while the far end with the dark box was placed in darkness, providing motivation to cross. Performance was quantified by measuring the latency to start crossing, the time to reach the dark box or the time to fall, the total distance traveled and the number of paw slips or incomplete falls (mice able to climb back on the rod). Animals were successively trained on three different beams: 1 inch, ½ inch and ¼ inch diameter and scored on four consecutive trials per beam with 1 min of rest between trials on the same beam and 20–30 min between each beam. Mice that did not reach the box after 2 min were gently placed inside the box and allowed to stay inside for 1 min.

#### Righting reflex

The subject was grasped by the nape of the neck and base of the tail, inverted so back faced down, and released 30 cm above subject’s home cage floor. Righting ability was scored as follow: 0 = no impairment, 1 = lands on side, 2 = lands on back, 3 = fails to right even when placed on back on the floor.

#### Hindlimb placing

Subject was lowered by the base of the tail until it grasped a horizontal wire grid with both forepaws. The grid was rotated to vertical and the tail was released. Mice were evaluated over three trials, 3 min apart for their latency to fall or latency to pull body on the grid and the ability to place hind paws was scored as follow: 0 = grabs but falls, 1 = grabs but hangs, 3 = grabs and pulls body onto grid. Maximum cutoff was 60 s.

#### Hanging

The subject, held from the base of the tail, was allowed to grasp a wooden rod with both forepaws, rotated to horizontal and release. Test was repeated three times with a 3-min interval between trials and a 60-s maximum cutoff. Both the latency to fall and overall performance scored as follow were recorded: 0 = does not grasp, 1 = grasps but falls immediately, 2 = grasps but then falls off, 3 = grasps and stays on for 60 s.

#### Negative geotaxis

The subject was placed on a wire mesh grid and the grid was lift vertically, with subject facing down. Test was repeated three times with a 3-min interval between trials and a 60-s maximum cutoff. Both the latency to fall and overall performance scored as follow were recorded: 0 = falls off, 1 = does not move, 2 = moves but does not turn, 3 = turns but does not climb, 4 = turns and climbs up.

#### Inverted screen

The subject was placed on a grid screen. The grid was waved lightly in the air, then inverted 60 cm over a cage with soft bedding material. Mice were tested only one time with a 60-s maximum cutoff, and the latency to fall was recorded.

#### Grip strength

Forelimb muscle strength and function was evaluated with a strength meter (Ametek). This test relies on the instinctive tendency of mice to grasp an object with their forelimbs. The animal was pulled backward gently by the tail, while grasping a pull bar connected to a tension meter and the force at the moment when the mouse lost its grip was recorded as the peak tension. Test was repeated three times with a 3-min interval between trials. Each trial consisted in five attempts in quick successions for which the best value was recorded therefore increasing the chances that the measure will accurately reflect maximum strength. The mean of three trials and the largest value from all trials were used as parameters.

### Sensory testing

#### Sensory reflexes

Sensory abilities were evaluated through the reflex response to several sensory modalities using the following scales. Pinna reflex in response to a gentle touch of the auditory meatus with a cotton-tipped applicator repeated three times with a 10- to 15-s interval: 0 = none, 1 = active retraction, moderately brisk flick, 2 = hyperactive, repetitive flick. Corneal reflex in response to a gentle puff of air repeated three times with a 10- to 15-s interval: 0 = no eye blink, 1 = active eye blink, 2 = multiple eye blink. Toe pinch normal retraction reflexes in all four limbs when lightly pinching each paw successively by applying a gentle lateral compression with fine forceps while the mouse is lifted by its tail so the hind limbs are clear of the table. Score is cumulative of four limbs: 0 = no retraction, 1 = active retraction, 2 = repetitive retractions. Preyer reflex in response to a 90-dB click 30 cm above mouse repeated three times with a 10- to 15-s interval: 0 = None, 1 = Preyer reflex (head twitch), 2 = jump <1 cm, 3 = jump >1 cm.

#### Tail flick test

The automated tail flick test (Omnitech Electronics Inc) was used to assess nociceptive threshold. Awake mice were placed in a contention tube to limit movement with their tail resting on the groove of a heating panel. When the mice were calm, a narrow heat producing beam was directed at a small discrete spot ∼15 mm from the tip of the tail. When the subject’s tail was removed from the beam, an automatic timer recorded the latency. The test was repeated five times with a 3-min interval between each trial. The latency of the mice to flick their tail was recorded and the two trials with the shorter latencies were discarded since the tail is not always fully in the beam and this is often an outlier.

#### Acoustic startle response and pre-pulse inhibition of startle

Subjects were placed in isolation boxes outfitted with accelerometers to measure magnitude of subject movement (Med Associates). After 5 min of acclimation mice were first tested for acoustic startle response. Mice were presented with six discrete blocks of six trials over 8 min, for a total of thirty-six trials. The trials consisted in six responses to no stimulus (baseline movement), six responses to 40-ms sound bursts of 74 dB, six responses to 40-ms sound bursts of 78 dB, six responses to 82-ms sound bursts of 100 dB, five responses to 40-ms sound bursts of 86 dB, and six responses to 40-ms sound bursts of 92 dB. The six trials type were presented in pseudorandom order such that each trial type was presented once within a block of six trials. Mice were then tested for pre-pulse inhibition of startle. They were presented with seven discrete blocks of trials of six trials over 10.5 min for a total of 42 trials. The trials consisted in six response to no stimulus (baseline movement), six startle response to a 40-ms, 110-dB sound burst, six prepulse inhibition trials where the 110-dB tone was preceded by a 20-ms 74-dB tone 100 ms earlier, six prepulse inhibition trials where the 110-dB tone was preceded by a 20-ms 78-dB tone 100 ms earlier, six prepulse inhibition trials where the 110-dB tone was preceded by a 20-ms 82-dB tone 100 ms earlier, six prepulse inhibition trials where the 110-dB tone was preceded by a 20-ms 86-dB tone 100 ms earlier and six prepulse inhibition trials where the 110-dB tone was preceded by a 20-ms 92-dB tone 100 ms earlier. The seven trial types were presented in pseudorandom order such that each trial type was presented once within a block of seven trials. Startle amplitude was measured every 1 ms over a 65-ms period, beginning at the onset of the startle stimulus. The intertrial interval was 10–20 s. The maximum startle amplitude over this sampling period was taken as the dependent variable. A background noise level of 70 dB was maintained over the duration of the test session.

#### Visual acuity

Visual acuity was tested using the visual placing test that takes advantage of the forepaw-reaching reflex: the mouse was held by its tail ∼20 cm above the surface and progressively lowered. As it approaches the surface, the mouse should expand its forepaws to reach the floor. The test was repeated three times with a 30-s interval and the forepaw reaching reflex was quantified as the percentage of forepaw-reaching episodes that did not involve the vibrissae and/or nose touching the surface before the forepaws.

#### Buried food test

The buried food test (Yang and Crawley, 2009) measures how quickly an overnight-fasted animal can find a small piece of familiar palatable food, that is hidden underneath a layer of bedding using olfactory clues. Fruit Loops (Kellog’s) were used as familiar food. For three consecutive days before the test, three to four pieces were offered to the subjects to make sure it was highly palatable for all the subjects. At 18–24 h before the test, all chow pellets were removed from the subjects’ home cages. The water bottle was not removed. On the testing day, the subject was placed in a clean cage (28 cm long × 18 cm wide × 12 cm high) containing 3 cm deep of clean bedding and the subject was allowed to acclimate to the cage for 10 min. While the subject was temporary placed in an empty clean cage, four to five pieces of Fruit Loops were buried ∼1 cm beneath the surface of the bedding, in a random corner of the cage and the bedding surface was smoothed out. The subject was placed back in the testing cage and given 15 min to retrieve and eat the hidden food. Latency to find the food was recorded. If a subject did not find the food, 15 min was recorded as its latency score and the food was unburied and presented to the mouse by the experimenter to make sure that it was palatable for the mouse. At the end of testing, subjects were hold in a temporary cage until all animals from the same home cage were tested.

#### Olfactory habituation and dishabituation

This test consisted of sequential presentations of different nonsocial and social odors in the following order: water, lemon extract (McCormick; 1:100 dilution), banana extract (McCormick; 1:100 dilution), unfamiliar males and unfamiliar females (Yang and Crawley, 2009). Lemon and banana solutions were freshly prepared everyday using distilled water. Social odors were obtained from cages of unfamiliar C56BL/6 mice of the same and opposite sex as the subject which have not been changed for at least 3 d and were maintained outside of the experimental testing room. Social odor stimuli were prepared by wiping a cotton swab in a zigzag motion across the cage. The subject was placed in a clean bedding-covered testing cage covered with the cage grid. A clean dry applicator (10-cm cotton swab) was inserted through the cage grid water bottle hole and the animal was allowed to acclimate for 30 min to reduce novelty-induced exploratory activity during the olfaction test. Each odor (or water) was presented in three consecutive trials for a duration of 2 min. The intertrial interval was 1 min, which is about the amount of time needed to change the odor stimulus. At the end of testing, subjects were hold in a temporary cage until all animals from the same home cage were tested. The test was videotaped and subsequently scored. Sniffing and direct interaction time (touching, biting, climbing the applicator) were quantified separately.

### Social tests

#### Three-chambered social approach test

Sociability and preference for social novelty and social recognition were tested in a three-chambered apparatus (Nadler et al., 2004). The subject mouse was first placed in the central, neutral chamber and allowed to explore for 10 min with all doors closed. Next, doors were opened and the mouse was allowed to freely explore the three empty chambers for an additional 10 min. Lack of side preference was confirmed during this habituation. The subject was then temporary placed in a holding cage while two empty wire cages which allow for olfactory, visual, auditory, and tactile contacts but not for sexual contact or fighting containing either an inanimate object (black cone) or a male mouse were placed in each of the testing chambers and the subject was returned to the apparatus for a 10-min testing phase. Adult mice from the same strain that was previously habituated to the wire cup and did not exhibit aggressive behaviors but had no previous contact with the subject were used for unfamiliar mice. Unfamiliar mice were not used more than twice a day with at least 2 h before two tests. At the end of testing, subjects were hold in a temporary cage until all animals from the same home cage have been tested. The side position of the interacting animal and the object was randomly determined. All the sessions were videotracked (Noldus Ethovision) and the amount of time spent in each chamber, close to the holding cages or in direct interaction with the holding cage was automatically calculated.

#### Male-female social interaction

Male-female social interactions were evaluated in in a regular clean cage during a 10-min test session as previously described (Scattoni et al., 2011). Each subject male was paired with an unfamiliar estrus C57BL/6J female under low light (10 lux) conditions. A total of 20 females were used for this test allowing to avoid to reuse the same female more than twice on the same day. The sessions were videotaped and ultrasonic vocalizations were recorded using an ultrasonic microphone with a 250-kHz sampling rate (Noldus Ultravox XT) positioned 10 cm above the cage. The entire set-up was installed in a sound-attenuating room. Videos from the male subjects were subsequently manually scored to quantify (number of events and total time of male to female nose-to-nose sniffing, nose-to-anogenital sniffing, and sniffing of other body regions. Ultrasonic vocalizations were played back and spectrograms were displayed using the Ultravox XT software and ultrasonic vocalizations were manually quantified.

#### Social transmission of food preference

The social transmission of food preference is a test of olfaction memory that involves a social component through the use of a demonstrator mouse (Wrenn et al., 2003). The demonstrator mouse is a conspecific mouse of same sex and similar age that was labeled by bleaching before testing. To minimize neophobia during the experiments, both subjects and demonstrator mice were habituated to eat powdered rodent chow (AIN-93M, Dyets, Inc.) from 4-oz (113.40-g) glass food jar assemblies (Dyets, Inc.). This habituation was performed for 48 h in the mice home cage while the regular pellet chow was removed from the cages. After the habituation, both subject mice and demonstrator mice were food deprived for 18–24 h before testing with free access to water. The test was divided into three phases.

##### Demonstrator exposition

During the first phase the demonstrator was presented with a jar of powder food mixed with either 1% cinnamon or 2% cocoa. The flavor was randomly assigned to the demonstrators so half of them received the cocoa flavored food while the other half received the cinnamon flavored food. Each demonstrator was used only once a day. The demonstrators were allowed to eat the flavored food for 1 h. The jars were weighed before and after presentation to the demonstrators. The criterion for inclusion in the experiment was consumption of 0.2 g or more.

##### Interaction phase

After eating the flavored food, a demonstrator was placed in an interaction cage with the observer subject mouse and mice were allowed to freely interact for 30 min.

##### Choice phase

Immediately after the interaction phase, the observer mouse was placed in a clean cage and presented with one jar containing the flavor of food eaten by the demonstrator (cued) and another jar containing the other flavor and given 1 h to freely explore the jar and eat. The demonstrator flavor and the position of the jar (front or back of the cage) was randomly assigned.

All phases were videotaped and food jars were weighed before and after the sessions to determine the amount of food eaten. At the end of testing, demonstrators and observers were hold in temporary cages until all animals from the same home cage had been tested. Video recordings from the interaction phase were used to score the number and total time of sniffing bouts from the observer to the nose or head of the demonstrator. Video recordings from the choice phase were used to score the total time spent in interaction with each food jar (mouse observed in the top of the jar with nose in jar hole).

### Avoidance, escape behavior, and hyper-reactivity

Object avoidance and escape behavior was observed in several tests initially designed to assess other behaviors, including the novel object recognition, the marble burying, and the nest building.

#### Novel object recognition

The novel object test for object recognition and memory takes place in an opacified open field arena (45 × 45 cm). The test involves a set of two unique novel objects, each about the size of a mouse, constructed from two different materials and nonuniform in shape. The test consisted of one 10-min habituation session, a 5-min familiarization session and a 5-min recognition test, each videotracked (Noldus Ethovision). During the habituation, animals were allowed to freely explore an empty open field. At the end of the session, they were removed from the open field and place in a temporary clean holding cage for about 2 min. Two identical objects were placed on the median line at ∼10 cm from each wall and the animal was returned to the open field and allowed to explore the objects for 5 min before being returned to its home cage. After 1 h, one familiar object and one novel object were placed in the open field to the location where the identical objects were placed during the familiarization session and the mouse was allowed to explore them for a 5-min recognition test. The side of the novel object position was randomly assigned so half of the animals were exposed to a novel object placed on the right of the open field and half of the animals were exposed to a novel object placed on the left of the open field.

Between each session, the open field and the objects were carefully cleaned with 70% ethanol and let dry. Familiarization and recognition sessions were scored for total time spent investigating each object, the number of object interactions and the latency o the first object interaction. Time spend in each side during habituation and familiarization and time spent sniffing two identical objects during the familiarization phase were used to examine an innate side bias. Total time spent sniffing both objects was used as a measure of general exploration.

#### Marble burying test

The marble-burying assay is a tool for assessing either anxiety-like and/or repetitive-like behaviors in mice (Thomas et al., 2009). Subjects were tested in a regular clean cage (28 cm long × 18 cm wide × 12 cm high) with 3 cm of fresh bedding. The subject was first placed in the empty cage for a 5-min habituation. It was then temporary placed in an empty clean cage while 20 dark blue glass marbles (15 mm in diameter) were positioned over the bedding equidistant in a 4 × 5 arrangement to cover the whole cage surface. The subject was then returned in the test cage and allowed to explore and bury the marbles during a 15-min session that was videotaped. At the end of the session the subject was removed and the number of marbles buried (>50% marble covered by bedding material) was recorded.

#### Nest building

For small rodents, nests are important for heat conservation as well as for reproduction and shelter (Deacon, 2006). Mice were initially single housed in cages containing no environmental enrichment items such as bedding, cardboard houses or tunnels. To test their ability to build nests animals were temporarily single housed. One hour before the dark phase, any building material present in the home cage was removed and replaced by two cotton nestlets (Ancare, NES3600 nestlets). The test was repeated twice and scored on the next morning of the second repeat using the following multicriteria scale adapted from (Deacon, 2006; maximum score = 11): nestlet shredding: 0 = not at all, 1 = partially, 2 = fully shredded; nestlet dispersion: 0 = nestlet dispersed all over the cage, 1 = mostly used to build nest, 2 = fully used to build a nest; nest density: 0: not dense, 1 = medium density, 2 = high density; nest shape: 0: no nest, 1 = ball shape, 2 = nest shape but no bottom, 3 = full nest; presence of walls: 0 = no walls, 1 = partial walls, 2 = nest fully surrounded by walls; maximum score = 11.

#### Escape behavior

Escape behavior evaluated in three different tests all taking place in regular home cages (28 cm long × 18 cm wide × 12 cm high) by counting the number of unsuccessful (mouse climbing on cage walls) or successful (mice jumping out of the cage) attempts. The three tests, selected for their increasing anxiogenic properties, were the habituation phase of the buried food test (first test in the home cage set-up, no object at the surface of the bedding), the repetitive novel object contact task (four objects visible at the surface of the bedding) and the marble burying test (20 objects visible at the surface of the bedding). Each test was scored for 10 min.

#### Hyper-reactivity

Hyper-reactivity was recorded by looking at touch escape response, positional passivity, trunk curl and catalepsy during the handling of the mice using the following scales. Touch escape to cotton-tipped applicator stroke from above starting light and slowly getting firmer recorded over five trials: 0 = no response, 1 = mild (escape response to firm stroke), 2 = moderate (rapid response to light stroke), 3 = vigorous (escape response to approach). Positional passivity or struggle response to sequential handling: 0 = struggles when restrained by tail, 1 = struggles when restrained by neck (finger grip, not scruffed), 2 = struggles when held supine (on back), 3 = struggles when restrained by hind legs, 4 = does not struggle. Trunk curl: 0 = absent, 1 = present. Catalepsy when subject front paws are positioned on a rod elevated 3 cm from floor, the amount of time the animal stayed immobile and kept its paws on rod was recorded, with a maximum cutoff of 120 s over three trials separated by 30 s. Hyper-reactivity was also observed in other tests such as the beam walking tests or the negative geotaxis test.

### Stereotypies, repetitive behavior, perseveration

#### Repetitive novel object contact task

This novel object investigation task looks for specific unfamiliar objects preference as well as patterned sequences of sequential investigations of those items (Pearson et al., 2011; Steinbach et al., 2016). Subjects were tested in a regular clean cage (28 cm long × 18 cm wide × 12 cm high) with 1 cm of fresh bedding. The subject was first placed in the empty cage for a 20-min habituation. It was then temporary placed in an empty clean cage while four unfamiliar objects (a Lego piece, 3 cm in length; a jack, 4 cm in length; a dice, 1.5 cm in length; and a bowling pin, 3.5 cm in length) were place in the cage’s corners at ∼3 cm from the edges. The subject was then able to investigate the environment and objects during a 10-min session that was videotaped. The videos were manually scored for the occurrence of investigation of each of the four toys. Investigation was defined as clear facial or vibrissae contact with objects or burying of the objects. The number of contacts and the cumulative contact time was evaluated for each object. to determine if there was a genotype effect on the tendency to display preferences for particular toys, the frequencies of contact with each object were ranked in decreasing order from maximum to minimum preference for each subject and the frequencies were averaged by group and compared. To assess the pattern of object investigation, each specific toy was given an arbitrary number (1–4) and all possible three-digit and four-digit combinations without repeat numbers were identified. For both three- and four-object sequences the total number of choice, the number of unique sequences, and the number of choices of the three most repeated sequence was calculated for each subject as described in (Steinbach et al., 2016). To take in account the overall mouse activity, the percentage of top, top two, and top three preferred choices over the total number of choices were also calculated.

#### Barnes maze

The Barnes maze is a test of spatial memory comparable to a dry version of the Morris water maze (Barnes, 1979). In this assay, mice use spatial memory and navigation skills to orient themselves thanks to extra-maze cues placed in the test room, with the goal of locating one of 20 identical holes evenly spaced around the edge of a brightly-lit 100 cm in diameter circular arena (Maze Engineers). While most of the holes (nontarget) have nothing beneath them and lead nowhere, the target escape hole leads to shelter in a desirably darkened and enclosed goal box below the table. Two days before the beginning of the training, habituation was performed by allowing each subject to freely explore the arena (without escape box) under modest light for 5 min. At the end of the second habituation, subjects were pre-trained to learn of the presence of the escape hole by placing them for 1 min in a clear box in the middle of the arena under bright light conditions. After 1 min, the box was lifted up and the subject was gently guided near the escape hole selected randomly on the table, allowing it to enter the hole and remain inside for 1 min. For the initial training, animals were trained for 4 d to locate the escape box (in a position different from the pre-training). All trials began with the subject in a clear box in the center of the table. The trial started when the box was lifted up. If the subject located and entered the escape box within 3 min, it was left in the box for 1 min. If the subject failed to find the escape box within 3 min, it was gently guided to near the escape hole, and allowed to stay in the box for 1 min. Animals received four trials per day with an intertrial interval of 20 min for 4 d. After each trial, the maze and the escape box were cleaned using cleaning wipes to remove odors and fresh bedding was placed in the escape box. On the fifth day, animals were tested for 3 min without the escape box for a probe test. Time spent in the different quadrants was recorded. For the reversal training, the escape hole was moved to the opposite position on the maze and animals received four additional days of training followed by a reversal probe test on the fifth day. All trials were recorded by overhead camera (Noldus Ethovision) and scored for distance and latency to find escape box.

### Cognition

#### Y-maze test

Y-maze alternation is a test of working memory based on the natural tendency of mice to explore new territory whenever possible. Mice were placed in the center of a Y-maze (three 5-cm-wide and 50-cm-long arms, each set 130° from each other) and given 15 min to freely explore the three arms of the maze. The number of arm entries and the number of triads were recorded to calculate the percentage of alternation. An entry occurs when all four limbs are within the arm. A successful score is defined by three successive choices that includes one instance of each arm by the total number of opportunities for alternation. A type 1 error is determined by three consecutive choices where the first and third choices are identical. A type 2 error is defined by three consecutive choices where the second and third choices are identical. Perseverance is defined as three or more repetitive entries in the same arm.

#### Contextual and cued fear conditioning

To isolate the effects of cued and contextual fear conditioning, a 3-d assay was employed. During the training session, the mice were placed in an ethanol cleaned contextual box with a bar floor, black and white striped walls in which all movements can be recorded (Med Associate fear conditioning boxes coupled with Noldus Ethovision for control an analysis) and given 5 min to habituate. Movements were then recorded for 540 s. At 120, 260, and 400 s after the beginning of the recording, the mice were exposed to a 20-s tone (80 dB, 2 kHz) and coterminating shock (1 s, 0.7 mA). Twenty-four hours after the training phase, the animals were tested for contextual memory in the identical enclosure and movements were recorded for 240 s to assess the ability of the animal to remember the context in which the shocks had occurred the previous day. Forty-eight hours after the training phase, the animals were tested for cued memory in a different context (isopropanol cleaned, white wall insert over a mesh grid floor). They were recorded for 330 s and were presented with the identical tone from the training session at 120 s, and 260 s after the beginning of the recording session to assess the ability of each animal to remember the tone and pair it with the shock from training session. The three sessions were recorded using a camera located on the side of the boxes. Freezing, defined as lack of movement except for respiration, was scored using Noldus Ethovision software during each phase.

### Anxiety

#### Elevated zero-maze

Fear and anxiety were tested in an elevated zero-maze. The apparatus consisted of a circular black Plexiglas runway, 5 cm wide, 60 cm in diameter, and raised 60 cm off the ground (Maze Engineers). The runway was divided equally into four alternating quadrants of open arcs, enclosed only by a 1 cm inch lip, and closed arcs, with 25-cm walls. All subjects received one 5-min trial on two consecutive days starting in the center of a closed arm and were recorded by video tracking (Noldus Ethovision). Measures of cumulative open and closed arc times, latency to enter an open arc for the first time (for trials with a closed arc start), total open arm entries, latency to completely cross an open arc for the first time (for trials with a closed arc start) between two closed arcs, closed arc dipping (body in closed arc, head in open arc), open arc dipping (body in open arc, head outside of the maze) were calculated using the mean of the two trials.

#### Open field

The vertical activity in the open field was scored by counting the numbers of wall rears (while touching a side of the open field) and free-standing rears. The thigmotaxis was measured by quantifying the amount of time or distance traveled on the side of the open field compared to the center of the open field.

### Statistical analyses

*Shank3^Δ4-22^* wild-type, heterozygous, and knock-out littermates were compared for each parameter. Statistical analyses were performed with SPSS 23.0 software using different types of ANOVA with or without repeated time measures with genotype as independent variable followed by Tukey pair-wise comparisons and correction for multiple comparisons if needed or equivalent nonparametric tests when required. Newborn developmental milestones were analyzed by two-way ANCOVA using genotype and gender as between-subject factors and litter number as co-variate to take in account possible gender and litter effects. As we did not observe a gender effect, males and females were grouped together in figures and tables. to account for possible cohort effects, cohorts 3 and 4 were analyzed either together using two-way ANOVA with genotype and cohort as between-subject factors or separately using ANOVA or Kruskal–Wallis tests. Figures represent results for both cohorts analyzed together. Each cohort data and all statistical results including cohort effects are reported in tables and corresponding extended data tables. In tests comparing activity in two or more locations (open field thigmotaxis, social preference test, social transmission of food preference, novel object recognition, zero-maze) genotype × zone interactions were assessed using repeated measures. When sphericity was found violated, the Greenhouse–Geisser values were reported. The distribution of the genotypes was compared to Mendelian expectation using Pearson’s χ^2^ test, the survival curves were analyzed using survival Kaplan–Meyer χ^2^. The comparison to chance level was evaluated using either one-sample *t* test or Wilcoxon test. Normality was assessed using data visualization and Shapiro–Wilk test. All values are expressed as mean ± SEM.

## Results

### Generation of a *Shank3^Δ4-22^* mouse with a complete deletion of the *Shank3* gene

A mouse line with a complete disruption of the *Shank3* gene was generated by retargeting ES cells previously used to disrupt exons 4 through 9 (Bozdagi et al., 2010). To do this, an additional loxP site was inserted directly after exon 22 while leaving intact the two existing loxP sites flanking exons 4 and 9 (Fig. 1*A*). To generate the *Shank3^Δ4-22^*mouse line used in the present study, the floxed allele was then excised by breeding with a CMV-Cre transgenic line resulting in a deletion of exons 4-22 and therefore a constitutive disruption of all the *Shank3* murine isoforms.

Immunoblot analyses using antibodies which cross-react either with an epitope in the SH3 domain (antibody N367/62; Fig. 1*B*, left panel) or with the COOH terminal (antibody H1160, Fig. 1*B*, right panel) showed no expression of Shank3 protein in post synaptic density fractions from *Shank3^Δ4-22^*homozygous mice and reduced expression consistent with haploinsuficiency in the heterozygotes. As in humans, in mice, the *Shank3* gene has 22 exons, spans ∼58 kb of genomic DNA, and undergoes complex transcriptional regulation controlled by a combination of five intragenic promoters and extensive alternative splicing resulting in in a complex pattern of mRNA and protein isoforms (Wang et al., 2011, 2014; Kouser et al., 2013; Waga et al., 2014; Speed et al., 2015). The loss of all known major Shank3 mRNA isoforms was confirmed by RT-PCR (Fig. 1*C*).

The *Shank3^Δ4-22^*mouse line was maintained on a C57BL/6 background by heterozygote × heterozygote mating, allowing for the production of all genotypes (wild-type, heterozygous, and homozygous) as littermates. *Shank3^Δ4-22^*heterozygous and homozygous animals were viable, however abnormal Mendelian ratios were observed at the time of weaning, with a significant deficit for *Shank3^Δ4-22^*knock-out mice (Fig. 1*D*; Table 3). Adult survival curves between 1 and 22 months did not show a significant genotype difference with the current sample size, but there was evidence for higher numbers of deaths in *Shank3^Δ4-22^* homozygous mice between 18 and 22 months (Fig. 1*E*; Table 3). Although the human clinical *SHANK3* mutation is hemizygous, for completeness, we have conducted our studies in *Shank3*-null mutant mice (homozygous knock-out, KO), along with their heterozygous (Het) and wild-type (WT) littermates. The KO mice are instrumental to understand the function of Shank3, while the Het mice have significantly greater construct validity for PMS, a haploinsufficiency syndrome. To ensure the robustness of behavioral abnormalities in the adult mice, two cohorts representing all three genotypes were compared. All the cohorts used in the present study are described in Table 2.

**Table 3.** Genotype distribution at weaning and postnatal mortality

Genotype distribution at weaning								
	WT	Het	KO	%WT	%Het	%KO	χ^2^ (df2)	Asymp *p* value
All animals, observed *N*	365	686	278	27.46	51.62	20.92	12.78	**0.0017**
All animals, expected *N*	332.25	664.5	332.25	25.00	50.00	25.00		
All animals, residual *N*	32.75	21.5	-54.25	2.46	1.62	-4.08		
Males, observed *N*	185	357	147	26.85	51.81	21.34	5.10	0.0781
Males, expected *N*	172.25	344.5	172.25	25.00	50.00	25.00		
Males, residual *N*	12.75	12.5	-25.25	1.85	1.81	-3.66		
Females, observed *N*	180	329	131	28.13	51.41	20.47	8.01	**0.0182**
Females, expected *N*	160	320	160	25.00	50.00	25.00		
Females, residual *N*	20	9	-29	3.13	1.41	-4.53		

**Table 2.** Cohorts used and order of behavioral testing

Cohort 1 (10 litters) - developmental milestones								
	WT	Het	KO	Age at testing				
All animals	14	30	10	P0–P21				
Males	7	16	5	P0–P21				
Females	7	14	5	P0–P21				
								
								
Cohort 2 (10 litters) - ultrasonic vocalizations								
	WT	Het	KO	Age at testing				
All animals	16	32	9	P6				
Males	4	15	6	P6				
Females	12	17	3	P6				
								
Cohorts 3 and 4 - adult behavior								
	Cohort 3				Cohort 4			
	WT	Het	KO	Age at testing	WT	Het	KO	Age at testing
Handling, cage observation, neurological and motor reflexes	11	10	9	P86–P90	8	9	10	P103–P107
15-month weight	8	8	6	P460	5	7	4	P455
20-month weight	7	7	2	P610	4	5	3	P600
Open field	11	10	9	P93–P94	8	9	10	P106–P108
Zero-maze	11	10	9	P95–P96	8	9	10	P109–P110
Y-maze	11	10	9	P99–P101	8	9	10	P114–P122
Beam walking	11	10	9	P102–P103	8	9	10	P124–P125
Grip strength	11	10	9	P104	8	9	10	P125
Gait analysis	11	10	9	P105	8	9	10	P126
Rotarod	11	10	9	P107–P108	8	9	10	P127
Three-chambered social interaction task	11	10	9	P113–P114	8	9	10	P130–P131
Nest building	11	10	9	P120	8	9	10	P137
Novel object	11	10	9	P123–P125	8	9	10	P139–P140
Fear conditioning	11	10	9	P126–P128	8	9	10	P141–P143
Startle response *	11	10	9	P137–P139	3*	4*	4*	P155–P157
Pre-pulse inhibition	11	10	9	P137–P139	8	9	10	P155–P157
Tail flick	11	10	9	P144–P145	8	9	10	P158–P159
Olfactory habituation/dishabituation	11	10	9	P149–P157	8	9	10	P162–P165
Buried food	11	10	9	P163–P164	8	9	10	P178
Social transmission of food preference	11	10	9	P206–P215	8	9	10	P185–P192
Marble burying	11	10	8	P227–P228	8	9	10	P197
Four-object repetitivenovelobject contact task	11	10	8	P232	7	9	9	P215
Male-female social interaction	11	10	8	P240–P241	7	9	9	P217–P219
Barnes maze	11	10	7	P247–P274	7	9	8	P222–P250

For adult animals, the age indicated corresponds to the average age of the cohort. For each cohort all mice were born within two weeks of each other. *: missing animals due to technical problems during startle recording.

### Developmental milestones in *Shank3^Δ4-22^* neonates

Ten litters were used to study developmental milestones. The average litter size was 7.2 pups (ranging from five to nine), with 54 surviving passed postnatal day 2 (28 males and 26 females). As very limited gender effects were observed (for detailed analysis, see Table 4), males and females were analyzed together using both genotype and gender as fixed factors and the litter number as a covariate.

**Table 4.** Detailed results and statistical analyses related to developmental milestones

**Weight**																	
Repeated measures, sphericity violated		*F*	*p* value	Power	WT vs Het	WT vs KO	Het vs KO										
Day effect		466.906	0.000	1.000	−	−	−										
Day × genotype effect		2.275	**0.045**	0.754	−	−	−										
Day × gender effect		0.363	0.765	0.117	−	−	−										
Day × genotype × gender effect		0.569	0.742	0.214	−	−	−										
Genotype effect		3.046	**0.048**	0.560	0.144	**0.018**	0.147										
Gender effect		0.933	0.339	0.157	−	−	−										
Genotype × gender		0.686	0.509	0.158	−	−	−										
																	
												Gender effect			Gender × genotype effect		
Multifactiorial ANCOVA		WT	Het	KO	*F*	*p* value	Power	WT vs Het	WT vs KO	Het vs KO		*F*	*p* value	Power	*F*	*p* value	Power
Weight - P1	Nonnormal	1.47 ± 0.02	1.38 ± 0.02	1.36 ± 0.03	2.244	0.118	0.433	−	−	−		1.067	0.307	0.173	0.016	0.984	0.052
Weight - P2	Nonnormal	1.51 ± 0.03	1.42 ± 0.03	1.4 ± 0.04	2.010	0.146	0.393	−	−	−		0.510	0.479	0.108	0.193	0.825	0.078
Weight - P3	Nonnormal	1.62 ± 0.05	1.59 ± 0.04	1.55 ± 0.07	0.451	0.640	0.119	−	−	−		0.030	0.863	0.053	1.047	0.360	0.221
Weight - P4	Nonnormal	1.95 ± 0.09	1.87 ± 0.06	1.87 ± 0.1	0.610	0.548	0.145	−	−	−		0.822	0.369	0.144	0.378	0.688	0.107
Weight - P5	Nonnormal	2.34 ± 0.1	2.27 ± 0.07	2.27 ± 0.12	0.305	0.739	0.095	−	−	−		0.803	0.375	0.142	1.021	0.368	0.217
Weight - P6	Nonnormal	2.77 ± 0.14	2.71 ± 0.08	2.7 ± 0.15	0.320	0.728	0.098	−	−	−		0.436	0.512	0.099	0.356	0.703	0.104
Weight - P7	Nonnormal	3.29 ± 0.12	3.25 ± 0.09	3.13 ± 0.15	0.682	0.511	0.158	−	−	−		0.835	0.366	0.145	0.934	0.401	0.201
Weight - P8	Nonnormal	3.8 ± 0.14	3.73 ± 0.1	3.65 ± 0.15	0.493	0.614	0.126	−	−	−		0.723	0.400	0.132	1.023	0.368	0.217
Weight - P9	Nonnormal	4.26 ± 0.14	4.23 ± 0.1	4 ± 0.17	1.146	0.327	0.239	−	−	−		3.146	0.083	0.411	0.883	0.421	0.192
Weight - P10	Nonnormal	4.86 ± 0.11	4.72 ± 0.1	4.58 ± 0.16	1.013	0.371	0.215	−	−	−		0.299	0.587	0.083	0.051	0.951	0.057
Weight - P11	Nonnormal	5.42 ± 0.11	5.21 ± 0.1	5.03 ± 0.18	1.837	0.171	0.363	−	−	−		0.781	0.382	0.139	0.023	0.978	0.053
Weight - P12	Nonnormal	5.85 ± 0.11	5.7 ± 0.11	5.39 ± 0.13	2.148	0.129	0.417	−	−	−		0.092	0.764	0.060	0.362	0.698	0.105
Weight - P13	Nonnormal	6.22 ± 0.12	6.01 ± 0.11	5.72 ± 0.2	1.787	0.179	0.354	−	−	−		0.853	0.361	0.147	0.657	0.524	0.153
Weight - P14	Nonnormal	6.62 ± 0.12	6.42 ± 0.11	5.83 ± 0.17	4.891	**0.012**	0.777	0.274	**0.004**	**0.016**		0.577	0.451	0.115	0.618	0.544	0.147
Weight - P15	Nonnormal	7.01 ± 0.14	6.73 ± 0.12	6.38 ± 0.22	2.504	*0.093*	0.476	0.175	**0.031**	0.198		0.595	0.445	0.117	0.238	0.789	0.085
Weight - P16	Nonnormal	7.31 ± 0.14	6.96 ± 0.13	6.69 ± 0.19	2.668	*0.081*	0.502	*0.094*	**0.030**	0.318		0.157	0.694	0.067	0.072	0.931	0.060
Weight - P17	Nonnormal	7.55 ± 0.14	7.2 ± 0.13	6.83 ± 0.22	2.973	*0.061*	0.549	0.118	**0.020**	0.192		0.889	0.351	0.152	0.170	0.845	0.075
Weight - P18	Nonnormal	7.76 ± 0.14	7.43 ± 0.14	6.98 ± 0.2	3.160	**0.050**	0.577	0.152	**0.016**	0.127		0.790	0.379	0.140	0.187	0.830	0.077
Weight - P19	Nonnormal	7.98 ± 0.13	7.58 ± 0.16	7.1 ± 0.18	3.534	**0.038**	0.628	0.115	**0.011**	0.121		1.170	0.285	0.185	0.861	0.430	0.189
Weight - P20	Nonnormal	8.31 ± 0.19	7.69 ± 0.19	7.18 ± 0.2	4.268	**0.020**	0.716	*0.051*	**0.006**	0.146		0.729	0.398	0.133	1.415	0.254	0.287
Weight - P21	Nonnormal	8.67 ± 0.21	8.05 ± 0.27	7.38 ± 0.28	3.366	**0.044**	0.605	0.127	**0.013**	0.127		0.263	0.611	0.079	0.839	0.439	0.185
																	
**Eye opening**																	
Repeated measures, sphericity assumed		*F*	*p* value	Power	WT vs Het	WT vs KO	Het vs KO										
Day effect		192.080	**0.000**	**1.000**	−	−	−										
Day × genotype effect		1.565	0.190	0.469	−	−	−										
Day × gender effect		0.716	0.494	0.169	−	−	−										
Day × genotype × gender effect		0.653	0.629	0.544													
Genotype effect		1.403	0.257	0.285	−	−	−										
Gender effect		1.852	0.181	0.265	−	−	−										
Genotype × gender		0.957	0.392	0.205													
												Gender effect			Gender × genotype effect		
Multifactiorial ANCOVA		WT	Het	KO	*F*	*p* value	Power	WT vs Het	WT vs KO	Het vs KO		*F*	*p* value	Power	*F*	*p* value	Power
Eye opening score - P9	−	0 ± 0	0 ± 0	0 ± 0	−	−	−	−	−	−		−	−	−	−	−	−
Eye opening score - P10	−	0 ± 0	0 ± 0	0 ± 0	−	−	−	−	−	−		−	−	−	−	−	−
Eye opening score - P11	−	0 ± 0	0 ± 0	0 ± 0	−	−	−	−	−	−		−	−	−	−	−	−
Eye opening score - P12	Nonnormal	0.3 ± 0.2	0.28 ± 0.13	0.1 ± 0.1	0.534	0.590	0.132	−	−	−		1.917	0.173	0.273	0.496	0.613	0.126
Eye opening score - P13	Nonnormal	1.23 ± 0.34	1.35 ± 0.25	0.6 ± 0.3	1.445	0.247	0.292	−	−	−		0.707	0.405	0.130	0.032	0.969	0.055
Eye opening score - P14	Nonnormal	2.38 ± 0.18	2.75 ± 0.16	2.1 ± 0.09	4.723	**0.014**	**0.761**	0.134	0.167	**0.005**		2.464	0.124	0.336	2.248	0.118	0.433
Eye opening score - P15	Nonnormal	3 ± 0.25	3.1 ± 0.17	2.7 ± 0.26	0.646	0.529	0.151	−	−	−		0.043	0.837	0.055	1.262	0.293	0.260
Eye opening score - P16	Nonnormal	4 ± 0	3.85 ± 0.06	3.9 ± 0.1	0.734	0.486	0.166	−	−	−		3.076	0.087	0.403	1.249	0.297	0.257
Eye opening score - P17	Nonnormal	4 ± 0	3.85 ± 0.06	4 ± 0	1.665	0.201	0.332	−	−	−		1.155	0.288	0.183	1.971	0.152	0.386
Eye opening score - P18	Nonnormal	4 ± 0	3.89 ± 0.05	4 ± 0	0.957	0.392	0.205	−	−	−		0.690	0.411	0.128	1.041	0.362	0.220
Eye opening score - P19	Nonnormal	4 ± 0	3.96 ± 0.03	4 ± 0	0.428	0.654	0.115	−	−	−		0.320	0.575	0.086	0.426	0.656	0.115
Eye opening score - P20	−	4 ± 0	4 ± 0	4 ± 0	−	−	−	−	−	−		−	−	−	−	−	−
Average day of full opening	Nonnormal	15.53 ± 0.18	15.57 ± 0.33	15.9 ± 0.17	0.469	0.629	0.122	−	−	−		1.472	0.232	0.220	0.749	0.479	0.169
																	
**Ear opening**																	
Repeated measures, sphericity violated		*F*	*p* value	Power	WT vs Het	WT vs KO	Het vs KO										
Day effect		316.707	**0.000**	1.000	−	−	−										
Day × genotype effect		0.807	0.594	0.361	−	−	−										
Day × gender effect		2.150	0.079	0.617	−	−	−										
Day × genotype × gender effect		1.056	0.396	0.472	−	−	−										
Genotype effect		0.113	0.893	0.066	−	−	−										
Gender effect		0.438	0.512	0.099	−	−	−										
Genotype × gender		0.676	0.514	0.156	−	−	−										

												Gender effect			Gender × genotype effect		
Multifactiorial ANCOVA		WT	Het	KO	*F*	*p* value	Power	WT vs Het	WT vs KO	Het vs KO		*F*	*p* value	Power	*F*	*p* value	Power
Ear opening score - P1	Nonnormal	0.23 ± 0.23	0.13 ± 0.09	0 ± 0	0.753	0.477	0.170	−	−	−		2.371	0.131	0.325	0.669	0.517	0.155
Ear opening score - P2	Nonnormal	2.15 ± 0.15	2.06 ± 0.04	2 ± 0	0.675	0.514	0.156	−	−	−		2.261	0.140	0.313	0.468	0.629	0.122
Ear opening score - P3	Nonnormal	2.38 ± 0.21	2.31 ± 0.13	2.3 ± 0.21	0.123	0.885	0.068	−	−	−		1.054	0.310	0.171	0.994	0.378	0.212
Ear opening score - P4	Nonnormal	3.15 ± 0.27	3.27 ± 0.13	3.6 ± 0.22	0.966	0.389	0.207	−	−	−		2.693	0.108	0.362	1.501	0.234	0.303
Ear opening score - P5	Nonnormal	4.15 ± 0.1	4.2 ± 0.11	4.1 ± 0.1	0.167	0.847	0.074	−	−	−		0.106	0.746	0.062	0.382	0.685	0.108
Ear opening score - P6	Nonnormal	5.76 ± 0.16	5.93 ± 0.06	6 ± 0	1.052	0.358	0.222	−	−	−		0.780	0.382	0.139	0.733	0.486	0.166
Ear opening score - P7	−	6 ± 0	6 ± 0	6 ± 0	0.439	0.647	0.117	−	−	−		0.270	0.606	0.080	0.617	0.544	0.146
Ear opening score - P8	−	6 ± 0	6 ± 0	6 ± 0	−	−	−	−	−	−		−	−	−	−	−	−
Ear opening score - P9	−	6 ± 0	6 ± 0	6 ± 0	−	−	−	−	−	−		−	−	−	−	−	−
Average day of full opening	Nonnormal	6.15 ± 0.1	5.93 ± 0.06	6 ± 0	0.622	0.541	0.147	−	−	−		0.070	0.793	0.058	0.274	0.761	0.091
																	
**Tooth eruption**																	
Bottom incisor - repeated measures, sphericity violated		*F*	*p* value	Power	WT vs Het	WT vs KO	Het vs KO										
Day effect		120.634	**0.000**	1.000	−	−	−										
Day × genotype effect		1.452	0.177	0.648	−	−	−										
Day × gender effect		1.873	0.116	0.564	−	−	−										
Day × genotype × gender effect		1.671	0.107	0.723	−	−	−										
Genotype effect		1.855	0.169	0.366	−	−	−										
Gender effect		0.094	0.761	0.060	−	−	−										
Genotype × gender		0.637	0.533	0.150	−	−	−										
												Gender effect			Gender × genotype effect		
Multifactiorial ANCOVA		WT	Het	KO	*F*	*p* value	Power	WT vs Het	WT vs KO	Het vs KO		*F*	*p* value	Power	*F*	*p* value	Power
Bottom incisor score - P7	−	0 ± 0	0 ± 0	0 ± 0	−	−	−	−	−	−		−	−	−	−	−	−
Bottom incisor score - P8	Nonnormal	0.38 ± 0.14	0.06 ± 0.04	0.1 ± 0.1	3.701	**0.033**	0.650	**0.010**	0.072	0.746		0.685	0.412	0.128	1.274	0.290	0.262
Bottom incisor score - P9	Nonnormal	0.92 ± 0.07	0.75 ± 0.08	1 ± 0.14	1.247	0.297	0.257	−	−	−		1.198	0.280	0.188	1.949	0.155	0.382
Bottom incisor score - P10	Nonnormal	1.15 ± 0.1	1.03 ± 0.06	1.1 ± 0.1	0.661	0.521	0.164	−	−	−		2.184	0.147	0.304	1.371	0.265	0.279
Bottom incisor score - P11	Nonnormal	1.84 ± 0.1	1.65 ± 0.08	1.5 ± 0.16	1.438	0.248	0.291	−	−	−		0.212	0.647	0.074	0.873	0.425	0.191
Bottom incisor score - P12	Nonnormal	1.92 ± 0.07	1.93 ± 0.04	1.8 ± 0.13	0.795	0.458	0.177	−	−	−		4.249	**0.045**	0.523	1.594	0.215	0.319
Bottom incisor score - P13	−	2 ± 0	2 ± 0	2 ± 0	−	−	−	−	−	−		−	−	−	−	−	−
Bottom incisor, day of full eruption	Nonnormal	11.07 ± 0.21	11.37 ± 0.15	11.5 ± 0.37	0.720	0.492	0.164	−	−	−		0.018	0.895	0.052	0.141	0.869	0.070
																	
Top incisor - repeated measures, sphericity violated		*F*	*p* value	Power	WT vs Het	WT vs KO	Het vs KO										
Day effect		41.000	**0.000**	**1.000**	−	−	−										
Day × genotype effect		84.000	0.587	0.355	−	−	−										
Day × gender effect		41.000	0.150	0.497	−	−	−										
Day × genotype × gender effect		84.000	0.563	0.370	−	−	−										
Genotype effect		0.314	0.732	0.097	−	−	−										
Gender effect		0.845	0.363	0.147	−	−	−										
Genotype × gender		1.028	0.366	0.218	−	−	−										
												Gender effect			Gender × genotype effect		
Multifactiorial ANCOVA		WT	Het	KO	*F*	*p* value	Power	WT vs Het	WT vs KO	Het vs KO		*F*	*p* value	Power	*F*	*p* value	Power
Top incisor score - P10	−	0 ± 0	0 ± 0	0 ± 0	−	−	−	−	−	−		−	−	−	−	−	−
Top incisor score - P11	Nonnormal	0.23 ± 0.12	0.1 ± 0.05	0 ± 0	1.757	0.184	0.348	−	−	−		0.021	0.886	0.052	0.903	0.413	0.196
Top incisor score - P12	Nonnormal	0.76 ± 0.12	0.79 ± 0.07	0.8 ± 0.13	0.010	0.990	0.051	−	−	−		1.285	0.263	0.198	1.558	0.222	0.313
Top incisor score - P13	Nonnormal	1.3 ± 0.17	1.24 ± 0.13	1 ± 0.21	0.590	0.559	0.142	−	−	−		0.009	0.925	0.051	0.804	0.454	0.179
Top incisor score - P14	Nonnormal	1.76 ± 0.12	1.79 ± 0.07	1.8 ± 0.13	0.010	0.990	0.051	−	−	−		1.285	0.263	0.198	1.558	0.222	0.313
Top incisor score - P15	Nonnormal	1.92 ± 0.07	1.89 ± 0.05	1.8 ± 0.13	0.487	0.618	0.125	−	−	−		4.489	**0.040**	0.545	0.861	0.430	0.189
Top incisor score - P16	−	2 ± 0	2 ± 0	2 ± 0	−	−	−	−	−	−		−	−	−	−	−	−
Top incisor, day of full eruption	Nonnormal	13.92 ± 0.26	14.41 ± 0.17	14.2 ± 0.32	1.110	0.339	0.233	−	−	−		0.816	0.371	0.143	1.006	0.374	0.214
																	
**Fur development**																	
Repeated measures, sphericity violated		*F*	*p* value	Power	WT vs Het	WT vs KO	Het vs KO										
Day effect		347.979	**0.000**	1.000	−	−	−										
Day × genotype effect		0.885	0.546	0.458	−	−	−										
Day × gender effect		1.948	0.089	0.646	−	−	−										
Day × genotype × gender effect		3.234	**0.001**	0.986	−	−	−										
Genotype effect		1.683	0.198	0.335	−	−	−										
Gender effect		0.635	0.430	0.122	−	−	−										
Genotype × gender		8.265	**0.001**	0.950	−	−	−										
												Gender effect			Gender × genotype effect		
Multifactiorial ANCOVA		WT	Het	KO	*F*	*p* value	Power	WT vs Het	WT vs KO	Het vs KO		*F*	*p* value	Power	*F*	*p* value	Power
Fur score - P1	Nonnormal	1 ± 0	0.92 ± 0.07	1 ± 0	0.439	0.647	0.117	−	−	−		0.270	0.606	0.080	0.617	0.544	0.146
Fur score - P2	Nonnormal	1.76 ± 0.12	1.79 ± 0.07	2 ± 0	1.484	0.238	0.300	−	−	−		0.000	0.995	0.050	6.724	**0.003**	0.897
Fur score - P3	Nonnormal	2.53 ± 0.18	2.37 ± 0.1	2.5 ± 0.16	0.898	0.415	0.195	−	−	−		1.736	0.194	0.252	7.221	**0.002**	0.918
Fur score - P4	Nonnormal	3.23 ± 0.2	3.13 ± 0.11	3.4 ± 0.16	1.605	0.212	0.321	−	−	−		5.973	**0.019**	0.667	9.904	**0.000**	0.978

Fur score - P5	Nonnormal	3.92 ± 0.13	3.86 ± 0.06	4 ± 0	1.034	0.364	0.219	−	−	−		0.013	0.909	0.051	4.885	**0.012**	0.777
Fur score - P6	Nonnormal	3.92 ± 0.13	3.89 ± 0.05	4 ± 0	0.657	0.523	0.153	−	−	−		0.090	0.766	0.060	4.026	**0.025**	0.689
Fur score - P7	Nonnormal	4.15 ± 0.19	4.27 ± 0.1	4.5 ± 0.16	1.002	0.375	0.213	−	−	−		0.353	0.556	0.089	1.378	0.263	0.281
Fur score - P8	Nonnormal	4.69 ± 0.17	4.62 ± 0.09	5 ± 0	2.746	0.075	0.514	−	−	−		1.116	0.297	0.178	4.075	**0.024**	0.694
Fur score - P9	Nonnormal	5 ± 0	4.93 ± 0.04	5 ± 0	0.927	0.403	0.200	−	−	−		0.604	0.441	0.118	1.203	0.310	0.249
Fur score - P10	Nonnormal	5.23 ± 0.12	5.24 ± 0.08	5.3 ± 0.15	0.125	0.882	0.068	−	−	−		0.007	0.936	0.051	2.343	0.108	0.450
Fur score - P11	Nonnormal	5.53 ± 0.14	5.72 ± 0.08	5.8 ± 0.13	0.906	0.411	0.196	−	−	−		0.997	0.324	0.164	3.754	**0.031**	0.656
Fur score - P12	Nonnormal	6 ± 0	5.96 ± 0.03	6 ± 0	0.401	0.672	0.111	−	−	−		0.274	0.603	0.081	0.479	0.622	0.123
Fur score - P13	−	6 ± 0	6 ± 0	6 ± 0	−	−	.	−	−	−		−	−	−	−	−	−
Fur score - P14	−	6 ± 0	6 ± 0	6 ± 0	−	−	.	−	−	−		−	−	−	−	−	−
Day of full fur	Nonnormal	11.3 ± 0.2	10.75 ± 0.37	10.9 ± 0.23	0.460	0.634	0.120	−	−	−		0.110	0.741	0.062	1.960	0.153	0.384
																	
**Auditory startle**																	
Repeated measures, sphericity violated		*F*	*p* value	Power	WT vs Het	WT vs KO	Het vs KO										
Day effect		56.506	**0.000**	****	−	−	−										
Day × genotype effect		3.280	**0.002**	****	−	−	−										
Day × gender effect		0.283	0.873		−	−	−										
Day × genotype × gender effect		1.321	0.241		−	−	−										
Genotype effect		12.867	**0.000**	****	0.070	**0.000**	**0.000**										
Gender effect		0.058	0.811		−	−	−										
Genotype × gender		0.358	0.701		−	−	−										
												Gender effect			Gender × genotypeeffect		
Multifactiorial ANCOVA		WT	Het	KO	*F*	*p* value	Power	WT vs Het	WT vs KO	Het vs KO		*F*	*p* value	Power	*F*	*p* value	Power
Percentage of responders - P10	−	0 ± 0	0 ± 0	0 ± 0	−	−	−	−	−	−		−	−	−	−	−	−
Percentage of responders - P11	Nonnormal	15.38 ± 10.41	13.79 ± 6.51	0 ± 0	1.308	0.281	0.268	−	−	−		0.001	0.971	0.050	3.054	0.057	0.561
Percentage of responders - P12	Nonnormal	53.84 ± 14.39	13.79 ± 6.51	0 ± 0	8.700	**0.001**	0.959	**0.001**	**0.000**	0.178		0.488	0.488	0.105	1.584	0.217	0.318
Percentage of responders - P13	Nonnormal	53.84 ± 14.39	55.17 ± 9.39	10 ± 10	3.045	0.058	0.560	0.969	**0.043**	**0.023**		0.238	0.628	0.077	1.082	0.348	0.228
Percentage of responders - P14	Nonnormal	100 ± 0	86.2 ± 6.51	60 ± 16.32	3.161	0.052	0.577	0.265	**0.016**	0.072		0.000	0.990	0.050	0.009	0.991	0.051
Percentage of responders - P15	Nonnormal	100 ± 0	100 ± 0	70 ± 15.27	8.228	**0.001**	0.949	0.970	**0.001**	**0.000**		1.019	0.318	0.167	0.865	0.428	0.189
Percentage of responders - P16	−	100 ± 0	100 ± 0	100 ± 0	−	−	−	−	−	−		−	−	−	−	−	−
Percentage of responders - average	Nonnormal	51.92 ± 1.67	47.41 ± 1.31	36.66 ± 2.83	7.944	**0.001**	0.995	0.286	**0.000**	**0.002**		0.466	0.498	0.056	0.144	0.866	0.104
First day of two consecutive successes	Nonnormal	14.07 ± 0.26	14.41 ± 0.16	15.6 ± 0.33	12.867	**0.000**	0.941	0.070	**0.000**	**0.000**		0.058	0.811	0.102	0.358	0.701	0.071
																	
**Cliff aversion**																	
Repeated measures, sphericity violated		*F*	*p* value	Power	WT vs Het	WT vs KO	Het vs KO										
Day effect		3.957	**0.000**	**0.995**	−	−	−										
Day × genotype effect		0.796	0.702	0.580	−	−	−										
Day × gender effect		0.613	0.782	0.299	−	−	−										
Day × genotype × gender effect		1.266	0.209	0.835	−	−	−										
Genotype effect		1.355	0.269	0.276	−	−	−										
Gender effect		0.218	0.643	0.074	−	−	−										
Genotype × gender		0.116	0.891	0.067	−	−	−										
												Gender effect			Gender × genotype effect		
Multifactiorial ANCOVA		WT	Het	KO	*F*	*p* value	Power	WT vs Het	WT vs KO	Het vs KO		*F*	*p* value	Power	*F*	*p* value	Power
Time to turn (seconds) - P2	Nonnormal	23.61 ± 2.77	24.44 ± 1.86	21.77 ± 3.56	0.745	0.481	0.168	−	−	−		0.482	0.491	0.104	0.866	0.428	0.189
Time to turn (seconds) - P3	Nonnormal	15.76 ± 3.37	14.17 ± 2.06	14.7 ± 4.34	0.099	0.906	0.064	−	−	−		0.056	0.814	0.056	1.513	0.232	0.304
Time to turn (seconds) - P4	Nonnormal	8.61 ± 3	4.93 ± 1.35	7.6 ± 3.73	0.963	0.390	0.206	−	−	−		0.340	0.563	0.088	1.916	0.160	0.376
Time to turn (seconds) - P5	Nonnormal	6.84 ± 2.86	7.03 ± 1.69	8.4 ± 3.61	0.156	0.856	0.073	−	−	−		0.898	0.349	0.153	2.242	0.119	0.432
Time to turn (seconds) - P6	Nonnormal	11.15 ± 3.42	8.75 ± 2.07	9.6 ± 3.55	0.126	0.882	0.068	−	−	−		0.040	0.843	0.054	2.223	0.121	0.429
Time to turn (seconds) - P7	Nonnormal	14.38 ± 3.83	9.75 ± 2.21	10 ± 4.36	1.057	0.356	0.223	−	−	−		1.259	0.268	0.195	0.368	0.694	0.105
Time to turn (seconds) - P8	Nonnormal	12.61 ± 3.97	4.82 ± 1.34	6.55 ± 3.08	2.580	0.087	0.488		0.144	0.788		0.618	0.436	0.120	0.315	0.731	0.097
Time to turn (seconds) - P9	Nonnormal	10.69 ± 3.19	9.72 ± 2.18	3.8 ± 0.92	1.129	0.333	0.236	−	−	−		0.011	0.917	0.051	0.285	0.753	0.092
Time to turn (seconds) - P10	Nonnormal	13.46 ± 3.45	7.03 ± 1.62	5.6 ± 2.77	2.447	0.098	0.466	**0.048**	0.076	0.770		1.109	0.298	0.177	0.316	0.731	0.097
Time to turn (seconds) - P11	Nonnormal	9.3 ± 2.66	11.51 ± 2.38	9 ± 3.55	0.634	0.535	0.149	−	−	−		0.263	0.611	0.079	1.486	0.238	0.300
Time to turn (seconds) - P12	Nonnormal	8.61 ± 1.52	8.93 ± 1.67	5.3 ± 1.21	0.467	0.630	0.121	−	−	−		0.001	0.975	0.050	0.651	0.527	0.152
Time to turn (seconds) - P13	Nonnormal	5.46 ± 1.7	6.48 ± 1.42	5.3 ± 2.78	0.125	0.883	0.068	−	−	−		2.134	0.151	0.298	0.791	0.460	0.176
Time to turn (seconds) - P14	Nonnormal	5.76 ± 2.16	4.1 ± 0.67	4.3 ± 1.67	0.605	0.551	0.144	−	−	−		1.345	0.253	0.205	0.941	0.398	0.202
Number of falls	Nonnormal	1.07 ± 0.53	0.44 ± 0.11	0.6 ± 0.26	1.568	0.220	0.314	−	−	−		3.688	0.061	0.467	1.125	0.334	0.235
First day of two consecutive successes (10-s cutoff)	Nonnormal	4.84 ± 0.29	4.75 ± 0.22	5.2 ± 0.55	0.546	0.583	0.134	−	−	−		2.726	0.106	0.365	2.174	0.126	0.421
First day of two consecutive successes (30-s cutoff)	Nonnormal	4.38 ± 0.33	4.06 ± 0.14	4.5 ± 0.45	1.370	0.265	0.279	−	−	−		0.037	0.849	0.054	1.044	0.361	0.221
Time to turn (seconds) - mean	Nonnormal	11.25 ± 1.25	9.36 ± 0.65	8.5 ± 0.85	1.315	0.279	0.269	−	−	−		0.259	0.613	0.079	0.119	0.888	0.067
																	

Day effect		5.197	**0.000**	0.994	−	−	−										
Day × genotype effect		0.866	0.581	0.502	−	−	−										
Day × gender effect		0.830	0.547	0.325	−	−	−										
Day × genotype × gender effect		1.115	0.348	0.637	−	−	−										
Genotype effect		2.147	0.129	0.416	−	−	−										
Gender effect		0.152	0.698	0.067	−	−	−										
Genotype × gender		0.834	0.441	0.184	−	−	−										
												Gender effect			Gender × genotype effect		
Multifactiorial ANCOVA		WT	Het	KO	*F*	*p* value	Power	WT vs Het	WT vs KO	Het vs KO		*F*	*p* value	Power	*F*	*p* value	Power
Percentage of responders - P7	Nonnormal	46.15 ± 14.39	17.24 ± 7.13	50 ± 16.66	2.610	0.085	0.493	0.076	0.870	0.073		0.347	0.559	0.089	0.888	0.419	0.193
Percentage of responders - P8	Nonnormal	30.76 ± 13.32	6.89 ± 4.78	30 ± 15.27	2.340	0.108	0.449	−	−	−		0.089	0.766	0.060	0.834	0.441	0.184
Percentage of responders - P9	Nonnormal	46.15 ± 14.39	17.24 ± 7.13	10 ± 10	2.860	0.068	0.532	**0.046**	**0.037**	0.551		0.244	0.624	0.077	0.368	0.694	0.105
Percentage of responders - P10	Nonnormal	38.46 ± 14.04	24.13 ± 8.08	30 ± 15.27	0.394	0.677	0.110	−	−	−		3.054	0.088	0.401	1.829	0.173	0.361
Percentage of responders - P11	Nonnormal	53.84 ± 14.39	62.06 ± 9.16	70 ± 15.27	0.461	0.633	0.121	−	−	−		0.143	0.707	0.066	2.804	0.071	0.524
Percentage of responders - P12	Nonnormal	46.15 ± 14.39	41.37 ± 9.3	50 ± 16.66	0.214	0.808	0.081	−	−	−		0.527	0.472	0.109	0.270	0.765	0.090
Percentage of responders - P13	Nonnormal	46.15 ± 14.39	48.27 ± 9.44	60 ± 16.32	0.064	0.938	0.059	−	−	−		1.507	0.226	0.225	0.890	0.418	0.194
Percentage of responders - P14	Nonnormal	61.53 ± 14.04	72.41 ± 8.44	80 ± 13.33	0.379	0.687	0.107	−	−	−		0.022	0.882	0.052	0.468	0.629	0.122
Percentage of responders - P15	Nonnormal	100 ± 0	96.55 ± 3.44	90 ± 10	0.497	0.612	0.126	−	−	−		0.569	0.455	0.114	2.150	0.129	0.417
Percentage of responders - Average	Nonnormal	52.13 ± 5.39	42.91 ± 2.62	52.22 ± 3.33	2.147	0.129	0.416	−	−	−		0.152	0.698	0.067	0.834	0.441	0.184
First day of two consecutive successes	Nonnormal	9.23 ± 0.63	10.13 ± 0.36	8.8 ± 0.61	1.851	0.169	0.365	−	−	−		0.137	0.713	0.065	1.106	0.340	0.232
																	
**Rooting reflex**																	
Repeated measures, sphericity violated		*F*	*p* value	Power	WT vs Het	WT vs KO	Het vs KO										
Day effect		8.013	**0.000**	0.999	−	−	−										
Day × genotype effect		1.657	0.107	0.735	−	−	−										
Day × gender effect		0.847	0.503	0.276	−	−	−										
Day × genotype × gender effect		1.347	0.219	0.625	−	−	−										
Genotype effect		1.689	0.196	0.336	−	−	−										
Gender effect		4.277	**0.045**	0.525	−	−	−										
Genotype × gender		0.283	0.755	0.092	−	−	−										
												Gender effect			Gender × genotype effect		
Multifactiorial ANCOVA		WT	Het	KO	*F*	*p* value	Power	WT vs Het	WT vs KO	Het vs KO		*F*	*p* value	Power	*F*	*p* value	Power
Percentage of responders - P2	Nonnormal	23.07 ± 12.16	6.89 ± 4.78	20 ± 13.33	1.259	0.294	0.259	−	−	−		2.018	0.163	0.285	2.605	0.085	0.492
Percentage of responders - P3	Nonnormal	38.46 ± 14.04	34.48 ± 8.98	20 ± 13.33	0.643	0.531	0.151	−	−	−		1.878	0.177	0.268	0.771	0.469	0.173
Percentage of responders - P4	Nonnormal	46.15 ± 14.39	58.62 ± 9.3	50 ± 16.66	0.292	0.748	0.093	−	−	−		0.701	0.407	0.130	2.220	0.121	0.429
Percentage of responders - P5	Nonnormal	61.53 ± 14.04	82.75 ± 7.13	60 ± 16.32	1.489	0.237	0.301	−	−	−		3.072	0.087	0.403	0.308	0.736	0.096
Percentage of responders - P6	Nonnormal	92.3 ± 7.69	68.96 ± 8.74	70 ± 15.27	1.499	0.234	0.302	−	−	−		3.120	0.084	0.408	0.395	0.676	0.110
Percentage of responders - P7	Nonnormal	84.61 ± 10.41	68.96 ± 8.74	90 ± 10	1.161	0.323	0.242	−	−	−		0.959	0.333	0.160	1.595	0.214	0.320
Percentage of responders - P8	Nonnormal	84.61 ± 10.41	58.62 ± 9.3	40 ± 16.32	2.196	0.123	0.425	−	−	−		0.618	0.436	0.120	0.193	0.826	0.078
Percentage of responders - P9	Nonnormal	76.92 ± 12.16	31.03 ± 8.74	40 ± 16.32	4.400	**0.018**	0.730	**0.005**	**0.055**	0.687		2.183	0.147	0.304	0.616	0.545	0.146
Percentage of responders - P10	Nonnormal	38.46 ± 14.04	13.79 ± 6.51	20 ± 13.33	1.743	0.187	0.346	−	−	−		0.010	0.919	0.051	1.945	0.155	0.381
Percentage of responders - P11	Nonnormal	0 ± 0	0 ± 0	10 ± 10	2.310	0.111	0.444	−	−	−		2.619	0.113	0.353	2.258	0.117	0.435
Percentage of responders - P12	−	0 ± 0	0 ± 0	0 ± 0	−	−	−	−	−	−		−	−	−	−	−	−
Day of first observation	Nonnormal	4.15 ± 0.45	4.31 ± 0.28	5 ± 0.66	0.843	0.437	0.185	−	−	−		1.546	0.220	0.229	0.757	0.475	0.170
Day of last observation	Nonnormal	9.61 ± 0.56	8.58 ± 0.3	9.1 ± 0.48	1.599	0.214	0.320	−	−	−		1.428	0.239	0.215	1.200	0.311	0.249
																	
**Grasping reflex**																	
Repeated measures, sphericity assumed		*F*	*p* value	Power	WT vs Het	WT vs KO	Het vs KO										
Day effect		28.265	0.000	1.000	−	−	−										
Day × genotype effect		1.038	0.415	0.591	−	−	−										
Day × gender effect		0.534	0.850	0.208	−	−	−										
Day × genotype × gender effect		1.356	0.150	0.725	−	−	−										
Genotype effect		3.923	**0.027**	0.677	0.304	0.116	**0.008**										
Gender effect		0.052	0.821	0.056	−	−	−										
Genotype × gender		0.320	0.728	0.098	−	−	−										
												Gender effect			Gender × genotype effect		
Multifactiorial ANCOVA		WT	Het	KO	*F*	*p* value	Power	WT vs Het	WT vs KO	Het vs KO		*F*	*p* value	Power	*F*	*p* value	Power
Grasping score - P5	Nonnormal	1.3 ± 0.23	1.34 ± 0.16	1.6 ± 0.16	0.197	0.822	0.079	−	−	−		0.877	0.354	0.150	2.268	0.115	0.437
Grasping score - P6	Nonnormal	2.46 ± 0.36	2.93 ± 0.21	2.8 ± 0.38	0.580	0.564	0.140	−	−	−		0.184	0.670	0.070	0.595	0.556	0.143
Grasping score - P7	Nonnormal	2.92 ± 0.28	3.34 ± 0.17	2.9 ± 0.23	2.260	0.116	0.436	−	−	−		0.858	0.359	0.148	1.670	0.200	0.333
Grasping score - P8	Nonnormal	3.38 ± 0.28	3.41 ± 0.15	3.2 ± 0.24	0.316	0.731	0.097	−	−	−		1.283	0.264	0.198	1.183	0.316	0.246
Grasping score - P9	Nonnormal	3.76 ± 0.3	3.79 ± 0.09	3.8 ± 0.41	0.035	0.966	0.055	−	−	−		0.336	0.565	0.088	1.031	0.365	0.218
Grasping score - P10	Nonnormal	4.38 ± 0.24	4.65 ± 0.19	3.9 ± 0.37	2.102	0.134	0.409	−	−	−		0.035	0.852	0.054	1.782	0.180	0.353
Grasping score - P11	Nonnormal	5.3 ± 0.23	5.41 ± 0.15	4.9 ± 0.31	1.591	0.215	0.319	−	−	−		0.016	0.899	0.052	1.314	0.279	0.269
Grasping score - P12	Nonnormal	5.3 ± 0.23	5.37 ± 0.15	5 ± 0.29	0.477	0.624	0.123	−	−	−		0.028	0.868	0.053	0.393	0.678	0.109
Grasping score - P13	Nonnormal	5.69 ± 0.17	5.86 ± 0.09	5.2 ± 0.24	3.789	**0.030**	0.660	0.399	0.092	**0.009**		1.328	0.255	0.204	0.287	0.752	0.093
Grasping score - P14	Nonnormal	5.69 ± 0.13	5.68 ± 0.12	4.6 ± 0.26	10.311	**0.000**	0.982	0.945	**0.000**	**0.000**		0.034	0.855	0.054	0.334	0.718	0.100
Grasping score - Average	Nonnormal	4.02 ± 0.13	4.18 ± 0.07	3.79 ± 0.13	3.923	**0.027**	0.677	0.304	0.116	**0.008**		0.052	0.821	0.056	0.320	0.728	0.098
First day of two consecutive successes (score 4)	Nonnormal	9.38 ± 0.43	8.75 ± 0.29	10.9 ± 0.37	10.068	**0.000**	0.979	0.233	**0.005**	**0.000**		0.131	0.719	0.065	1.318	0.278	0.270

Day effect		21.337	**0.000**	**1.000**	−	−	−										
Day × genotype effect		0.988	0.460	0.563	−	−	−										
Day × gender effect		0.921	0.478	0.356	−	−	−										
Day × genotype × gender effect		0.688	0.758	0.390	−	−	−										
Genotype effect		1.593	0.215	0.319	−	−	−										
Gender effect		0.857	0.360	0.148	−	−	−										
Genotype × gender		0.865	0.428	0.189	−	−	−										
												Gender effect			Gender × genotype effect		
Multifactiorial ANCOVA		WT	Het	KO	*F*	*p* value	Power	WT vs Het	WT vs KO	Het vs KO		*F*	*p* value	Power	*F*	*p* value	Power
Time to turn (seconds) - P2	Nonnormal	18.15 ± 2.97	19.93 ± 2.09	24.2 ± 2.64	0.933	0.401	0.201	−	−	−		0.382	0.540	0.093	1.161	0.323	0.242
Time to turn (seconds) - P3	Nonnormal	20.76 ± 3.19	18.13 ± 1.98	19.7 ± 3.05	0.398	0.674	0.110	−	−	−		0.020	0.887	0.052	1.146	0.327	0.239
Time to turn (seconds) - P4	Nonnormal	23.61 ± 2.26	18.03 ± 2.12	22.8 ± 3.71	1.506	0.233	0.304	−	−	−		0.078	0.781	0.059	1.392	0.259	0.283
Time to turn (seconds) - P5	Nonnormal	18.61 ± 3.27	16.82 ± 2.19	25.5 ± 2.29	2.431	0.100	0.464	−	−	−		0.284	0.597	0.082	0.129	0.879	0.069
Time to turn (seconds) - P6	Nonnormal	18.69 ± 3.26	14.2 ± 2.28	18.8 ± 3.14	1.242	0.299	0.256	−	−	−		0.163	0.688	0.068	0.079	0.925	0.061
Time to turn (seconds) - P7	Nonnormal	10.38 ± 3.18	8.37 ± 1.72	9 ± 3.16	0.155	0.857	0.072	−	−	−		4.992	**0.031**	0.589	0.741	0.482	0.168
Time to turn (seconds) - P8	Nonnormal	5 ± 1.91	5.34 ± 1.27	4.5 ± 1.43	0.073	0.930	0.060	−	−	−		3.064	0.087	0.402	0.023	0.978	0.053
Time to turn (seconds) - P9	Nonnormal	3 ± 0.62	4.24 ± 1.09	3.3 ± 0.83	0.397	0.675	0.110	−	−	−		0.371	0.546	0.092	0.413	0.664	0.113
Time to turn (seconds) - P10	Nonnormal	1.3 ± 0.13	2.06 ± 0.43	1.5 ± 0.16	0.901	0.414	0.196	−	−	−		0.002	0.966	0.050	0.059	0.943	0.058
Time to turn (seconds) - P11	Nonnormal	1 ± 0	1.27 ± 0.15	1.3 ± 0.15	1.320	0.277	0.270	−	−	−		0.034	0.854	0.054	0.314	0.732	0.097
Time to turn (seconds) - P12	Nonnormal	1 ± 0	1.03 ± 0.03	1 ± 0	0.398	0.674	0.110	−	−	−		0.331	0.568	0.087	0.196	0.823	0.079
Time to turn (seconds) - P13	−	1 ± 0	1 ± 0	1 ± 0	−	−	−	−	−	−		−	−	−	−	−	−
Time to turn (seconds) - Mean	Normal	10.21 ± 1.03	9.2 ± 0.59	11.05 ± 0.79	1.593	0.215	0.319	−	−	−		0.857	0.360	0.148	0.865	0.428	0.189
Time to turn (days) - first day of two consecutive successes	Nonnormal	9.61 ± 0.26	9.44 ± 0.34	9.7 ± 0.36	0.139	0.871	0.070	−	−	−		3.774	0.058	0.476	1.174	0.319	0.244
																	
**Negative geotaxis**																	
Repeated measures, sphericity violated		*F*	*p* value	Power	WT vs Het	WT vs KO	Het vs KO										
Day effect		12.128	**0.000**	1.000	−	−	−										
Day × genotype effect		1.526	0.086	0.895	−	−	−										
Day × gender effect		1.036	0.409	0.488	−	−	−										
Day × genotype × gender effect		1.386	0.144	0.855	−	−	−										
Genotype effect		2.110	0.133	0.410	−	−	−										
Gender effect		0.493	0.486	0.106	−	−	−										
Genotype × gender		0.090	0.914	0.063	−	−	−										
												Gender effect			Gender × genotype effect		
Multifactiorial ANCOVA		WT	Het	KO	*F*	*p* value	Power	WT vs Het	WT vs KO	Het vs KO		*F*	*p* value	Power	*F*	*p* value	Power
Time to turn (seconds) - P2	Nonnormal	-9.66 ± 5.21	-18.82 ± 2.48	-11.6 ± 4.73	1.821	0.174	0.360	−	−	−		3.882	0.055	0.487	1.077	0.350	0.227
Time to turn (seconds) - P3	Nonnormal	-8.69 ± 5.99	-15.58 ± 2.88	-11.8 ± 5.61	0.847	0.436	0.186	−	−	−		0.443	0.509	0.100	0.364	0.697	0.105
Time to turn (seconds) - P4	Nonnormal	-6 ± 5.23	-10.82 ± 1.86	-15.2 ± 3.51	1.537	0.226	0.309	−	−	−		0.656	0.422	0.124	4.540	**0.016**	0.744
Time to turn (seconds) - P5	Nonnormal	9.69 ± 3.67	-4.2 ± 3.15	-2.2 ± 5.3	4.418	**0.018**	0.732	**0.006**	**0.033**	0.936		0.399	0.531	0.095	0.766	0.471	0.172
Time to turn (seconds) - P6	Nonnormal	6.84 ± 4.38	3.2 ± 2.97	8.5 ± 3.87	0.517	0.600	0.130	−	−	−		0.010	0.920	0.051	0.905	0.412	0.196
Time to turn (seconds) - P7	Nonnormal	6.3 ± 4.2	6.31 ± 3.13	12.4 ± 5.82	0.281	0.756	0.092	−	−	−		0.699	0.408	0.129	0.013	0.987	0.052
Time to turn (seconds) - P8	Nonnormal	8.3 ± 4.42	11.44 ± 2.71	6.8 ± 6.1	0.331	0.720	0.100	−	−	−		0.124	0.726	0.064	0.862	0.429	0.189
Time to turn (seconds) - P9	Nonnormal	12.76 ± 3.75	12.17 ± 2.44	23.1 ± 1.6	3.787	**0.030**	0.660	0.828	**0.035**	**0.010**		0.023	0.880	0.053	1.215	0.307	0.251
Time to turn (seconds) - P10	Nonnormal	9.84 ± 2.94	10.03 ± 2.29	19 ± 3.22	2.707	0.078	0.508	0.959	0.055	**0.032**		2.235	0.142	0.310	0.497	0.612	0.126
Time to turn (seconds) - P11	Nonnormal	10.92 ± 3.44	13.62 ± 2.17	15 ± 3.88	0.580	0.564	0.140	−	−	−		2.444	0.125	0.334	4.258	**0.020**	0.715
Time to turn (seconds) - P12	Nonnormal	11.92 ± 2.92	15.96 ± 1.74	22.2 ± 1.33	3.269	**0.047**	0.592	0.379	**0.014**	0.073		0.205	0.653	0.073	2.337	0.109	0.448
Time to turn (seconds) - P13	Nonnormal	15.23 ± 3.04	18.58 ± 1.5	24.1 ± 0.62	3.112	0.054	0.570	0.219	**0.017**	0.091		1.431	0.238	0.216	1.112	0.338	0.233
Time to turn (seconds) - P14	Nonnormal	22.69 ± 1.43	21.82 ± 1.17	22.3 ± 2.25	0.135	0.874	0.070	−	−	−		2.267	0.139	0.313	1.471	0.241	0.297
Time to turn (seconds) - Mean	Normal	6.99 ± 1.25	4.9 ± 1.06	8.66 ± 1.1	2.110	0.133	0.410	−	−	−		0.493	0.486	0.106	0.090	0.914	0.063
Falls	Nonnormal	9 ± 0.83	10.31 ± 0.48	9.1 ± 0.45	1.527	0.228	0.307	−	−	−		0.192	0.664	0.071	0.307	0.737	0.096
																	
**Air righting**																	
Repeated measures, sphericity violated		*F*	*p* value	Power	WT vs Het	WT vs KO	Het vs KO										
Day effect		21.651	**0.000**	1.000	−	−	−										
Day × genotype effect		3.211	**0.001**	0.986	−	−	−										
Day × gender effect		2.423	0.037	0.760	−	−	−										
Day × genotype × gender effect		1.309	0.227	0.664	−	−	−										
Genotype effect		3.166	0.052	0.577	0.693	0.070	**0.016**										
Gender effect		0.464	0.499	0.102	−	−	−										
Genotype × gender		1.482	0.238	0.299	−	−	−										
												Gender effect			Gender × genotype effect		
Multifactiorial ANCOVA		WT	Het	KO	*F*	*p* value	Power	WT vs Het	WT vs KO	Het vs KO		*F*	*p* value	Power	*F*	*p* value	Power
Air righting score - P8	Nonnormal	0.61 ± 0.26	0.41 ± 0.13	0.8 ± 0.29	1.160	0.323	0.242	−	−	−		5.791	**0.020**	0.653	5.562	**0.007**	0.831
Air righting score - P9	Nonnormal	0.38 ± 0.21	0.93 ± 0.17	0.4 ± 0.22	2.272	0.115	0.438	−	−	−		0.004	0.948	0.050	0.439	0.648	0.117
Air righting score - P10	Nonnormal	1.69 ± 0.2	1.44 ± 0.13	0.7 ± 0.3	5.755	**0.006**	0.844	0.426	**0.003**	**0.005**		1.456	0.234	0.219	0.438	0.648	0.117
Air righting score - P11	Nonnormal	1.3 ± 0.26	1.44 ± 0.16	0.4 ± 0.26	5.407	**0.008**	0.819	0.703	**0.015**	**0.002**		2.641	0.111	0.356	0.639	0.533	0.150
Air righting score - P12	Nonnormal	1.84 ± 0.15	1.89 ± 0.05	1.4 ± 0.22	4.066	**0.024**	0.693	0.742	**0.034**	**0.007**		0.332	0.567	0.087	0.647	0.528	0.152
Air righting score - P13	Nonnormal	1.76 ± 0.16	1.68 ± 0.13	1.7 ± 0.21	0.069	0.934	0.060	−	−	−		2.104	0.154	0.295	0.396	0.675	0.110

Air righting score - P14	Nonnormal	2 ± 0	2 ± 0	2 ± 0	−	−	.	−	−	−		−	−	.	−	−	.
Air righting score - P15	Nonnormal	1.92 ± 0.07	2 ± 0	2 ± 0	1.621	0.209	0.324	−	−	−		1.716	0.197	0.249	1.464	0.242	0.296
Air righting score - P16	Nonnormal	2 ± 0	2 ± 0	2 ± 0	−	−	.	−	−	−		−	−	.			.
Air righting score - P17	Nonnormal	2 ± 0	1.96 ± 0.03	2 ± 0	0.398	0.674	0.110	−	−	−		0.331	0.568	0.087	0.196	0.823	0.079
Air righting score - P18	Nonnormal	2 ± 0	2 ± 0	2 ± 0	−	−	.	−	−	−		−	−	.	−	−	.
Air righting score - P19	Nonnormal	2 ± 0	2 ± 0	2 ± 0	−	−	.	−	−	−		−	−	.	−	−	.
Air righting score - P20	Nonnormal	2 ± 0	2 ± 0	2 ± 0	−	−	.	−	−	−		−	−	.	−	−	.
Air righting score - Mean	Nonnormal	1.65 ± 0.06	1.67 ± 0.03	1.49 ± 0.06	3.166	**0.049**	0.577	0.693	0.070	**0.016**		0.464	0.499	0.102	1.482	0.238	0.299
First day of two consecutive successes	Nonnormal	11.84 ± 0.5	11.37 ± 0.29	12.9 ± 0.56	2.814	0.071	0.525	0.378	0.184	**0.023**		0.959	0.333	0.160	1.173	0.319	0.244
																	
**Wire suspension**																	
Repeated measures, sphericity violated		*F*	*p* value	Power	WT vs Het	WT vs KO	Het vs KO										
Day effect		16.511	**0.000**	1.000	−	−	−										
Day × genotype effect		3.538	**0.000**	0.994	−	−	−										
Day × gender effect		0.497	0.782	0.186	−	−	−										
Day × genotype × gender effect		0.635	0.787	0.333	−	−	−										
Genotype effect		13.553	**0.000**	0.997	**0.013**	**0.000**	**0.001**										
Gender effect		0.303	0.585	0.084	−	−	−										
Genotype × gender		2.871	0.067	0.534	−	−	−										
												Gender effect			Gender × genotype effect		
Multifactiorial ANCOVA		WT	Het	KO	*F*	*p* value	Power	WT vs Het	WT vs KO	Het vs KO		*F*	*p* value	Power	*F*	*p* value	Power
Suspension time (seconds) - P11	Nonnormal	5.15 ± 1.44	4.37 ± 0.63	2.7 ± 0.47	1.701	0.194	0.338	−	−	−		0.045	0.833	0.055	0.287	0.752	0.093
Suspension time (seconds) - P12	Nonnormal	3.23 ± 1.06	3.13 ± 0.45	3.9 ± 1.65	0.220	0.803	0.082	−	−	−		0.856	0.360	0.148	0.662	0.521	0.154
Suspension time (seconds) - P13	Nonnormal	2.69 ± 0.47	4 ± 0.52	2.8 ± 0.87	1.660	0.202	0.331	−	−	−		2.518	0.120	0.342	0.036	0.965	0.055
Suspension time (seconds) - P14	Nonnormal	7.61 ± 1.97	5.17 ± 0.58	3.7 ± 1.12	2.196	0.123	0.425	−	−	−		0.006	0.938	0.051	1.893	0.163	0.372
Suspension time (seconds) - P15	Nonnormal	9.92 ± 2.29	4.82 ± 0.53	1.7 ± 0.42	10.137	**0.000**	0.980	**0.002**	**0.000**	0.054		0.290	0.593	0.082	0.611	0.547	0.146
Suspension time (seconds) - P16	Nonnormal	13.38 ± 2.18	6.41 ± 0.53	3.7 ± 0.91	15.666	**0.000**	0.999	**0.000**	**0.000**	0.100		0.744	0.393	0.135	1.971	0.151	0.386
Suspension time (seconds) - P17	Nonnormal	18.53 ± 2.34	11.82 ± 1.22	9.3 ± 1.6	6.683	**0.003**	0.896	**0.004**	**0.002**	0.288		0.538	0.467	0.111	1.214	0.307	0.251
Suspension time (seconds) - P18	Nonnormal	16.15 ± 2.22	18.34 ± 1.78	11.1 ± 2.37	2.398	0.103	0.459	−	−	−		0.551	0.462	0.112	0.722	0.491	0.164
Suspension time (seconds) - P19	Nonnormal	18.38 ± 2.11	19.48 ± 1.73	8 ± 1.97	7.474	**0.002**	0.927	0.921	**0.003**	**0.001**		0.000	0.995	0.050	1.609	0.212	0.322
Suspension time (seconds) - P20	Nonnormal	17.07 ± 2.31	12.13 ± 1.54	5.6 ± 0.85	6.858	**0.003**	0.903	0.053	**0.001**	**0.019**		0.646	0.426	0.123	1.283	0.287	0.264
Suspension time (seconds) - Average	Nonnormal	11.21 ± 1	8.97 ± 0.55	5.25 ± 0.54	13.553	**0.000**	0.997	**0.013**	**0.000**	**0.001**		0.303	0.585	0.084	2.871	0.067	0.534
Suspension time (seconds) - Best score	Nonnormal	24.3 ± 2.06	23.31 ± 1.37	13.7 ± 1.99	7.828	**0.001**	0.938	0.525	**0.001**	**0.001**		0.168	0.684	0.069	1.205	0.309	0.250
																	
**Open field**																	
Repeated measures, sphericity violated		*F*	*p* value	Power	WT vs Het	WT vs KO	Het vs KO										
Day effect		31.056	**0.000**	1.000	−	−	−										
Day × genotype effect		0.874	0.572	0.501	−	−	−										
Day × gender effect		0.630	0.702	0.247	−	−	−										
Day × genotype × gender effect		1.857	**0.042**	0.887	−	−	−										
Genotype effect		0.117	0.890	0.067	−	−	−										
Gender effect		0.046	0.831	0.055	−	−	−										
Genotype × gender		1.755	0.185	0.348	−	−	−										
												Gender effect			Gender × genotype effect		
Multifactiorial ANCOVA		WT	Het	KO	*F*	*p* value	Power	WT vs Het	WT vs KO	Het vs KO		*F*	*p* value	Power	*F*	*p* value	Power
Time to escape (seconds) - P8	Nonnormal	26.07 ± 2.09	27.55 ± 1.19	28.9 ± 0.99	0.473	0.626	0.122	−	−	−		0.928	0.341	0.156	0.010	0.990	0.051
Time to escape (seconds) - P9	Nonnormal	23.3 ± 2.82	21.34 ± 1.83	24.2 ± 2.64	0.305	0.739	0.095	−	−	−		0.774	0.384	0.138	0.929	0.403	0.201
Time to escape (seconds) - P10	Nonnormal	20.46 ± 2.55	19.96 ± 1.72	22.1 ± 2.34	0.286	0.753	0.093	−	−	−		0.010	0.922	0.051	1.615	0.210	0.323
Time to escape (seconds) - P11	Nonnormal	22.07 ± 2.18	17.55 ± 1.94	20.2 ± 3.04	0.938	0.399	0.202	−	−	−		1.030	0.316	0.168	3.307	0.046	0.597
Time to escape (seconds) - P12	Nonnormal	17 ± 2.34	19.27 ± 1.49	16.2 ± 2.09	0.660	0.522	0.154	−	−	−		0.718	0.401	0.132	1.010	0.372	0.215
Time to escape (seconds) - P13	Nonnormal	15.61 ± 2.38	18.2 ± 1.72	15.6 ± 2.25	0.573	0.568	0.139	−	−	−		0.015	0.902	0.052	0.685	0.509	0.158
Time to escape (seconds) - P14	Nonnormal	17.23 ± 2.78	12.72 ± 1.6	10.6 ± 1.14	2.349	0.107	0.450	−	−	−		0.421	0.520	0.097	5.349	0.008	0.815
Time to escape (seconds) - P15	Nonnormal	4.92 ± 0.38	5.65 ± 0.39	7 ± 1.46	1.768	0.183	0.350	−	−	−		0.215	0.645	0.074	0.019	0.981	0.053
Time to escape (seconds) - P16	Nonnormal	3.61 ± 0.28	3.65 ± 0.25	4.5 ± 0.87	1.119	0.336	0.234	−	−	−		2.068	0.158	0.290	0.043	0.958	0.056
Time to escape (seconds) - P17	Nonnormal	2.46 ± 0.24	3.48 ± 0.26	3.6 ± 0.37	3.840	**0.029**	0.667	**0.014**	**0.026**	0.745		1.782	0.189	0.257	1.179	0.317	0.245
Time to escape (seconds) - P18	Nonnormal	2.23 ± 0.32	2.1 ± 0.21	2.1 ± 0.4	0.028	0.972	0.054	−	−	−		0.121	0.730	0.063	0.313	0.733	0.097
Time to escape (seconds) - P19	Nonnormal	2.38 ± 0.33	2.17 ± 0.29	2 ± 0.36	0.011	0.989	0.051	−	−	−		1.947	0.170	0.276	2.592	0.086	0.490
Time to escape (seconds) - P20	Nonnormal	1.92 ± 0.21	2.13 ± 0.16	1.9 ± 0.17	0.632	0.536	0.149	−	−	−		0.044	0.834	0.055	0.205	0.816	0.080
Time to escape (seconds) - average	Normal	12.25 ± 0.59	11.98 ± 0.49	12.22 ± 0.53	0.117	0.890	0.067	−	−	−		0.046	0.831	0.055	1.755	0.185	0.348
First day of two consecutive successes (30-s cutoff)	Nonnormal	11.92 ± 0.58	11.24 ± 0.32	11.5 ± 0.4	0.589	0.559	0.142	−	−	−		0.701	0.407	0.130	0.297	0.745	0.094
																	
**Ultrasonic vocalizations**																	
Number of calls - repeated measures, sphericity assumed		*F*	*p* value	Power	WT vs Het	WT vs KO	Het vs KO										
Day effect		4.600	0.012	0.767	−	−	−										
Day × genotype effect		0.991	0.416	0.303	−	−	−										
Day × gender effect		1.430	0.244	0.300	−	−	−										
Day × genotype × gender effect		0.305	0.874	0.116	−	−	−										

Genotype effect		0.533	0.590	0.133	−	−	−										
Gender effect		1.697	0.199	0.248	−	−	−										
Genotype × gender		0.869	0.426	0.191	−	−	−										
												Gender effect			Gender × genotype effect		
Multifactiorial ANCOVA		WT	Het	KO	*F*	*p* value	Power	WT vs Het	WT vs KO	Het vs KO		*F*	*p* value	Power	*F*	*p* value	Power
Number of calls - minute 1	Nonnormal	13.81 ± 4.24	11.46 ± 2.89	15.88 ± 5.31	0.586	0.562	0.139	−	−	−		0.163	0.689	0.068	0.090	0.914	0.063
Number of calls - minute 2	Nonnormal	18.68 ± 5.15	11.56 ± 2.73	13.22 ± 4.78	0.567	0.572	0.136	−	−	−		0.327	0.571	0.086	0.068	0.935	0.059
Number of calls - minute 3	Nonnormal	15.75 ± 4.72	13.96 ± 3.72	8.33 ± 4.2	0.172	0.843	0.074	−	−	−		3.481	0.071	0.442	0.097	0.908	0.064
Number of calls - total	Nonnormal	48.43 ± 13.28	37.06 ± 7.85	37.44 ± 12.08	0.156	0.856	0.072	−	−	−		1.323	0.258	0.201	0.052	0.949	0.057
Calling time - minute 1	Nonnormal	0.93 ± 0.29	0.75 ± 0.19	1.08 ± 0.38	0.738	0.485	0.165	−	−	−		0.106	0.746	0.062	0.083	0.920	0.062
Calling time - minute 2	Nonnormal	1.24 ± 0.39	0.76 ± 0.19	0.85 ± 0.3	0.368	0.695	0.104	−	−	−		0.231	0.634	0.075	0.059	0.943	0.058
Calling time - minute 3	Nonnormal	1.16 ± 0.4	0.96 ± 0.25	0.53 ± 0.27	0.162	0.851	0.073	−	−	−		4.123	**0.050**	0.505	0.006	0.994	0.051
Calling time - total	Nonnormal	3.41 ± 1.05	2.48 ± 0.52	2.47 ± 0.81	0.189	0.828	0.077	−	−	−		1.431	0.240	0.213	0.023	0.977	0.053
Average duration - min 1	Nonnormal	0.05 ± 0	0.05 ± 0	0.03 ± 0.01	0.138	0.872	0.069	−	−	−		0.056	0.815	0.056	0.950	0.400	0.197
Average duration - min 2	Nonnormal	0.06 ± 0	0.06 ± 0	0.03 ± 0.01	0.319	0.730	0.095	−	−	−		1.879	0.182	0.262	1.561	0.229	0.301
Average duration - min 3	Nonnormal	0.06 ± 0	0.06 ± 0	0.02 ± 0.01	6.759	**0.004**	0.883	−	−	−		23.838	**0.000**	0.997	7.350	**0.003**	0.909
Average duration - total	Nonnormal	0.08 ± 0.01	0.06 ± 0	0.03 ± 0.01	0.515	0.604	0.125	−	−	−		1.663	0.209	0.237	0.847	0.440	0.179
Latency to first call	Nonnormal	77.28 ± 17.98	80.04 ± 12.54	75.57 ± 27.65	0.411	0.665	0.113	−	−	−		0.155	0.696	0.067	3.311	**0.045**	0.602
Mean frequency - total	Nonnormal	71337.77 ± 1902.04	73128.73 ± 3879.33	75883.81 ± 3957.75	0.024	0.976	0.053	−	−	−		0.036	0.851	0.054	0.029	0.971	0.054
Mean amplitude - total	Nonnormal	78.43 ± 29.11	66.33 ± 20.80	37.15 ± 17.64	0.189	0.829	0.076	−	−	−		2.440	0.130	0.325	0.442	0.648	0.114
Percentage of noncaller	Nonnormal	18.75 ± 10.08	28.13 ± 8.08	33.33 ± 16.67	0.401	0.672	0.111	−	−	−		0.587	0.447	0.117	1.933	0.155	0.382

WT, wild-type mice; Het, heterozygous mice; KO, homozygous knock-out mice. Group values are reported as mean ± SEM. Bold font indicates significant results (*p* < 0.05). P: postnatal day.

**Figure 2.**
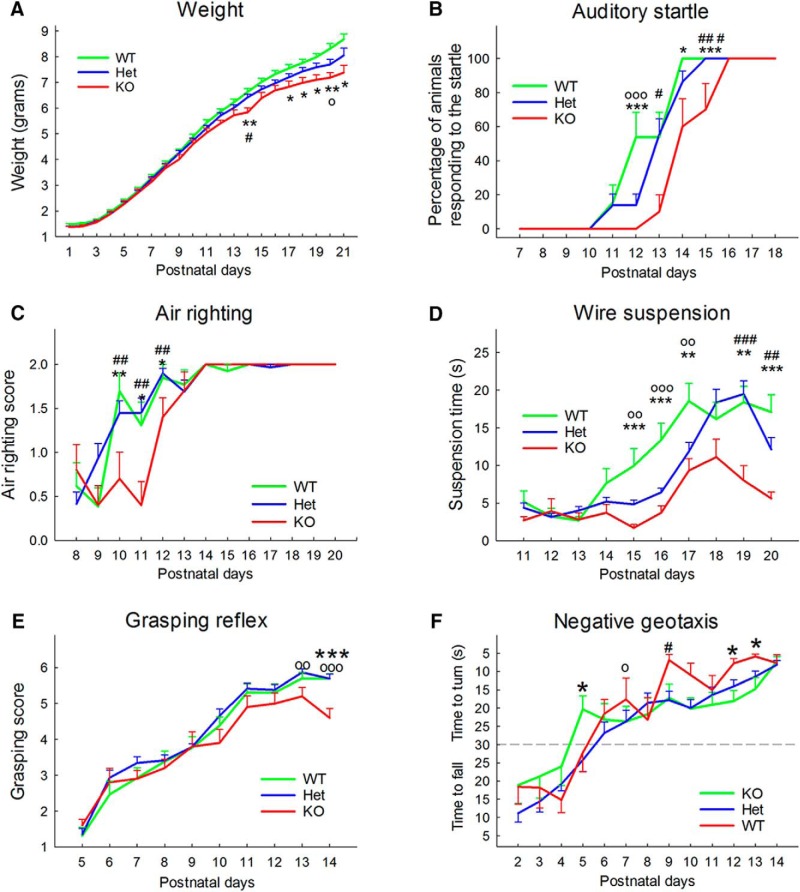
Delayed developmental milestones of in *Shank3^Δ4-22^*-deficient mice. Analysis of markers of developmental milestones revealed genotype differences in *Shank3^Δ4-22^* wild-type, heterozygous, and homozygous pups between postnatal days 1 and 21 on measures of (***A***) body weight, (***B***) auditory startle, (***C***) air righting, (***D***) wire suspension, (***E***) grasping reflex, and (***F***) negative geotaxis. Additional milestones (jar opening, tooth eruption, fur development, eye opening, rooting reflex, cliff aversion, ear twitch, surface righting, open field crossing, and ultrasonic vocalizations) are displayed in Extended Data Figure 2-1. WT, wild-type mice; Het, heterozygous mice; KO, homozygous knock-out mice. *: WT versus KO; o: WT versus Het, #: Het versus KO. *p < 0.05, **p < 0.1, ***p < 0.001.

Developmental delays were observed in the *Shank3^Δ4-22^*homozygote neonates in several of the parameters studied (Fig. 2; Extended Data Fig. 2-1; Table 4). While the birth weight was not significantly different, the growth rate of *Shank3^Δ4-22^*homozygote pups was slower and by P14, the weight of Shank3Δ4-22 homozygous mice was significantly lower than the weight of their wild-type littermates (Fig. 2*A*). Additionally, an unusual postnatal mortality was observed when breeding heterozygous animals together, with 6.9% of the pups dying between birth and P1. Eighty-six dead pups were genotyped, showing that the percentage of *Shank3^Δ4-22^*homozygote knock-out mice dying at or shortly after birth was higher than expected if the death was equally affecting all the genotypes (WT: *n* = 20, Het = 33, KO: *n* = 33, χ^2^ df2 = 8.66, *p* = 0.0137), this could explain, at least partially, the deficit observed at weaning. No differences were observed in any of the other physical developmental milestones, including eye opening, ear opening, tooth eruption or fur development (Extended Data Fig. 2-1*A–D*; Table 4).

10.1523/ENEURO.0046-18.2018.f2-1 Extended Data Figure 2-1 **Normal developmental milestones of Shank3^Δ4-22^ deficient mice**. Analysis of the markers of developmental milestones that revealed no major genotype differences in Shank3 wild-type, heterozygous and homozygous pups between postnatal days 1 and 21 on measures of (A) ear opening, (B) tooth eruption, (C) fur development, (D) eye opening, (E) rooting reflex, (F) cliff aversion, (G) ear twitch, (H) surface righting and (I) open field crossing. (K) Only non-significant differences were detected in the number and quality of ultrasonic vocalizations emitted by 6-day old pups. WT, wild-type mice; Het, heterozygous mice; KO, homozygous knockout mice. *: WT vs KO; o: WT vs Het, #: Het vs KO. *: p < 0.05, **: p < 0.1, ***: p < 0.001. Download Figure 2-1, TIF file.

A significant delay was observed for *Shank3* ^Δ4-22^ homozygotes in the response to auditory startle (Fig. 2*B*) and in the mid-air righting task (Fig. 2*C*) although all the mice were able to properly respond at the end of the observation period. In the wire suspension (Fig. 2*D*) and grasping reflex (Fig. 2*E*) tasks, however, not only was the acquisition of the response delayed, but *Shank3^Δ4-22^*homozygous animals remained significantly impaired until the time of weaning. In the negative geotaxis test, an initial delay was observed at P5 were most wild-type animals were able to turn while homozygous and heterozygous *Shank3^Δ4-22^*animals were still falling or staying in the starting position (Fig. 2*F*). Moreover, after P9 when most of the animals were able to master the task, higher reactivity (characterized by a shorter latency to turn) was observed for the *Shank3^Δ4-22^*homozygous mice. The acquisition of the rooting reflex was similar for the three groups; however, a premature disappearance of the reflex was observed in both the *Shank3^Δ4-22^*heterozygous and homozygous pups (Extended Data Fig. 2-1*E*; Table 4).

Other sensory-motor and neurologic milestones such as cliff aversion, ear twitch, surface righting, negative geotaxis, and open field crossing (Extended Data Fig. 2-1*F–I* ; Table 4) were not significantly affected by the disruption of the *Shank3* gene.

Ultrasonic vocalizations were recorded at postnatal day 6 on an independent cohort of mice and a genotype difference was detected in the number and quality of ultrasonic vocalizations emitted by the pups (Table 4). *Shank3^Δ4-22^* heterozygous and homozygous mice emitted fewer ultrasonic vocalizations than wild-type littermates (Extended Data Fig. 2-1*K*; Table 4). The total calling time was also affected with *Shank3^Δ4-22^*-deficient mice both spending less time calling and having shorter calls than wild-type littermates. Additionally, the peak amplitude was shorter in *Shank3^Δ4-22^*-deficient mice. However, none of these parameters were significantly different, probably due to a high interindividual variability within each group with some animals emitting no vocalizations during the 3-min recording. The percentage of noncallers was higher, although not significantly, in *Shank3^Δ4-22^*-deficient animals. Genotype did not affect the latency to the first call or the peak frequency of calls and no difference was observed in the time course of the emission of ultrasonic vocalizations.

### Adult general health in *Shank3^Δ4-22^*-deficient mice

Adult *Shank3^Δ4-22^* mice were evaluated for general health at three months of age (Table 5). The three genotypes did not differ on physical measure of weight and length. Additional weight measures at the age of 15 and 20 months showed a trend in reduced weight of *Shank3^Δ4-22^*homozygous mice compared to their littermates. Genotypes scored similarly and in the normal range for other physical characteristics including coat appearance (grooming, piloerection, patches of missing fur on face or body), skin pigmentation, whisker appearance, wounding, and palpebral closure. Observation in a beaker or after transfer to a housing cage revealed no abnormalities in term of spontaneous general activity, stereotypies (rears, jumps, circling, wild running), transfer arousal, gait, pelvic, and tail elevation.

**Table 5.** Detailed results and statistical analyses related to general health, physical factors, gross appearance, and spontaneous activity

**Physical factors and gross appearance**																	
						Genotype			Cohort			Genotype × cohort			Pairwise comparisons		
	Test	Data structure	WT	Het	KO	*F*	*p* value	Power	*F*	*p* value	Power	*F*	*p* value	Power	WT vs Het	WT vs KO	Het vs KO
3-months weight (g)	2wANOVA	Normal	26.33 ± 0.87	27.18 ± 0.57	26.01 ± 0.54	1.241	0.298	0.258	2.546	0.117	0.347	2.000	0.146	0.394	−	−	−
15-months weight (g)	2wANOVA	Normal	33.41 ± 2	31.06 ± 1.21	29.36 ± 1.73	1.578	0.222	0.310	0.269	0.608	0.079	0.491	0.617	0.123	−	−	−
20-months weight (g)	2wANOVA	Normal	32.9 ± 1.86	31.43 ± 1.2	28.84 ± 1.5	0.982	0.390	0.199	0.034	0.856	0.054	0.018	0.982	0.052	−	−	−
Length	2wANOVA	Nonnormal	16.45 ± 0.24	16.84 ± 0.24	16.73 ± 0.25	1.062	0.353	0.226	91.207	**0.000**	1.000	0.471	0.627	0.123	−	−	−
Coat appearance	2wANOVA	Nonnormal	2.63 ± 0.13	2.89 ± 0.07	2.94 ± 0.05	2.558	*0.087*	0.489	2.615	0.112	0.355	1.424	0.250	0.291	0.119	**0.050**	0.915
Skin color	−	−	0 ± 0	0 ± 0	0 ± 0	−	−	−	−	−	−	−	−	−	−	−	−
Whisker barbering	−	−	0 ± 0	0 ± 0	0 ± 0	−	−	−	−	−	−	−	−	−	−	−	−
Missing fur on face	−	−	0 ± 0	0 ± 0	0 ± 0	−	−	−	−	−	−	−	−	−	−	−	−
Missing fur on body	−	−	0 ± 0	0 ± 0	0 ± 0	−	−	−	−	−	−	−	−	−	−	−	−
Wounding	2wANOVA	Nonnormal	0.15 ± 0.11	0 ± 0	0.05 ± 0.05	1.078	0.348	0.229	0.028	0.869	0.053	0.279	0.758	0.092	−	−	−
Body tone	2wANOVA	Nonnormal	1.1 ± 0.07	1.1 ± 0.07	1 ± 0.07	0.563	0.573	0.138	2.225	0.142	0.310	0.563	0.573	0.138	−	−	−
Palpebral closure	−	−	0 ± 0	0 ± 0	0 ± 0	−	−	−	−	−	−	−	−	−	−	−	−
Piloerection	−	−	0 ± 0	0 ± 0	0 ± 0	−	−	−	−	−	−	−	−	−	−	−	−
																	
**Jar observation**	****	****	****	****	****	****	****	****	****	****	****	****	****	****	****	****	****
						Genotype			Cohort			Genotype × cohort			Pairwise comparisons		
	Test	Data structure	WT	Het	KO	*F*	*p* value	Power	*F*	*p* value	Power	*F*	*p* value	Power	WT vs Het	WT vs KO	Het vsKO
Body position	2wANOVA	Nonnormal	4.15 ± 0.08	4.1 ± 0.07	4.26 ± 0.1	0.702	0.500	0.162	4.949	0.031	0.588	0.139	0.871	0.070	−	−	−
Spontaneous activity	2wANOVA	Nonnormal	1.68 ± 0.1	1.57 ± 0.11	1.63 ± 0.11	0.223	0.801	0.083	0.282	0.598	0.082	0.279	0.758	0.092	−	−	−
Latency to sit/stand (s)	−	−	0 ± 0	0 ± 0	0 ± 0	−	−	−	−	−	−	−	−	−	−	−	−
Latency to rear (s)	2wANOVA	Nonnormal	8.94 ± 1.36	8.05 ± 1.03	5.63 ± 1.02	2.137	0.128	0.418	0.036	0.850	0.054	0.046	0.955	0.057	−	−	−
Repeated jumps (%)	2wANOVA	Nonnormal	15.78 ± 8.59	10.52 ± 7.23	26.31 ± 10.37	0.702	0.500	0.162	4.949	**0.031**	0.588	0.139	0.871	0.070	−	−	−
Circling (%)	2wANOVA	Nonnormal	5.26 ± 5.26	10.52 ± 7.23	10.52 ± 7.23	0.289	0.750	0.093	5.177	**0.027**	0.607	0.289	0.750	0.093	−	−	−
Urination	2wANOVA	Nonnormal	0.47 ± 0.19	0.1 ± 0.1	0.15 ± 0.11	1.540	0.224	0.312	0.023	0.881	0.052	1.213	0.306	0.253	−	−	−
Defecation (number)	2wANOVA	Nonnormal	1.57 ± 0.35	0.94 ± 0.29	0.94 ± 0.27	1.065	0.352	0.226	0.003	0.958	0.050	2.592	0.085	0.494	−	−	−
Respiration	−	−	2 ± 0	2 ± 0	2 ± 0	−	−		−	−		−	−		−	−	−
Tremor	−	−	0 ± 0	0 ± 0	0 ± 0	−	−		−	−		−	−		−	−	−
																	
**Cage transfer**	****	****	****	****	****	****	****	****	****	****	****	****	****	****	****	****	****
						Genotype			Cohort			Genotype × cohort			Pairwise comparisons		
	Test	Data structure	WT	Het	KO	*F*	*p* value	Power	*F*	*p* value	Power	*F*	*p* value	Power	WT vs Het	WT vs KO	Het vs KO
Transfer arousal	2wANOVA	Nonnormal	3.21 ± 0.22	3.21 ± 0.22	3.15 ± 0.2	0.037	0.964	0.055	1.470	0.231	0.221	2.055	0.139	0.404	−	−	−
Gait	2wANOVA	Nonnormal	0 ± 0	0.15 ± 0.08	0.05 ± 0.05	1.783	0.178	0.356	0.003	0.957	0.050	0.637	0.533	0.151	−	−	−
Pelvic elevation	2wANOVA	Nonnormal	2 ± 0	2.15 ± 0.08	2 ± 0.07	1.730	0.188	0.347	0.141	0.709	0.066	0.141	0.869	0.071	−	−	−
Tail elevation	2wANOVA	Nonnormal	1.89 ± 0.15	1.73 ± 0.18	1.21 ± 0.18	4.003	**0.024**	0.691	4.469	0.039	0.545	0.204	0.816	0.080	0.530	**0.009**	**0.043**

WT, wild-type mice; Het, heterozygous mice; KO, homozygous knock-out mice. Group values are reported as mean ± SEM. Bold font indicates significant results (*p* < 0.05). Individual results and statistical analyses for cohorts 1 and 2 are available in Extended Data Table 5-1. 2wANOVA: 2-way ANOVA.

10.1523/ENEURO.0046-18.2018.t5-1 Extended Data Table 5-1 **Individual results and statistical analyses for cohorts 1 and 2 related to general health, physical factors, gross appearance and spontaneous activity.** WT, wild-type mice; Het, heterozygous mice; KO, homozygous knockout mice. Group values are reported as means ± s.e.m. Red font indicates significant results (p < 0.05), orange font indicates trends (0.1 < p < 0.05). Download Table 5-1, DOCX file.

### Motor functions in *Shank3^Δ4-22^*-deficient mice

**Figure 3.**
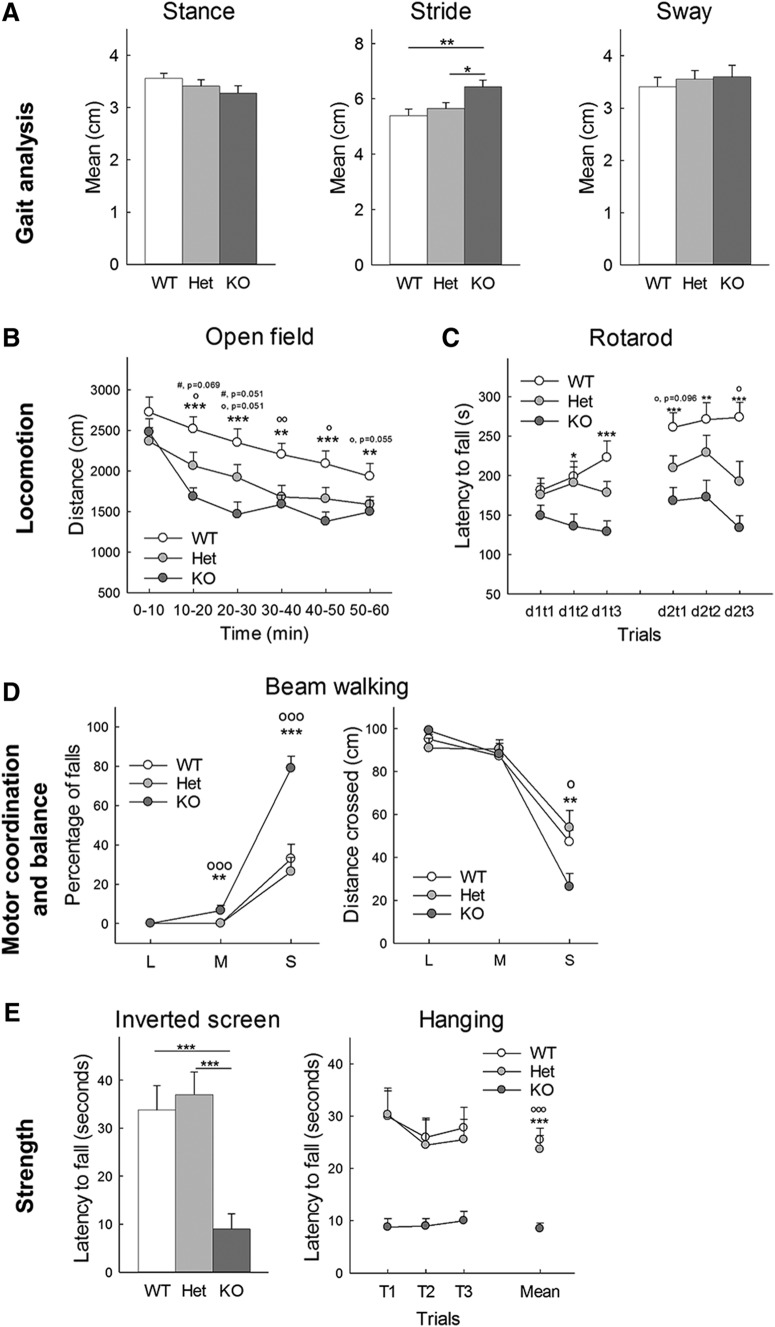
Impaired motor performances in in *Shank3^Δ4-22^*-deficient mice. ***A***, Average stance, stride, and sway. Gait analysis showed an increase stride length in *Shank3^Δ4-22^* homozygous mice. ***B***, Distance traveled during a 60-min session in an open field. Spontaneous locomotor activity in the open field was reduced in *Shank3^Δ4-22^* homozygous mice relative to other genotypes. ***C***, Latency to fall over six trials (three trials per day for two consecutive days) in the accelerating rotarod task. Motor learning on the accelerating rotarod was deficient in *Shank3^Δ4-22^* homozygous mice compared to wild-type animals as they failed to improve over time. Heterozygous mice had an intermediate phenotype. ***D***, Percentage of falls and distance crossed during the beam walking test. While not different on the large (L, 1 inch) and medium (M, ½ inch) beams, *Shank3^Δ4-22^* homozygous mice were strongly impaired in the small (S, ¼ inch) beam walking test, as shown by a significant increase of the number of falls and a decrease of the distance crossed. ***E***, Strength and endurance measured in the inverted screen and hanging tests. Endurance strength was significantly impaired in *Shank3^Δ4-22^* homozygous mice as they exhibited significantly shorter latency to fall in both the inverted screen and hanging tests. Additional results of motor tests (hindlimb placing and grip strength) are available in Extended Data Figure 3-1. WT, wild-type mice; Het, heterozygous mice; KO, homozygous knock-out mice. *: WT versus KO; o: WT versus Het, #: Het versus KO. *p < 0.05, **p < 0.1, ***p < 0.001.

Motor functions were examined using several different paradigms (Table 6). Footprint gait analysis showed normal stance and sway but increased stride in *Shank3^Δ4-22^*homozygous mice compared to wild-type and heterozygous animals (Fig. 3*A*) and reduced spontaneous locomotion was observed during a 1-h open field session in both *Shank3^Δ4-22^* heterozygous and homozygous mice (Fig. 3*B*). Across the 60-min session, the time course for total distance traversed by all three genotypes declined as expected, representing habituation to the open field. However, while the distance traveled during the first 10 min was similar for the three groups, the decline was faster for *Shank3^Δ4-22^* homozygous mice, possibly reflecting a higher fatigability. Similarly, in the accelerating rotarod test, which assay for gait, balance, motor coordination and endurance, shorter latencies to fall where observed in *Shank3^Δ4-22^-*deficient mice after the first trial, with a milder phenotype observed in the heterozygotes compared to homozygotes. When examining learning in this paradigm, characterized by an improvement of performance (latency to fall) over the trials, *Shank3^Δ4-22^* heterozygous and homozygous animals failed to improve over time, in contrast to wild-type animals which showed typical learning (Fig. 3*C*).

10.1523/ENEURO.0046-18.2018.f3-1 Extended Data Figure 3-1 **Motor functions in in Shank3^Δ4-22^ -deficient mice.** (A) Number of failed attempts (falls or hanging without being able to pull itself up in less than 60 seconds) in the hind placing test. The number of failed attempts was more important in Shank3^Δ4-22^ homozygous mice. (B) Strength measured in the grip strength. No genotype difference was found for acute grip strength. WT, wild-type mice; Het, heterozygous mice; KO, homozygous knockout mice. *: WT vs KO; o: WT vs Het, #: Het vs KO. *: p < 0.05, **: p < 0.1, ***: p < 0.001. Download Figure 3-1, TIF file.

**Table 6.** Detailed results and statistical analyses related to motor functions

**Gait analysis**																	
						Genotype			Cohort			Genotype ×cohort			Pairwisecomparisons		
	Test	Datastructure	WT	Het	KO	*F*	*p*value	Power	*F*	*p*value	Power	*F*	*p*value	Power	WT vsHet	WT vsKO	Het vs KO
Stance mean (cm)	2wANOVA	Normal	3.55 ± 0.09	3.41 ± 0.12	3.27 ± 0.14	1.466	0.240	0.299	40.902	**0.000**	1.000	1.291	0.284	0.267	−	−	−
Stance variance (cm)	2wANOVA	Nonnormal	0.15 ± 0.04	0.15 ± 0.03	0.17 ± 0.05	0.189	0.829	0.078	14.972	**0.000**	0.967	0.735	0.484	0.168	−	−	−
Stride mean (cm)	2wANOVA	Normal	5.39 ± 0.23	5.64 ± 0.21	6.43 ± 0.24	5.443	**0.007**	0.826	15.476	**0.000**	0.971	0.499	0.610	0.127	0.674	**0.003**	**0.028**
Stride variance (cm)	2wANOVA	Nonnormal	0.77 ± 0.13	0.86 ± 0.14	0.83 ± 0.18	0.137	0.873	0.070	12.150	**0.001**	0.928	3.459	**0.039**	0.622	−	−	−
Sway mean (cm)	2wANOVA	Nonnormal	3.4 ± 0.17	3.55 ± 0.16	3.59 ± 0.22	0.191	0.826	0.078	186.368	**0.000**	1.000	2.443	*0.097*	0.470	−	−	−
Sway variance (cm)	2wANOVA	Nonnormal	0.08 ± 0.01	0.17 ± 0.04	0.14 ± 0.02	1.909	0.159	0.378	3.781	*0.057*	0.479	0.400	0.672	0.111	−	−	−
																	
**Open field spontaneous activity (traveled distance)**																	
						Genotype			Cohort			Genotype × cohort			Pairwise comparisons		
	Test	Data structure	WT	Het	KO	*F*	*p* value	Power	*F*	*p* value	Power	*F*	*p* value	Power	WT vs Het	WT vs KO	Het vsKO
Total distance (cm)	2wANOVA	Normal	13,816.17 ± 828.27	11,273.16 ± 764.09	10,099.24 ± 621.43	6.633	**0.003**	0.896	12.836	**0.001**	0.940	0.073	0.930	0.061	**0.016**	**0.001**	0.299
																	
Distance	Test	Data structure				*F*	*p* value	Power	WT vs Het	WT vs KO	Het vsKO						
- Time	rMeasures	Sph.viol				36.350	**0.000**	1.000	−	−	−						
- Time × gen.	rMeasures	Sph.viol				2.235	**0.029**	0.917	−	−	−						
- Genotype	rMeasures	Sph.viol				6.633	**0.003**	0.896	**0.029**	**0.001**	0.449						
- Cohort	rMeasures	Sph.viol				12.836	**0.001**	0.940	−	−	−						
- Time × gen. × coh.	rMeasures	Sph.viol				0.878	0.532	0.461	−	−	−						
- gen. × coh.	rMeasures	Sph.viol				0.073	0.930	0.061	−	−	−						
																	
						Genotype			Cohort			Genotype × cohort			Pairwise comparisons		
Individual time bins	Test	Data structure	WT	Het	KO	*F*	*p* value	Power	*F*	*p* value	Power	*F*	*p* value	Power	WT vs Het	WT vs KO	Het vsKO
Distance 0-10 min	2wANOVA	Normal	2723.06 ± 185.28	2365.11 ± 166.4	2479.64 ± 164.36	0.894	0.415	0.196	7.573	**0.008**	0.770	0.224	0.800	0.083	−	−	−
Distance 10-20 min	2wANOVA	Nonnormal	2516.77 ± 150.09	2064.33 ± 169.75	1684.88 ± 108.32	8.357	**0.001**	0.954	13.148	**0.001**	0.945	0.026	0.975	0.054	**0.030**	**0.000**	*0.069*
Distance 20-30 min	2wANOVA	Normal	2349.52 ± 168.15	1919.27 ± 159.57	1466.99 ± 150.82	7.936	**0.001**	0.943	11.962	**0.001**	0.924	0.362	0.698	0.105	*0.051*	**0.000**	*0.051*
Distance 30-40 min	2wANOVA	Normal	2203.27 ± 139.79	1680.09 ± 143.29	1589.41 ± 114.03	6.091	**0.004**	0.868	11.521	**0.001**	0.915	0.020	0.980	0.053	**0.007**	**0.002**	0.710
Distance 40-50 min	2wANOVA	Normal	2090.38 ± 156.71	1657.47 ± 139.76	1380 ± 116.11	6.255	**0.004**	0.877	5.904	**0.019**	0.664	0.035	0.965	0.055	**0.035**	**0.001**	0.185
Distance 50-60 min	2wANOVA	Nonnormal	1933.14 ± 160.59	1586.87 ± 98.6	1498.28 ± 139.38	3.074	*0.055*	0.568	3.755	*0.058*	0.477	1.459	0.242	0.298	*0.055*	**0.026**	0.733
																	
**Rotarod**	****	****	****	****	****	****	****	****	****	****	****	****	****	****	****	****	****
Latency	Test	Data structure				*F*	*p* value	Power	WT vs Het	WT vs KO	Het vsKO						
- Trial	rMeasures	Sph.viol				9.369	**0.000**	1.000	−	−	−						
- Trial × gen.	rMeasures	Sph.viol				2.268	**0.015**	0.921	−	−	−						
- Genotype	rMeasures	Sph.viol				8.888	**0.000**	0.964	0.123	**0.000**	**0.044**						
- Cohort	rMeasures	Sph.viol				2.573	0.115	0.350	−	−	−						
- Session × gen. × coh.	rMeasures	Sph.viol				1.867	**0.050**	0.848	−	−	−						
- Gen. × coh.	rMeasures	Sph.viol				0.726	0.489	0.166	−	−	−						
																	
						Genotype			Cohort			Genotype × cohort			Pairwise comparisons		
Individual trials	Test	Data structure	WT	Het	KO	*F*	*p*value	Power	*F*	*p*value	Power	*F*	*p*value	Power	WT vs Het	WT vs KO	Het vsKO
Latency trial 1	2wANOVA	Normal	181.28 ± 15.25	175.51 ± 14.58	149.17 ± 13.24	1.323	0.275	0.273	0.053	0.819	0.056	1.320	0.276	0.273	−	−	−
Latency trial 2	2wANOVA	Normal	198.66 ± 19.58	190.76 ± 20.22	135.84 ± 15.82	3.395	**0.041**	0.614	0.001	0.979	0.050	3.956	**0.025**	0.685	0.947	**0.042**	0.085
Latency trial 3	2wANOVA	Normal	222.75 ± 20.99	178.3 ± 14.43	128.94 ± 14.01	7.010	**0.002**	0.913	1.047	0.311	0.171	0.816	0.448	0.182	0.159	**0.001**	0.106
Latency trial 4	2wANOVA	Normal	260.92 ± 18.73	209.71 ± 15.54	168.01 ± 16.99	6.767	**0.002**	0.902	3.832	*0.056*	0.484	0.017	0.983	0.052	0.094	**0.001**	0.203
Latency trial 5	2wANOVA	Normal	270.8 ± 21.62	228.95 ± 22.25	172.55 ± 21.59	4.498	**0.016**	0.744	1.536	0.221	0.229	1.050	0.357	0.224	0.368	**0.007**	0.168
Latency trial 6	2wANOVA	Normal	273.51 ± 19.16	192.29 ± 25.69	133.8 ± 15.59	11.838	**0.000**	0.992	9.021	0.004	0.838	0.222	0.802	0.083	**0.013**	**0.000**	0.094
Latency day 1	2wANOVA	Normal	200.9 ± 15.13	181.52 ± 14.32	137.98 ± 11.9	5.026	**0.010**	0.793	0.108	0.744	0.062	2.253	0.115	0.438	0.578	**0.006**	*0.072*
Latency day 2	2wANOVA	Normal	268.41 ± 16.68	210.32 ± 19.07	158.12 ± 14.54	10.061	**0.000**	0.980	5.783	**0.020**	0.655	0.060	0.942	0.059	**0.041**	**0.000**	*0.073*
																	
**Beam walking**	****	****	****	****	****	****	****	****	****	****	****	****	****	****	****	****	****
						Genotype			Cohort			Genotype × cohort			Pairwisecomparisons		
	Test	Data structure	WT	Het	KO	*F*	*p* value	Power	*F*	*p* value	Power	*F*	*p* value	Power	WT vs Het	WT vs KO	Het vsKO
% mice falling (large)	2wANOVA	NA	0 ± 0	0 ± 0	0 ± 0	NA	NA	NA	NA	NA	NA	NA	NA	NA	−	−	−
% mice falling (medium)	2wANOVA	Nonnormal	0 ± 0	0 ± 0	6.57 ± 2.59	6.339	**0.003**	0.882	0.395	0.533	0.095	0.396	0.675	0.111	1.000	**0.003**	**0.003**
% mice falling (small)	2wANOVA	Nonnormal	32.89 ± 7.41	26.31 ± 7.27	78.94 ± 6.12	16.788	**0.000**	1.000	3.622	*0.063*	0.463	0.972	0.385	0.210	0.366	**0.000**	**0.000**
Distance (large,cm)	2wANOVA	Nonnormal	95.05 ± 2.27	90.92 ± 4.66	99.07 ± 0.92	1.819	0.173	0.362	1.416	0.240	0.215	0.242	0.786	0.086	−	−	−
Distance (medium, cm)	2wANOVA	Nonnormal	87.17 ± 4.25	90.41 ± 4.35	88.15 ± 5.03	0.129	0.879	0.069	2.525	0.118	0.344	0.482	0.620	0.125	−	−	−
Distance (small, cm)	2wANOVA	Normal	47.19 ± 5.45	53.77 ± 8.03	26.24 ± 6.14	4.380	**0.018**	0.732	0.447	0.507	0.101	0.346	0.709	0.102	0.438	**0.044**	**0.006**
% mice fully crossing (large)	2wANOVA	Nonnormal	94.73 ± 2.4	89.47 ± 5.83	98.68 ± 1.31	1.567	0.219	0.317	1.486	0.228	0.223	0.107	0.899	0.066	−	−	−
% mice fully crossing (medium)	2wANOVA	Nonnormal	76.31 ± 7.01	80.7 ± 7.12	80.26 ± 7.29	0.089	0.915	0.063	4.278	0.044	0.528	0.947	0.394	0.205	−	−	−
% mice fully crossing (small)	2wANOVA	Nonnormal	27.63 ± 7.12	38.4 ± 9.57	9.21 ± 5.47	3.568	**0.035**	0.637	0.692	0.409	0.129	0.316	0.730	0.098	0.278	0.128	**0.010**

Paw misplacements (large, all mice)	2wANOVA	Nonnormal	0.47 ± 0.14	0.56 ± 0.16	1.06 ± 0.14	4.197	**0.021**	0.712	0.137	0.713	0.065	1.662	0.200	0.334	0.693	**0.010**	**0.026**
Paw misplacements (medium, all mice)	2wANOVA	Normal	1.71 ± 0.29	1.5 ± 0.26	2.38 ± 0.41	1.762	0.182	0.352	0.525	0.472	0.110	0.055	0.947	0.058	−	−	*-*
Paw misplacements (small, all mice)	2wANOVA	Nonnormal	1.44 ± 0.13	2.48 ± 0.47	1.78 ± 0.18	3.330	**0.044**	0.605	3.700	*0.060*	0.471	0.760	0.473	0.172	**0.015**	0.462	0.082
Paw misplacements (large, crossing mice)	2wANOVA	Nonnormal	0.52 ± 0.17	0.6 ± 0.17	1.07 ± 0.14	3.194	**0.049**	0.585	0.210	0.649	0.073	1.333	0.273	0.275	0.937	**0.053**	0.119
Paw misplacements (medium, crossing mice)	2wANOVA	Nonnormal	1.67 ± 0.33	1.37 ± 0.27	2.33 ± 0.53	1.343	0.271	0.276	0.851	0.361	0.148	0.027	0.973	0.054	−	−	−
Paw misplacements (small, crossing mice)	2wANOVA	Nonnormal	1.79 ± 0.23	2.77 ± 0.53	2.81 ± 0.64	1.189	0.327	0.227	2.465	0.134	0.318	0.102	0.904	0.063	−	−	−
Time to cross (large, fully crossing)	2wANOVA	Nonnormal	10.05 ± 1.3	9.11 ± 1.91	7.17 ± 1.15	0.911	0.409	0.199	3.043	*0.087*	0.402	1.213	0.306	0.253	−	−	−
Time to cross (medium, fully crossing)	2wANOVA	Nonnormal	28.54 ± 5.32	16.62 ± 3.28	18.14 ± 4.47	1.643	0.204	0.330	2.898	*0.095*	0.386	2.415	0.100	0.464	−	−	−
Time to cross (small, fully crossing)	2wANOVA	Normal	54.56 ± 7.16	44.74 ± 5.25	22.43 ± 6	4.119	**0.030**	0.667	0.037	0.850	0.054	2.660	0.092	0.473	0.479	**0.030**	0.169
Time to cross (large, all mice)	2wANOVA	Nonnormal	15.75 ± 3.07	20.75 ± 6.73	8.5 ± 2.21	4.443	**0.027**	0.370	0.327	0.574	0.094	0.427	0.659	0.070	**0.015**	**0.033**	0.820
Time to cross (medium, all mice)	2wANOVA	Nonnormal	46.34 ± 8.74	36.11 ± 8.58	34.61 ± 8.93	1.157	0.337	0.109	1.444	0.245	0.682	0.472	0.632	0.218	−	−	−
Time to cross (small, all mice)	2wANOVA	Normal	99.92 ± 4.67	89.98 ± 7.71	111.65 ± 4.61	3.540	*0.051*	0.606	0.524	0.478	0.073	3.236	0.063	0.158	**0.017**	0.428	0.220
																	
**Motor reflexes**	****	****	****	****	****	****	****	****	****	****	****	****	****	****	****	****	****
						Genotype			Cohort			Genotype × cohort			Pairwise comparisons		
	Test	Data structure	WT	Het	KO	*F*	*p* value	Power	*F*	*p* value	Power	*F*	*p* value	Power	WT vs Het	WT vs KO	Het vsKO
Righting reflex	2wANOVA	Nonnormal	0.05 ± 0.05	0 ± 0	0 ± 0	0.721	0.491	0.165	0.727	0.398	0.133	0.721	0.491	0.165	−	−	−
Hindlimb placing, score	2wANOVA	Nonnormal	5.57 ± 0.24	5.26 ± 0.34	4.21 ± 0.57	2.778	*0.072*	0.524	0.093	0.762	0.060	0.117	0.890	0.067	0.618	**0.029**	*0.086*
Hindlimb placing, latency to climb	2wANOVA	Nonnormal	8.29 ± 1.61	7.21 ± 1.66	10.96 ± 2.57	0.836	0.439	0.186	0.333	0.567	0.087	0.117	0.890	0.067	−	−	−
Hindlimb placing, failed attempts	2wANOVA	Nonnormal	0.21 ± 0.12	0.36 ± 0.17	0.89 ± 0.28	2.778	*0.072*	0.524	0.093	0.762	0.060	0.117	0.890	0.067	0.618	**0.029**	*0.086*
Inverted screen, latency to fall	2wANOVA	Nonnormal	33.78 ± 5.09	37 ± 4.72	9 ± 3.13	11.464	**0.000**	0.991	0.701	0.406	0.130	0.645	0.529	0.152	0.522	**0.000**	**0.000**
Hanging, score	2wANOVA	Nonnormal	6.26 ± 0.18	6 ± 0.25	4.84 ± 0.27	10.223	**0.000**	0.982	2.691	0.107	0.363	0.834	0.440	0.185	0.486	**0.000**	**0.001**
Hanging, latency to fall	2wANOVA	Nonnormal	25.44 ± 2.27	23.66 ± 2.66	8.48 ± 1.05	18.838	**0.000**	1.000	0.269	0.606	0.080	0.816	0.448	0.182	0.643	**0.000**	**0.000**
																	
**Grip strength**	****	****	****	****	****	****	****	****	****	****	****	****	****	****	****	****	****
							
Latency	Test	Data structure				*F*	*p* value	Power	WT vs Het	WT vs KO	Het vsKO						
- Session	rMeasures	Sph.viol				3.520	**0.033**	0.644	−	−	−						
- Session × gen.	rMeasures	Sph.viol				1.971	0.105	0.575	−	−	−						
- Genotype	rMeasures	Sph.viol				0.324	0.725	0.099	−	−	−						
- Cohort	rMeasures	Sph.viol				47.402	**0.000**	1.000	−	−	−						
- Session × gen. × coh.	rMeasures	Sph.viol				2.687	**0.035**	0.729	−	−	−						
- Gen. × coh.	rMeasures	Sph.viol				1.044	0.359	0.223	−	−	−						
																	
						Genotype			Cohort			Genotype × cohort			Pairwise comparisons		
Individual trials	Test	Data structure	WT	Het	KO	*F*	*p* value	Power	*F*	*p* value	Power	*F*	*p* value	Power	WT vs Het	WT vs KO	Het vsKO
Session 1	2wANOVA	Normal	0.94 ± 0.05	0.99 ± 0.06	1.03 ± 0.06	0.502	0.608	0.128	25.973	**0.000**	0.999	1.564	0.219	0.317	−	−	−
Session 2	2wANOVA	Normal	0.86 ± 0.07	0.85 ± 0.06	1.01 ± 0.05	2.222	0.119	0.433	36.967	**0.000**	1.000	1.631	0.206	0.329	−	−	−
Session 3	2wANOVA	Normal	0.91 ± 0.06	0.92 ± 0.06	0.89 ± 0.06	0.320	0.728	0.098	23.585	**0.000**	0.997	1.883	0.163	0.374	−	−	−
Mean strength	2wANOVA	Normal	0.9 ± 0.05	0.92 ± 0.05	0.98 ± 0.04	0.324	0.725	0.099	47.402	**0.000**	1.000	1.044	0.359	0.223	−	−	−
Highest score	2wANOVA	Nonnormal	1.08 ± 0.05	1.07 ± 0.05	1.13 ± 0.05	0.243	0.785	0.086	26.793	**0.000**	0.999	0.821	0.446	0.183	−	−	−

WT, wild-type mice; Het, heterozygous mice; KO, homozygous knock-out mice. Group values are reported as mean ± SEM. Bold font indicates significant results (*p* < 0.05). Individual results and statistical analyses for cohorts 1 and 2 are available in Extended Data Table 6-1. 2wANOVA: 2-way ANOVA, rMeasures: repeated measures, Sph.viol: sphericity violated, gen: genotype, coh: cohort.

10.1523/ENEURO.0046-18.2018.t6-1 Extended Data Table 6-1 **Individual results and statistical analyses for cohorts 1 and 2 related to motor functions.** WT, wild-type mice; Het, heterozygous mice; KO, homozygous knockout mice. Group values are reported as means ± s.e.m. Red font indicates significant results (p < 0.05), orange font indicates trends (0.1 < p < 0.05). Download Table 6-1, DOCX file.

Impairment of motor coordination and balance was also observed in *Shank3^Δ4-22^* homozygous in the beam walking test (Fig. 3*D*; Table 6) as well by reduced strength and endurance in both the inverted screen and hanging tests (Fig. 3*E*), but with no differences in forelimb grip strength (Extended Data Fig. 3-1*A*). There was also a trend toward an increased number of failed attempt in the hindlimb placing for *Shank3^Δ4-22^* homozygous mice, compared to their littermates (Extended Data Fig. 3-1*B*).

### Sensory abilities in *Shank3^Δ4-22^*-deficient mice

For all sensory-related assays, detailed results are reported in Table 7.

**Table 7.** Detailed results and statistical analyses related to the sensory profile

**Reflexes and reactions to simple stimuli**																	
						Genotype			Cohort			Genotype × cohort			Pairwise comparisons		
	Test	Data structure	WT	Het	KO	*F*	*p* value	Power	*F*	*p* value	Power	*F*	*p* value	Power	WT vs Het	WT vs KO	Het vs KO
Pinna reflex	2wANOVA	Nonnormal	0.89 ± 0.07	0.68 ± 0.1	0.73 ± 0.1	1.790	0.1773	0.357	13.988	0.000	0.956	1.155	0.323	0.243	−	−	−
Cornel reflex	2wANOVA	Nonnormal	1.05 ± 0.05	0.94 ± 0.05	1.05 ± 0.05	1.518	0.2289	0.308	0.389	0.536	0.094	1.518	0.229	0.308	−	−	−
Toe pinch retraction	2wANOVA	Nonnormal	2.05 ± 0.37	2.36 ± 0.33	2.26 ± 0.43	0.144	0.8659	0.071	1.073	0.305	0.174	0.072	0.931	0.060	−	−	−
Preyer reflex	2wANOVA	Nonnormal	1.47 ± 0.15	1.36 ± 0.13	1.42 ± 0.17	0.135	0.8740	0.070	22.250	0.000	0.996	1.478	0.238	0.301	−	−	−
Visual placing	NA	NA	9 ± 0	9 ± 0	9 ± 0	−	−		−	−		−	−		−	−	−
																	
**Tail flick**	****	****	****	****	****	****	****	****	****	****	****	****	****	****	****	****	****
Latency	Test	Data structure				*F*	*p* value	Power	WT vs Het	WT vs KO	Het vs KO						
- Trial	rMeasures	Sph.viol				3.081	**0.0500**	0.583	−	−	−						
- Trial × gen.	rMeasures	Sph.viol				0.169	0.9535	0.085	−	−	−						
- Genotype	rMeasures	Sph.viol				1.118	0.3347	0.236	−	−	−						
- Cohort	rMeasures	Sph.viol				83.467	**0.0000**	1.000	−	−	−						
- Trial × gen. × coh.	rMeasures	Sph.viol				0.489	0.7438	0.162	−	−	−						
- Gen. × coh.	rMeasures	Sph.viol				2.162	0.1255	0.422	−	−	−						
																	
						Genotype			Cohort			Genotype × cohort			Pairwise comparisons		
Individual trials	Test	Data structure	WT	Het	KO	*F*	*p* value	Power	*F*	*p* value	Power	*F*	*p* value	Power	WT vs Het	WT vsKO	Het vs KO
Latency trial 1 (s)	2wANOVA	Normal	11.39 ± 0.97	11.32 ± 0.78	10.28 ± 0.82	0.356	0.7023	0.104	13.680	**0.001**	0.952	0.145	0.865	0.071	−	−	−
Latency trial 2 (s)	2wANOVA	Normal	10.99 ± 1.08	9.67 ± 0.98	8.86 ± 0.82	0.961	0.3892	0.208	36.614	**0.000**	1.000	2.286	0.112	0.444	−	−	−
Latency trial 3 (s)	2wANOVA	Normal	11.05 ± 1.23	10.06 ± 1.01	9.29 ± 0.94	0.526	0.5942	0.132	91.329	**0.000**	1.000	1.578	0.216	0.319	−	−	−
Shortest latency (s)	2wANOVA	Normal	8.03 ± 0.82	7.99 ± 0.87	7.47 ± 0.78	0.064	0.9379	0.059	42.964	**0.000**	1.000	1.039	0.361	0.222	−	−	−
Longest latency (s)	2wANOVA	Nonnormal	14.17 ± 0.94	12.82 ± 0.75	11.91 ± 0.72	2.213	0.1198	0.431	64.815	**0.000**	1.000	1.659	0.200	0.334	−	−	−
Mean latency (s)	2wANOVA	Nonnormal	11.14 ± 0.88	10.35 ± 0.78	9.48 ± 0.72	1.118	0.3347	0.236	83.467	**0.000**	1.000	2.162	0.126	0.422	−	−	−
																	
**Startle response**																	
						Genotype			Cohort			Genotype × cohort			Pairwise comparisons		
	Test	Data structure	WT	Het	KO	*F*	*p* value	Power	*F*	*p* value	Power	*F*	*p* value	Power	WT vsHet	WT vs KO	Het vs KO
Startle 74 dB	2wANOVA	Nonnormal	179.96 ± 20.24	165.57 ± 17.22	158.01 ± 10.36	2.376	0.1077	0.448	9.123	0.005	0.836	2.255	0.120	0.428	−	−	−
Startle 78 dB	2wANOVA	Nonnormal	183.48 ± 22	157.4 ± 16.93	160.69 ± 14.89	3.181	*0.0538*	0.571	5.779	0.022	0.647	3.673	**0.036**	0.638	**0.046**	**0.025**	0.770
Startle 82 dB	2wANOVA	Nonnormal	197.45 ± 26.03	160.09 ± 14.07	175.58 ± 13.87	3.254	**0.0500**	0.582	3.939	*0.055*	0.488	2.621	*0.087*	0.487	**0.019**	*0.063*	0.591
Startle 86 dB	2wANOVA	Nonnormal	246.33 ± 35.36	162.3 ± 15.61	176.15 ± 12.88	3.255	**0.0500**	0.582	0.082	0.777	0.059	0.271	0.764	0.089	**0.024**	**0.042**	0.812
Startle 92 dB	2wANOVA	Nonnormal	257.25 ± 40.4	192.63 ± 19.03	201.41 ± 23.01	2.153	0.1313	0.411	0.039	0.845	0.054	1.323	0.279	0.267	−	−	−
																	
Startle response	Test	Data structure				*F*	*p* value	Power	WT vs Het	WT vs KO	Het vs KO						
- Sound intensity	rMeasures	Sph.ass				2.900	*0.0510*	0.605	−	−	−						
- Sound intensity × gen.	rMeasures	Sph.ass				0.642	0.6620	0.219	−	−	−						
- Genotype	rMeasures	Sph.ass				3.649	**0.0364**	0.635	**0.022**	**0.024**	0.989						
- Cohort	rMeasures	Sph.ass				1.842	0.1835	0.262	−	−	−						
- Sound intensity × gen. × coh.	rMeasures	Sph.ass				0.822	0.5335	0.276	−	−	−						
- Gen. × coh.	rMeasures	Sph.ass				1.922	0.1614	0.372	−	−	−						
																	
						Genotype			Cohort			Genotype × cohort			Pairwise comparisons		
	Test	Data structure	WT	Het	KO	*F*	*p* value	Power	*F*	*p* value	Power	*F*	*p* value	Power	WT vs Het	WT vs KO	Het vs KO
Startle 74 dB norm. to weight	2wANOVA	Nonnormal	7.01 ± 0.82	6.18 ± 0.64	6.17 ± 0.32	2.843	*0.0718*	0.522	5.749	**0.022**	0.645	2.780	*0.076*	0.512	**0.045**	**0.042**	0.959
Startle 78 dB norm. to weight	2wANOVA	Nonnormal	7.19 ± 0.89	5.86 ± 0.64	6.35 ± 0.58	3.051	*0.0601*	0.553	2.972	*0.094*	0.389	3.611	**0.038**	0.630	**0.038**	**0.036**	0.964
Startle 82 dB norm. to weight	2wANOVA	Nonnormal	7.74 ± 1.06	5.89 ± 0.41	6.98 ± 0.63	3.378	**0.0456**	0.599	1.700	0.201	0.245	2.365	0.109	0.446	**0.015**	*0.089*	0.409
Startle 86 dB norm. to weight	2wANOVA	Nonnormal	9.86 ± 1.66	6 ± 0.49	7.03 ± 0.64	2.888	*0.0690*	0.529	0.030	0.864	0.053	0.268	0.766	0.089	**0.028**	*0.070*	0.677
Startle 92 dB norm. to weight	2wANOVA	Nonnormal	10.04 ± 1.77	7.09 ± 0.58	8.18 ± 1.15	1.758	0.1873	0.343	0.323	0.573	0.086	1.291	0.288	0.261	−	−	−
																	
Startle response normalized to weight	Test	Data structure				*F*	*p* value	Power	WT vs Het	WT vs KO	Het vs KO						
- Sound intensity	rMeasures	Sph.ass				2.506	**0.0449**	0.248	−	−	−						
- Sound intensity × gen.	rMeasures	Sph.ass				0.580	0.7933	0.098	−	−	−						
- Genotype	rMeasures	Sph.ass				3.338	**0.0471**	0.593	0.127	0.407	0.783						
- Cohort	rMeasures	Sph.ass				0.455	0.5046	0.101	−	−	−						
- Sound intensity × gen. × coh.	rMeasures	Sph.ass				0.716	0.5922	0.079	−	−	−						
- Gen. × coh.	rMeasures	Sph.ass				1.812	0.1783	0.353	−	−	−						
																	
**Pre-pulse inhibition (PPI)**																	
						Genotype			Cohort			Genotype × cohort			Pairwise comparisons		
	Test	Data structure	WT	Het	KO	*F*	*p* value	Power	*F*	*p* value	Power	*F*	*p* value	Power	WT vs Het	WT vs KO	Het vs KO
PPI % (74 dB)	2wANOVA	Normal	22.47 ± 5.7	15.1 ± 4.72	10.56 ± 6.03	0.625	0.5409	0.212	11.061	**0.002**	0.519	1.821	0.177	0.190	−	−	−
PPI % (78 dB)	2wANOVA	Normal	31.81 ± 4.48	14.84 ± 6.7	21.18 ± 5.03	3.513	**0.0407**	0.543	21.819	**0.000**	0.793	1.008	0.375	0.352	*0.058*	0.312	0.656
PPI % (82 dB)	2wANOVA	Normal	30.39 ± 5.51	21.53 ± 6.08	13.34 ± 5.99	0.151	0.8604	0.426	14.892	**0.000**	0.480	0.042	0.959	0.348	−	−	−

PPI % (86 dB)	2wANOVA	Nonnormal	31.73 ± 7.95	21.04 ± 7.37	27.04 ± 4.63	1.411	0.2575	0.176	37.139	**0.000**	0.909	0.463	0.633	0.217	−	−	−
PPI % (92 dB)	2wANOVA	Nonnormal	43.62 ± 7.01	34.35 ± 5.82	29.93 ± 6.97	0.989	0.3821	0.212	54.683	**0.000**	0.892	2.258	0.120	0.222	−	−	−
PPI % (average)	2wANOVA	Normal	32 ± 5.53	21.37 ± 5.48	20.41 ± 5.03	0.155	0.8569	0.305	37.489	**0.000**	0.867	0.310	0.735	0.278	−	−	−
																	
PPI	Test	Data structure				*F*	*p* value	Power	WT vs Het	WT vs KO	Het vs KO						
- Sound intensity	rMeasures	Sph.viol				15.396	**0.0000**	1.000	−	−	−						
- Sound intensity × gen.	rMeasures	Sph.viol				1.409	0.1943	0.603	−	−	−						
- Genotype	rMeasures	Sph.viol				1.502	0.2325	0.305	−	−	−						
- Cohort	rMeasures	Sph.viol				9.806	**0.0029**	0.867	−	−	−						
- Sound intensity × gen. × coh.	rMeasures	Sph.viol				0.978	0.4540	0.427	−	−	−						
- Gen. × coh.	rMeasures	Sph.viol				1.349	0.2686	0.278	−	−	−						
																	
**Burried food test**																	
						Genotype			Cohort			Genotype × cohort			Pairwise comparisons		
	Test	Data structure	WT	Het	KO	*F*	*p* value	Power	*F*	*p* value	Power	*F*	*p* value	Power	WT vs Het	WT vs KO	Het vs KO
Latency to retrieve and eat food (s)	2wANOVA	Nonnormal	51.11 ± 9.9	94.81 ± 23.69	500.28 ± 94.92	17.848	**0.0000**	1.000	0.001	0.976	0.050	0.000	1.000	0.050	0.858	**0.000**	**0.000**
																	
**Olfactory habituation/dishabituation, sniffing only**																	
Water	Test	Data structure				*F*	*p* value	Power	WT vs Het	WT vs KO	Het vs KO						
- Trial	rMeasures	Sph.viol				8.290	**0.0019**	0.958	−	−	−						
- Trial × gen.	rMeasures	Sph.viol				1.108	0.3505	0.337	−	−	−						
- Genotype	rMeasures	Sph.viol				2.973	*0.0602*	0.553	0.660	**0.027**	*0.066*						
- Cohort	rMeasures	Sph.viol				0.073	0.7886	0.058	−	−	−						
- Trial × gen. × coh.	rMeasures	Sph.viol				1.739	0.1683	0.515	−	−	−						
- Gen. × coh.	rMeasures	Sph.viol				1.015	0.3696	0.217	−	−	−						
																	
Banana	Test	Data structure				*F*	*p* value	Power	WT vs Het	WT vs KO	Het vs KO						
- Trial	rMeasures	Sph.ass				10.117	**0.0001**	0.983	−	−	−						
- Trial × gen.	rMeasures	Sph.ass				3.908	**0.0054**	0.888	−	−	−						
- Genotype	rMeasures	Sph.ass				5.681	**0.0060**	0.842	0.433	**0.002**	**0.017**						
- Cohort	rMeasures	Sph.ass				11.933	**0.0011**	0.923	−	−	−						
- Trial × gen. × coh.	rMeasures	Sph.ass				0.486	0.7461	0.161	−	−	−						
- Gen. × coh.	rMeasures	Sph.ass				0.134	0.8752	0.069	−	−	−						
																	
Lemon	Test	Data structure				*F*	*p* value	Power	WT vs Het	WT vs KO	Het vs KO						
- Trial	rMeasures	Sph.viol				6.699	**0.0041**	0.908	−	−	−						
- Trial × gen.	rMeasures	Sph.viol				0.047	0.9890	0.059	−	−	−						
- Genotype	rMeasures	Sph.viol				2.715	*0.0760*	0.513	0.404	0.166	**0.025**						
- Cohort	rMeasures	Sph.viol				15.327	**0.0003**	0.970	−	−	−						
- Trial × gen. × coh.	rMeasures	Sph.viol				0.667	0.5828	0.211	−	−	−						
- Gen. × coh.	rMeasures	Sph.viol				0.159	0.8534	0.073	−	−	−						
																	
Male scent	Test	Data structure				*F*	*p*value	Power	WT vs Het	WT vs KO	Het vs KO						
- Trial	rMeasures	Sph.viol				27.903	**0.0000**	1.000	−	−	−						
- Trial × gen.	rMeasures	Sph.viol				1.089	0.3581	0.332	−	−	−						
- Genotype	rMeasures	Sph.viol				0.739	0.4828	0.168	−	−	−						
- Cohort	rMeasures	Sph.viol				2.500	0.1201	0.341	−	−	−						
- Trial × gen. × coh.	rMeasures	Sph.viol				1.603	0.1976	0.479	−	−	−						
- Gen. × coh.	rMeasures	Sph.viol				0.541	0.5857	0.134	−	−	−						
																	
Female scent	Test	Data structure				*F*	*p* value	Power	WT vs Het	WT vs KO	Het vs KO						
- Trial	rMeasures	Sph.viol				20.922	**0.0000**	1.000	−	−	−						
- Trial × gen.	rMeasures	Sph.viol				0.131	0.9321	0.076	−	−	−						
- Genotype	rMeasures	Sph.viol				0.585	0.5609	0.142	−	−	−						
- Cohort	rMeasures	Sph.viol				14.771	**0.0003**	0.965	−	−	−						
- Trial × gen. × coh.	rMeasures	Sph.viol				0.348	0.7765	0.126	−	−	−						
- Gen. × coh.	rMeasures	Sph.viol				1.651	0.2022	0.332	−	−	−						
																	
						Genotype			Cohort			Genotype × cohort			Pairwisecomparisons		
Individual trials	Test	Data structure	WT	Het	KO	*F*	*p* value	Power	*F*	*p* value	Power	*F*	*p* value	Power	WT vs Het	WT vs KO	Het vs KO
Water 1	2wANOVA	Nonnormal	1.82 ± 0.37	1.81 ± 0.42	0.9 ± 0.16	2.205	0.1208	0.429	0.199	0.658	0.072	1.697	0.194	0.340	−	−	−
Water 2	2wANOVA	Nonnormal	1.02 ± 0.15	0.98 ± 0.14	0.7 ± 0.15	1.438	0.2470	0.294	0.078	0.781	0.059	0.711	0.496	0.163	−	−	−
Water 3	2wANOVA	Nonnormal	0.97 ± 0.15	0.82 ± 0.14	0.7 ± 0.11	1.724	0.1888	0.345	2.900	*0.095*	0.386	0.366	0.696	0.106	−	−	−
Banana 1	2wANOVA	Nonnormal	1.34 ± 0.12	1.04 ± 0.23	0.44 ± 0.15	8.742	**0.0006**	0.961	6.201	**0.016**	0.685	0.466	0.630	0.122	0.322	**0.001**	0.052
Banana 2	2wANOVA	Nonnormal	0.65 ± 0.12	0.65 ± 0.1	0.44 ± 0.13	1.641	0.2040	0.330	6.658	**0.013**	0.716	0.240	0.787	0.086	−	−	−
Banana 3	2wANOVA	Nonnormal	0.52 ± 0.09	0.72 ± 0.19	0.44 ± 0.13	1.167	0.3196	0.245	7.761	**0.008**	0.780	0.050	0.951	0.057	−	−	−

Lemon 1	2wANOVA	Nonnormal	0.58 ± 0.18	0.77 ± 0.15	0.52 ± 0.17	0.720	0.4916	0.165	8.572	**0.005**	0.819	0.041	0.960	0.056	−	−	−
Lemon 2	2wANOVA	Nonnormal	0.39 ± 0.11	0.52 ± 0.1	0.24 ± 0.07	2.679	*0.0785*	0.507	9.784	**0.003**	0.866	0.037	0.964	0.055	0.709	0.411	*0.099*
Lemon 3	2wANOVA	Nonnormal	0.35 ± 0.06	0.48 ± 0.09	0.25 ± 0.08	2.547	*0.0885*	0.486	7.405	**0.009**	0.761	2.493	*0.093*	0.478	0.594	0.540	0.106
Male 1	2wANOVA	Nonnormal	6.1 ± 1.12	4.66 ± 0.88	4.94 ± 1.07	0.659	0.5219	0.154	1.095	0.300	0.177	1.091	0.344	0.231	−	−	−
Male 2	2wANOVA	Nonnormal	2.25 ± 0.34	2.77 ± 0.46	1.26 ± 0.31	4.095	**0.0225**	0.700	0.564	0.456	0.114	3.078	*0.055*	0.568	0.752	0.116	**0.020**
Male 3	2wANOVA	Nonnormal	1.54 ± 0.38	1.25 ± 0.24	1.72 ± 0.63	0.322	0.7264	0.098	2.465	0.123	0.337	0.312	0.733	0.097	−	−	−
Female 1	2wANOVA	Nonnormal	5.68 ± 0.98	6.43 ± 1.34	6.32 ± 1.93	0.129	0.8790	0.069	11.509	**0.001**	0.914	0.879	0.422	0.193	−	−	−
Female 2	2wANOVA	Nonnormal	2.18 ± 0.3	2.24 ± 0.43	2.62 ± 0.81	0.189	0.8283	0.078	1.936	0.170	0.276	0.289	0.751	0.093	−	−	−
Female 3	2wANOVA	Nonnormal	1.42 ± 0.26	1.82 ± 0.52	2.76 ± 0.88	1.600	0.2120	0.323	7.672	**0.008**	0.775	1.792	0.177	0.357	−	−	−
																	
**Olfactory habituation/dishabituation, all interactions**																	
Water	Test	Data structure				*F*	*p* value	Power	WT vs Het	WT vs KO	Het vs KO						
- Trial	rMeasures	Sph.viol				1.891	0.1664	0.385	−	−	−						
- Trial × gen.	rMeasures	Sph.viol				0.718	0.5478	0.225	−	−	−						
- Genotype	rMeasures	Sph.viol				7.210	**0.0017**	0.920	0.637	**0.001**	**0.015**						
- Cohort	rMeasures	Sph.viol				2.639	0.1104	0.357	−	−	−						
- Trial × gen. × coh.	rMeasures	Sph.viol				0.920	0.4371	0.283	−	−	−						
- Gen. × coh.	rMeasures	Sph.viol				1.133	0.3300	0.239	−	−	−						
																	
Banana	Test	Data structure				*F*	*p* value	Power	WT vs Het	WT vs KO	Het vs KO						
- Trial	rMeasures	Sph.viol				5.229	**0.0133**	0.821	−	−	−						
- Trial × gen.	rMeasures	Sph.viol				1.282	0.2866	0.388	−	−	−						
- Genotype	rMeasures	Sph.viol				5.737	**0.0057**	0.846	0.744	0.060	**0.008**						
- Cohort	rMeasures	Sph.viol				7.922	**0.0070**	0.788	−	−	−						
- Trial × gen. × coh.	rMeasures	Sph.viol				0.936	0.4284	0.287	−	−	−						
- Gen. × coh.	rMeasures	Sph.viol				0.990	0.3787	0.213	−	−	−						
																	
Lemon	Test	Data structure				*F*	*p* value	Power	WT vs Het	WT vs KO	Het vs KO						
- Trial	rMeasures	Sph.viol				1.303	0.2728	0.276	−	−	−						
- Trial × gen.	rMeasures	Sph.viol				0.703	0.5597	0.221	−	−	−						
- Genotype	rMeasures	Sph.viol				4.893	**0.0115**	0.781	0.295	**0.048**	**0.003**						
- Cohort	rMeasures	Sph.viol				5.152	**0.0276**	0.605	−	−	−						
- Trial × gen. × coh.	rMeasures	Sph.viol				0.527	0.6738	0.172	−	−	−						
- Gen. × coh.	rMeasures	Sph.viol				2.405	0.1006	0.463	−	−	−						
																	
Male scent	Test	Data structure				*F*	*p* value	Power	WT vs Het	WT vs KO	Het vs KO						
- Trial	rMeasures	Sph.viol				28.652	**0.0000**	1.000	−	−	−						
- Trial × gen.	rMeasures	Sph.viol				0.790	0.4993	0.246	−	−	−						
- Genotype	rMeasures	Sph.viol				5.722	**0.0057**	0.845	0.839	**0.005**	**0.022**						
- Cohort	rMeasures	Sph.viol				2.953	*0.0918*	0.392	−	−	−						
- Trial × gen. × coh.	rMeasures	Sph.viol				0.367	0.7697	0.131	−	−	−						
- Gen. × coh.	rMeasures	Sph.viol				0.009	0.9906	0.051	−	−	−						
																	
Female scent	Test	Data structure				*F*	*p* value	Power	WT vs Het	WT vs KO	Het vs KO						
- Trial	rMeasures	Sph.ass				25.044	**0.0000**	1.000	−	−	−						
- Trial × gen.	rMeasures	Sph.ass				0.884	0.4762	0.264	−	−	−						
- Genotype	rMeasures	Sph.ass				1.119	0.3346	0.236	−	−	−						
- Cohort	rMeasures	Sph.ass				6.400	**0.0145**	0.699	−	−	−						
- Trial × gen. × coh.	rMeasures	Sph.ass				1.911	0.1143	0.541	−	−	−						
- Gen. × coh.	rMeasures	Sph.ass				0.431	0.6521	0.116	−	−	−						
																	
						Genotype			Cohort			Genotype × cohort			Pairwise comparisons		
Individual trials	Test	Data structure	WT	Het	KO	*F*	*p* value	Power	*F*	*p* value	Power	*F*	*p* value	Power	WT vs Het	WT vs KO	Het vs KO
Water 1	2wANOVA	Nonnormal	3.52 ± 0.9	3.88 ± 1	1.35 ± 0.27	2.357	0.1053	0.455	3.302	*0.075*	0.429	1.116	0.336	0.235	−	−	−
Water 2	2wANOVA	Nonnormal	3.45 ± 0.71	1.78 ± 0.48	0.95 ± 0.23	4.964	**0.0109**	0.786	0.588	0.447	0.117	1.295	0.283	0.267	0.161	**0.006**	0.364
Water 3	2wANOVA	Nonnormal	2.53 ± 0.54	2.3 ± 0.6	0.95 ± 0.2	2.997	*0.0592*	0.556	0.033	0.856	0.054	0.373	0.690	0.107	0.949	*0.078*	0.147
Banana 1	2wANOVA	Nonnormal	2.8 ± 0.42	3.38 ± 1.16	0.72 ± 0.43	3.854	**0.0279**	0.672	6.603	**0.013**	0.712	1.080	0.348	0.229	−	−	−
Banana 2	2wANOVA	Nonnormal	1.46 ± 0.49	1.06 ± 0.35	0.57 ± 0.18	1.902	0.1601	0.376	4.991	**0.030**	0.591	1.056	0.355	0.224	−	−	−
Banana 3	2wANOVA	Nonnormal	1.38 ± 0.55	2 ± 0.63	0.4 ± 0.1	3.927	**0.0262**	0.680	1.920	0.172	0.274	1.097	0.342	0.232	0.334	0.380	**0.020**
Lemon 1	2wANOVA	Nonnormal	1.47 ± 0.37	2.21 ± 0.74	0.45 ± 0.19	3.707	**0.0317**	0.653	2.710	0.106	0.365	0.878	0.422	0.193	0.412	0.339	**0.024**
Lemon 2	2wANOVA	Nonnormal	1.21 ± 0.35	0.98 ± 0.36	0.28 ± 0.09	2.536	*0.0895*	0.484	3.609	*0.063*	0.461	1.012	0.371	0.216	0.781	*0.091*	0.315
Lemon 3	2wANOVA	Nonnormal	0.8 ± 0.18	0.61 ± 0.15	0.2 ± 0.08	1.362	0.2656	0.280	1.615	0.210	0.238	1.334	0.273	0.275	−	−	−
Male 1	2wANOVA	Nonnormal	55.55 ± 6.73	48.98 ± 7.22	27.15 ± 6.11	3.341	**0.0436**	0.605	2.419	0.126	0.332	0.054	0.948	0.058	0.655	**0.030**	0.199
Male 2	2wANOVA	Nonnormal	27.09 ± 6.01	29.19 ± 5.33	7.48 ± 4.34	4.753	**0.0130**	0.768	1.196	0.280	0.189	0.012	0.988	0.052	0.986	**0.020**	**0.030**
Male 3	2wANOVA	Nonnormal	17.57 ± 5.5	14.39 ± 4.43	5.26 ± 1.63	2.323	0.1087	0.449	1.329	0.255	0.204	0.978	0.383	0.210	−	−	−
Female 1	2wANOVA	Nonnormal	53.65 ± 5.97	50.59 ± 5.48	31.16 ± 7.21	1.781	0.1792	0.355	6.118	**0.017**	0.679	1.565	0.219	0.316	−	−	−
Female 2	2wANOVA	Nonnormal	23.26 ± 6.37	18.85 ± 6.77	14.61 ± 4.3	0.427	0.6551	0.115	2.116	0.152	0.297	0.350	0.707	0.103	−	−	−
Female 3	2wANOVA	Nonnormal	14.57 ± 3.85	11.5 ± 3.69	14.21 ± 4.61	0.269	0.7650	0.090	0.575	0.452	0.115	1.703	0.193	0.341	−	−	−

WT, wild-type mice; Het, heterozygous mice; KO, homozygous knock-out mice. Group values are reported as mean ± SEM. Bold font indicates significant results (*p* < 0.05). Individual results and statistical analyses for cohorts 1 and 2 are available in Extended Data Table 7-1. 2wANOVA: 2-way ANOVA, rMeasures: repeated measures, Sph.ass: sphericity assumed, Sph.viol: sphericity violated, gen: genotype, coh: cohort.

10.1523/ENEURO.0046-18.2018.t7-1 Extended Data Table 7-1 **Individual results and statistical analyses for cohorts 1 and 2 related to the sensory profile.** WT, wild-type mice; Het, heterozygous mice; KO, homozygous knockout mice. Group values are reported as means ± s.e.m. Red font indicates significant results (p < 0.05), orange font indicates trends (0.1 < p < 0.05). Download Table 7-1, DOCX file.

**Figure 4.**
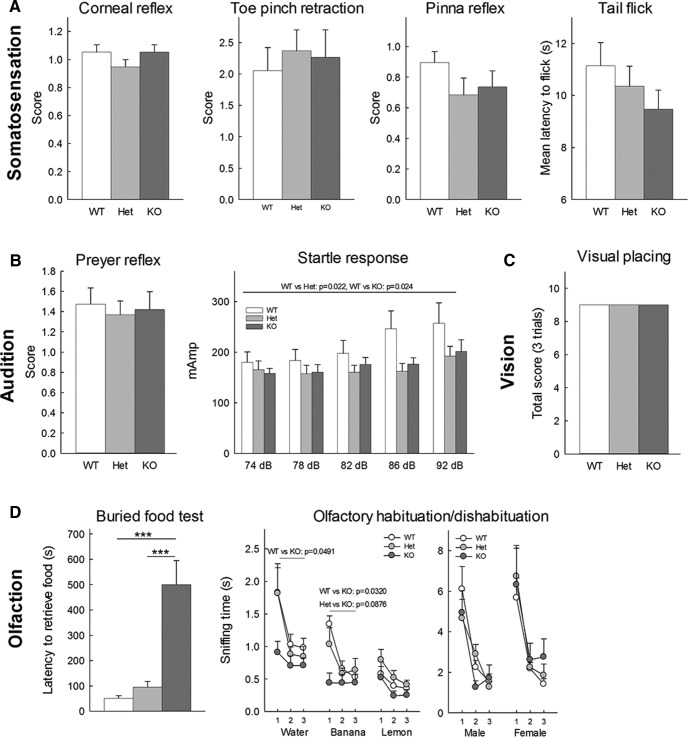
Altered sensory profile in *Shank3^Δ4-22^*-deficient mice. ***A***, Somatosensation evaluated with corneal reflex, toe pinch retraction, pinna reflex, and tail flick. Normal tactile and pain responses were observed in *Shank3^Δ4-22^-*deficient mice. ***B***, Auditory functions measured with the Preyer reflex and startle response to increasing sound intensities. No genotype difference was observed for Preyer reflex, however startle response was decreased in both heterozygous and homozygous *Shank3^Δ4-22^* mice compared to their wild-type littermate with genotype differences being more marked for the higher startle intensities. Pre-pulse inhibition results are displayed in Extended Data Figure 4-1*A*. ***C***, Gross visual function assessed by the visual placing test. Normal visual placing was observed for all genotypes. ***D***, Olfactory abilities evaluated by the time to find hidden food in buried food test and the cumulative time sniffing the applicator without direct interactions during olfactory habituation and dishabituation to nonsocial and social odors. Strong impairments were observed in the buried food test for *Shank3^Δ4-22^* homozygous mice as shown by a significant increase in the latency to retrieve the buried food, compared to their heterozygous and wild-type littermates. Individual performances are available in Extended Data Figure 4-1*B*. Similarly, a significant lack of interest for nonsocial scents (water, banana, and lemon) was observed in *Shank3^Δ4-22^*homozygous mice but not in heterozygotes and wild-type during olfactory habituation/dishabituation, while they still displayed normal habituation/dishabituation for social scents (unfamiliar male and female bedding). The olfactory habituation and dishabituation to nonsocial and social odors was measured as cumulative time spent sniffing a sequence of identical and novel odors delivered on cotton swabs inserted into a clean cage. WT, wild-type mice; Het, heterozygous mice; KO, homozygous knock-out mice. *p < 0.05, **p < 0.1, ***p < 0.001.

No genotype differences were detected in tactile tests including the pinna reflex, the palpebral reflex, and the toe pinch retraction test. In the tail flick pain sensitivity test, a trend toward a decreased latency to flick the tail in response to a noxious thermal stimulation a was observed in *Shank3^Δ4-22^* homozygous animals (Fig. 4*A*).

10.1523/ENEURO.0046-18.2018.f4-1 Extended Data Figure 4-1 **Altered sensory profile in Shank3 complete knockout mice.** (A) Pre-pulse inhibition. A non-significant decrease of pre-pulse inhibition was observed in both heterozygous and homozygous Shank3^Δ4-22^ mice compared to wild-type animals. (B)Individual scores in buried food test showing that 50% of Shank3^Δ4-22^ homozygous mice fail to retrieve buried food (cut-off of 900 seconds). WT, wild-type mice; Het, heterozygous mice; KO, homozygous knockout mice. Download Figure 4-1, TIF file.

Normal Preyer reflexes were observed in all genotypes; however, *Shank3^Δ4-22^* heterozygous and homozygous mice showed a reduced startle response throughout all the sound intensities (74–92 dB, analyzed as repeated measures) indicating an impaired sound discrimination (Fig. 4*B*). Changes in pre-pulse inhibition of acoustic startle in *Shank3^Δ4-22^-*deficient mice are consistent with abnormalities in auditory processing, rather than sensorimotor gating deficits (Extended Data Fig. 4-1*A*).

Normal visual placing/reaching reflexes were observed for all the mice, thus ruling out strong visual impairments (Fig. 4*C*).

*Shank3^Δ4-22^* homozygous mice demonstrated strong deficits in the buried food test (Fig. 4*D*, left panel) with only seven out of 19 mice able to retrieve the food in less than 2 min and nine out of 19 mice not being able to find the food at all (Extended Data Fig. 4-1*B*). However, all animals showed interest for the food and ate it when it was made visible. To further investigate olfactory function, animals were subjected to the olfactory habituation/dishabituation paradigm using three nonsocial scents (water, banana, and lemon) and two social scents (unfamiliar males and unfamiliar females). Wild-type and *Shank3^Δ4-22^* heterozygous animals displayed a normal response, characterized by a robust sniffing elicited by the first scent presentation of each nonsocial and social scent that declined over the second and third presentation of the same scent. In contrast, *Shank3^Δ4-22^* homozygous animals had little response to any of the nonsocial scents, even on their first presentation (Fig. 4*D*, middle panel), thus confirming the results of the buried food test. Interestingly the lack of interest for olfactory stimuli does not appear to be the consequence of anosmia as a normal response to both social scents was observed in *Shank3^Δ4-22^* homozygous mice (Fig. 4*D*, right panel).

### Social interactions in *Shank3^Δ4-22^*-deficient mice

**Figure 5.**
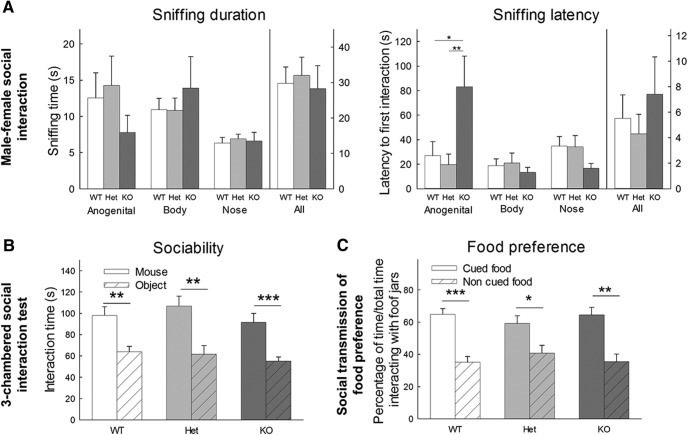
Social behavior of *Shank3^Δ4-22^-*deficient mice. ***A***, Male social interaction in response to the presentation of an unfamiliar conspecific female in estrus and scored by the cumulative sniffing time and latency from the male toward different body regions of the female. No genotype differences were evident in the dyadic male-female social interaction for the overall sniffing time from the male toward the female, however a trend toward a decrease in anogenital sniffing as well as a significant increase of the latency to initiate the first anogenital sniffing event was observed in *Shank3^Δ4-22^* homozygous mice. ***B***, Preference for social stimulus in the three-chambered social interaction test measured by cumulative time interacting with either a mouse or an inanimate object. All three genotypes demonstrated a significant preference for an unfamiliar mouse over a nonsocial object. ***C***, Social transmission of food preference measured by the time spent by the test mouse sniffing the demonstrator mouse and the time spent interacting with both cued and noncued food. All genotypes had a strong preference for the food flavor presented by the demonstrator mouse. USVs and time spent sniffing the demonstrator during the demonstrator interaction phase are displayed in Extended Data Figure 5-1. WT, wild-type mice; Het, heterozygous mice; KO, homozygous knock-out mice. *p < 0.05, **p < 0.1, ***p < 0.001.

Mice were evaluated for social abilities during male-female dyadic social interaction, in the three-chambered social interaction task, and in the social transmission of food preference test and detailed results are reported in Table 8. In freely moving male-female dyads of male mice paired with unfamiliar wild-type estrous C57BL6 females, sniffing time was generally similar across genotypes (Fig. 5*A*, left panel). A significant increase in latency for the first event of anogenital sniffing was found in male *Shank3^Δ4-22^* homozygous mice (Fig. 5*A*, right panel), and we can note that this latency may contribute to trend toward reduced anogenital sniffing time in those animals. Ultrasonic vocalizations did not show significant difference across genotypes (Extended Data Fig. 5-1*A*).

10.1523/ENEURO.0046-18.2018.f5-1 Extended Data Figure 5-1 **Social interactions in ShankΔ4-22-deficient mice.** (A) Male female social interaction. A non-significant decrease of the number of ultrasonic vocalization was observed in males Shank3^Δ4-22^ homozygous mice upon exposure to an estrus female. (B)Social transmission of food preference. A trend toward a reduction of sniffing during the demonstrator interaction phase was observed in in Shank3^Δ4-22^ homozygous mice. Download Figure 5-1, TIF file.

**Table 8.** Detailed results and statistical analyses related to social behavior

**Three chambered social interaction test - social preference**																	
Zone comparison, three zones, time in chambers																	
All mice	Test	Data structure				*F*	*p* value	Power	C vs M	C vs O	M vs O						
- Chamber	rMeasures	Sph.viol				149.525	**0.0000**	1.000	**0.000**	**0.000**	**0.000**						
- Cohort	rMeasures	Sph.viol				1.456	0.2328	0.452	−	−	−						
- Chamber × cohort	rMeasures	Sph.viol				2.267	0.1149	0.220	−	−	−						
																	
WT	Test	Data structure				*F*	*p* value	Power	WT vs Het	WT vs KO	Het vs KO						
- Chamber	rMeasures	Sph.ass				78.786	**0.0000**	1.000	**0.000**	**0.001**	**0.000**						
- Cohort	rMeasures	Sph.ass				5.360	**0.0342**	0.585	−	−	−						
- Chamber × cohort	rMeasures	Sph.ass				1.546	0.2285	0.297	−	−	−						
																	
Het	Test	Data structure				*F*	*p* value	Power	WT vs Het	WT vs KO	Het vs KO						
- Chamber	rMeasures	Sph.viol				61.909	**0.0000**	1.000	**0.000**	**0.001**	**0.000**						
- Cohort	rMeasures	Sph.viol				1.252	0.2787	0.184	−	−	−						
- Chamber × cohort	rMeasures	Sph.viol				3.768	*0.0508*	0.543	−	−	−						
																	
KO	Test	Data structure				*F*	*p* value	Power	WT vs Het	WT vs KO	Het vs KO						
- Chamber	rMeasures	Sph.ass				30.043	**0.0000**	1.000	**0.000**	**0.002**	**0.000**						
- Cohort	rMeasures	Sph.ass				2.003	0.1751	0.267	−	−	−						
- Chamber × cohort	rMeasures	Sph.ass				0.227	0.7982	0.082	−	−	−						
																	
Zone comparison, two zones, Mouse A vs object interaction time																	
	Test	Data structure				All *F*	All *p* value	Power	WT *F*	WT *p* value	Power	Het *F*	Het *p* value	Power	KO *F*	KO *p* value	Power
- Chamber	rMeasures	Sph.ass				40.069	**0.0000**	1.000	10.622	**0.005**	0.864	14.120	**0.002**	0.943	19.123	**0.000**	0.984
- Cohort	rMeasures	Sph.ass				1.078	0.3038	0.175	0.561	0.465	0.109	3.631	0.769	0.059	0.434	0.519	0.095
- Chamber × cohort	rMeasures	Sph.ass				0.921	0.3414	0.156	0.002	0.963	0.050	0.089	*0.074*	0.436	0.617	0.443	0.115
																	
						Genotype			Cohort			Genotype × cohort			Pairwise comparisons		
	Test	Data structure	WT	Het	KO	*F*	*p* value	Power	*F*	*p* value	Power	*F*	*p* value	Power	WT vs Het	WT vs KO	Het vs KO
Time in mouse or object chamber	2wANOVA	Nonnormal	528.42 ± 10.99	509.46 ± 27.36	501.19 ± 14.27	0.428	0.6540	0.116	2.557	0.116	0.348	0.131	0.878	0.069	−	−	−
Time sniffing mouse or object	2wANOVA	Normal	89.02 ± 6.62	103.43 ± 10.43	101.27 ± 11.18	0.670	0.5160	0.156	2.367	0.130	0.326	1.153	0.324	0.242	−	−	−
Time close to mouse or object	2wANOVA	Normal	162.11 ± 8.5	168.73 ± 11.02	146.49 ± 10.45	1.165	0.3200	0.244	0.888	0.351	0.152	0.037	0.964	0.055	−	−	−
																	
**Male-female social interactions, sniffing**																	
						Genotype			Cohort			Genotype × cohort			Pairwise comparisons		
	Test	Data structure	WT	Het	KO	*F*	*p* value	Power	*F*	*p* value	Power	*F*	*p* value	Power	WT vs Het	WT vs KO	Het vs KO
Anogenital, time (s)	2wANOVA	Nonnormal	10.22 ± 1.62	12.36 ± 1.86	9.05 ± 1.43	0.933	0.4000	0.202	0.154	0.696	0.067	0.097	0.908	0.064	−	−	−
Anogenital, number	2wANOVA	Nonnormal	12.56 ± 3.44	14.28 ± 4.04	7.77 ± 2.36	1.049	0.3580	0.223	0.050	0.824	0.056	0.653	0.525	0.153	−	−	−
Anogenital, latency to first (s)	2wANOVA	Nonnormal	26.91 ± 11.44	19.61 ± 8.63	83.18 ± 25.05	4.238	**0.0200**	0.715	2.222	0.143	0.309	1.172	0.318	0.245	0.619	**0.032**	**0.008**
Nose to body, time (s)	2wANOVA	Nonnormal	14.94 ± 2.05	14.94 ± 2.71	16.58 ± 2.86	0.348	0.7080	0.103	0.783	0.381	0.140	1.722	0.190	0.344	−	−	−
Nose to body, number	2wANOVA	Nonnormal	10.93 ± 1.54	10.83 ± 1.68	13.91 ± 4.35	0.333	0.7190	0.100	0.483	0.490	0.105	2.192	0.123	0.426	−	−	−
Nose to body, latency to first (s)	2wANOVA	Nonnormal	18.89 ± 5.38	20.94 ± 8.22	13.38 ± 3.97	0.332	0.7190	0.100	1.025	0.316	0.168	0.915	0.408	0.199	−	−	−
Nose to nose, time (s)	2wANOVA	Nonnormal	8.55 ± 0.85	10.73 ± 1.07	9.58 ± 1.13	0.133	0.8760	0.069	0.717	0.401	0.132	1.107	0.339	0.233	−	−	−
Nose to nose number	2wANOVA	Nonnormal	6.31 ± 0.79	6.91 ± 0.63	6.61 ± 1.18	1.118	0.3350	0.235	0.020	0.889	0.052	0.394	0.676	0.110	−	−	−
Nose to nose, latency to first (s)	2wANOVA	Nonnormal	34.72 ± 7.85	34.09 ± 9.24	16.58 ± 3.97	1.599	0.2130	0.322	0.062	0.804	0.057	0.417	0.661	0.114	−	−	−
All sniffing, time (s)	2wANOVA	Nonnormal	33.77 ± 3.7	38.05 ± 4.77	35.23 ± 4.63	0.155	0.8570	0.073	0.686	0.412	0.128	0.654	0.524	0.153	−	−	−
All sniffing, number	2wANOVA	Nonnormal	29.81 ± 4.59	32.03 ± 5.13	28.29 ± 6.45	0.313	0.7330	0.097	0.138	0.712	0.065	1.522	0.229	0.308	−	−	−
All sniffing, latency to first (s)	2wANOVA	Nonnormal	5.52 ± 1.85	4.29 ± 1.55	7.41 ± 2.92	0.586	0.5610	0.142	1.267	0.266	0.197	0.042	0.959	0.056	−	−	−

**Male-female social interactions, ultrasonic vocalization**																	
						Genotype			Cohort			Genotype × cohort			Pairwise comparisons		
	Test	Data structure	WT	Het	KO	*F*	*p* value	Power	*F*	*p* value	Power	*F*	*p* value	Power	WT vs Het	WT vs KO	Het vs KO
USV, all calls	2wANOVA	Nonnormal	380.11 ± 50.36	378.33 ± 64.78	287.58 ± 31.87	1.345	0.2704	0.276	3.242	*0.078*	*0.422*	0.193	0.825	0.078	−	−	−
USV, minute 1	2wANOVA	Nonnormal	84.16 ± 12.35	94.11 ± 21.67	64.7 ± 8.21	1.071	0.3507	0.227	1.180	0.283	0.186	0.865	0.428	0.190	−	−	−
USV, minute 2	2wANOVA	Nonnormal	68.11 ± 9.01	73.22 ± 14.22	57.41 ± 5.55	0.649	0.5271	0.152	1.150	0.289	0.183	0.070	0.932	0.060	−	−	−
USV, minute 3	2wANOVA	Nonnormal	77.61 ± 11.62	68.72 ± 8.59	57.7 ± 8.2	1.363	0.2659	0.279	2.155	0.149	0.301	0.799	0.456	0.178	−	−	−
USV, minute 4	2wANOVA	Nonnormal	74.5 ± 14.48	76.44 ± 13	52.7 ± 4.98	1.566	0.2197	0.316	4.139	**0.048**	**0.513**	0.276	0.760	0.091	−	−	−
USV, minute 5	2wANOVA	Nonnormal	75.72 ± 14.21	65.83 ± 11.73	55.05 ± 8.13	1.092	0.3439	0.230	5.269	**0.026**	**0.613**	0.049	0.952	0.057	−	−	−
																	
USV	Test	Data structure				*F*	*p* value	Power	WT vs Het	WT vs KO	Het vs KO						
- Time	rMeasures	Sph.viol				2.964	*0.0210*	0.785	−	−	−						
- Time × genotype	rMeasures	Sph.viol				0.558	0.8110	0.254	−	−	−						
- Genotype	rMeasures	Sph.viol				1.345	0.2704	0.276	−	−	−						
- Cohort	rMeasures	Sph.viol				3.242	*0.0782*	0.422	−	−	−						
- Time × gen. × coh.	rMeasures	Sph.viol				1.245	0.2750	0.564	−	−	−						
- Gen. × coh.	rMeasures	Sph.viol				0.193	0.8248	0.078	−	−	−						

**Social transmission of food preference**																	
						Genotype			Cohort			Genotype × cohort			Pairwise comparisons		
	Test	Data structure	WT	Het	KO	*F*	*p* value	Power	*F*	*p* value	Power	*F*	*p* value	Power	WT vs Het	WT vs KO	Het vs KO
Demonstrator sniffing time (s)	2wANOVA	Nonnormal	29.8 ± 6.01	37.44 ± 6.25	24.69 ± 6.39	0.756	0.4752	0.171	4.407	**0.041**	**0.538**	0.202	0.818	0.080	−	−	−
Number of sniffing bouts	2wANOVA	Nonnormal	9.68 ± 1.51	13.57 ± 1.48	7.26 ± 1.36	4.064	**0.0236**	0.695	2.772	0.103	0.371	0.099	0.906	0.064	0.126	0.733	**0.021**
Time exploring all food (s)	2wANOVA	Nonnormal	1533.36 ± 98.47	1456.68 ± 98.14	1715.26 ± 124.97	1.372	0.2635	0.281	0.361	0.551	0.091	0.216	0.806	0.082	−	−	−
Time pre-exposed/all food (%)	2wANOVA	Nonnormal	64.89 ± 3.55	59.21 ± 4.8	64.54 ± 4.71	0.589	0.5589	0.142	0.150	0.700	0.067	1.790	0.178	0.356	−	−	−
Time new/all food (%)	2wANOVA	Nonnormal	35.1 ± 3.55	40.78 ± 4.8	35.45 ± 4.71	0.589	0.5589	0.142	0.150	0.700	0.067	1.790	0.178	0.356	−	−	−
Ratio time pre-exposed/new	2wANOVA	Nonnormal	2.6 ± 0.45	2.31 ± 0.47	3.06 ± 0.64	0.636	0.5338	0.150	1.077	0.305	0.174	0.739	0.483	0.168	−	−	−
Time spent exploring cocoa/all food (%)	2wANOVA	Nonnormal	51.21 ± 4.98	50.65 ± 5.26	50.97 ± 5.82	0.003	0.9969	0.050	0.240	0.626	0.077	0.856	0.431	0.188	−	−	−
Time exploring cinnamon/all food (%)	2wANOVA	Nonnormal	48.78 ± 4.98	49.34 ± 5.26	49.02 ± 5.82	0.003	0.9969	0.050	0.240	0.626	0.077	0.856	0.431	0.188	−	−	−
Ratio time cocoa/cinnamon	2wANOVA	Nonnormal	1.55 ± 0.31	1.67 ± 0.4	2.07 ± 0.64	0.585	0.5611	0.141	0.769	0.385	0.138	0.433	0.651	0.116	−	−	−
																	
Total amount of eaten food (g)	2wANOVA	Nonnormal	1.21 ± 0.18	0.82 ± 0.15	0.58 ± 0.06	4.286	**0.0195**	0.720	1.848	0.180	0.265	0.726	0.489	0.166	0.146	**0.011**	0.491
Amount of eaten food, pre-exposed (g)	2wANOVA	Nonnormal	0.87 ± 0.14	0.67 ± 0.14	0.46 ± 0.07	2.346	0.1068	0.452	2.802	0.101	0.375	1.058	0.355	0.224	−	−	−
Amount of eaten food, new (g)	2wANOVA	Nonnormal	0.34 ± 0.08	0.15 ± 0.04	0.13 ± 0.03	4.130	**0.0223**	0.703	0.063	0.803	0.057	1.108	0.339	0.233	*0.065*	**0.048**	0.990
Amount of eaten food, cocoa (g)	2wANOVA	Nonnormal	0.59 ± 0.13	0.36 ± 0.11	0.3 ± 0.07	1.887	0.1629	0.373	0.343	0.561	0.089	0.720	0.492	0.165	−	−	−
Amount of eaten food, cinnamon (g)	2wANOVA	Nonnormal	0.62 ± 0.13	0.46 ± 0.13	0.3 ± 0.05	1.563	0.2202	0.315	1.125	0.294	0.180	0.165	0.849	0.074	−	−	−
																	
Percentage pre-exposed vs new	Test	Data structure				All *F*	All *p* value	Power	WT *F*	WT *p* value	Power	Het *F*	Het *p* value	Power	KO *F*	KO *p* value	Power
- Flavor	rMeasures	Sph.ass				25.686	**0.0000**	0.999	18.792	**0.000**	0.983	4.601	**0.045**	0.433	9.230	**0.007**	0.817
- Cohort	rMeasures	Sph.ass				0.000	1.0000	0.050	0.800	0.384	NA	0.196	0.663	NA	0.531	0.476	0.106
- Flavor × cohort	rMeasures	Sph.ass				0.009	0.9227	0.051	6.593	**0.020**	0.678	0.195	0.665	0.070	1.137	0.301	0.172
																	
Time cacao vs cinnamon	Test	Data structure				All *F*	All *p* value	Power	WT *F*	WT *p* value	Power	Het *F*	Het *p* value	Power	KO *F*	KO *p* value	Power
- Flavor	rMeasures	Sph.ass				0.001	0.9745	0.050	0.058	0.812	0.050	0.035	0.854	0.054	0.058	0.812	0.063
- Cohort	rMeasures	Sph.ass				0.100	0.7525	0.061	0.080	0.780	0.135	0.196	0.663	0.070	0.080	0.780	0.058
- Flavor × cohort	rMeasures	Sph.ass				0.001	0.9702	0.050	0.004	0.950	0.178	0.957	0.342	0.152	0.004	0.950	0.050
																	
Amount of eaten food, pre-expose vs new	Test	Data structure				All *F*	All *p* value	Power	WT *F*	WT *p* value	Power	Het *F*	Het *p*value	Power	KO *F*	KO *p* value	Power
- Flavor	rMeasures	Sph.ass				42.099	**0.0000**	1.000	13.852	**0.002**	0.935	13.378	**0.002**	0.929	13.503	**0.002**	0.931
- Cohort	rMeasures	Sph.ass				2.399	0.1276	0.330	0.400	0.537	0.091	2.323	0.147	0.299	0.131	0.722	0.063
- Flavor × cohort	rMeasures	Sph.ass				3.445	*0.0692*	0.445	4.346	*0.055*	0.496	0.872	0.364	0.142	0.080	0.781	0.058
																	
Amount of eaten food, cacao vs cinnamon	Test	Data structure				All *F*	All *p* value	Power	WT *F*	WT *p* value	Power	Het *F*	Het *p* value	Power	KO *F*	KO *p* value	Power
- Flavor	rMeasures	Sph.ass				0.212	0.6473	0.074	0.005	0.945	0.050	0.265	0.614	0.077	0.011	0.918	0.051
- Cohort	rMeasures	Sph.ass				2.399	0.1276	0.330	0.400	0.537	0.091	2.323	0.147	0.299	0.131	0.722	0.063
- Flavor × cohort	rMeasures	Sph.ass				0.117	0.7342	0.063	0.178	0.679	0.068	0.052	0.822	0.055	0.271	0.610	0.078

WT, wild-type mice; Het, heterozygous mice; KO, homozygous knock-out mice. C, center chamber; M, mouse chamber; O, object chamber. Group values are reported as mean ± SEM. Bold font indicates significant results (*p* < 0.05). Individual results and statistical analyses for cohorts 1 and 2 are available in Extended Data Table 8-1. 2wANOVA: 2-way ANOVA, rMeasures: repeated measures, Sph.ass: sphericity assumed, Sph.viol: sphericity violated, gen: genotype, coh: cohort.

10.1523/ENEURO.0046-18.2018.t8-1 Extended Data Table 8-1 **Individual results and statistical analyses for cohorts 1 and 2 related to social behavior.** WT, wild-type mice; Het, heterozygous mice; KO, homozygous knockout mice. C, center chamber; M, mouse chamber; O, object chamber. Group values are reported as means ± s.e.m. Red font indicates significant results (p < 0.05), orange font indicates trends (0.1 < p < 0.05). Download Table 8-1, DOCX file.

Similarly, In the three-chambered test for social preference, sociability, defined as spending more time interacting with the mouse than with the object, was found in all genotypes. Hence, in all groups, significantly more time was spent in the chamber containing the novel mouse than in the chamber containing the novel object, and more time was spent sniffing the novel mouse than the novel object (Fig. 5*B*). All genotypes showed the normal absence of innate chamber side bias during the 10-min habituation phase before the start of the sociability test.

Finally, mice were tested in the social transmission of food preference test that combines social behavior, olfactory recognition and memory skills. A modest decrease of the number of sniffing bouts initiated by the observer mouse toward the demonstrator mouse was observed during the observer-demonstrator interaction phase in *Shank3^Δ4-22^* homozygous mice but not in heterozygotes (Extended Data Fig. 5-1*B*). All genotypes showed a strong preference for the cued food flavor that was exposed to them through the demonstrator, as compared to the noncued food flavor, as shown both by significantly more time spent interacting with the jar containing the cued food than the noncued food (Fig. 5*C*) or by eating significantly more cued food than noncued food during the choice phase (Table 8). Note that two flavors were randomly used as cued and noncued food flavor and all genotypes showed an absence of flavor preference. However, the total amount of food (cued and noncued) eaten by *Shank3^Δ4-22^* homozygous mice was significantly lower than the total amount of food eaten by their wild-type and homozygous littermates.

### Object avoidance in *Shank^Δ4-22^*-deficient mice

While testing mice in different set-ups involving object interactions, a strong avoidance toward inanimate objects was observed in *Shank3^Δ4-22^* homozygous mice (Table 9).

**Table 9.** Detailed results and statistical analyses related to the avoidance behavior

**Novel object habituation**																	
						Genotype			Cohort			Genotype × cohort			Pairwise comparisons		
	Test	Data structure	WT	Het	KO	*F*	*p* value	Power	*F*	*p* value	Power	*F*	*p* value	Power	WT vs Het	WT vs KO	Het vs KO
Total distance (cm)	2wANOVAA	Nonnormal	3616.84 ± 351.4	3111.83 ± 221.31	3118.64 ± 277.29	0.724	0.490	0.166	15.022	**0.000**	0.967	0.927	0.402	0.202	−	−	−
Time in left side (s)	2wANOVA	Normal	314.74 ± 20.15	306.42 ± 15.26	276.81 ± 23.06	1.079	0.348	0.229	0.100	0.753	0.061	2.014	0.144	0.397	−	−	−
Time in right side (s)	2wANOVA	Normal	284.74 ± 20.17	293.06 ± 15.23	322.76 ± 23.1	1.086	0.345	0.230	0.112	0.739	0.062	2.009	0.145	0.396	−	−	−
																	
Time spend in left vs right half side	Test	Data structure				All *F*	All *p* value	Power	WT *F*	WT *p* value	Power	Het *F*	Het *p* value	Power	KO *F*	KO *p* value	Power
- Side	rMeasures	Sph.ass				0.001	0.979	0.052	0.445	0.514	0.097	0.145	0.708	0.065	1.249	0.279	0.184
- Cohort	rMeasures	Sph.ass				12.824	**0.001**	0.937	3.326	*0.086*	0.406	10.401	**0.005**	0.860	3.035	0.100	0.376
- Side × cohort	rMeasures	Sph.ass				0.044	0.835	0.062	0.096	0.761	0.060	1.352	0.261	0.195	2.506	0.132	0.321
																	
**Novel object recognition: training with two identical objects**																	
						Genotype			Cohort			Genotype × cohort			Pairwise comparisons		
	Test	Data structure	WT	Het	KO	*F*	*p* value	Power	*F*	*p* value	Power	*F*	*p* value	Power	WT vsHet	WT vs KO	Het vs KO
Total distance (cm)	2wANOVA	Nonnormal	2421.86 ± 196.94	2175 ± 173.66	1631.78 ± 118.14	5.366	**0.008**	0.820	2.961	*0.091*	0.393	0.804	0.453	0.180	0.540	**0.004**	*0.059*
Time in left side (s)	2wANOVA	Normal	150.86 ± 9.32	148.91 ± 10.25	176.59 ± 16.36	1.489	0.235	0.303	0.040	0.843	0.054	0.905	0.411	0.198	−	−	−
Time in right side (s)	2wANOVA	Normal	148.86 ± 9.35	150.82 ± 10.23	122.95 ± 16.33	1.511	0.230	0.307	0.042	0.839	0.055	0.896	0.414	0.196	−	−	−
Number of side switches	2wANOVA	Normal	15.94 ± 1.28	14.26 ± 1	13.89 ± 1.21	0.822	0.445	0.183	0.259	0.613	0.079	0.459	0.635	0.121	−	−	−
Number of left object exploration	2wANOVA	Nonnormal	21.84 ± 2.83	19.52 ± 2.54	14.1 ± 1.67	2.641	*0.081*	0.502	0.335	0.565	0.088	1.071	0.350	0.227	0.777	*0.070*	0.261
Number of right object exploration	2wANOVA	Nonnormal	33.42 ± 7.21	28.84 ± 7.87	16.78 ± 2.65	1.558	0.220	0.316	2.295	0.136	0.318	0.443	0.645	0.118	−	−	−
Time exploring left object (s)	2wANOVA	Nonnormal	22.2 ± 3.15	19.89 ± 3.1	10.67 ± 2.11	4.527	**0.015**	0.747	0.536	0.467	0.111	0.993	0.377	0.214	0.834	**0.016**	*0.066*
Time exploring right object (s)	2wANOVA	Nonnormal	23.97 ± 3.28	23.94 ± 4.06	11.28 ± 2.37	5.322	**0.008**	0.817	2.582	0.114	0.351	0.277	0.759	0.092	1.000	**0.024**	**0.025**
Latency to left object (s)	2wANOVA	Nonnormal	15.89 ± 4.3	21.74 ± 7.62	20.67 ± 3.94	0.353	0.704	0.104	0.068	0.796	0.057	0.138	0.872	0.070	−	−	−
Latency to right object (s)	2wANOVA	Nonnormal	15.03 ± 3.6	13.99 ± 5.42	17.92 ± 3.01	0.348	0.708	0.103	3.325	*0.074*	0.432	0.816	0.448	0.182	−	−	−
Total number of object exploration	2wANOVA	Nonnormal	55.26 ± 9.69	48.36 ± 9.86	30.89 ± 4.09	1.992	0.147	0.393	0.959	0.332	0.161	0.596	0.555	0.144	−	−	−
Total time exploring objects (s)	2wANOVA	Nonnormal	46.18 ± 5.82	43.83 ± 6.59	21.95 ± 4.26	5.733	**0.006**	0.846	1.680	0.201	0.246	0.041	0.960	0.056	0.954	**0.011**	**0.024**
Latency to any object (s)	2wANOVA	Nonnormal	8.92 ± 3.11	13.43 ± 5.8	17.5 ± 3.36	0.674	0.514	0.157	1.455	0.233	0.220	0.482	0.621	0.125	−	−	−
																	
Sniffing time,left vs right (s)	Test	Data structure				All *F*	All *p* value	Power	WT *F*	WT *p* value	Power	Het *F*	Het *p* value	Power	KO *F*	KO *p* value	Power
- Side	rMeasures	Sph.ass				2.505	0.119	0.343	1.375	0.257	0.198	1.701	0.209	0.234	0.128	0.725	0.063
- Cohort	rMeasures	Sph.ass				0.943	0.336	0.159	0.245	0.627	0.075	0.607	0.447	0.114	1.226	0.284	0.182
- Side × cohort	rMeasures	Sph.ass				1.598	0.211	0.237	7.358	0.015	0.725	0.829	0.375	0.138	1.756	0.203	0.240
																	
Number of interactions, left vs right	Test	Data structure				All *F*	All *p* value	Power	WT *F*	WT *p* value	Power	Het *F*	Het *p* value	Power	KO *F*	KO *p* value	Power
- Side	rMeasures	Sph.ass				8.030	**0.006**	0.795	4.264	*0.055*	0.495	2.075	0.168	0.275	3.519	*0.078*	0.425
- Cohort	rMeasures	Sph.ass				1.277	0.263	0.199	1.149	0.299	0.173	0.375	0.548	0.089	0.311	0.585	0.082
- Side × cohort	rMeasures	Sph.ass				6.312	**0.015**	0.694	1.703	0.209	0.234	2.023	0.173	0.269	6.059	**0.025**	0.641
																	
Time in left vs right halves (s)	Test	Data structure				All *F*	All *p* value	Power	WT *F*	WT *p* value	Power	Het *F*	Het *p* value	Power	KO *F*	KO *p* value	Power
- Side	rMeasures	Sph.ass				1.555	0.218	0.232	0.000	0.994	0.050	0.013	0.909	0.051	2.506	0.132	0.321
- Cohort	rMeasures	Sph.ass				0.591	0.445	0.117	0.142	0.711	0.065	3.906	0.065	0.462	0.161	0.693	0.067
- Side × cohort	rMeasures	Sph.ass				0.110	0.742	0.062	0.371	0.551	0.089	0.224	0.642	0.073	0.856	0.368	0.141
																	
**Novel object recognition: test with one new object**																	
						Genotype			Cohort			Genotype × cohort			Pairwise comparisons		
	Test	Data structure	WT	Het	KO	*F*	*p* value	Power	*F*	*p* value	Power	*F*	*p* value	Power	WT vs Het	WT vs KO	Het vs KO
Total distance (cm)	2wANOVA	Normal	1973.56 ± 156.94	1482.1 ± 167.46	1057.83 ± 110.93	9.082	**0.000**	0.968	0.000	0.989	0.050	1.066	0.352	0.227	0.059	**0.000**	0.117
Time in new object side (s)	2wANOVA	Normal	151.59 ± 9.78	143.64 ± 15.18	174.14 ± 19.84	0.985	0.380	0.212	0.004	0.947	0.050	0.545	0.583	0.135	−	−	−
Time pre-exposed object side (s)	2wANOVA	Normal	147.79 ± 9.8	155.86 ± 15.26	125.22 ± 19.97	0.983	0.381	0.212	0.001	0.972	0.050	0.533	0.590	0.133	−	−	−
Number of side switches	2wANOVA	Normal	14.57 ± 0.77	9.84 ± 1.32	8.52 ± 1.05	8.853	**0.001**	0.964	0.422	0.519	0.098	1.128	0.332	0.238	**0.009**	**0.001**	0.666
Number of new object exploration	2wANOVA	Nonnormal	22.73 ± 2.66	17.84 ± 2.68	6.26 ± 0.93	14.115	**0.000**	0.998	1.316	0.257	0.203	0.241	0.787	0.086	0.289	**0.000**	**0.002**
Number of pre-exposed object exploration	2wANOVA	Nonnormal	18.52 ± 4.35	13.05 ± 2.92	5.78 ± 0.76	3.898	**0.027**	0.678	1.389	0.244	0.212	1.540	0.224	0.312	0.413	**0.012**	0.216
Time exploring new object (s)	2wANOVA	Nonnormal	27.84 ± 3.58	21.74 ± 2.89	6.04 ± 1.39	20.724	**0.000**	1.000	11.516	**0.001**	0.915	0.182	0.835	0.077	0.225	**0.000**	**0.000**
Time exploring pre-exposed object (s)	2wANOVA	Nonnormal	12.49 ± 2.05	12.03 ± 2.55	3.8 ± 0.78	6.051	**0.004**	0.866	0.047	0.829	0.055	0.351	0.706	0.103	0.985	**0.009**	**0.014**
Latency new object (s)	2wANOVA	Nonnormal	16.68 ± 5.17	50.97 ± 18.18	72.65 ± 14.88	3.295	**0.045**	0.600	1.589	0.213	0.236	0.299	0.743	0.095	0.193	**0.043**	0.753
Latency to pre-exposed object (s)	2wANOVA	Nonnormal	30.73 ± 8.99	49.62 ± 18.45	68.71 ± 13.88	1.728	0.188	0.346	0.164	0.687	0.068	0.245	0.783	0.087	−	−	−
Total number of object exploration	2wANOVA	Nonnormal	41.26 ± 6.59	30.89 ± 5.37	12.05 ± 1.53	8.267	**0.001**	0.952	0.036	0.850	0.054	0.920	0.405	0.200	0.321	**0.000**	**0.029**
Total time exploringobjects (s)	2wANOVA	Nonnormal	40.33 ± 5.04	33.77 ± 4.55	9.85 ± 1.88	17.130	**0.000**	1.000	5.284	**0.026**	0.616	0.009	0.991	0.051	0.481	**0.000**	**0.000**
Novel object, latency to observe any object (s)	2wANOVA	Nonnormal	14.17 ± 4.79	16.53 ± 7.27	41.36 ± 11.68	2.538	*0.089*	0.485	3.004	*0.089*	0.398	0.853	0.432	0.188	0.581	*0.081*	0.459

Sniffing time, new vs pre-exposed object	Test	Data structure				All *F*	All *p* value	Power	WT *F*	WT *p* value	Power	Het *F*	Het *p* value	Power	KO *F*	KO *p* value	Power
- Side	rMeasures	Sph.ass				37.818	**0.000**	1.000	37.629	**0.000**	1.000	13.312	**0.002**	0.930	3.302	*0.087*	0.403
- Cohort	rMeasures	Sph.ass				2.146	0.149	0.302	1.221	0.285	0.181	1.164	0.296	0.175	14.732	**0.001**	0.951
- Side × cohort	rMeasures	Sph.ass				8.100	**0.006**	0.798	5.648	**0.029**	0.611	4.628	**0.046**	0.528	3.660	*0.073*	0.439
																	
Number of interactions, new vs pre-exposed	Test	Data structure				All *F*	All *p* value	Power	WT *F*	WT *p* value	Power	Het *F*	Het *p* value	Power	KO *F*	KO *p* value	Power
- Side	rMeasures	Sph.ass				9.499	**0.003**	0.857	3.824	0.067	0.454	11.442	**0.004**	0.890	0.314	0.582	0.083
- Cohor	rMeasures	Sph.ass				0.221	0.640	0.075	0.567	0.462	0.110	0.045	0.835	0.055	16.708	**0.001**	0.970
- Side × cohort	rMeasures	Sph.ass				9.882	**0.003**	0.870	5.751	**0.028**	0.618	5.258	**0.035**	0.580	1.686	0.211	0.232
																	
Time in new vs pre-exposed halves (s)	Test	Data structure				All *F*	All *p* value	Power	WT *F*	WT *p* value	Power	Het *F*	Het *p* value	Power	KO *F*	KO *p* value	Power
- Side	rMeasures	Sph.ass				0.565	0.456	0.114	0.029	0.867	0.053	0.185	0.673	0.069	1.375	0.257	0.198
- Cohort	rMeasures	Sph.ass				5.062	**0.028**	0.599	2.758	0.115	0.347	2.527	0.130	0.323	1.348	0.262	0.195
- Side × cohort	rMeasures	Sph.ass				0.020	0.889	0.052	0.010	0.920	0.051	0.446	0.513	0.097	0.397	0.537	0.091
																	
**Marble burying**	****	****	****	****	****	****	****	****	****	****	****	****	****	****	****	****	****
						Genotype			Cohort			Genotype × cohort			Pairwise comparisons		
	Test	Data structure	WT	Het	KO	*F*	*p* value	Power	*F*	*p* value	Power	*F*	*p* value	Power	WT vs Het	WT vs KO	Het vs KO
Number of burried marbles	2wANOVA	Nonnormal	13.63 ± 1.29	13.78 ± 1	3.77 ± 1.07	18.723	**0.000**	1.000	0.069	0.793	0.217	0.370	0.693	0.051	0.995	**0.000**	**0.000**
																	
**Repetitive novel object contact task, exploration**																	
						Genotype			Cohort			Genotype × cohort			Pairwise comparisons		
	Test	Data structure	WT	Het	KO	*F*	*p* value	Power	*F*	*p* value	Power	*F*	*p* value	Power	WT vs Het	WT vs KO	Het vs KO
Time exploring all the objects	2wANOVA	Nonnormal	82.08 ± 11.28	84.8 ± 7	53.37 ± 5.01	7.964	0.001	0.943	24.654	0.000	0.998	0.647	0.528	0.152	0.956	**0.014**	**0.006**
Total number of object interactions	2wANOVA	Normal	83.27 ± 7.92	94.21 ± 6.22	83.47 ± 6.4	2.108	0.133	0.412	73.475	0.000	1.000	1.110	0.338	0.234	−	−	−
																	
**Nest building**	****	****	****	****	****	****	****	****	****	****	****	****	****	****	****	****	****
						Genotype			Cohort			Genotype × cohort			Pairwise comparisons		
	Test	Data structure	WT	Het	KO	*F*	*p* value	Power	*F*	*p* value	Power	*F*	*p* value	Power	WT vs Het	WT vs KO	Het vs KO
Nest shredded	2wANOVA	Nonnormal	1.84 ± 0.08	1.94 ± 0.05	1.36 ± 0.15	7.785	**0.001**	0.939	2.814	0.100	0.377	0.364	0.697	0.105	0.455	**0.005**	**0.000**
Nest dispersion	2wANOVA	Nonnormal	1.94 ± 0.05	1.94 ± 0.05	1.73 ± 0.14	1.580	0.216	0.320	0.919	0.342	0.156	3.425	**0.040**	0.618	−	−	−
Nest density	2wANOVA	Nonnormal	1.26 ± 0.14	0.73 ± 0.16	0.73 ± 0.21	2.726	*0.075*	0.515	0.393	0.534	0.094	0.266	0.768	0.090	**0.050**	**0.046**	0.966
Nest shape	2wANOVA	Nonnormal	2.57 ± 0.19	2.05 ± 0.23	1.36 ± 0.27	5.851	**0.005**	0.854	3.065	*0.086*	0.404	0.097	0.908	0.064	0.153	**0.001**	*0.055*
Nest walls	2wANOVA	Nonnormal	1.21 ± 0.14	1 ± 0.18	0.42 ± 0.17	5.649	**0.006**	0.840	0.097	0.756	0.061	0.833	0.440	0.185	0.359	**0.002**	**0.023**
Nest total score	2wANOVA	Nonnormal	8.84 ± 0.45	7.68 ± 0.51	5.63 ± 0.73	7.223	**0.002**	0.921	1.121	0.295	0.180	0.020	0.980	0.053	0.184	**0.000**	**0.019**

WT, wild-type mice; Het, heterozygous mice; KO, homozygous knock-out mice. Group values are reported as mean ± SEM. Bold font indicates significant results (*p* < 0.05). Individual results and statistical analyses for cohorts 1 and 2 are available in Extended Data Table 9-1. 2wANOVA: 2-way ANOVA, rMeasures: repeated measures, Sph.ass: sphericity assumed, gen: genotype, coh: cohort.

10.1523/ENEURO.0046-18.2018.t9-1 Extended Data Table 9-1 **Individual results and statistical analyses for cohorts 1 and 2 related to the avoidance behavior.** WT, wild-type mice; Het, heterozygous mice; KO, homozygous knockout mice. Group values are reported as means ± s.e.m. Red font indicates significant results (p < 0.05), orange font indicates trends (0.1 < p < 0.05). Download Table 9-1, DOCX file.

**Figure 6.**
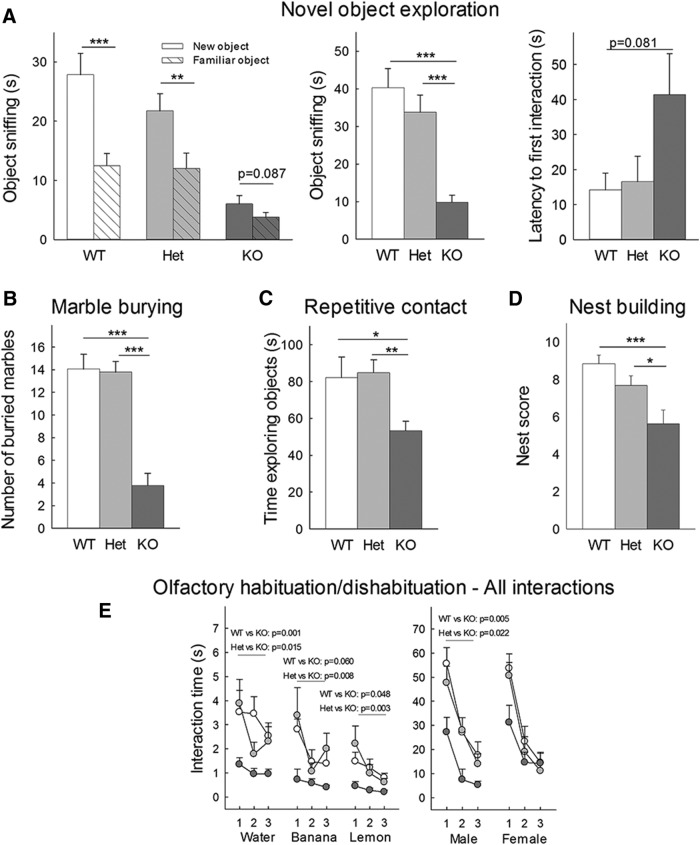
Object avoidance behavior in *Shank3^Δ4-22^-*deficient mice. ***A***, Short-term memory measured by the time of interaction with familiar and new object in the novel object recognition test. The test consisted of a training with two identical objects followed 1 h later by a testing session where one of the object was replaced by a novel object. During the testing session, both wild-type and *Shank3^Δ4-22^* heterozygous mice had a strong preference for the novel object over the familiar object, while *Shank3^Δ4-22^* homozygous mice failed to display a preference. However, this failure was due to an avoidance of both objects as shown by the strong decrease in object interaction and the increase in latency to explore any of the object for the first time in *Shank3^Δ4-22^* homozygous animals, rather than to a real lack of object preference. Representative heatmaps for the three genotypes are available in Extended Data Figure 6-1*A*. ***B***, Repetitive behavior and object avoidance measured in the marble burying test by the number of marble buried during a 30-min session. *Shank3^Δ4-22^*homozygous mice displayed a strongly impaired burying behavior, leaving most of the marbles undisturbed. Representative pictures and individual data are displayed in Extended Data Figure 6-1*B*. ***C***, Time spend exploring objects in the repetitive novel object contact task. *Shank3^Δ4-22^* homozygous mice spent significantly less time interacting with the objects than their wild-type and heterozygous littermates. ***D***, Nest building scores. *Shank3^Δ4-22^* homozygous mice are building less elaborate nests and use less nesting material than their wild-type and heterozygous littermates. Representative pictures of the nests and individual data are displayed in Extended Data Figure 6-1*C*. ***E***, Time interacting with the scent applicator (touching, biting, climbing) during the olfactory habituation/dishabituation test. *Shank3^Δ4-22^* homozygous mice are avoiding interaction with the scent applicator for all nonsocial scents and for a social male scent but have interaction level similar to wild-type and heterozygous animals when presented with a female scent. WT, wild-type mice; Het, heterozygous mice; KO, homozygous knock-out mice. *p < 0.05, **p < 0.1, ***p < 0.001.

This avoidance behavior was initially observed in the novel object recognition task. This highly validated test for recognition memory is designed to evaluate differences in the exploration time of novel and familiar objects. Mice are expected to spend more time investigating a novel object than a familiar object, and this is what was observed for wild-type and heterozygous mice (Fig. 6*A*, left panel). However, in homozygous mice, results were difficult to interpret due to strikingly reduced object interactions (Fig. 6*A*, left and middle panels). Homozygous mice spent most of both of the test sessions (the first involving familiarization with identical objects and the second involving interaction with one familiar and one novel object) away from both objects, spending excessive time in the corners of the open field as shown on heatmaps (Extended Data Fig. 6-1*A*) and demonstrating longer latency to explore any of the objects (Fig. 6*A*, right panel).

10.1523/ENEURO.0046-18.2018.f6-1 Extended Data Figure 6-1 **Pictures and individual scores representative of object avoidance behavior.** (A) Heatmaps of the novel object recognition task showing object interactions in wild-type and heterozygous Shank3^Δ4-22^ mice and object avoidance in homozygous Shank3^Δ4-22^ mice. (B) Representative picture of marble positions and individual scores in the marble burying test showing that in many instance Shank3^Δ4-22^ homozygous mice left the marbles completely undisturbed.(C) Representative picture of marble positions and individual scores in the nest building test showing a lower quality of nest building in Shank3^Δ4-22^ homozygous mice with some animals only coarsely shredding the nestlets without building a real nest and others even leaving the nestlets completely untouched. WT, wild-type mice; Het, heterozygous mice; KO, homozygous knockout mice. Download Figure 6-1, TIF file.

Object avoidance was further confirmed in multiple independent tests, including the marble burying, a test used to assess stereotypic behavior and/or anxiety. In this paradigm, 20 marbles were spread across the cage floor in a 4 × 5 pattern, leaving little space for the mice to move around the marbles. While both wild-type and *Shank3^Δ4-22^* heterozygous mice quickly buried most of the marbles as is typical, *Shank3^Δ4-22^* homozygous mice left the marbles almost completely undisturbed for the whole 15-min duration of the test (Fig. 6*B*; Extended Data Fig. 6-1*B*).

Consistent with these result, a significant decrease of the time spent exploring objects in the four-object exploration test was observed in the *Shank3^Δ4-22^* homozygous mice as compared to their littermate (Fig. 6*C*).

During assessment of nest building, nests build by *Shank3^Δ4-22^* homozygous mice were significantly less elaborate than nests built by wild-type or heterozygous mice, with some homozygotes leaving the nesting material completely untouched (Fig. 6*D*; Extended Data Fig. 6-1*C*). Note that, in an attempt to reduce stress and improve breeding rates, dams used to produce the cohorts described here were provided with plastic huts in their home cage. Interestingly, while most of the wild-type dams (seven out of ten) chose to build their nest inside the huts, only a single *Shank3^Δ4-22^* heterozygous dam out of 20 used the hut to establish their nests (wild-type vs heterozygotes: *t*_(28)_ = -5.085, *p* < 0.001). Additionally, three of the *Shank3^Δ4-22^* heterozygous dams did not build a nest until after the birth.

Object avoidance might also explain the reduction of the total time of direct interactions (grabbing, touching, biting, or climbing) with the applicator used to present the different scents during the olfactory habituation and dishabituation test in *Shank3^Δ4-22^* homozygous mice, compared to their wild-type and heterozygous littermates (Fig. 6*E*).

### Hyper-reactivity and escape behaviors in *Shank3^Δ4-22^*-deficient mice

**Figure 7.**
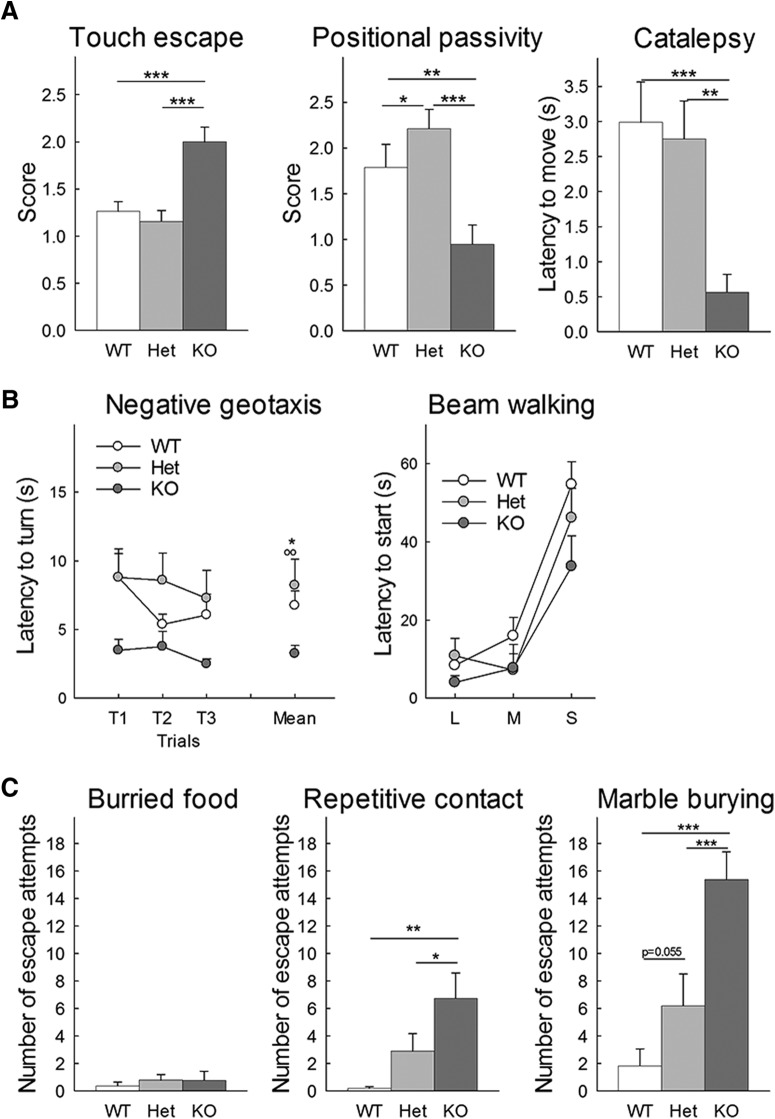
Hyper-reactivity and escape behavior in *Shank3^Δ4-22^-*deficient mice. ***A***, Hyper-reactivity measured by animal response in touch escape, positional passivity, and catalepsy. *Shank3^Δ4-22^* homozygous mice have hyper-reactive responses as shown by a higher score in the touch escape indicating an escape response to lighter strokes, a lower score in positional passivity indicating that they struggle more when restrained, and a lower latency to get off a rdownod in the catalepsy test. ***B***, Impulsivity in the negative geotaxis and beam walking tests. The latency to start turning in the negative geotaxis test and to start crossing in the beam walking test are significantly lower in *Shank3^Δ4-22^* homozygous mice compared to their wild-type and heterozygous littermates and often associated with higher failure rates (data not shown) thus demonstrating impulsive behavior. ***C***, Escape behavior measured in different tests with increased inanimate object exposure. No escape attempts were observed for any genotype during the habituation phase of the buried food test (empty home cage with clean bedding). Object exposure induced a significant escape behavior in *Shank3^Δ4-22^* homozygous mice with a number of attempts increasing with the number of objects in the cage (same home cage, four objects in the repetitive novel object contact task, 20 objects in the marble burying test). Very little escape attempts were observed in wild-type mice, while an intermediate phenotype was observed in heterozygous mice. WT, wild-type mice; Het, heterozygous mice; KO, homozygous knock-out mice. *: WT vs KO; o: WT vs Het. *p < 0.05, **p < 0.1, ***p < 0.001.

Unusual hyper-reactivity was observed in *Shank3^Δ4-22^* homozygous mice during handling and confirmed in several behavioral tests (Table 10). This hyper-reactivity was characterized by a higher score in the touch escape test (Fig. 7*A*, left panel), a lower score (reflecting a higher tendency to struggle in response to sequential handling) in the positional passivity (Fig. 7*A*, middle panel), and a shorter latency to move from the beam during the catalepsy test (Fig. 7*A*, right panel). As in newborn mice, a shorter latency to turn was seen for *Shank3^Δ4-22^* homozygous mice in the negative geotaxis test (Fig. 7*B*, left panel). Similarly, in the beam walking test, the latency to start crossing on the smallest beam was shorter in *Shank3^Δ4-22^* homozygous mice (Fig. 7*B*, right panel) but often led to a premature fall (Fig. 3*D*).

**Table 10.** Detailed results and statistical analyses related to the hyper-reactivity and escape behavior

**Reflexes and reactions to simple stimuli**																	
						Genotype			Cohort			Genotype × cohort			Pairwise comparisons		
	Test	Data structure	WT	Het	KO	*F*	*p* value	Power	*F*	*p* value	Power	*F*	*p* value	Power	WT vs Het	WT vs KO	Het vs KO
Touch escape	2wANOVA	Nonnormal	1.26 ± 0.1	1.15 ± 0.11	2 ± 0.15	12.962	**0.000**	0.996	0.046	0.831	0.055	0.862	0.428	0.190	0.648	**0.000**	**0.000**
Positional passivity (sum)	2wANOVA	Nonnormal	2.15 ± 0.25	1.84 ± 0.23	2.84 ± 0.2	11.737	**0.000**	0.992	106.722	**0.000**	1.000	1.993	0.147	0.393	**0.034**	**0.011**	**0.000**
Positional passivity (score)	2wANOVA	Nonnormal	1.78 ± 0.24	2.21 ± 0.21	0.94 ± 0.2	14.029	**0.000**	0.998	44.935	**0.000**	1.000	3.871	**0.027**	0.675	**0.034**	**0.004**	**0.000**
Catalepsy (4 trials)	2wANOVA	Nonnormal	2.98 ± 0.57	2.75 ± 0.54	0.56 ± 0.25	7.578	**0.001**	0.933	4.681	**0.035**	0.565	1.116	0.336	0.236	0.836	**0.001**	**0.002**
Trunk curl	2wANOVA	Nonnormal	1 ± 0	0.89 ± 0.07	1 ± 0	2.547	*0.088*	0.487	2.537	0.117	0.346	2.547	*0.088*	0.487	*0.057*	1.000	*0.056*
Negative geotaxis, latency to turn	2wANOVA	Nonnormal	6.73 ± 1.06	8.21 ± 1.9	3.22 ± 0.58	4.201	**0.020**	0.713	2.978	0.090	0.395	0.707	0.498	0.163	0.499	**0.042**	**0.008**
																	
**Beam walking**	****	****	****	****	****	****	****	****	****	****	****	****	****	****	****	****	****
						Genotype			Cohort			Genotype × cohort			Pairwise comparisons		
	Test	Data structure	WT	Het	KO	*F*	*p* value	Power	*F*	*p* value	Power	*F*	*p* value	Power	WT vsHet	WT vsKO	Het vs KO
Latency to cross (large, s)	2wANOVA	Nonnormal	8.34 ± 2.79	10.8 ± 4.52	3.96 ± 1.75	1.163	0.321	0.244	0.251	0.618	0.078	1.300	0.281	0.269	−	−	−
Latency to cross (medium, s)	2wANOVA	Nonnormal	15.9 ± 4.8	7.17 ± 4.16	7.65 ± 6.13	0.811	0.450	0.181	3.349	*0.073*	0.435	0.121	0.887	0.068	−	−	−
Latency to cross (small, s)	2wANOVA	Nonnormal	54.71 ± 5.89	46.15 ± 7.59	33.72 ± 7.84	2.204	0.121	0.430	0.235	0.630	0.076	0.437	0.648	0.117	−	−	−
																	
**Escape behavior**	****	****	****	****	****	****	****	****	****	****	****	****	****	****	****	****	****
						Genotype			Cohort			Genotype × cohort			Pairwise comparisons		
	Test	Datastructure	WT	Het	KO	*F*	*p* value	Power	*F*	*p* value	Power	*F*	*p* value	Power	WT vs Het	WT vs KO	Het vs KO
Buried food, number of attempts	2wANOVA	Nonnormal	0.63 ± 0.35	0.68 ± 0.32	0.42 ± 0.31	0.666	0.519	0.155	1.738	0.194	0.253	0.220	0.804	0.082			
Buried food, % of escapers	2wANOVA	Nonnormal	21.05 ± 9.6	26.31 ± 10.37	15.78 ± 8.59	0.760	0.473	0.172	3.639	*0.062*	0.464	0.159	0.853	0.073			
Four-object, number of attempts	2wANOVA	Nonnormal	0.41 ± 0.21	2.05 ± 0.73	3.88 ± 1.21	5.323	**0.008**	0.815	5.320	**0.025**	0.618	3.316	**0.045**	0.601	0.187	**0.002**	**0.050**
Four-object, % of escapers	2wANOVA	Nonnormal	23.52 ± 10.6	36.84 ± 11.36	50 ± 12.12	1.502	0.233	0.305	4.351	**0.042**	0.534	2.575	*0.087*	0.490			
Marble burying, number of attempts	2wANOVA	Nonnormal	4.32 ± 1.28	10.63 ± 1.98	16.05 ± 2.38	8.063	**0.001**	0.946	6.649	**0.013**	0.715	1.239	0.299	0.257	**0.034**	**0.000**	*0.055*
Marble burying, % of escapers	2wANOVA	Nonnormal	47.36 ± 11.76	94.73 ± 5.26	100 ± 0	12.009	**0.000**	0.993	7.713	**0.008**	0.777	4.474	**0.017**	0.740	**0.000**	**0.000**	0.598

WT, wild-type mice; Het, heterozygous mice; KO, homozygous knock-out mice. Group values are reported as mean ± SEM. Red font indicates significant results (*p* < 0.05). Individual results and statistical analyses for cohorts 1 and 2 are available in Extended Data Table 10-1. 2wANOVA: 2-way ANOVA.

10.1523/ENEURO.0046-18.2018.t10-1 Extended Data Table 10-1 **Individual results and statistical analyses for cohorts 1 and 2 related to the hyper-reactivity and escape behavior.** WT, wild-type mice; Het, heterozygous mice; KO, homozygous knockout mice. Group values are reported as means ± s.e.m. Red font indicates significant results (p < 0.05), orange font indicates trends (0.1 < p < 0.05). Download Table 10-1, DOCX file.

Escape attempts were observed in several tests and high-wall enclosures had to be built around testing cages to prevent successful attempts. Escape behaviors were scored in three different home cage tests. During the habituation portion of the buried food test (where no objects were visible at the surface of the cage bedding), no escape behavior nor genotype differences were observed (Fig. 7*C*, left panel). However, when the mice were tested in the same cages during the four-object interaction test both the number of escape attempts and the percentage of mice engaged in this behavior increased and significant genotype differences were observed (Fig. 7*C*, middle panel). This behavior was even more marked in the marble burying test (Fig. 7*C*, right panel), during which 94% of heterozygous mice and 100% of homozygous mice tried to escape. This indicated that the escape behavior is elicited by the presence of unfamiliar objects in the testing cage.

### Repetitive behaviors, stereotypies, and inflexibly in *Shank3^Δ4-22^*-deficient mice

Repetitive and restricted behaviors are one of the core features of ASD. Therefore, during all of the behavioral tests, mice were also carefully monitored for stereotypies, as well as perseverative and repetitive behaviors. Detailed results are reported in Table 11.

**Table 11.** Detailed results and statistical analyses related to stereotypies, repetitive behavior, perseveration, and cognitive flexibility

**Sterotypies in open field**																	
						Genotype			Cohort			Genotype × cohort			Pairwise comparisons		
	Test	Data structure	WT	Het	KO	*F*	*p* value	Power	*F*	*p* value	Power	*F*	*p* value	Power	WT vs Het	WT vs KO	Het vs KO
Grooming, time (s)	2wANOVA	Nonnormal	67.01 ± 7.01	62.6 ± 5.58	92.49 ± 10.45	4.929	**0.011**	**0.784**	22.806	0.000	0.997	2.530	0.090	0.484	0.883	**0.023**	**0.006**
Grooming, number	2wANOVA	Normal	25.42 ± 1.51	22.26 ± 1.5	27.1 ± 1.93	2.000	0.146	0.394	1.402	0.242	0.213	1.745	0.185	0.349	−	−	−
Jumping, time (s)	2wANOVA	Nonnormal	0.07 ± 0.05	0 ± 0	0.18 ± 0.1	1.666	0.199	0.335	0.033	0.857	0.054	1.149	0.325	0.242	−	−	−
Jumping, number	2wANOVA	Nonnormal	0.36 ± 0.23	0 ± 0	0.42 ± 0.23	1.300	0.281	0.269	0.155	0.696	0.067	1.816	0.173	0.362	−	−	−
Rotation, time (s)	2wANOVA	Nonnormal	0.39 ± 0.1	1.49 ± 0.81	4.21 ± 2.76	1.560	0.220	0.316	2.069	0.156	0.292	1.038	0.361	0.222	−	−	−
Rotation, number	2wANOVA	Nonnormal	1.63 ± 0.39	2.21 ± 0.46	6.15 ± 1.82	5.883	**0.005**	**0.856**	3.301	0.075	0.430	3.022	0.057	0.561	0.920	**0.010**	**0.028**
Twitching/shaking, time (s)	2wANOVA	Nonnormal	0.28 ± 0.07	0.69 ± 0.33	0.63 ± 0.1	1.089	0.344	0.231	0.540	0.466	0.111	0.879	0.422	0.193	−	−	−
Twitching/shaking, number	2wANOVA	Nonnormal	1.73 ± 0.42	2.63 ± 0.88	3 ± 0.47	1.194	0.311	0.250	1.484	0.229	0.223	0.589	0.559	0.143	−	−	−
																	
**Repetitive novel object contact task, object preference, time**																	
Object exploration, time (s)	Test	Data structure				*F*	*p* value	Power	WT vs Het	WT vs KO	Het vsKO						
- Object	rMeasures	Sph.viol				10.533	**0.000**	0.999	−	−	−						
- Object × gen.	rMeasures	Sph.viol				2.150	*0.069*	0.753	−	−	−						
- Genotype	rMeasures	Sph.viol				7.964	**0.001**	0.943	0.956	**0.014**	**0.006**						
- Cohort	rMeasures	Sph.viol				24.654	**0.000**	0.998	−	−	−						
- Object × gen. × coh.	rMeasures	Sph.viol				0.366	0.859	0.152	−	−	−						
- Gen. × coh.	rMeasures	Sph.viol				0.647	0.528	0.152	−	−	−						
																	
						Genotype			Cohort			Genotype × cohort			Pairwise comparisons		
Object exploration	Test	Data structure	WT	Het	KO	*F*	*p* value	Power	*F*	*p* value	Power	*F*	*p* value	Power	WT vs Het	WT vs KO	Het vs KO
Dice, time (s)	2wANOVA	Nonnormal	16.51 ± 2.49	14.82 ± 1.66	12.14 ± 1.79	1.748	0.185	0.349	4.640	0.036	0.560	0.833	0.441	0.185	−	−	−
Jack, time (s)	2wANOVA	Nonnormal	21.78 ± 3.18	27.99 ± 5.1	15.39 ± 2	4.078	**0.023**	0.697	14.158	0.000	0.958	0.500	0.610	0.127	0.311	0.443	**0.025**
Lego, time (s)	2wANOVA	Nonnormal	24.91 ± 3.59	28.25 ± 2.94	14.97 ± 1.87	8.622	**0.001**	0.959	20.965	0.000	0.994	0.774	0.467	0.174	0.509	**0.031**	**0.001**
Pin, time (s)	2wANOVA	Nonnormal	20.8 ± 4.3	13.72 ± 1.82	10.86 ± 1.7	3.199	**0.050**	0.585	4.532	0.038	0.550	0.057	0.944	0.058	0.244	*0.067*	0.755
																	
Object exploration, %	Test	Data structure				*F*	*p* value	Power	WT vs Het	WT vs KO	Het vsKO						
- Object	rMeasures	Sph.ass				8.329	**0.000**	0.985	−	−	−						
- Object × gen.	rMeasures	Sph.ass				0.721	0.633	0.259	−	−	−						
- Genotype	rMeasures	Sph.ass				0.750	0.478	0.170	−	−	−						
- Cohort	rMeasures	Sph.ass				0.000	1.000	0.050	−	−	−						
- Object × gen. × coh.	rMeasures	Sph.ass				0.652	0.688	0.236	−	−	−						
- Gen. × coh.	rMeasures	Sph.ass				0.000	1.000	0.050	−	−	−						
																	
						Genotype			Cohort			Genotype × cohort			Pairwise comparisons		
Object exploration	Test	Data structure	WT	Het	KO	*F*	*p* value	Power	*F*	*p*value	Power	*F*	*p* value	Power	WT vs Het	WT vs KO	Het vsKO
Dice, time (%)	2wANOVA	Nonnormal	20.09 ± 1.78	19.13 ± 2.11	24.11 ± 3.72	1.259	0.293	0.261	4.635	**0.036**	0.560	1.179	0.316	0.246	−	−	−
Jack, time (%)	2wANOVA	Nonnormal	26.86 ± 3.3	30.29 ± 3.61	28.38 ± 3.26	0.221	0.803	0.083	1.529	0.222	0.228	0.022	0.978	0.053	−	−	−
Lego, time (%)	2wANOVA	Normal	29.85 ± 2.19	32.98 ± 2.28	27.98 ± 2.5	1.174	0.318	0.245	0.986	0.326	0.164	0.072	0.931	0.060	−	−	−
Pin, time (%)	2wANOVA	Nonnormal	23.18 ± 3.02	17.57 ± 2.65	19.51 ± 2.32	0.701	0.501	0.161	0.186	0.669	0.071	1.684	0.196	0.337	−	−	−
																	
Object exploration ranked by preference	Test	Data structure				*F*	*p* value	Power	WT vs Het	WT vs KO	Het vsKO						
- Object	rMeasures	Sph.viol				110.887	**0.000**	1.000	−	−	−						
- Object × gen.	rMeasures	Sph.viol				5.483	**0.002**	0.996	−	−	−						
- Genotype	rMeasures	Sph.viol				8.054	**0.001**	0.946	0.948	**0.014**	**0.006**						
- Cohort	rMeasures	Sph.viol				24.578	**0.000**	0.998	−	−	−						
- Object × gen. × coh.	rMeasures	Sph.viol				1.187	0.321	0.457	−	−	−						
- Gen. × coh.	rMeasures	Sph.viol				0.643	0.530	0.152	−	−	−						
																	
						Genotype			Cohort			Genotype × cohort			Pairwise comparisons		
Object exploration	Test	Data structure	WT	Het	KO	*F*	*p* value	Power	*F*	*p* value	Power	*F*	*p*value	Power	WT vs Het	WT vs KO	Het vs KO
Object #1, time (s)	2wANOVA	Nonnormal	31.83 ± 3.59	37.45 ± 4.09	20.53 ± 1.64	9.051	**0.000**	0.967	15.093	**0.000**	0.968	0.934	0.400	0.202	0.264	**0.048**	**0.001**
Object #2, time (s)	2wANOVA	Nonnormal	24.5 ± 3.27	22.95 ± 2.14	15.23 ± 1.78	6.709	**0.003**	0.899	22.529	**0.000**	0.996	0.456	0.637	0.120	0.941	**0.016**	**0.033**
Object #3, time (s)	2wANOVA	Nonnormal	15.68 ± 2.69	15.29 ± 1.56	10.48 ± 1.15	4.224	**0.020**	0.714	20.037	**0.000**	0.992	1.306	0.280	0.269	0.998	*0.094*	0.102
Object #4, time (s)	2wANOVA	Nonnormal	12 ± 2.41	9.34 ± 0.96	7.12 ± 0.99	3.763	**0.030**	0.660	12.155	**0.001**	0.927	0.630	0.537	0.149	0.451	*0.074*	0.533
																	
Object exploration,ranked by preference	Test	Data structure				*F*	*p* value	Power	WT vs Het	WT vs KO	Het vsKO						
- Object	rMeasures	Sph.viol				146.534	**0.000**	1.000	−	−	−						
- Object × gen.	rMeasures	Sph.viol				0.832	0.490	0.321	−	−	−						
- Genotype	rMeasures	Sph.viol				0.812	0.450	0.181	−	−	−						
- Cohort	rMeasures	Sph.viol				0.783	0.381	0.140	−	−	−						
- Object × gen. × coh.	rMeasures	Sph.viol				1.054	0.377	0.407	−	−	−						
- Gen. × coh.	rMeasures	Sph.viol				0.812	0.450	0.181	−	−	−						
														
						Genotype			Cohort			Genotype × cohort			Pairwise comparisons		
Object exploration	Test	Data structure	WT	Het	KO	*F*	*p* value	Power	*F*	*p* value	Power	*F*	*p* value	Power	WT vs Het	WT vs KO	Het vs KO
Object #1, time (%)	2wANOVA	Nonnormal	39.5 ± 2.19	43 ± 1.71	40.58 ± 2.74	1.029	0.365	0.219	1.745	0.193	0.254	0.963	0.389	0.207	−	−	−
Object #2, time (%)	2wANOVA	Nonnormal	28.98 ± 1.16	27.21 ± 1.2	27.58 ± 1.39	0.805	0.453	0.180	1.231	0.273	0.193	2.400	0.102	0.461	−	−	−
Object #3, time (%)	2wANOVA	Normal	17.99 ± 1.18	18.73 ± 1.21	19.28 ± 1.12	0.093	0.911	0.063	0.228	0.635	0.076	0.347	0.709	0.102	−	−	−
Object #4, time (s)	2wANOVA	Normal	13.51 ± 0.92	11.62 ± 0.99	12.54 ± 1.16	1.091	0.344	0.230	0.423	0.518	0.098	0.747	0.479	0.169	−	−	−

**Repetitive novel object contact task, object preference, number**																	
Object interactions, number	Test	Data structure				*F*	*p* value	Power	WT vs Het	WT vs KO	Het vsKO						
- Object	rMeasures	Sph.viol				2.653	*0.051*	0.638	−	−	−						
- Object × gen.	rMeasures	Sph.viol				0.858	0.528	0.331	−	−	−						
- Genotype	rMeasures	Sph.viol				2.108	0.133	0.412	−	−	−						
- Cohort	rMeasures	Sph.viol				73.475	**0.000**	1.000	−	−	−						
- Object × gen. × coh.	rMeasures	Sph.viol				0.459	0.837	0.184	−	−	−						
- Gen. × coh.	rMeasures	Sph.viol				1.110	0.338	0.234	−	−	−						
																	
						Genotype			Cohort			Genotype × cohort			Pairwise comparisons		
Exploration numbers	Test	Data structure	WT	Het	KO	*F*	*p* value	Power	*F*	*p*value	Power	*F*	*p*value	Power	WT vs Het	WT vs KO	Het vsKO
Dice	2wANOVA	Nonnormal	20.17 ± 1.7	21.05 ± 1.39	20.76 ± 1.69	0.054	0.948	0.058	15.707	**0.000**	0.973	0.620	0.542	0.148	−	−	−
Jack	2wANOVA	Normal	20.17 ± 2.46	23.42 ± 2.38	20.94 ± 1.95	1.196	0.311	0.249	72.111	**0.000**	1.000	1.200	0.310	0.250	−	−	−
Lego	2wANOVA	Normal	23.94 ± 2.46	26.31 ± 1.71	20.82 ± 2	2.785	*0.072*	0.523	17.510	**0.000**	0.984	0.367	0.695	0.106	0.495	0.573	*0.091*
Pin	2wANOVA	Normal	20.17 ± 2.46	23.42 ± 2.38	20.94 ± 1.95	1.196	0.311	0.249	72.111	**0.000**	1.000	1.200	0.310	0.250	−	−	−
																	
Object interaction %	Test	Data structure				*F*	*p* value	Power	WT vs Het	WT vs KO	Het vsKO						
- Object	rMeasures	Sph.viol				4.812	**0.022**	0.897	−	−	−						
- Object × gen.	rMeasures	Sph.viol				0.363	0.762	0.151	−	−	−						
- Genotype	rMeasures	Sph.viol				0.328	0.722	0.099	−	−	−						
- Cohort	rMeasures	Sph.viol				7.375	**0.009**	0.758	−	−	−						
- Object × gen. × coh.	rMeasures	Sph.viol				0.560	0.627	0.220	−	−	−						
- Gen. × coh.	rMeasures	Sph.viol				0.328	0.722	0.099	−	−	−						
																	
						Genotype			Cohort			Genotype × cohort			Pairwise comparisons		
Object interaction number	Test	Datastructure	WT	Het	KO	*F*	*p* value	Power	*F*	*p*value	Power	*F*	*p*value	Power	WT vs Het	WT vs KO	Het vsKO
Dice	2wANOVA	Normal	24.87 ± 1.44	23.13 ± 1.27	25.81 ± 1.7	1.119	0.335	0.235	10.075	**0.003**	0.875	0.297	0.745	0.094	−	−	−
Jack	2wANOVA	Normal	23.14 ± 1.35	24.09 ± 1.09	24.69 ± 0.91	0.164	0.849	0.074	11.956	**0.001**	0.923	0.546	0.583	0.135	−	−	−
Lego	2wANOVA	Nonnormal	28.84 ± 2	28.66 ± 1.57	24.78 ± 1.41	1.507	0.232	0.305	2.901	0.095	0.386	1.286	0.286	0.265	−	−	−
Pin	2wANOVA	Nonnormal	33.55 ± 7.69	32.7 ± 6.64	30.85 ± 4.83	0.235	0.792	0.085	10.476	**0.002**	0.887	0.467	0.630	0.122	−	−	−
																	
Object interaction number, object ranked by preference	Test	Data structure				*F*	*p* value	Power	WT vs Het	WT vs KO	Het vsKO						
- Object	rMeasures	Sph.viol				74.224	**0.000**	1.000	−	−	−						
- Object × gen.	rMeasures	Sph.viol				0.867	0.499	0.335	−	−	−						
- Genotype	rMeasures	Sph.viol				2.228	0.119	0.432	−	−	−						
- Cohort	rMeasures	Sph.viol				72.229	**0.000**	1.000	−	−	−						
- Object × gen. × coh.	rMeasures	Sph.viol				0.653	0.649	0.254	−	−	−						
- Gen. × coh.	rMeasures	Sph.viol				1.142	0.328	0.239	−	−	−						
						Genotype			Cohort			Genotype × cohort			Pairwise comparisons		
Object interaction number	Test	Data structure	WT	Het	KO	*F*	*p* value	Power	*F*	*p*value	Power	*F*	*p* value	Power	WT vs Het	WT vs KO	Het vsKO
Object #1	2wANOVA	Normal	26.88 ± 2.3	29.15 ± 1.88	25.58 ± 1.75	1.682	0.197	0.337	30.522	**0.000**	1.000	0.173	0.842	0.075	−	−	−
Object #2	2wANOVA	Nonnormal	23.23 ± 2.05	25.15 ± 2.07	21.64 ± 1.85	2.474	*0.095*	0.473	59.810	**0.000**	1.000	0.752	0.477	0.170	0.421	0.829	0.167
Object #3	2wANOVA	Nonnormal	17.94 ± 2.12	21.94 ± 1.92	18.88 ± 1.7	2.307	0.110	0.446	66.844	**0.000**	1.000	1.460	0.242	0.297	−	−	−
Object #4	2wANOVA	Nonnormal	16.41 ± 1.91	17.94 ± 1.34	16.94 ± 1.62	0.441	0.646	0.118	54.987	**0.000**	1.000	2.377	0.104	0.457	−	−	−
																	
Object interaction %, object ranked by preference	Test	Data structure				*F*	*p* value	Power	WT vs Het	WT vs KO	Het vs KO						
- Object	rMeasures	Sph.viol				86.885	**0.000**	**1.000**	−	−	−						
- Object × gen.	rMeasures	Sph.viol				0.745	0.578	0.288	−	−	−						
- Genotype	rMeasures	Sph.viol				1.010	0.411	0.207	−	−	−						
- Cohort	rMeasures	Sph.viol				0.960	0.390	0.163	−	−	−						
- Object × gen. × coh.	rMeasures	Sph.viol				0.979	0.327	0.390	−	−	−						
- Gen. × coh.	rMeasures	Sph.viol				0.960	0.390	0.207	−	−	−						
														
						Genotype			Cohort			Genotype × cohort			Pairwise comparisons		
Object interaction %	Test	Data structure	WT	Het	KO	*F*	*p* value	Power	*F*	*p* value	Power	*F*	*p* value	Power	WT vs Het	WT vs KO	Het vs KO
Object #1	2wANOVA	Nonnormal	32.55 ± 1.41	31.48 ± 1.02	31.19 ± 0.99	0.066	0.936	0.059	11.328	**0.002**	0.910	0.966	0.388	0.208	−	−	−
Object #2	2wANOVA	Normal	28.09 ± 0.86	26.68 ± 0.74	25.78 ± 0.8	1.433	0.249	0.292	0.009	0.926	0.051	0.256	0.775	0.088	−	−	−
Object #3	2wANOVA	Nonnormal	20.49 ± 0.83	22.71 ± 0.81	22.52 ± 0.79	1.538	0.225	0.311	9.140	**0.004**	0.842	2.049	0.140	0.402	−	−	−
Object #4	2wANOVA	Normal	18.85 ± 0.7	19.11 ± 0.83	20.08 ± 0.64	0.454	0.638	0.120	2.559	0.116	0.348	0.975	0.385	0.210	−	−	−
																	
**Repetitive novel object contact task, pattern of object investigation**																	
						Genotype			Cohort			Genotype × cohort			Pairwise comparisons		
Three-object sequences	Test	Data structure	WT	Het	KO	*F*	*p* value	Power	*F*	*p* value	Power	*F*	*p* value	Power	WT vs Het	WT vs KO	Het vs KO
Total number of 3-object choices	2wANOVA	Normal	56.11 ± 3.2	53.57 ± 4.82	53.11 ± 3.29	0.077	0.926	0.061	12.053	**0.001**	0.925	0.573	0.567	0.140	−	−	−
Number of different 3-object sequences	2wANOVA	Normal	26.5 ± 0.57	25.36 ± 1.21	25.47 ± 0.93	0.405	0.669	0.112	7.502	**0.009**	0.765	0.830	0.442	0.184	−	−	−

Number of repetition of top preferred sequence	2wANOVA	Nonnormal	4.88 ± 0.4	4.78 ± 0.37	4.64 ± 0.29	0.012	0.988	0.052	10.796	**0.002**	0.896	0.052	0.950	0.057	−	−	−
Number of repetition of 2nd preferred sequence	2wANOVA	Nonnormal	4.27 ± 0.27	4.15 ± 0.33	4.05 ± 0.26	0.017	0.983	0.052	8.748	**0.005**	0.826	0.312	0.733	0.097	−	−	−
Number of repetition of 3rd preferred sequence	2wANOVA	Nonnormal	3.83 ± 0.23	3.68 ± 0.3	3.7 ± 0.25	0.042	0.959	0.056	6.542	**0.014**	0.708	0.303	0.740	0.095	−	−	−
Number of repetition of top 3 preferred sequences	2wANOVA	Normal	13 ± 0.87	12.63 ± 0.98	12.41 ± 0.78	0.008	0.992	0.051	9.545	**0.003**	0.857	0.146	0.865	0.071	−	−	−
% top preferred sequence choice	2wANOVA	Nonnormal	8.57 ± 0.32	9.41 ± 0.61	8.75 ± 0.19	1.324	0.276	0.272	0.000	0.994	0.050	1.436	0.248	0.293	−	−	−
% top 2 preferred sequence choice	2wANOVA	Nonnormal	16.22 ± 0.52	17.48 ± 1.01	16.43 ± 0.32	1.179	0.316	0.246	0.143	0.707	0.066	1.543	0.224	0.312	−	−	−
% top 3 preferred sequence choice	2wANOVA	Nonnormal	23.14 ± 0.71	24.69 ± 1.43	23.44 ± 0.51	0.837	0.439	0.185	0.564	0.456	0.114	1.040	0.361	0.221	−	−	−
																	
						Genotype			Cohort			Genotype × cohort			Pairwise comparisons		
Four-object sequences	Test	Data structure	WT	Het	KO	*F*	*p* value	Power	*F*	*p* value	Power	*F*	*p* value	Power	WT vs Het	WT vs KO	Het vs KO
Total number of 4-object choices	2wANOVA	Normal	55.55 ± 3.26	53.26 ± 4.79	52.58 ± 3.24	0.067	0.935	0.060	10.400	**0.002**	0.885	0.528	0.593	0.132	−	−	−
Number of different 4-object sequences	2wANOVA	Normal	40.77 ± 1.59	39.63 ± 2.78	39 ± 2.03	0.097	0.908	0.064	9.857	**0.003**	0.868	0.895	0.415	0.195	−	−	−
Number of repetition of top preferred sequence	2wANOVA	Nonnormal	3.05 ± 0.2	3.1 ± 0.2	3.41 ± 0.17	1.297	0.283	0.267	4.144	**0.047**	0.514	0.050	0.951	0.057	−	−	−
Number of repetition of 2nd preferred sequence	2wANOVA	Nonnormal	2.83 ± 0.2	2.84 ± 0.2	2.76 ± 0.18	0.034	0.967	0.055	4.324	**0.043**	0.531	1.214	0.306	0.253	−	−	−
Number of repetition of 3rd preferred sequence	2wANOVA	Nonnormal	2.44 ± 0.16	2.47 ± 0.19	2.23 ± 0.13	0.468	0.629	0.122	4.499	**0.039**	0.547	1.063	0.353	0.225	−	−	−
Number of repetition of top 3 preferred sequences	2wANOVA	Nonnormal	8.33 ± 0.53	8.42 ± 0.55	8.41 ± 0.42	0.087	0.916	0.063	5.267	**0.026**	0.614	0.634	0.535	0.150	−	−	−
% top preferred sequence choice	2wANOVA	Nonnormal	5.58 ± 0.3	6.31 ± 0.47	6.66 ± 0.27	2.187	0.123	0.425	2.759	0.103	0.370	1.056	0.356	0.224	−	−	−
% top 2 preferred sequence choice	2wANOVA	Nonnormal	10.7 ± 0.44	11.99 ± 0.77	12.09 ± 0.51	1.734	0.187	0.346	2.734	0.105	0.367	1.166	0.320	0.244	−	−	−
% top 3 preferred sequence choice	2wANOVA	Nonnormal	15.14 ± 0.54	16.93 ± 1.02	16.43 ± 0.67	1.557	0.221	0.314	3.181	*0.081*	0.416	1.237	0.299	0.257	−	−	−
																	
**Barnes maze initial training - distance**																	
Distance	Test	Data structure				*F*	*p* value	Power	WT vs Het	WT vs KO	Het vsKO						
- Day	rMeasures	Sph.ass				13.695	**0.000**	1.000	−	−	−						
- Day × gen.	rMeasures	Sph.ass				2.062	*0.062*	0.684	−	−	−						
- Genotype	rMeasures	Sph.ass				2.663	*0.080*	0.503	0.659	0.145	0.515						
- Cohort	rMeasures	Sph.ass				11.841	**0.001**	0.920	−	−	−						
- Day × gen. × coh.	rMeasures	Sph.ass				1.173	0.324	0.416	−	−	−						
- Gen. × coh.	rMeasures	Sph.ass				1.114	0.337	0.234	−	−	−						
																	
						Genotype			Cohort			Genotype × cohort			Pairwise comparisons		
Individual days	Test	Data structure	WT	Het	KO	*F*	*p* value	Power	*F*	*p* value	Power	*F*	*p*value	Power	WT vsHet	WT vsKO	Het vs KO
Day 1	2wANOVA	Normal	501.24 ± 48.28	485.42 ± 47.71	484.49 ± 53.07	0.003	0.997	0.050	4.283	**0.044**	0.526	0.084	0.919	0.062	−	−	−
Day 2	2wANOVA	Normal	427.6 ± 43.5	468.59 ± 40.26	504.18 ± 47.17	1.234	0.301	0.256	9.205	**0.004**	0.844	1.918	0.158	0.378	−	−	−
Day 3	2wANOVA	Normal	292.36 ± 29.11	340.26 ± 31.24	485.74 ± 41.16	11.293	**0.000**	0.989	6.902	**0.012**	0.730	3.082	*0.055*	0.567	0.496	**0.000**	**0.005**
Day 4	2wANOVA	Normal	311.01 ± 34.75	370.86 ± 29.61	367.31 ± 42.11	1.479	0.239	0.300	5.449	**0.024**	0.628	0.666	0.519	0.155	−	−	−
																	
**Barnes maze reversal - distance**																	
Distance	Test	Data structure				*F*	*p* value	Power	WT vs Het	WT vs KO	Het vs KO						
- Day effect	rMeasures	Sph.ass				26.455	**0.000**	1.000	−	−	−						
- Day × gen.	rMeasures	Sph.ass				2.612	**0.023**	0.824	−	−	−						
- Genotype	rMeasures	Sph.ass				1.811	0.175	0.359	−	−	−						
- Cohort	rMeasures	Sph.ass				1.924	0.172	0.274	−	−	−						
- Day × gen. × coh.	rMeasures	Sph.ass				3.192	**0.007**	0.902	−	−	−						
- Genotype × cohort effect	rMeasures	Sph.ass				0.290	0.750	0.093	−	−	−						
																	
						Genotype			Cohort			Genotype × cohort			Pairwise comparisons		
Individual days	Test	Data structure	WT	Het	KO	*F*	*p* value	Power	*F*	*p*value	Power	*F*	*p* value	Power	WT vs Het	WT vs KO	Het vsKO
Day 1	2wANOVA	Nonnormal	420.93 ± 37.75	437.03 ± 37.86	591.59 ± 38.48	5.592	**0.007**	0.834	0.793	0.378	0.141	1.475	0.239	0.299	0.948	**0.009**	**0.018**
Day 2	2wANOVA	Normal	336.81 ± 35.36	413.64 ± 32.4	390.91 ± 45	1.285	0.286	0.265	0.374	0.544	0.092	2.525	0.091	0.481	−	−	−
Day 3	2wANOVA	Normal	357.93 ± 35.96	421.04 ± 44.36	395.85 ± 48.06	0.666	0.519	0.155	3.371	*0.073*	0.436	0.116	0.890	0.067	−	−	−
Day 4	2wANOVA	Normal	288.54 ± 39.85	288.65 ± 37.41	337.24 ± 38.59	0.965	0.389	0.207	8.849	**0.005**	0.829	1.373	0.264	0.281	−	−	−
																	
**Barnes maze initial training probe test**																	
						Genotype			Quadrant pairwise comparisons								
All animals	Test	Data structure				*F*	*p* value	Power	T vs L	T vs R	T vs O	L vsR	L vs O	R vs O			
- Quadrant	rMeasures	Sph.viol				296.653	**0.000**	1.000	**0.000**	**0.000**	**0.000**	0.555	0.201	0.628			
- Cohort	rMeasures	Sph.viol				10.200	**0.002**	1.000									
- Quadrant × coh.	rMeasures	Sph.viol				11.435	**0.000**	0.983									

WT	Test	Data structure				*F*	*p* value	Power	T vs L	T vs R	T vs O	L vs R	L vs O	R vs O			
- Quadrant	rMeasures	Sph.viol				58.318	**0.000**	1.000	**0.000**	**0.000**	**0.000**	*0.057*	0.168	0.335			
- Cohort	rMeasures	Sph.viol				9.373	**0.007**	0.820									
- Quadrant × coh.	rMeasures	Sph.viol				4.241	**0.010**	0.831									
																	
Het	Test	Data structure				*F*	*p* value	Power	T vs L	T vs R	T vs O	L vsR	L vsO	R vs O			
- Quadrant	rMeasures	Sph.viol				107.980	**0.000**	1.000	**0.000**	**0.000**	**0.000**	0.205	0.895	0.278			
- Cohort	rMeasures	Sph.viol				65.390	**0.000**	1.000									
- Quadrant × coh.	rMeasures	Sph.viol				5.366	**0.003**	0.915									
																	
KO	Test	Data structure				*F*	*p* value	Power	T vsL	T vsR	T vsO	L vsR	L vs O	R vs O			
- Quadrant	rMeasures	Sph.viol				378.546	**0.000**	1.000	**0.000**	**0.000**	**0.000**	0.832	0.278	0.341			
- Cohort	rMeasures	Sph.viol				890.226	**0.000**	1.000									
- Quadrant × coh.	rMeasures	Sph.viol				1.683	0.186	0.406									
																	
						Genotype			Cohort			Genotype × cohort			Pairwise comparisons		
Probe test quadrants	Test	Data structure	WT	Het	KO	*F*	*p* value	Power	*F*	*p* value	Power	*F*	*p* value	Power	WT vsHet	WT vsKO	Het vsKO
Target	2wANOVA	Nonnormal	107.29 ± 7.96	125.68 ± 7.96	142.87 ± 4.53	5.342	**0.008**	0.816	12.144	**0.001**	0.927	0.935	0.400	0.202	0.112	**0.001**	0.173
Left	2wANOVA	Nonnormal	28.57 ± 4.6	14.87 ± 2.32	11.37 ± 2.49	7.081	**0.002**	0.914	4.207	**0.046**	0.519	0.660	0.522	0.154	**0.010**	**0.002**	0.740
Right	2wANOVA	Nonnormal	16.92 ± 3.86	20.57 ± 4.93	12.28 ± 2.15	1.097	0.342	0.231	7.056	**0.011**	0.739	1.048	0.359	0.222	0.763	0.678	0.290
Opposite	2wANOVA	Nonnormal	22.28 ± 4.49	14.37 ± 3.47	9.2 ± 2.09	2.438	*0.099*	0.466	10.443	**0.002**	0.886	1.149	0.326	0.240	0.199	**0.024**	0.526
																	
**Barnes maze reversal probe test**																	
						Genotype			Quadrant pairwise comparisons								
All animals	Test	Data structure				*F*	*p* value	Power	T vs L	T vsR	T vs O	L vsR	L vs O	R vs O			
- Quadrant	rMeasures	Sph.viol				50.865	**0.000**	**1.000**	**0.000**	**0.000**	**0.000**	0.242	**0.000**	**0.000**			
- Cohort	rMeasures	Sph.viol				24.530	**0.000**	**0.998**									
- Quadrant × coh.	rMeasures	Sph.viol				4.443	**0.005**	**0.870**									
																	
WT	Test	Data structure				*F*	*p* value	Power	T vsL	T vsR	T vsO	L vs R	L vs O	R vs O			
- Quadrant	rMeasures	Sph.viol				32.279	**0.000**	**1.000**	**0.000**	**0.000**	**0.000**	**0.005**	**0.024**	**0.003**			
- Cohort	rMeasures	Sph.viol				159.377	**0.000**	**1.000**									
- Quadrant × coh.	rMeasures	Sph.viol				0.007	0.956	0.051									
																	
Het	Test	Data structure				*F*	*p* value	Power	T vs L	T vs R	T vs O	L vsR	L vs O	R vs O			
- Quadrant	rMeasures	Sph.viol				28.198	**0.000**	**1.000**	**0.000**	**0.000**	**0.001**	0.235	**0.001**	*0.086*			
- Cohort	rMeasures	Sph.viol				6.412	**0.021**	**0.666**									
- Quadrant × coh.	rMeasures	Sph.viol				10.315	**0.000**	**0.998**									
																	
KO	Test	Data structure				*F*	*p* value	Power	T vsL	T vs R	T vs O	L vs R	L vs O	R vs O			
- Quadrant	rMeasures	Sph.viol				12.026	**0.000**	**0.999**	**0.000**	**0.010**	0.646	*0.070*	**0.000**	**0.000**			
- Cohort	rMeasures	Sph.viol				397.250	**0.000**	**1.000**									
- Quadrant × coh.	rMeasures	Sph.viol				2.273	*0.095*	0.531									
																	
						Genotype			Cohort			Genotype × cohort			Pairwise comparisons		
Probe test quadrants	Test	Data structure	WT	Het	KO	*F*	*p* value	Power	*F*	*p* value	Power	*F*	*p*value	Power	WT vs Het	WT vs KO	Het vsKO
Target	2wANOVA	Nonnormal	115.33 ± 11.27	105.35 ± 12.55	67.82 ± 11.65	5.430	**0.008**	0.822	8.183	**0.006**	0.800	3.469	**0.040**	0.621	0.773	**0.010**	**0.046**
Left	2wANOVA	Nonnormal	18.44 ± 3.76	8.03 ± 2.15	9.68 ± 2.79	3.343	**0.044**	0.604	2.079	0.156	0.292	0.172	0.842	0.075	**0.039**	0.123	0.923
Right	2wANOVA	Nonnormal	9.8 ± 2.46	18.48 ± 7.8	23.17 ± 5.32	1.367	0.265	0.280	1.873	0.178	0.268	2.980	0.061	0.551	0.489	0.229	0.826
Opposite	2wANOVA	Nonnormal	32.67 ± 6.87	42.82 ± 10	75.15 ± 10.65	6.632	**0.003**	0.894	7.210	**0.010**	0.748	2.097	0.134	0.409	0.662	**0.004**	0.030

WT, wild-type mice; Het, heterozygous mice; KO, homozygous knock-out mice. Group values are reported as mean ± SEM. Bold font indicates significant results (*p* < 0.05). Individual results and statistical analyses for cohorts 1 and 2 are available in Extended Data Table 11-1. 2wANOVA: 2-way ANOVA, rMeasures: repeated measures, Norm: normal, No-norm: non-normal, Sph.ass: sphericity assumed, Sph.viol: sphericity violated, gen: genotype, coh: cohort. 2wANOVA: 2-way ANOVA, rMeasures: repeated measures, Sph.ass: sphericity assumed, Sph.viol: sphericity violated, gen: genotype, coh: cohort.

10.1523/ENEURO.0046-18.2018.t11-1 Extended Data Table 11-1 **Individual results and statistical analyses for cohorts 1 and 2 related to stereotypies, repetitive behavior, perseveration and cognitive flexibility.** WT, wild-type mice; Het, heterozygous mice; KO, homozygous knockout mice. Group values are reported as means ± s.e.m. Red font indicates significant results (p < 0.05), orange font indicates trends (0.1 < p < 0.05). Download Table 11-1, DOCX file.

**Figure 8.**
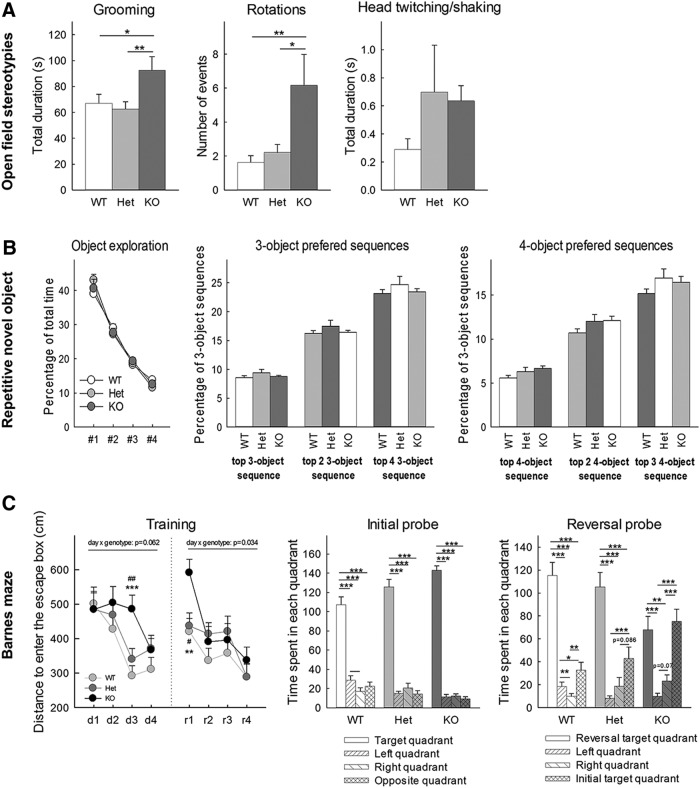
Repetitive behavior, stereotypies, and cognitive flexibility in *Shank3^Δ4-22^-*deficient mice. ***A***, Repetitive behaviors in the open field test. *Shank3^Δ4-22^* homozygous mice engaged in significantly more self-grooming and rotations relative to the other genotypes. A trend toward an increase amount of head stereotypies was also observed. ***B***, Object preference and pattern of exploration in the repetitive novel object contact task. For each mouse, the time spent interacting with each object was measured and the objects were then ranked from the most (1) to less (4) preferred (left panel). No genotype differences were observed for the proportions of visits to each object. The pattern of object exploration was analyzed by recording specific sequential pattern of visits to three or four specific toys to identify the total number of three-object or four-object sequence investigations, the number of unique sequences, and the percentage of choices of the top, top two, or top three preferred sequences. All groups had identical percentage of their preferred three-object or four-object sequences choices over the total number of sequence choices. ***C***, Cognitive flexibility measured by reversal learning in the Barnes maze. During initial learning (d1 to d4, each point represents the mean of traveled distance for four independent trials), improvement shown by reduction of the travel distance was faster in *Shank3^Δ4-22^* wild-type and heterozygous mice than in homozygous animals; however, by day 4, the three groups were not different anymore and all of them had a strong preference for the escape hole quadrant during the initial probe test. During the reversal training (r1 to r4, each point represents the mean of travel distance for four independent trials), *Shank3^Δ4-22^* homozygous mice initially traveled for longer distances but were still able to learn the new position and performed as well as their littermates on reversal days 2, 3, and 4. However, the reversal probe test at the end of the reversal training showed that while wild-type and heterozygous animals had a significant preference for the new target quadrant, the homozygous mice had a similar preference for the quadrants containing the initial and the reversal escape holes. WT, wild-type mice; Het, heterozygous mice; KO, homozygous knock-out mice. *: WT versus KO; #: Het versus KO. *p < 0.05, **p < 0.1, ***p < 0.001.

While no genotype difference was observed in the number of spontaneous grooming bouts observed during the 10 first minutes of the open field test, *Shank3^Δ4-22^*homozygous mice engaged in longer episodes of self-grooming, as shown by a significant increase in the cumulative time spent grooming all body regions when compared to their wild-type and heterozygous littermates. However, skin lesions were frequently observed in older mice (over eight-month-old) of all three genotypes without obvious genotype effect. Significantly more rotations were also observed in *Shank3^Δ4-22^* homozygous animals as well as a trend toward an increase of head twitching/shaking in both *Shank3^Δ4-22^*heterozygous and homozygous mice, as compared to their wild-type littermates (Fig. 8*A*).

Object preferences, exploration patterns and frequency of repetitive contacts with novel objects were evaluated in the repetitive novel object contact task. Although the cumulative time spend interacting with the objects was decreased in *Shank3^Δ4-22^* homozygous mice (Fig. 6*C*), this test failed to display genotype difference in either the total number of interactions, the preference for any specific objects or the preference for any specific preferred sequence of three-object or four-object explorations (Fig. 8*B*).

Individuals with ASD can maintain rigid habits and frequently show strong insistence on sameness and upset by changes in routine. To examine this domain, *Shank3^Δ4-22^* mice were trained for 4 d in the Barnes maze, a test of spatial learning and memory, until all the mice were able to quickly locate an escape box hidden under one of the target locations, then the location of the escape box was moved and mice were tested for reversal learning for four additional days. During the initial learning, all the genotypes were able to find the escape hatch equally well, although *Shank3^Δ4-22^* homozygous mice took 1 d longer to reach criteria (Fig. 8*C*, left panel). All genotypes preferred the correct quadrant in the first probe test ran immediately after the initial training (Fig. 8*C*, middle panel). When the escape hatch was moved to the opposite side of the maze, both *Shank3^Δ4-22^* wild-type and heterozygotes immediately learned the new position, while a 1-d delay was, once again, observed for the *Shank3^Δ4-22^* homozygous mice. Genotypes differed markedly in the second probe test, however; while wild-type mice spent most time in the new target quadrant, *Shank3* ^Δ4–22^ heterozygous mice split their time 75/25% between new and old targets, whereas Shank3^Δ4–22^ homozygous animals spent equal time in both targets (Fig. 8*C*, right panel). This impaired reversal learning implies that *Shank3* deficiency increases susceptibility to proactive interference where learning of a previous rule interferes with the new rule.

### Learning and memory in *Shank3^Δ4-22^*-deficient mice

In addition to the Barnes maze, animals were tested in two additional learning and memory tests, specifically, the Y-maze spontaneous alternation test and the fear conditioning test. Detailed results are reported in Table 12.

**Table 12.** Detailed results and statistical analyses related to learning and memory

**Y-maze, spontaneous alternation behavior**																	
						Genotype			Cohort			Genotype × cohort			Pairwise comparisons		
% of choices		Data structure	WT	Het	KO	*F*	*p* value	Power	*F*	*p* value	Power	*F*	*p* value	Power	WT vs Het	WT vs KO	Het vs KO
Arm 1	2wANOVA	Normal	32.34 ± 0.87	34.24 ± 0.92	32.77 ± 1.17	0.844	0.436	0.187	0.035	0.852	0.054	0.412	0.664	0.113	−	−	-
Arm 2	2wANOVA	Normal	35.17 ± 1.17	32.74 ± 1.1	35.18 ± 1.36	1.548	0.223	0.314	9.976	**0.003**	0.873	0.119	0.888	0.067	−	−	-
Arm 3	2wANOVA	Normal	32.19 ± 1.46	32.98 ± 1.09	32.04 ± 1.02	0.285	0.753	0.093	10.366	**0.002**	0.885	0.520	0.598	0.131	−	−	-
																	
Chance level comparison	Test	Data structure				All t	All *p* value	Power	WT t	WT *p* value	Power	Het t	Het *p* value	Power	KO t	KO *p* value	Power
Arm 1	1S-*t* test	Normal				Normal	Normal	Normal	Normal	Normal	Normal	Normal	Normal	Normal	Normal	Normal	Normal
Arm 2	1S-*t* test	Normal				1.465	0.148	NA	1.578	0.132	NA	-0.534	0.600	NA	1.354	0.193	NA
Arm 3	1S-*t* test	Normal				-1.338	0.186	NA	-0.772	0.450	NA	-0.312	0.759	NA	-1.262	0.223	NA
																	
						Genotype			Cohort			Genotype × cohort			Pairwise comparisons		
	Test	Data structure	WT	Het	KO	*F*	*p* value	Power	*F*	*p* value	Power	*F*	*p* value	Power	WT vs Het	WT vs KO	Het vs KO
Total number of choices	2wANOVA	Normal	43.42 ± 3.25	40.26 ± 2.54	38.47 ± 2.75	0.612	0.546	0.147	0.164	0.687	0.068	2.244	0.116	0.437	−	−	-
Number of correct choice	2wANOVA	Normal	57.46 ± 1.59	60.68 ± 1.93	57.52 ± 1.61	1.227	0.302	0.256	2.987	*0.090*	0.396	0.927	0.402	0.202	−	−	-
Number of type 1 errors	2wANOVA	Normal	37.8 ± 1.43	34.09 ± 1.59	38.29 ± 1.79	2.296	0.111	0.445	0.300	0.586	0.084	5.165	**0.009**	0.804	−	−	-
Number of type 2 errors	2wANOVA	Nonnormal	4.04 ± 1.11	5.47 ± 1.02	4.51 ± 1.07	0.397	0.674	0.111	4.402	**0.041**	0.539	3.449	**0.039**	0.621	−	−	-
																	
**Fear conditioning**	****	****	****	****	****	****	****	****	****	****	****	****	****	****	****	****	****
Training	Test	Data structure				*F*	*p* value	Power	WT vs Het	WT vs KO	Het vs KO						
- Time	rMeasures	Sph.viol				43.998	**0.000**	1.000	−	−	−						
- Time × genotype	rMeasures	Sph.viol				3.194	**0.002**	0.970	−	−	−						
- Genotype	rMeasures	Sph.viol				14.505	**0.000**	0.998	0.809	**0.000**	**0.000**						
- Cohort	rMeasures	Sph.viol				12.351	**0.001**	0.932	−	−	−						
- Time × gen. × coh.	rMeasures	Sph.viol				0.602	0.782	0.281	−	−	−						
- Gen. × coh.	rMeasures	Sph.viol				0.494	0.613	0.127	−	−	−						
																	
						Genotype			Cohort			Genotype × cohort			Pairwise comparisons		
Training, individualtime bins	Test	Data structure	WT	Het	KO	*F*	*p*value	Power	*F*	*p*value	Power	*F*	*p*value	Power	WT vs Het	WT vs KO	Het vsKO
Habituation	2wANOVA	Nonnormal	16.85 ± 2.93	10.45 ± 2.37	21.6 ± 5.3	2.081	0.135	0.408	0.015	0.903	0.052	0.061	0.941	0.059	−	−	-
Pre-tone 0-120	2wANOVA	Nonnormal	10.45 ± 1.72	8.15 ± 1.43	21.01 ± 3.97	6.546	**0.003**	0.892	0.330	0.568	0.087	0.041	0.960	0.056	0.820	**0.021**	**0.004**
Tone/shock 120-140	2wANOVA	Nonnormal	10.58 ± 3.26	6.85 ± 2.82	19.94 ± 6.06	2.361	0.105	0.456	0.020	0.887	0.052	0.199	0.820	0.079	−	−	-
Post-tone 140-260	2wANOVA	Nonnormal	19.74 ± 3.73	18.83 ± 3.86	47.68 ± 6.71	13.149	**0.000**	0.996	5.506	**0.023**	0.634	2.222	0.119	0.433	0.990	**0.000**	**0.000**
Tone/shock 260-280	2wANOVA	Nonnormal	15.07 ± 4.7	24.58 ± 5.22	47.23 ± 7.47	7.613	**0.001**	0.934	0.026	0.871	0.053	0.762	0.472	0.173	0.507	**0.001**	**0.027**
Tone/shock 260-280	2wANOVA	Nonnormal	31.06 ± 5.14	37.88 ± 6.6	65.23 ± 6.72	12.505	**0.000**	0.995	18.006	**0.000**	0.986	0.640	0.532	0.151	0.650	**0.000**	**0.002**
Tone/shock 400-420	2wANOVA	Nonnormal	31.03 ± 5.86	40.59 ± 7.61	65.21 ± 5.94	9.728	**0.000**	0.977	12.565	**0.001**	0.935	0.061	0.941	0.059	0.503	**0.001**	**0.015**
Post-tone 420-540	2wANOVA	Nonnormal	36.71 ± 6.53	47.61 ± 7.36	61.74 ± 6.78	7.880	**0.001**	0.942	45.207	**0.000**	1.000	0.135	0.874	0.070	0.303	**0.003**	0.139
																	
Context	Test	Data structure				*F*	*p* value	Power	WT vs Het	WT vs KO	Het vs KO						
- Time	rMeasures	Sph.ass				4.558	**0.004**	0.880	−	−	−						
- Time × genotype	rMeasures	Sph.ass				0.675	0.670	0.262	−	−	−						
- Genotype	rMeasures	Sph.ass				1.788	0.178	0.357	−	−	−						
- Cohort	rMeasures	Sph.ass				0.542	0.465	0.112	−	−	−						
- Time × gen.× coh.	rMeasures	Sph.ass				0.918	0.481	0.355	−	−	−						
- Gen. × coh.	rMeasures	Sph.ass				1.026	0.366	0.219	−	−	−						
																	
						Genotype			Cohort			Genotype × cohort			Pairwise comparisons		
Context, individual time bins	Test	Datastructure	WT	Het	KO	*F*	*p* value	Power	*F*	*p*value	Power	*F*	*p* value	Power	WT vs Het	WT vs KO	Het vs KO
0-60	2wANOVA	Nonnormal	63.09 ± 5.13	57.19 ± 5.63	44.3 ± 6.21	2.643	0.081	0.502	0.582	0.449	0.116	0.558	0.576	0.137	0.750	0.063	0.261
60-120	2wANOVA	Nonnormal	66.34 ± 6.83	66 ± 6.94	59.6 ± 7.41	0.230	0.795	0.084	0.676	0.415	0.127	2.233	0.118	0.435	−	−	-
120-180	2wANOVA	Nonnormal	62.65 ± 7.14	62.11 ± 6.47	42.81 ± 7.83	2.263	0.114	0.440	0.392	0.534	0.094	0.958	0.390	0.207	−	−	-
180-240	2wANOVA	Nonnormal	56.12 ± 6.56	54.45 ± 7.58	43.33 ± 6.29	0.944	0.396	0.205	0.073	0.788	0.058	0.109	0.897	0.066	−	−	-
mean	2wANOVA	Nonnormal	62.05 ± 5.57	59.94 ± 5.31	47.51 ± 5.84	1.788	0.178	0.357	0.542	0.465	0.112	1.026	0.366	0.219	−	−	-
																	
Cued	Test	Data structure				*F*	*p* value	Power	WT vs Het	WT vs KO	Het vs KO						
- Time	rMeasures	Sph.viol				25.753	**0.000**	1.000	−	−	−						
- Time × genotype	rMeasures	Sph.viol				3.101	**0.002**	0.968	−	−	−						
- Genotype	rMeasures	Sph.viol				5.657	**0.006**	0.841	0.645	**0.007**	0.065						
- Cohort	rMeasures	Sph.viol				4.255	**0.044**	0.525	−	−	−						
- Time × gen. × coh.	rMeasures	Sph.viol				4.116	**0.000**	0.995	−	−	−						
- Gen. × coh.	rMeasures	Sph.viol				1.616	0.209	0.326	−	−	−						

						Genotype			Cohort			Genotype × cohort			Pairwise comparisons		
Cued, individual time bins	Test	Data structure	WT	Het	KO	*F*	*p* value	Power	*F*	*p* value	Power	*F*	*p* value	Power	WT vs Het	WT vs KO	Het vsKO
Pre-tone 0-60	2wANOVA	Nonnormal	1.64 ± 1.14	0.4 ± 0.28	4.9 ± 2.59	1.897	0.160	0.376	5.996	**0.018**	0.671	1.527	0.227	0.310	0.841	0.311	0.114
Pre-tone 60-120	2wANOVA	Nonnormal	0.84 ± 0.38	1.96 ± 0.89	2.37 ± 0.88	1.056	0.355	0.225	18.576	**0.000**	0.988	0.747	0.479	0.170	0.461	0.238	0.896
Tone 120-140	2wANOVA	Nonnormal	10.94 ± 4.37	12.14 ± 4.53	25.23 ± 6.38	3.005	*0.058*	0.558	7.144	**0.010**	0.746	1.298	0.282	0.268	0.984	0.106	0.150
Post-tone 140-200	2wANOVA	Nonnormal	8.52 ± 2.11	7.41 ± 1.76	13.09 ± 3.74	1.367	0.264	0.281	1.610	0.210	0.238	0.118	0.889	0.067	−	−	-
Post-tone 200-260	2wANOVA	Nonnormal	2.29 ± 1.03	6.59 ± 1.53	10.03 ± 4.8	1.551	0.222	0.314	1.595	0.212	0.236	1.203	0.309	0.251	−	−	-
Tone 260-280	2wANOVA	Nonnormal	13.87 ± 5.15	19.36 ± 5.92	39.08 ± 7.75	7.219	**0.002**	0.921	15.352	**0.000**	0.970	7.888	**0.001**	0.942	0.733	**0.003**	**0.025**
Post-tone 280-340	2wANOVA	Nonnormal	8.92 ± 2.71	19.61 ± 5.1	26.7 ± 6.01	3.891	**0.027**	0.677	5.448	**0.024**	0.629	1.180	0.316	0.247	0.240	**0.024**	0.527
Post-tone 340-400	2wANOVA	Nonnormal	5.09 ± 1.34	9.96 ± 2.78	20.99 ± 4.81	6.189	**0.004**	0.874	1.570	0.216	0.233	1.068	0.351	0.227	0.549	**0.003**	*0.054*

WT, wild-type mice; Het, heterozygous mice; KO, homozygous knock-out mice. Group values are reported as mean ± SEM. Bold font indicates significant results (*p* < 0.05). Individual results and statistical analyses for cohorts 1 and 2 are available in Extended Data Table 12-1. 2wANOVA: 2-way ANOVA, rMeasures: repeated measures, one sample t test: 1S-t test, Norm: normal, No-norm: non-normal, Sph.ass: sphericity assumed, Sph.viol: sphericity violated, gen: genotype, coh: cohort. 2wANOVA: 2-way ANOVA, rMeasures: repeated measures, one sample t test: 1S-t test, Sph.ass: sphericity assumed, Sph.viol: sphericity violated, gen: genotype, coh: cohort.

10.1523/ENEURO.0046-18.2018.t12-1 Extended Data Table 12-1 **Individual results and statistical analyses for cohorts 1 and 2 related to learning and memory.** WT, wild-type mice; Het, heterozygous mice; KO, homozygous knockout mice. Group values are reported as means ± s.e.m. Red font indicates significant results (p < 0.05), orange font indicates trends (0.1 < p < 0.05). Download Table 12-1, DOCX file.

**Figure 9.**
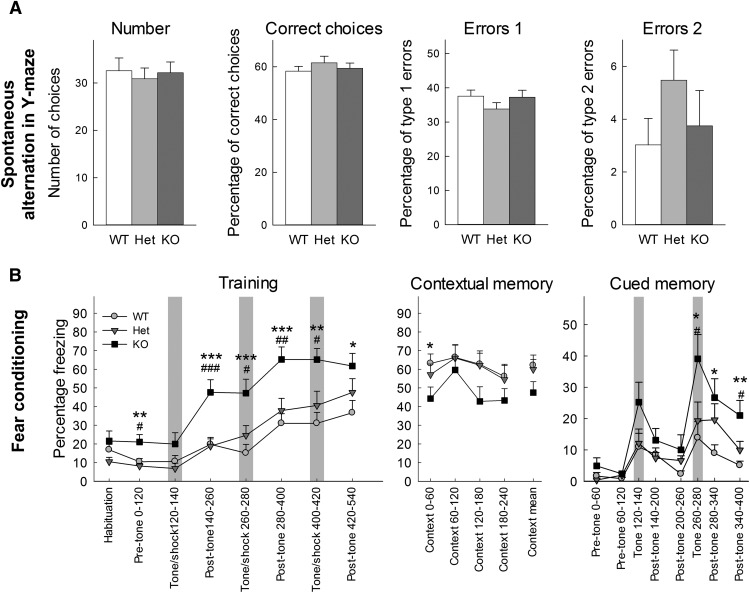
Learning and memory in *Shank3^Δ4-22^-*deficient mice. ***A***, Working memory in Y-maze measured by spontaneous alternation behavior. All genotypes showed comparable number of arm choices, percentage of correct choices (three-way alternation), type 1 error (three consecutive choices where the first and third choices are identical), or type 2 error (three consecutive choices where the second and third choices are identical). ***B***, Contextual and cued fear conditioning in *Shank3* mice. A higher percentage of freezing was observed in *Shank3^Δ4-22^* homozygous mice compared to wild-type and heterozygous animals on day 1. While the difference was already present before the sound-shocks associations, it was strongly increased posttraining. No genotype differences were detected in freezing scores in the posttraining session on day 1. Opposite results were observed for contextual conditioning (day 2) and cued conditioning (day 3): *Shank3^Δ4-22^* homozygous mice showed an impairment of contextual learning compared to their wild-type and heterozygous littermates but an enhancement of freezing postcues during the cued testing. WT, wild-type mice; Het, heterozygous mice; KO, homozygous knock-out mice. *: WT versus KO; #: Het versus KO. *p < 0.05, **p < 0.1, ***p < 0.001.

When looking at the spontaneous alternation behavior in the Y-maze, no differences were observed between the genotypes in any of the background strains regarding either the total number of choices, the percentage of correct choices or the percentage of errors (Fig. 9*A*). Moreover, no arm preference was seen for any of the groups.

In the training session of the fear conditioning test, minimal levels of freezing behavior were seen for all the genotypes during the 5-min habituation period; however, while this percentage of spontaneous freezing decreased before the presentations of cue-shock pairings for the *Shank3^Δ4-22^* wild-type and heterozygotes, it remained at significantly higher level for *Shank3^Δ4-22^* homozygous mice. A significant genotype effect was then found during the training session in postshock freezing, with *Shank3^Δ4-22^* homozygous mice displaying higher levels of freezing compared with wild-type and heterozygous mice (Fig. 9*B*, left panel). The opposite was observed during contextual recall where even if all the mice freeze significantly more than during the habituation of the training sessions a trend toward a decrease (significant during the first minute) of freezing was observed for *Shank3^Δ4-22^* homozygous mice compared to wild-type or heterozygous littermates (Fig. 9*B*, middle panel). An increase of freezing was seen in both during and after the cue presentation (trend for the first cue, significant during and after the second cue) *Shank3^Δ4-22^* homozygous mice (Fig. 9*B*, right panel).

### Anxiety-related behaviors in *Shank3^Δ4-22^*-deficient mice

Anxiety-like behaviors were monitored in the open field and in the elevated zero-maze, and detailed results are displayed in Table 13.

**Table 13.** Detailed results and statistical analyses related to anxiety-like behaviors

**Open field thigmotaxis**																	
						Genotype			Cohort			Genotype × cohort			Pairwise comparisons		
	Test	Data structure	WT	Het	KO	*F*	*p* value	Power	*F*	*p* value	Power	*F*	*p* value	Power	WT vs Het	WT vs KO	Het vs KO
Border distance (cm)	2wANOVA	Normal	10507.07 ± 558.17	8848.23 ± 548.19	7942.17 ± 394.99	6.537	**0.003**	0.892	10.443	**0.002**	0.887	0.080	0.923	0.062	**0.022**	**0.001**	0.235
																	
Border distance (cm)	Test	Data structure				*F*	*p* value	Power	WT vs Het	WT vs KO	Het vsKO						
- Time	rMeasures	Sph.viol				52.599	**0.000**	1.000	−	−	−						
- Time × genotype	rMeasures	Sph.viol				2.496	**0.007**	0.877	−	−	−						
- Genotype	rMeasures	Sph.viol				6.537	**0.003**	0.892	**0.043**	**0.001**	0.373						
- Cohort	rMeasures	Sph.viol				10.443	**0.002**	0.887	−	−	−						
- Time × gen. × coh.	rMeasures	Sph.viol				0.923	0.492	0.399	−	−	−						
- Genotype × cohort	rMeasures	Sph.viol				0.080	0.923	0.062	−	−	−						
																	
						Genotype			Cohort			Genotype × cohort			Pairwise comparisons		
	Test	Data structure	WT	Het	KO	*F*	*p* value	Power	*F*	*p* value	Power	*F*	*p* value	Power	WT vs Het	WT vs KO	Het vs KO
Center distance (cm)	2wANOVA	Nonnormal	3276.58 ± 335.66	2390.56 ± 291.66	2139.7 ± 246.94	3.932	**0.026**	0.682	9.890	**0.003**	0.870	0.049	0.952	0.057	**0.036**	**0.011**	0.622
																	
Center distance (cm)	Test	Data structure				*F*	*p* value	Power	WT vs Het	WT vs KO	Het vs KO						
- Time	rMeasures	Sph.viol				1.158	0.330	0.343	−	−	−						
- Time × genotype	rMeasures	Sph.viol				1.327	0.237	0.571	−	−	−						
- Genotype	rMeasures	Sph.viol				3.932	**0.026**	0.682	*0.070*	**0.015**	0.798						
- Cohort	rMeasures	Sph.viol				9.890	**0.003**	0.870	−	−	−						
- Time × gen. × coh.	rMeasures	Sph.viol				0.695	0.683	0.302	−	−	−						
- Genotype × cohort	rMeasures	Sph.viol				0.049	0.952	0.057	−	−	−						
														
						Genotype			Cohort			Genotype × cohort			Pairwise comparisons		
	Test	Data structure	WT	Het	KO	*F*	*p* value	Power	*F*	*p* value	Power	*F*	*p* value	Power	WT vsHet	WT vs KO	Het vs KO
Border/total distance	2wANOVA	Normal	76.87 ± 1.51	79.57 ± 1.69	79.69 ± 1.29	0.950	0.393	0.206	5.570	**0.022**	0.639	0.049	0.952	0.057	−	−	−
																	
Border/total distance	Test	Data structure				*F*	*p* value	Power	WT vs Het	WT vsKO	Het vs KO						
- Time	rMeasures	Sph.viol				5.035	**0.001**	0.957	−	−	−						
- Time × genotype	rMeasures	Sph.viol				1.182	0.312	0.531	−	−	−						
- Genotype	rMeasures	Sph.viol				1.017	0.369	0.218	−	−	−						
- Cohort	rMeasures	Sph.viol				5.820	**0.019**	0.658	−	−	−						
- Time × gen. × coh.	rMeasures	Sph.viol				0.679	0.705	0.305	−	−	−						
- Genotype × cohort	rMeasures	Sph.viol				0.094	0.911	0.064	−	−	−						
														
						Genotype			Cohort			Genotype × cohort			Pairwise comparisons		
	Test	Data structure	WT	Het	KO	*F*	*p* value	Power	*F*	*p* value	Power	*F*	*p* value	Power	WT vs Het	WT vs KO	Het vsKO
Center/total distance	2wANOVA	Normal	22.88 ± 1.51	20.15 ± 1.68	20.16 ± 1.27	0.939	0.398	0.204	4.471	**0.039**	0.546	0.048	0.953	0.057	−	−	−
																	
Center/total distance	Test	Data structure				*F*	*p* value	Power	WT vs Het	WT vs KO	Het vsKO						
- Time	rMeasures	Sph.viol				5.177	**0.001**	0.962	−	−	−						
- Time × genotype	rMeasures	Sph.viol				1.177	0.315	0.527	−	−	−						
- Genotype	rMeasures	Sph.viol				1.001	0.375	0.215	−	−	−						
- Cohort	rMeasures	Sph.viol				4.757	**0.034**	0.571	−	−	−						
- Time × gen. × coh.	rMeasures	Sph.viol				0.652	0.728	0.292	−	−	−						
- Genotype × cohort	rMeasures	Sph.viol				0.088	0.916	0.063	−	−	−						
														
						Genotype			Cohort			Genotype × cohort			Pairwise comparisons		
	Test	Data structure	WT	Het	KO	*F*	*p* value	Power	*F*	*p* value	Power	*F*	*p* value	Power	WT vs Het	WT vs KO	Het vs KO
Border/Center distance	2wANOVA	Nonnormal	3.78 ± 0.38	4.87 ± 0.74	4.34 ± 0.36	0.990	0.379	0.213	3.522	*0.066*	0.453	0.216	0.807	0.082	−	−	−
																	
Border/Center distance	Test	Data structure				*F*	*p*value	Power	WT vs Het	WT vs KO	Het vsKO						
- Time	rMeasures	Sph.viol				5.177	**0.001**	0.240	−	−	−						
- Time × genotype	rMeasures	Sph.viol				1.177	0.315	0.456	−	−	−						
- Genotype	rMeasures	Sph.viol				1.001	0.375	0.309	−	−	−						
- Cohort	rMeasures	Sph.viol				4.757	**0.034**	0.469	−	−	−						
- Time × gen. × coh.	rMeasures	Sph.viol				0.652	0.728	0.196	−	−	−						
- Genotype × cohort	rMeasures	Sph.viol				0.088	0.916	0.230	−	−	−						
																	
						Genotype			Cohort			Genotype × cohort			Pairwise comparisons		
	Test	Data structure	WT	Het	KO	*F*	*p* value	Power	*F*	*p*value	Power	*F*	*p*value	Power	WT vs Het	WT vsKO	Het vs KO
Border time (s)	2wANOVA	Normal	2969.88 ± 70.88	2993.1 ± 77.46	3067.4 ± 62.2	0.481	0.621	0.124	1.088	0.302	0.176	0.582	0.563	0.141	−	−	−

Border time (s)	Test	Data structure				*F*	*p* value	Power	WT vs Het	WT vs KO	Het vsKO						
- Time	rMeasures	Sph.viol				2.960	**0.023**	0.773	−	−	−						
- Time × genotype	rMeasures	Sph.viol				0.836	0.568	0.374	−	−	−						
- Genotype	rMeasures	Sph.viol				0.481	0.621	0.124	−	−	−						
- Cohort	rMeasures	Sph.viol				1.088	0.302	0.176	−	−	−						
- Time × gen. × coh.	rMeasures	Sph.viol				0.792	0.606	0.354	−	−	−						
- Genotype × cohort	rMeasures	Sph.viol				0.582	0.563	0.141	−	−	−						
														
						Genotype			Cohort			Genotype × cohort			Pairwise comparisons		
	Test	Data structure	WT	Het	KO	*F*	*p* value	Power	*F*	*p*value	Power	*F*	*p* value	Power	WT vs Het	WT vs KO	Het vsKO
Center time (s)	2wANOVA	Normal	612.08 ± 71.17	587.82 ± 77.5	517.59 ± 62.2	0.451	0.640	0.119	0.871	0.355	0.150	0.589	0.559	0.143	−	−	−
																	
Center time (s)	Test	Data structure				*F*	*p* value	Power	WT vs Het	WT vs KO	Het vsKO						
- Time	rMeasures	Sph.viol				3.200	**0.016**	0.807	−	−	−						
- Time × genotype	rMeasures	Sph.viol				0.836	0.568	0.363	−	−	−						
- Genotype	rMeasures	Sph.viol				0.481	0.621	0.119	−	−	−						
- Cohort	rMeasures	Sph.viol				1.088	0.302	0.150	−	−	−						
- Time × gen. × coh.	rMeasures	Sph.viol				0.792	0.606	0.090	−	−	−						
- Genotype × cohort	rMeasures	Sph.viol				0.582	0.563	0.143	−	−	−						
														
						Genotype			Cohort			Genotype × cohort			Pairwise comparisons		
	Test	Data structure	WT	Het	KO	*F*	*p* value	Power	*F*	*p* value	Power	*F*	*p* value	Power	WT vs Het	WT vs KO	Het vsKO
Border/center time	2wANOVA	Nonnormal	6.95 ± 1.32	8.3 ± 1.88	9.76 ± 2.27	0.476	0.624	0.124	0.533	0.469	0.111	0.107	0.898	0.066	−	−	−
																	
Border/center time	Test	Data structure				*F*	*p* value	Power	WT vs Het	WT vs KO	Het vsKO						
- Time	rMeasures	Sph.viol				0.290	0.822	0.103	−	−	−						
- Time × genotype	rMeasures	Sph.viol				1.575	0.163	0.575	−	−	−						
- Genotype	rMeasures	Sph.viol				0.429	0.653	0.116	−	−	−						
- Cohort	rMeasures	Sph.viol				1.546	0.220	0.230	−	−	−						
- Time × gen. × coh.	rMeasures	Sph.viol				0.575	0.740	0.219	−	−	−						
- Genotype × cohort	rMeasures	Sph.viol				1.594	0.213	0.322	−	−	−						
																	
**Vertical activity in open field**																	
						Genotype			Cohort			Genotype × cohort			Pairwise comparisons		
	Test	Data structure	WT	Het	KO	*F*	*p* value	Power	*F*	*p* value	Power	*F*	*p* value	Power	WT vs Het	WT vs KO	Het vsKO
Free rears duration (s)	2wANOVA	Nonnormal	4.66 ± 1.25	7.93 ± 1.62	6.18 ± 1.48	1.159	0.322	0.243	0.036	0.850	0.054	1.480	0.237	0.301	−	−	−
Free rears number	2wANOVA	Nonnormal	8.42 ± 2.13	10.63 ± 1.66	7.57 ± 1.21	0.837	0.439	0.186	1.988	0.165	0.283	1.369	0.264	0.281	−	−	−
Wall rears duration (s)	2wANOVA	Nonnormal	14.68 ± 1.77	16.13 ± 2.02	9.01 ± 0.84	5.023	**0.010**	0.793	1.924	0.171	0.275	0.397	0.675	0.111	0.805	**0.045**	**0.009**
Wall rears number	2wANOVA	Nonnormal	27.26 ± 2.68	27.52 ± 2.86	19.36 ± 1.88	3.576	**0.035**	0.638	19.306	0.000	0.991	0.414	0.663	0.113	0.996	**0.036**	**0.030**
All rears duration (s)	2wANOVA	Nonnormal	19.34 ± 2.3	24.07 ± 3.01	15.2 ± 1.99	3.140	*0.052*	0.578	0.646	0.425	0.124	1.240	0.298	0.258	0.374	0.468	**0.038**
All rears number	2wANOVA	Nonnormal	35.68 ± 3.83	38.15 ± 3.94	26.94 ± 2.08	3.240	**0.047**	0.592	16.104	0.000	0.976	1.267	0.290	0.263	0.829	0.107	**0.028**
																	
**Zero-maze**	****	****	****	****	****	****	****	****	****	****	****	****	****	****	****	****	****
						Genotype			Cohort			Genotype ×cohort			Pairwise comparisons		
	Test	Datastructure	WT	Het	KO	*F*	*p* value	Power	*F*	*p* value	Power	*F*	*p* value	Power	WT vs Het	WT vs KO	Het vs KO
Closed arc time, d1	2wANOVA	Normal	428.66 ± 18.61	441.68 ± 13.82	456.86 ± 18.31	0.400	0.672	0.111	13.684	**0.001**	0.952	0.311	0.734	0.097	−	−	−
Closed arc time, d2	2wANOVA	Nonnormal	448.33 ± 20.43	492.29 ± 16.58	487.59 ± 29.63	3.652	**0.033**	0.646	36.459	**0.000**	1.000	0.114	0.893	0.066	0.138	**0.009**	0.489
Closed arc time, m	2wANOVA	Normal	438.5 ± 18.27	466.99 ± 13.34	472.23 ± 20.15	1.917	0.158	0.379	28.873	**0.000**	1.000	0.253	0.778	0.088	−	−	−
Open arc time, d1	2wANOVA	Normal	166.03 ± 18.25	153.09 ± 13.6	134.72 ± 17.99	0.562	0.574	0.138	10.417	**0.002**	0.886	0.420	0.659	0.114	−	−	−
Open arc time, d2	2wANOVA	Nonnormal	143.08 ± 21.29	102.14 ± 16.74	78.39 ± 17.83	3.063	*0.056*	0.566	37.315	**0.000**	1.000	0.151	0.860	0.072	0.194	**0.015**	0.502
Open arc time, m	2wANOVA	Normal	154.55 ± 18.53	127.61 ± 13.25	106.56 ± 16.4	1.863	0.166	0.369	26.343	**0.000**	0.999	0.340	0.713	0.101	−	−	−
Close/open time, d1	2wANOVA	Nonnormal	4.08 ± 0.85	3.74 ± 0.98	5.76 ± 1.6	0.820	0.447	0.182	9.172	**0.004**	0.843	0.438	0.648	0.117	−	−	−
Close/open time, d2	2wANOVA	Nonnormal	6.95 ± 1.95	25.36 ± 18.5	28.98 ± 11.02	0.969	0.387	0.209	5.901	**0.019**	0.663	0.746	0.479	0.169	−	−	−
Close/open time, m	2wANOVA	Nonnormal	4.61 ± 1	5.55 ± 1.95	8.57 ± 2.42	1.207	0.308	0.251	11.378	**0.001**	0.911	0.538	0.587	0.134	−	−	−
Open arc entries, d1	2wANOVA	Nonnormal	48.47 ± 4.88	50.52 ± 4.46	59.36 ± 7.73	1.870	0.165	0.371	14.588	**0.000**	0.963	0.603	0.551	0.145	−	−	−
Open arc entries, d2	2wANOVA	Normal	42.42 ± 5.18	36.52 ± 4.83	29.31 ± 5.07	1.238	0.299	0.257	19.130	**0.000**	0.990	0.137	0.872	0.070	−	−	−
Open arc entries, m	2wANOVA	Normal	45.44 ± 4.16	43.52 ± 4.04	44.34 ± 5.81	0.150	0.861	0.072	24.720	**0.000**	0.998	0.447	0.642	0.119	−	−	−
Open entering arc latency	2wANOVA	Nonnormal	39.29 ± 13.16	47.08 ± 31.35	22.99 ± 10.38	0.310	0.735	0.097	0.684	0.412	0.128	1.282	0.287	0.265	−	−	−
Open arc crossing latency	2wANOVA	Nonnormal	149.34 ± 34.87	139.69 ± 35.2	95.2 ± 32.99	0.796	0.457	0.178	1.515	0.224	0.226	0.395	0.676	0.110	−	−	−
Close arc dipping number, d1	2wANOVA	Nonnormal	51.73 ± 6.81	55.42 ± 6.48	51.47 ± 7.31	0.303	0.740	0.096	54.807	**0.000**	1.000	0.035	0.965	0.055	−	−	−
Close arc dipping number, d2	2wANOVA	Nonnormal	31.94 ± 3.01	28.94 ± 3.69	22.21 ± 3.87	2.182	0.124	0.425	54.920	**0.000**	1.000	0.593	0.556	0.143	−	−	−
Close arc dipping number, m	2wANOVA	Nonnormal	41.84 ± 4.31	42.5 ± 4.48	36.84 ± 5.05	0.239	0.788	0.085	94.671	**0.000**	1.000	0.179	0.836	0.076	−	−	−
Close arc dipping time, d1	2wANOVA	Nonnormal	131.15 ± 14.57	145.82 ± 14.24	117.88 ± 13.74	0.984	0.381	0.211	57.892	**0.000**	1.000	0.648	0.527	0.153	−	−	−
Close arc dipping time, d2	2wANOVA	Nonnormal	94.51 ± 10	100.08 ± 13.98	64.33 ± 12.46	2.111	0.132	0.413	26.004	**0.000**	0.999	0.342	0.712	0.102	−	−	−
Close arc dipping time, m	2wANOVA	Normal	112.83 ± 11.57	124.49 ± 12.37	91.1 ± 10.18	2.386	0.103	0.459	72.828	**0.000**	1.000	0.811	0.450	0.181	−	−	−
Open arc dipping number, d1	2wANOVA	Nonnormal	27.15 ± 3.63	20.26 ± 2.46	19.15 ± 3.92	1.441	0.247	0.294	8.140	**0.006**	0.799	0.567	0.571	0.139	−	−	−
Open arc dipping number, d2	2wANOVA	Nonnormal	16.94 ± 3.04	12.22 ± 2.34	8.84 ± 2.54	1.938	0.155	0.383	17.131	**0.000**	0.982	0.332	0.719	0.100	−	−	−
Open arc dipping number, m	2wANOVA	Nonnormal	22.05 ± 3.15	16.97 ± 2.15	14 ± 2.9	2.060	0.138	0.404	15.216	**0.000**	0.969	0.502	0.609	0.128	−	−	−

**Zero-maze**	****	****	****	****	****	****	****	****	****	****	****	****	****	****	****	****	****
						Genotype			Cohort			Genotype ×cohort			Pairwise comparisons		
	Test	Datastructure	WT	Het	KO	*F*	*p* value	Power	*F*	*p* value	Power	*F*	*p* value	Power	WT vs Het	WT vs KO	Het vs KO
Open arc dipping time, d1	2wANOVA	Nonnormal	62.85 ± 10.83	42.28 ± 5.15	25.57 ± 5.27	5.700	**0.006**	0.843	13.928	**0.000**	0.955	1.730	0.188	0.346	*0.091*	**0.001**	0.245
Open arc dipping time, d2	2wANOVA	Nonnormal	51.3 ± 10.52	35.3 ± 8.44	17.87 ± 5.34	3.798	**0.029**	0.665	20.104	**0.000**	0.993	0.427	0.655	0.115	0.278	**0.008**	0.269
Open arc dipping time, m	2wANOVA	Nonnormal	57.07 ± 9.63	39.17 ± 5.86	21.72 ± 4.71	6.448	**0.003**	0.886	23.422	**0.000**	0.997	1.299	0.282	0.268	*0.088*	**0.001**	0.157
																	
Time open vs close, d1	Test	Data structure				All *F*	All *p* value	Power	WT *F*	WT *p* value	Power	Het *F*	Het *p* value	Power	KO *F*	KO *p* value	Power
- Zone	rMeasures	Sph.ass				277.319	**0.000**	1.000	73.861	**0.000**	1.000	125.301	**0.000**	1.000	83.255	**0.000**	1.000
- Cohort	rMeasures	Sph.ass				8.156	**0.006**	0.935	5.563	**0.031**	0.604	8.257	**0.011**	0.773	3.298	*0.087*	0.403
- Zone × cohort	rMeasures	Sph.ass				12.518	**0.001**	0.801	7.248	**0.015**	0.718	3.063	*0.098*	0.379	2.143	0.161	0.282
																	
Time open vs close, d2	Test	Data structure				All *F*	All *p* value	Power	WT *F*	WT *p* value	Power	Het *F*	Het *p* value	Power	KO *F*	KO *p* value	Power
- Zone	rMeasures	Sph.ass				440.281	**0.000**	1.000	94.767	**0.000**	1.000	278.317	**0.000**	1.000	119.843	**0.000**	1.000
- Cohort	rMeasures	Sph.ass				0.578	0.450	0.218	1.269	0.276	0.186	2.497	0.132	0.320	0.921	0.351	0.148
- Zone × cohort	rMeasures	Sph.ass				25.848	**0.000**	1.000	12.054	**0.003**	0.905	18.624	**0.000**	0.982	3.749	*0.070*	0.447
																	
Time open vs close, m	Test	Data structure				All *F*	All *p* value	Power	WT *F*	WT *p*value	Power	Het *F*	Het *p*value	Power	KO *F*	KO *p*value	Power
- Zone	rMeasures	Sph.ass				440.281	**0.000**	1.000	103.409	**0.000**	1.000	274.392	**0.000**	1.000	131.582	**0.000**	1.000
- Cohort	rMeasures	Sph.ass				0.578	0.450	0.116	0.006	0.941	0.051	0.422	0.524	0.094	0.484	0.496	0.101
- Zone × cohort	rMeasures	Sph.ass				25.848	**0.000**	0.999	11.720	**0.003**	0.897	12.720	**0.002**	0.919	3.786	*0.068*	0.451

WT, wild-type mice; Het, heterozygous mice; KO, homozygous knock-out mice. Group values are reported as mean ± SEM. Bold font indicates significant results (*p* < 0.05). Individual results and statistical analyses for cohorts 1 and 2 are available in Extended Data Table 13-1. 2wANOVA: 2-way ANOVA, rMeasures: repeated measures, Norm: normal, No-norm: non-normal, Sph.ass: sphericity assumed, Sph.viol: sphericity violated, gen: genotype, coh: cohort, d1: day 1, d2: day 2, m: day 1 - day 2 mean. 2wANOVA: 2-way ANOVA, rMeasures: repeated measures, Sph.ass: sphericity assumed, Sph.viol: sphericity violated, gen: genotype, coh: cohort, d1: day 1, d2: day 2, m: day 1 - day 2 mean.

10.1523/ENEURO.0046-18.2018.t13-1 Extended Data Table 13-1 **Individual results and statistical analyses for cohorts 1 and 2 related to anxiety-like behaviors.** WT, wild-type mice; Het, heterozygous mice; KO, homozygous knockout mice. Group values are reported as means ± s.e.m. Red font indicates significant results (p < 0.05), orange font indicates trends (0.1 < p < 0.05). Download Table 13-1, DOCX file.

**Figure 10.**
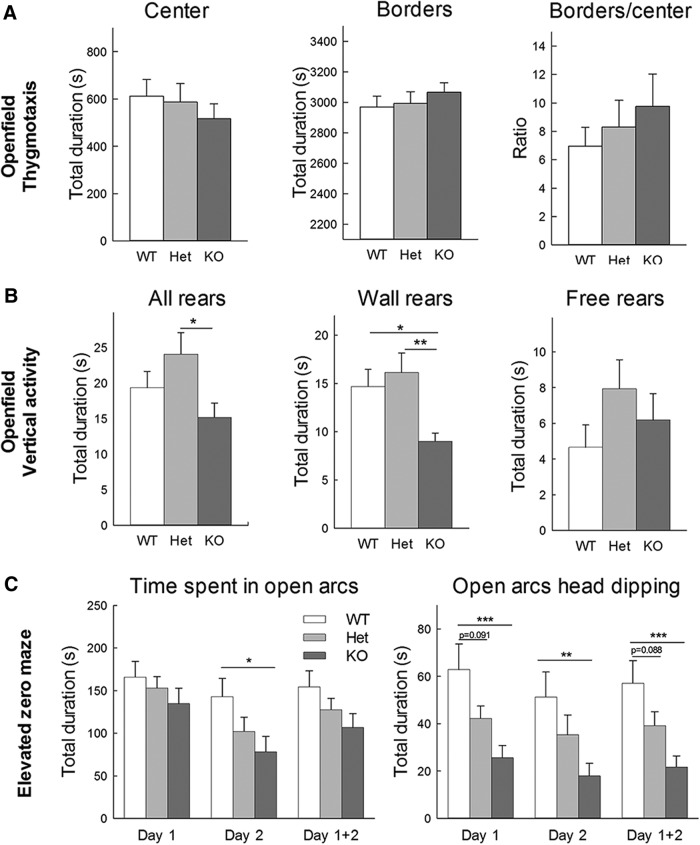
Anxiety-like behavior in *Shank3^Δ4-22^-*deficient mice. ***A***, Thigmotaxic behavior in open field. No genotype differences were found for the time spent in the center of the open field, the time spent close to the chamber walls (borders), or their ratio. ***B***, Vertical activity in open field. The cumulated time spend in free standing rears and rears against the walls of the open field were both counted. When compared to wild-type and heterozygotes littermates, *Shank3^Δ4-22^* homozygous mice displayed decreased rearing activity due to a decrease of wall rears rather than free standing rears. ***C***, *Shank3^Δ4-22^* homozygous mice spent a lower amount of time in the open area when compared to wild-type and heterozygous mice. Similarly, the number of head dipping from the open arcs to the outside of the maze was reduced in *Shank3^Δ4-22^* homozygous mice. WT, wild-type mice; Het, heterozygous mice; KO, homozygous knock-out mice. *p < 0.05, **p < 0.1, ***p < 0.001.

No significant difference between the genotypes was observed in the open field thigmotaxis level (Fig. 10*A*), but a decrease in the total number of times the mice reared (mainly driven by against wall rears) was observed in the *Shank3^Δ4-22^* homozygous animals (Fig. 10*B*). No significant effects of an interaction between the time and genotype were observed for any of the parameters.

In the elevated zero-maze, all animals showed a preference for the closed arcs versus the open arcs; however, *Shank3^Δ4-22^* homozygotes spent less time in the open arcs than their wild-type and heterozygous littermates. Similarly, a significant decrease of the duration of head dipping exploratory behavior in the open arcs was seen in those animals (Fig. 10*B*). No genotype differences were seen for other parameters.

This indicates increases in anxiety in the *Shank3^Δ4-22^* homozygotes. In support of this, the long-lasting spontaneous freezing observed in *Shank3^Δ4-22^* homozygous animals during the habituation and before the sound-shock association in the fear conditioning training (Fig. 9*B*) could also be explained by a higher anxiety level those animals.

## Discussion

**Figure 11.**
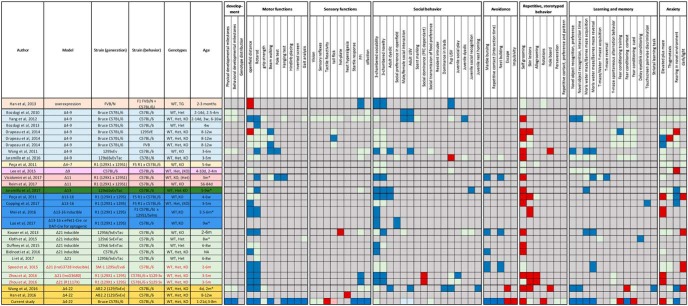
Main features and comorbidities associated with Phelan–McDermid displayed by different mouse models with *Shank3* deficits. Green indicates an absence of genotype difference. Blue indicates a decrease of the associated behavior in *Shank3*-deficient animals. Red indicates an increase of the associated behavior in *Shank3*-deficient animals. Gray indicates the behavior has not been studied in the corresponding article. Age column: d = days, w = weeks, m = months, * indicates that only the age at the beginning of the testing was provided.

Given the prevalence of complete *SHANK3* deletions in PMS, we generated *Shank3^Δ4–22^*mice by targeting exons 4-22, thereby disrupting all isoforms and providing improved construct validity compared to previously reported models. We conducted an extensive behavioral phenotyping of neonatal (P0–P21) and adult (three to eight months) mice to address both core symptoms and comorbidities observed in PMS. We confirmed our prediction that *Shank3* ^Δ4–22^ mice homozygous and in some instances heterozygous mice have a more severe phenotype than previously published models with partial deletions of *Shank3* (summarized in Fig. 11). Our findings are consistent with recent results from an independent model also generated by disrupting all *Shank3* isoforms (Wang et al., 2016b).

PMS is a neurodevelopmental disorder that manifests as early as in infancy by neonatal hypotonia and a generalized developmental delay. Previous studies have shown normal neonatal development in Δ4-9 mice (Bozdagi et al., 2010; Wang et al., 2011; Yang et al., 2012) or only minor delays limited to ear opening and paw positioning in Δ4-22 mice (Wang et al., 2016b). In the current study, both physical and behavioral developmental milestones were investigated. Physical delays were limited to a slower growth rate in *Shank3^Δ4–22^*-deficient animals. In addition, a non-Mendelian genotype distribution showing a deficit for *Shank3^Δ4–22^* homozygous mice was explained, at least partially, by an increased postnatal mortality observed in the *Shank3^Δ4–22^* mice homozygous animals. Similar non-Mendelian genotype distributions have been previously observed in other mouse and rat Shank3 models (Drapeau et al., 2014; Harony-Nicolas et al., 2017). As *Shank3* is known to be highly expressed in placenta (Beri et al., 2007), this suggests that *Shank3* deficiency could lead to placental insufficiency responsible for *in utero* developmental delays and increased perinatal mortality. Despite a slower growth curves during the first weeks of life, the weight of surviving homozygous animals is no longer different from their littermates when examined at three months of age, indicating a post birth correction, and survival curves between 2 and 22 months do not show any significant genotype difference.

Extensive sensory-motor deficits were observed in newborn *Shank3*-deficient mice. Some of them, such as the response to an auditory startle or the air righting ability, were only delayed, while others, such as performances in the wire suspension tests and the grasping reflex, were still present at the time of weaning. On home-cage observation and physical examination of adult mice we did not observe severe deficits that would preclude advanced testing.

Hypotonia, motor-coordination impairments and gait abnormalities are a hallmark of PMS that persists beyond childhood (Phelan and McDermid, 2012; Soorya et al., 2013). In previous studies, motor performances have been frequently found to be impaired in adult *Shank3-*deficient mice (Fig. 11). Hence, decreased locomotion in the open field has been reported in many existing models including models with Δ4-9, Δ13-16, Δ21 deletions, or point mutations (Yang et al., 2012; Kouser et al., 2013; Speed et al., 2015; Bidinosti et al., 2016; Mei et al., 2016; Zhou et al., 2016; Copping et al., 2017) even if not always replicated in other models with similar or different deletions (Δ4-9, Δ9, Δ13, Δ13-16, Δ21; Peça et al., 2011; Drapeau et al., 2014; Duffney et al., 2015; Lee et al., 2015; Jaramillo et al., 2016, 2017). Similarly, motor learning in accelerating rotarod was found to be impaired in Δ4-9, Δ11, Δ13, Δ13-16, and Δ21 models (Bozdagi et al., 2010; Wang et al., 2011; Yang et al., 2012; Kouser et al., 2013; Zhu et al., 2014; Speed et al., 2015; Mei et al., 2016; Jaramillo et al., 2017; Vicidomini et al., 2017) although not replicated in other studies (Δ4-9, Δ13-16, or Δ2; Peça et al., 2011; Drapeau et al., 2014; Duffney et al., 2015; Bidinosti et al., 2016; Jaramillo et al., 2016; Li et al., 2017). In agreement with Wang et al. (2011), both spontaneous locomotion and rotarod learning were strongly impaired in our *Shank3^Δ4–22^*mouse model. Interestingly, while most models only reported deficits in homozygous animals, heterozygous mice were also affected, albeit less severely. Difficulties in fine motor coordination have been described in Δ4-9 and Δ11 *Shank3-*deficient mice (Wang et al., 2011; Drapeau et al., 2014; Vicidomini et al., 2017) and were confirmed in the current study. In addition, our homozygous mice were strongly impaired in the hanging test, the hindlimb placing test and the inverted screen and had small gait abnormalities.

Hypersensitivity or hyposensitivity to sensory stimuli is frequently observed in PMS and ASD patients (Klintwall et al., 2011; Phelan and Betancur, 2011). However, little was known regarding the sensory abilities of *Shank3*-deficient mice. No deficits were reported in Δ4-9 or Δ4-22 animals for either olfaction, audition, vision, neuromuscular reflexes or pain sensitivity (Bozdagi et al., 2010; Wang et al., 2011, 2016b; Yang et al., 2012). Normal pre-pulse inhibition was observed in many models including Δ4-9, Δ13, Δ21, and Δ4-22 *Shank3*-deficient mice (Yang et al., 2012; Kouser et al., 2013; Wang et al., 2016b; Jaramillo et al., 2017) even if decreased pre-pulse inhibition was reported in in lines with point mutations in exon 21 (Zhou et al., 2016). Here, we observed that *Shank3^Δ4–22^* homozygous mice have no strong visual deficits, and normal neuromuscular reflexes, but are hyper-reactive in response to handling and tactile stimuli. In addition, we observed a delay in the acquisition of the startle response in newborns and a decrease of the startle response in both heterozygous and homozygous adults. Since social behavior strongly relies on olfaction in rodents, we used different behavioral paradigms to evaluate our model. Interestingly, *Shank3^Δ4–22^*homozygous mice had a low interest for nonsocial olfactory stimuli as shown by deficits in the buried food test and by low amount of sniffing during the olfactory habituation/dishabituation paradigm. However, *Shank3^Δ4-22^-*deficient mice were able to discriminate odors in the test for social transmission of food preference or to show interest for social stimuli during olfactory habituation/dishabituation, suggesting that they do not have anosmia but rather show reduced interest in nonsocial scents, which can be overcome when adding a social component.

One of the defining features of autism is the impairment of social interactions that can manifest by deficits in social approach, reciprocal social interactions and/or verbal and nonverbal communication. Mild social deficits have been reported, however with variability, in some of the previous studies of PMS mouse models (Fig. 11). In one of the most commonly used test, the three-chambered social approach test, no differences between the genotypes were reported in Δ4-9, Δ4-7, and Δ9 models (Peça et al., 2011; Yang et al., 2012; Drapeau et al., 2014; Lee et al., 2015), while social deficits characterized by a lack of preference for a social stimulus were reported the models targeting Δ11, Δ13, or Δ13-16 deletions (Peça et al., 2011; Duffney et al., 2015; Mei et al., 2016; Jaramillo et al., 2017; Luo et al., 2017; Vicidomini et al., 2017). Conflicting results were reported for Δ21 models (Kouser et al., 2013; Duffney et al., 2015; Speed et al., 2015; Bidinosti et al., 2016; Zhou et al., 2016). Interestingly, consistent with Wang et al., 2016b and colleagues’ study, we observed only minimal social deficit in our Δ4-22 model. All genotypes had a similar preference for social stimulus in the three-chambered social approach test or the social transmission of food preference and only trends toward a decrease of interaction time and vocalization were found during male-female social interactions. Rodent social behavior is highly influenced by experimental conditions such as the animals’ age, housing conditions, or animals handling and that can explain differences observed between cohorts of animals with identical or similar alterations of the Shank3 gene. While not representative of typical autism, this subtle behavior can reflect the phenotype of many patients with PMS. Indeed, unlike patients with idiopathic ASD, individuals with PMS show preserved responses to social communication cues (Soorya et al., 2013; Wang et al., 2016a) and roughly equal orienting to social versus nonsocial stimuli, despite meeting criteria for ASD. Moreover, the fact that not all individuals with PMS are diagnosed with ASD indicates that animal models for PMS should not necessarily present with strong social behavioral deficits. As the expression and alternative splicing of *Shank3* isoforms and even their subcellular distribution has been shown to be cell-type specific, activity dependent, and regionally and developmentally regulated (Wang et al., 2014), these differences also raise the possibility that different *Shank3* isoforms could make distinct contributions to the phenotype of PMS and suggests that *Shank3c* and *Shank3d* (affected by deletions containing exons 11-16) could be particularly involved in the regulation of social behavior compared to isoforms *Shank3a*, *Shank3b*, or *Shank3a/b* that are disrupted by deletions of exons 4-9. The apparent absence of social deficit in the models with a complete deletion of *Shank3* could be explained by the fact that those animals have a strong aversion for objects and be interpreted as an avoidance of the chamber containing the object rather than a real social preference.

One of the strongest phenotype observed in the current study was indeed an active avoidance of inanimate objects. In the novel object recognition test, lack of preference for a novel object had previously been observed in two lines of Δ4-9 mice (Wang et al., 2011; Yang et al., 2012) but not in a third line (Jaramillo et al., 2016) nor in Δ9 *Shank3*-deficient mice (Lee et al., 2015). However, in the present study, homozygous animals had very little interactions with both familiar and novel object making it impossible to properly compare novelty preference. Instead, they mostly spent their time in the corners of the open field away from the objects. Surprisingly, similar avoidance behavior was observed in the marble burying test and in the repetitive novel object contact task. We also observed a strong decrease of direct interactions with the applicator in the olfactory habituation/dishabituation test and a reduction of the quality of the nests build by *Shank3^Δ4–22^*-deficient animals with some mice even leaving the building material fully untouched. Some studies have reported that children with autism respond to novelty with avoidance behaviors and patients with PMS have enhanced reactivity to novel environments and reduced interest for objects. Decrease of marble burying has been consistently been described in other models of *Shank3* deficiency as were nest building impairments (Δ11, Δ13, Δ21, and exon 21 point mutations; Kouser et al., 2013; Speed et al., 2015; Bidinosti et al., 2016; Jaramillo et al., 2017; Vicidomini et al., 2017). While we have shown that those animals are hypoactive and have significant motor deficits that could impact behavioral assays relying on exploratory locomotion, it is unlikely that this avoidance behavior is attributable to impaired motor activity or poor motivation as homozygous mice have normal pattern of investigation in an empty open field and actively avoid objects or even escape from the cages by jumping out while they will not escape from an empty cage or a cage containing an unfamiliar mouse. Furthermore, the number of escape attempts increased in relation with the number of objects present in the cage. In addition to this escape behavior, a high level of impulsivity was observed for adult homozygous mice in the beam walking test and for both newborn and adult homozygous mice in the negative geotaxis test.

Stereotypies, repetitive behaviors with restricted interests and resistance to change form the second set of core symptoms of ASD. Excessive grooming with or without development of skin lesions is the most commonly observed repetitive behavior in rodents. Repetitive/compulsive grooming has been reported in most of the previously published *Shank3* mouse models (Fig. 11) while skin lesions where noticed only in some of them (Δ4-9, Δ11, Δ13-16, Δ21, and point mutations in exon 21; Peça et al., 2011; Schmeisser et al., 2012; Drapeau et al., 2014; Mei et al., 2016; Zhou et al., 2016) suggesting different levels of severity. The homozygous mice from Wang et al. (2016b) displayed both increased grooming and development of skin lesions. However, in the present study, even if we did occasionally observe some bald patches with or without skin lesions in our oldest animals all genotypes were impacted and group differences where only found for the grooming behavior. Our *Shank3^Δ4–22^*-deficient mice also engaged more frequently in other stereotyped and repetitive behaviors. By contrast, we did not observe any perseveration in the Y-maze nor object or pattern preference in the repetitive novel object contact task. To investigate both cognitive flexibility and insistence on sameness our animals were tested in the Barnes maze. The initial training showed a delay in the acquisition of the task in homozygous mice but after 4 d of training all genotypes had comparable performances and spent similar amount of time in the target quadrant during a probe test. Mice were then retrained after moving the escape box. Our homozygous mice exhibited impaired cognitive flexibility characterized by a delay in the time needed to learn the new rule and by a similar preference for either the reversal target quadrant or the initial target quadrant during the probe test; heterozygous mice had an intermediate phenotype. This suggests that *Shank3* deficiency increases susceptibility to proactive interference, a deficit associated with prefrontal cortex dysfunction. Similar reversal impairments have been published in either the Morris water maze or T-maze in Δ4-9, Δ11, Δ21, point mutations, or Δ4-22 mice (Wang et al., 2011; Kouser et al., 2013; Speed et al., 2015; Wang et al., 2016b; Vicidomini et al., 2017) while other models had comparable results for all genotypes (Δ4-9, Δ9; Yang et al., 2012; Lee et al., 2015; Jaramillo et al., 2016).

Because a majority of patients with *SHANK3* mutation/deletion exhibit some degree of ID, our animals were also tested for short-term memory by examining spontaneous alternation behavior in the Y-maze and for hippocampal or amygdala-dependent memories using contextual and cued fear conditioning. As in other models investigated (Δ4-9 and point insertions; Drapeau et al., 2014; Zhou et al., 2016), we found no differences in performance in the Y-maze spontaneous alternation test suggesting normal basic working memory. Neither contextual nor cued memories had been found to be affected by genotype in any of the previously published exon specific models (Δ4-9; Yang et al., 2012; Drapeau et al., 2014; Jaramillo et al., 2016) while a small increase of freezing was noticed in Δ4-22 homozygous mice during contextual recall (Wang et al., 2016b). Interestingly, in our new mouse model, we observed distinct responses to each phase of the testing. While not different at first during the pre-training habituation phase, the level of freezing quickly decreased in wild-type and heterozygous mice but not in the homozygous animals, likely reflecting a higher anxiety level. On presentation of the sound/shock associations, the increase of freezing was significantly more noted in homozygous mice. Remarkably, the opposite was observed during the contextual recall thus demonstrating an impairment of hippocampo dependent memory in homozygous animals, while the same mice displayed increased freezing on the presentation of sounds during the amygdala-dependent cued recall.

These region-specific alterations of behavior suggest that different *Shank3* deletions could alter different neuronal circuits through the modulation of the expression of different *Shank3* isoforms. The *Shank3b* isoform (present in the Δ21 mouse models) is expressed at low level throughout the brain, while a regional specificity was observed for the other *Shank3* isoforms. *Shank3a* (absent in all the mouse models) and *Shank3e* (absent only in Δ21 and complete gene models) are highly expressed in the striatum but are low in the olfactory bulb and the cerebellum. In contrast, *Shank3c* (absent in Δ9, Δ4-7, Δ4-9, and complete gene models) and *Shank3d* (absent in Δ13-16, Δ21, and complete gene models) are predominantly enriched in the cerebellum (Wang et al., 2014). Specific subcategories of learning and memory behaviors have only been studied in limited number of previous models. Heterozygous Δ21 mice lacking the cerebellum-specific *Shank3c* and *Shank3d* isoforms as well as *Shank3e* and *Shank3f* isoforms exhibit impaired eye-blink conditioning, a cerebellar-dependent learning task (Kloth et al., 2015). Δ13-16 *Shank3*-deficient mice are impaired in pairwise visual discrimination learning in the automated touchscreen task depending on normal functions of interconnected cortical and subcortical regions (Copping et al., 2017). Finally, Δ4-22 homozygous mice have deficits in a striatal-dependent instrumental learning task (Wang et al., 2016b). Further studies examining the extend of impairment of region-specific behaviors will be required to fully understand the relationships between brain circuitry, *Shank3* isoforms expression, and behavior.

Altogether, the hyper-reactivity to handling and tactile stimuli, the impulsivity, the object neophobia, the escape behavior, the increased freezing response in the pre-training phase of the fear conditioning and in cued retrieval suggest high levels of anxiety in our mouse model. Hyperactivity and anxiety symptoms are other common features of PMS (Dhar et al., 2010; Soorya et al., 2013; Sarasua et al., 2014a). In previously published models, analysis of anxiety-like behavior measured either elevated mazes, in the open fields or in dark/light emergence boxes have demonstrated a relationship between the targeted isoforms and the manifestations of anxiety like-behavior. While little differences were observed in mouse models with Δ4-9, Δ4-7, Δ9, and Δ11 deletions (Peça et al., 2011; Wang et al., 2011; Schmeisser et al., 2012; Yang et al., 2012; Drapeau et al., 2014; Lee et al., 2015; Jaramillo et al., 2016; Reim et al., 2017; Vicidomini et al., 2017) increased levels of anxiety were reported in mice with Δ13, Δ13-16, and Δ21 deletions or point mutations (Peça et al., 2011; Kouser et al., 2013; Speed et al., 2015; Mei et al., 2016; Zhou et al., 2016; Copping et al., 2017; Jaramillo et al., 2017). Increased levels of anxiety were confirmed in the light-dark emergence test and in the open field in the Δ4-22 mouse model from Wang et al., 2016b and colleagues and in the elevated maze and in the open field in our model.

In conclusion, our complete *Shank3^Δ4–22^*mouse line provides a new and improved genetic model for studying mechanisms underlying ASD and PMS and is characterized both by better construct and face validities than previously reported lines of *Shank3* mutants. Our in-depth behavioral characterization revealed behavioral features that reflect those observed in PMS and therefore suggest a greater potential as a translational model. Mice with a complete deletion of *Shank3* are more severely affected than previously published mouse models with a partial deletion. Both sensory and motor disabilities were detected in neonate and adult mice. *Shank3^Δ4–22^*-deficient mice showed modest deficits in social behavior, reflected in reduced male to female anogenital sniffing and ultrasonic vocalization, but no major deficits in social preference in the three-chambered social interaction task. These findings are consistent with an independently generated mouse model (Wang et al., 2016b). Also in agreement with Wang’s study, our *Shank3* ^Δ4–22^ mice showed increased anxiety and hyper-reactivity to novel stimuli, increased escape behaviors, and increased repetitive behaviors. Together with the increased freezing behavior in the cued fear conditioning, this suggest a dysregulation of amygdala circuitry that will require further investigation. In addition, our mice displayed impairments in several hippocampal-dependent learning and memory tests as well as cognitive inflexibility, thus recapitulating ID and insistence on sameness observed in autism and in the majority of patients with PMS. Although PMS patients are heterozygous for *Shank3* mutations/deletions, most of the previous models have failed to demonstrate any relevant phenotype in heterozygous animals. Here, we were able to observe an intermediate phenotype for heterozygous mice in several of the parameters tested, notably in the open field, rotarod, startle response, escape behavior, reversal probe test, and elevated zero-maze. Heterozygous animals being less affected than their homozygous, we hypothesize that more challenging paradigms, for example by introducing a variable reward probability in tests such as the Barnes maze, would allow us to further highlight differences in heterozygous animals. Past studies have often failed to replicate behavioral phenotype even in models with very similar *Shank3* disruption or in different cohorts from the same model. The concordant findings from two independently derived and analyzed *Shank3* mouse models, including the comparison of two independent cohorts in our laboratory, demonstrate, for the first time, strong reproducibility and validity for a genetically modified mouse model of PMS, providing a valuable model for further investigations of the neurobiological basis of PMS and ASD.

## Synthesis

Reviewing Editor: Lorna Role, Stony Brook Decisions are customarily a result of the Reviewing Editor and the peer reviewers coming together and discussing their recommendations until a consensus is reached. When revisions are invited, a fact-based synthesis statement explaining their decision and outlining what is needed to prepare a revision will be listed below. The following reviewer(s) agreed to reveal their identity: Lei Zhu.

This manuscript is a report of the generation and behavioral characterization of a novel Shank3 knockout mice. Mutations in the Shank3 gene -which encodes an important cytoskeletal protein with multiple protein-protein interaction domains -- have been linked to diseases including ASD and Phelan-McDermid Syndrome.

There have been many different lines of knockout/transgenic mice generated to target SHANK3. Because of the size and complexity of the SHANK3 gene, in most of these lines certain isoforms of Shank3 were deleted but the persistence of other isoforms has complicated the interpretation of the results. The first “complete” Shank3 deletion mice was reported by Wang et al in 2016. This paper represents the second report of a complete deletion in which all Shank3 isoforms are eliminated.

In general, it was felt that the manuscript is very strong. The writing is compelling and the data are convincing. The consensus was that the readers would benefit even more from the publication if some more of the methodological details were added and if the authors would consider adding to the text and discussion some highlights of the differences obtained by comparing their model with the other complete Shank 3 Kos ( and with the partial Kos)

Specifically,

1. It would be helpful if the authors could mention as many of the tests performed as possible in the abstract to the paper, as this could be used for indexing and keyword searches.

2. Please include the JAX mouse numbers or other public databases where appropriate for SHANK models highlighted in table 14.

3. Instead of referring to “an ubiquitous Cre” please specify that the promoter driving Cre was CMV in both the abstract and in the methods. Also please give the relevant JAX numbers for the Cre-mice used for mating with the loxP SHANK mouse.

4. Although the focus of this study is on behavior, it may be helpful to the reader to provide some discussion on the potential involvement of particular regions/circuits that are important for the expression of the behavior defects. The authors hinted that they thought that circuit defects within the amygdala, were relevant. Previous studies have addressed changes in striatum, hippocampus, etc. Is there reason to consider that some of the differences between the complete KOs, partial Shank 3 deletions and WT are due to the involvement of different circuits? (e.g. differential expression of various isoforms?)

5. The behavior tests focus on four cohorts of two age groups, new born and 3-10 months old. It might be helpful to the reader to include a discussion of how the age differences in this study and in comparison with others could impact the results. For example, at 3 weeks, the body weight of the KO mice is significantly less that of the WT mice, whereas by 3 months, the WT and KO mice are of comparable weight. Do the authors think the full KO may result in differences in in utero development (or some other developmental delay), that are subsequently corrected? ( eg placental insufficiency corrected post birth by feeding?)

6. Table 14 provides a comprehensive summary and comparison of the results from many different lines of Shank3 mice. Variations clearly exist among these results. Surprisingly, studies of the more recent complete Shank3 KO mice fail to reveal any differences from the WT mice in the 3-chamber social interaction test. This is in contrast with the findings in which exons 13-16 were deleted - where significant defects were reported compared with WT.

It would be helpful if the authors provided further clarification of their findings in comparison with others' results - especially where more complete deletions have lesser effects than the more limited ones. Where reproducibility is so important (and cost so great) the reader will benefit from discussion of each of the findings - whether positive or negative - in the current study.

## Author Response

Dear reviewers, We would like to thank you for your time, the thorough review of our work and your useful comments and suggestions. Please find enclosed the revised version of the manuscript entitled “Behavioral phenotyping of an improved mouse model of Phelan-McDermid Syndrome with a complete deletion of the Shank3 gene” that we would like to resubmit to eNeuro (reference number eN-CFN-004618). We have taken your comments and suggestions into account and we hope that the manuscript has improved in clarity, technical precision and general quality. Please find below a detailed reply to the comments of the referee and a description of the changes made.

1. It would be helpful if the authors could mention as many of the tests performed as possible in the abstract to the paper, as this could be used for indexing and keyword searches.

Answer: The abstract has been modified to mention the names of the most relevant tests used in the study.

2. Please include the JAX mouse numbers or other public databases where appropriate for SHANK models highlighted in table 14. Answer: We thank the reviewers for this suggestion. JAX mouse numbers were added into table 1 (contains more models than table 14 that is limited to behavioral studies and shows the relationships between the different models) whenever they were available. The name of the principal investigator responsible for providing the model was also included.

3. Instead of referring to “an ubiquitous Cre” please specify that the promoter driving Cre was CMV in both the abstract and in the methods. Also please give the relevant JAX numbers for the Cre-mice used for mating with the loxP SHANK mouse. Answer: Ubiquitous Cre was replaced by CMV-Cre throughout the manuscript and the reference of the mouse line used (Tg(CMV-cre)1Cgn, The Jackson Laboratory, #006054) was added in the Material and Methods section.

4. Although the focus of this study is on behavior, it may be helpful to the reader to provide some discussion on the potential involvement of particular regions/circuits that are important for the expression of the behavior defects. The authors hinted that they thought that circuit defects within the amygdala, were relevant. Previous studies have addressed changes in striatum, hippocampus, etc. Is there reason to consider that some of the differences between the complete KOs, partial Shank 3 deletions and WT are due to the involvement of different circuits? (e.g. differential expression of various isoforms?) Answer: As suggested by the reviewers, the differences observed between WT, partial KO and complete KO could be explained by the differential expression of various isoforms if different brains areas. While

Shank3b isoform (present only in the 21 mouse models) is expressed at low level throughout the brain a regional specificity was observed for the other Shank3 isoforms. Shank3a (absent in all the mouse models) and Shank3e (absent only in 21 and complete models) are highly expressed in the striatum but low in the olfactory bulb and the cerebellum. In contrast, Shank3c (absent in 9, 4-7, 4-9 and complete models) and Shank3d (absent in 13-16, 21 and complete models) are predominantly enriched in the cerebellum. Unfortunately, region specific behaviors have not been studied throughout all the models (for example, impaired eye-blink conditioning, a cerebellar-dependent learning task was studied only in 21 mice) making it difficult to fully correlate the behavior deficits to the region-specific isoform expression. However, discussion of relationships between brain regions, isoforms and deletions was added whenever possible.

5. The behavior tests focus on four cohorts of two age groups, new born and 3-10 months old. It might be helpful to the reader to include a discussion of how the age differences in this study and in comparison with others could impact the results. For example, at 3 weeks, the body weight of the KO mice is significantly less that of the WT mice, whereas by 3 months, the WT and KO mice are of comparable weight. Do the authors think the full KO may result in differences in in utero development (or some other developmental delay), that are subsequently corrected? ( eg placental insufficiency corrected post birth by feeding?) Answer: To facilitate the comparison with previous studies, the testing age range has been added in the behavioral summary table and the influence of age is discussed when appropriate.

In agreement with the reviewers' suggestion we have observed both a significant deficit in the number of homozygous mice compared to the expected Mendelian distribution and an increased postnatal mortality of the same homozygous mice (now added in the manuscript) that can partially explain this deficit. Altogether, this indeed suggest in utero developmental delays or deficits leading to an increased pre- and postnatal mortality of the homozygous animals. Animals that survive this period initially have a slower growth curve than their littermates but

6. Table 14 provides a comprehensive summary and comparison of the results from many different lines of Shank3 mice. Variations clearly exist among these results. Surprisingly, studies of the more recent complete Shank3 KO mice fail to reveal any differences from the WT mice in the 3-chamber social interaction test. This is in contrast with the findings in which exons 13-16 were deleted - where significant defects were reported compared with WT. It would be helpful if the authors provided further clarification of their findings in comparison with others' results - especially where more complete deletions have lesser effects than the more limited ones. Where reproducibility is so important (and cost so great) the reader will benefit from discussion of each of the findings - whether positive or negative - in the current study. Answer: The paragraph discussing social behavior has now been modified to contrast findings from models deleting exons 4 to 9 (social behavior weekly affected), models deleting exons 13 to 16 (social behavior strongly affected) and models with complete deletions (social behavior similar to 4-9) in order

to discuss the impact of differential isoform expression and provide hypothesis regarding the absence of deficits in mice with complete deletions.
