# Supplementary figures and images for: Behavioral Phenotyping of an Improved Mouse Model of Phelan–McDermid Syndrome with a Complete Deletion of the Shank3 Gene
Source: eNeuro. 2018 Oct 5;5(3):ENEURO.0046-18.2018. doi: 10.1523/ENEURO.0046-18.2018 (PMC6175061; doi:10.1523/ENEURO.0046-18.2018)

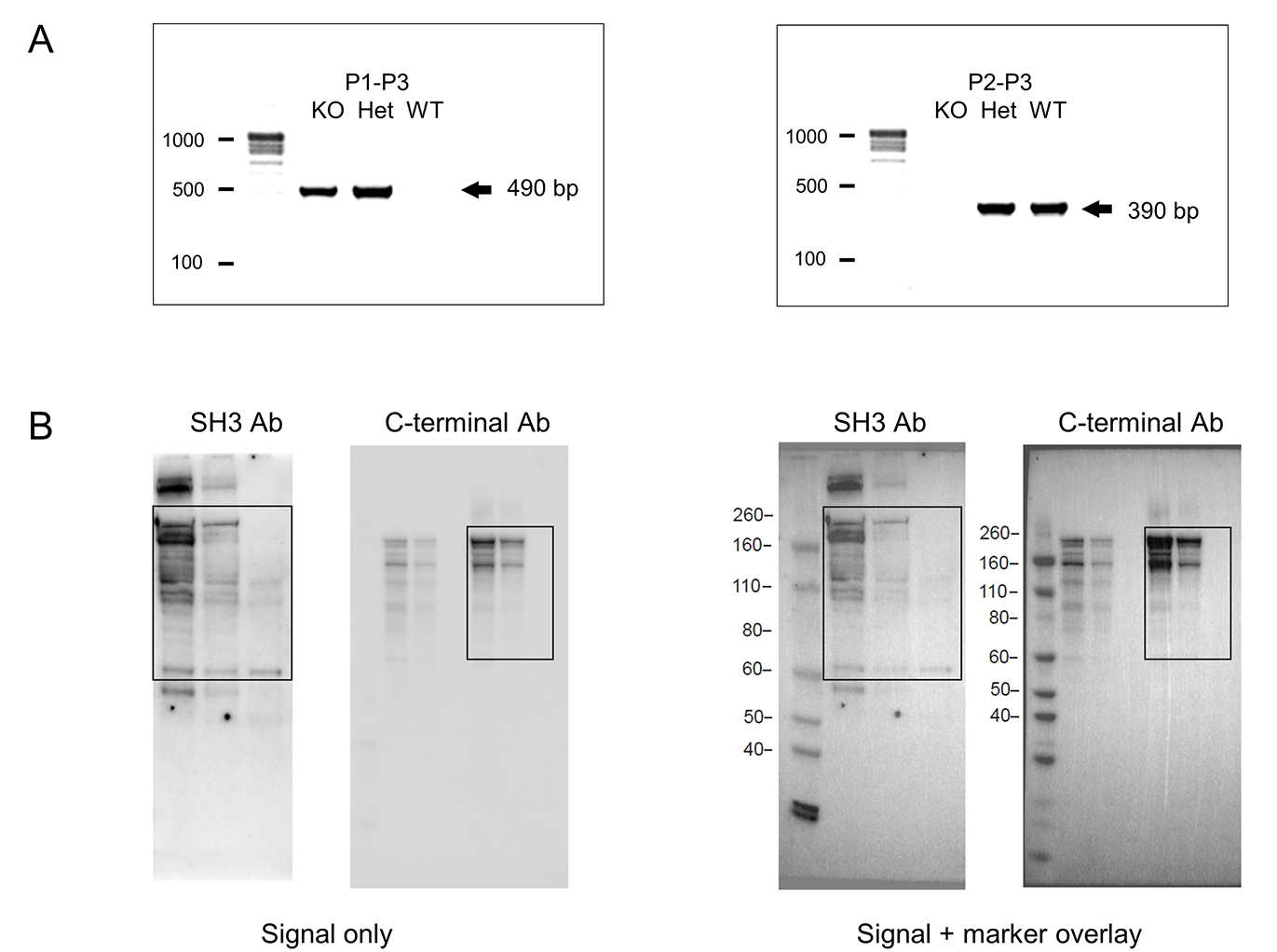

Supplement: Extended Data Figure 1-1 — Validation of a knockout mice with a complete deletion of Shank3 (A) Genotyping of Shank3∆4-22 mice by PCR. The P1-P3 primer pair produced a 490 bp band identifying the ∆4-22 allele, while the P2-P3 primer pair amplified the 390 bp product from the wild-type allele. (B) Original full scans of immunoblots related to Figure 1. WT, wild-type mice; Het, heterozygous mice; KO, homozygous knockout mice. Download Figure 1-1, TIF file. [file sup_enu-eN-CFN-0046-18-s03.tif]

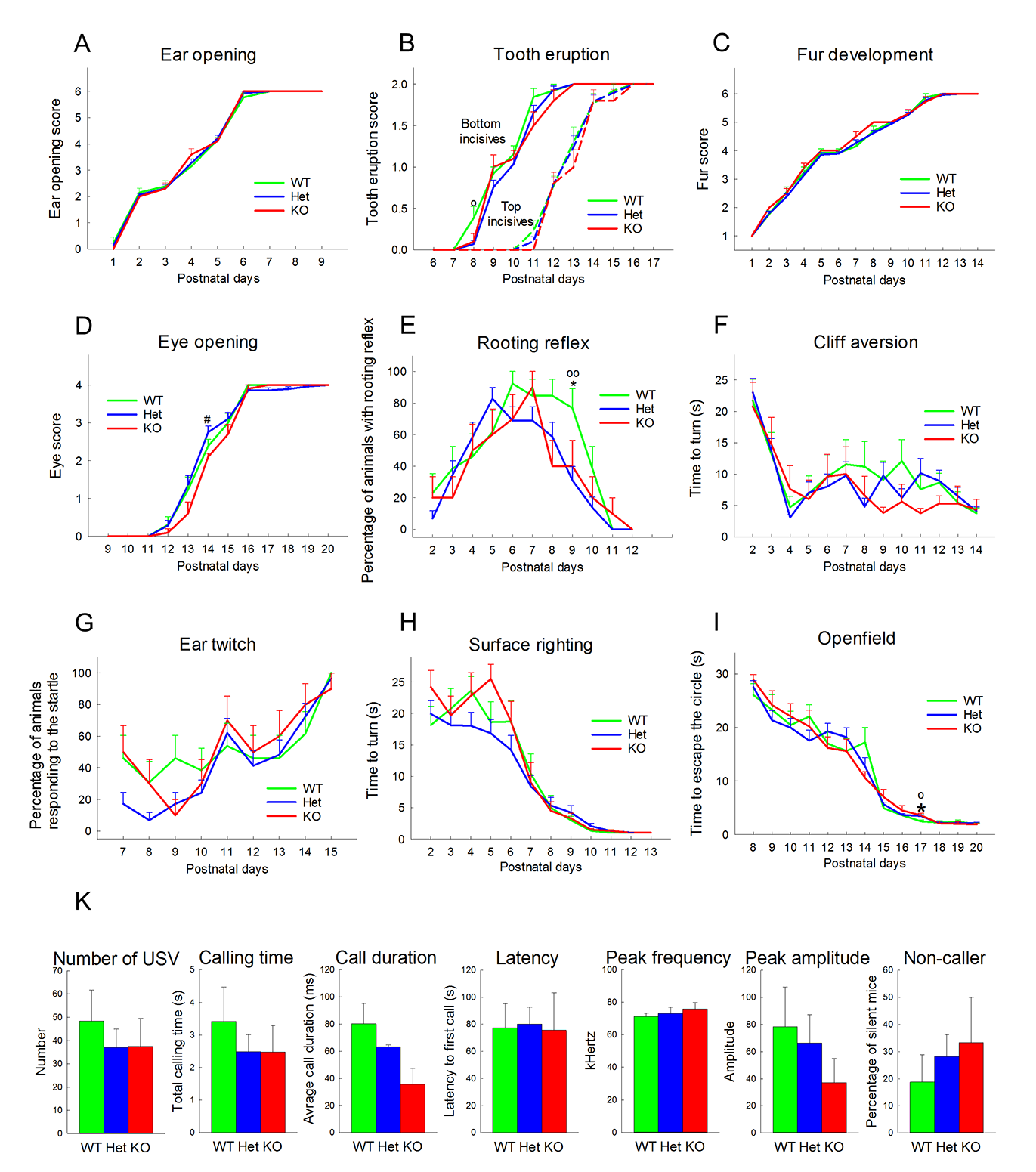

Supplement: Extended Data Figure 2-1 — Normal developmental milestones of Shank3Δ4-22 deficient mice. Analysis of the markers of developmental milestones that revealed no major genotype differences in Shank3 wild-type, heterozygous and homozygous pups between postnatal days 1 and 21 on measures of (A) ear opening, (B) tooth eruption, (C) fur development, (D) eye opening, (E) rooting reflex, (F) cliff aversion, (G) ear twitch, (H) surface righting and (I) open field crossing. (K) Only non-significant differences were detected in the number and quality of ultrasonic vocalizations emitted by 6-day old pups. WT, wild-type mice; Het, heterozygous mice; KO, homozygous knockout mice. *: WT vs KO; o: WT vs Het, #: Het vs KO. *: p < 0.05, **: p < 0.1, ***: p < 0.001. Download Figure 2-1, TIF file. [file sup_enu-eN-CFN-0046-18-s04.tif]

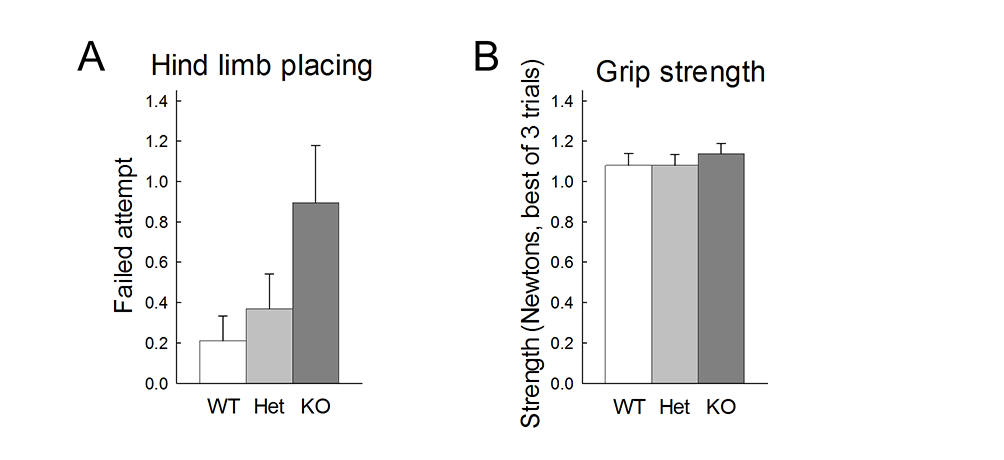

Supplement: Extended Data Figure 3-1 — Motor functions in in Shank3Δ4-22 -deficient mice. (A) Number of failed attempts (falls or hanging without being able to pull itself up in less than 60 seconds) in the hind placing test. The number of failed attempts was more important in Shank3Δ4-22 homozygous mice. (B) Strength measured in the grip strength. No genotype difference was found for acute grip strength. WT, wild-type mice; Het, heterozygous mice; KO, homozygous knockout mice. *: WT vs KO; o: WT vs Het, #: Het vs KO. *: p < 0.05, **: p < 0.1, ***: p < 0.001. Download Figure 3-1, TIF file. [file sup_enu-eN-CFN-0046-18-s05.tif]

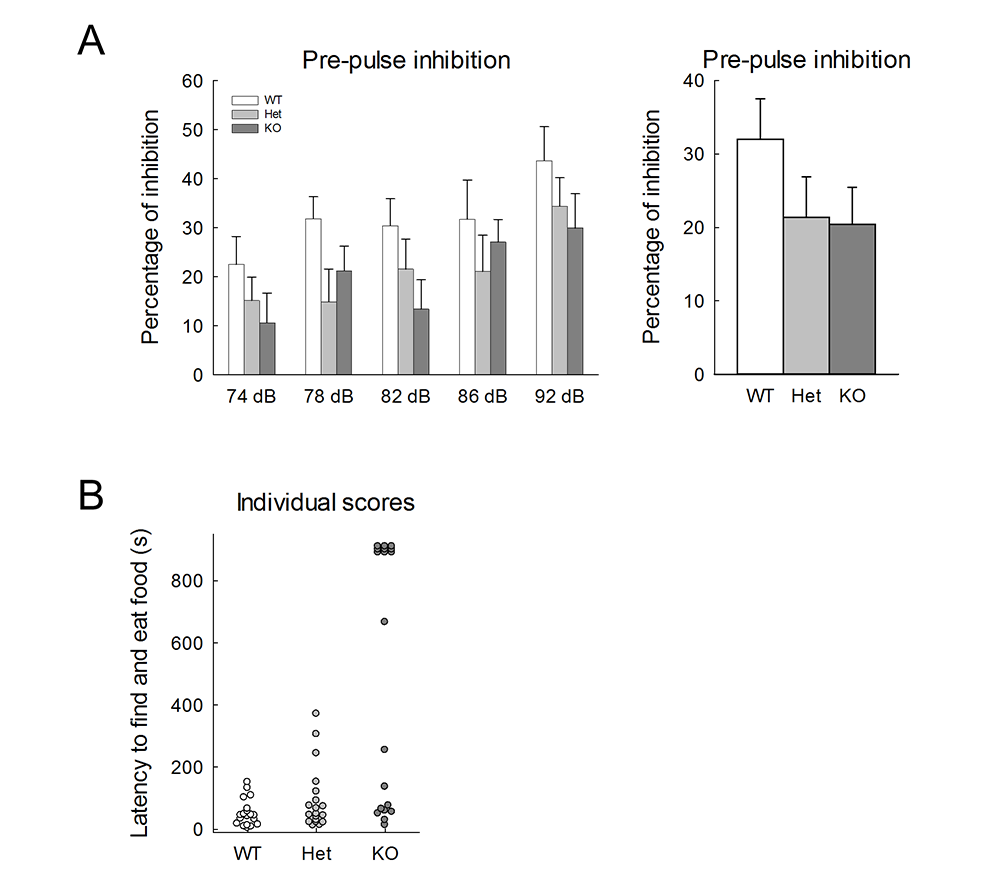

Supplement: Extended Data Figure 4-1 — Altered sensory profile in Shank3 complete knockout mice. (A) Pre-pulse inhibition. A non-significant decrease of pre-pulse inhibition was observed in both heterozygous and homozygous Shank3Δ4-22 mice compared to wild-type animals. (B)Individual scores in buried food test showing that 50% of Shank3Δ4-22 homozygous mice fail to retrieve buried food (cut-off of 900 seconds). WT, wild-type mice; Het, heterozygous mice; KO, homozygous knockout mice. Download Figure 4-1, TIF file. [file sup_enu-eN-CFN-0046-18-s06.tif]

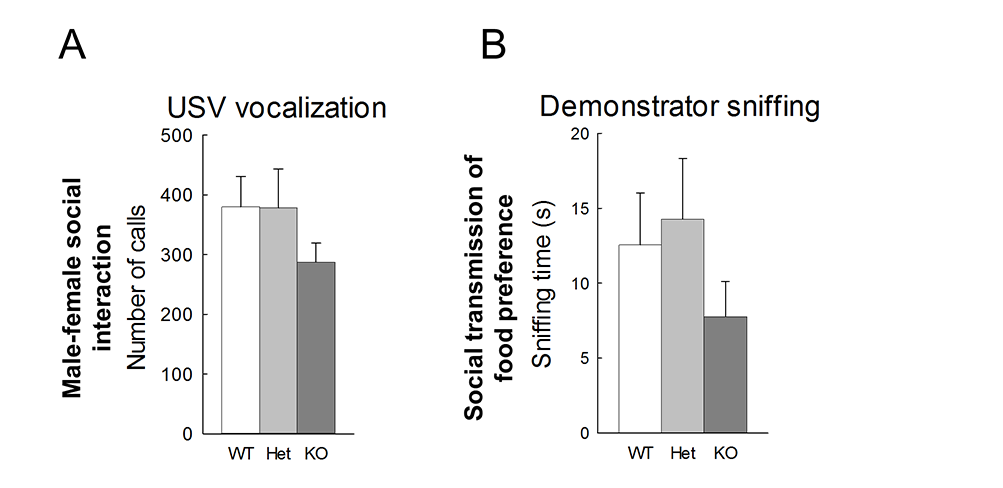

Supplement: Extended Data Figure 5-1 — Social interactions in ShankΔ4-22-deficient mice. (A) Male female social interaction. A non-significant decrease of the number of ultrasonic vocalization was observed in males Shank3Δ4-22 homozygous mice upon exposure to an estrus female. (B)Social transmission of food preference. A trend toward a reduction of sniffing during the demonstrator interaction phase was observed in in Shank3Δ4-22 homozygous mice. Download Figure 5-1, TIF file. [file sup_enu-eN-CFN-0046-18-s07.tif]

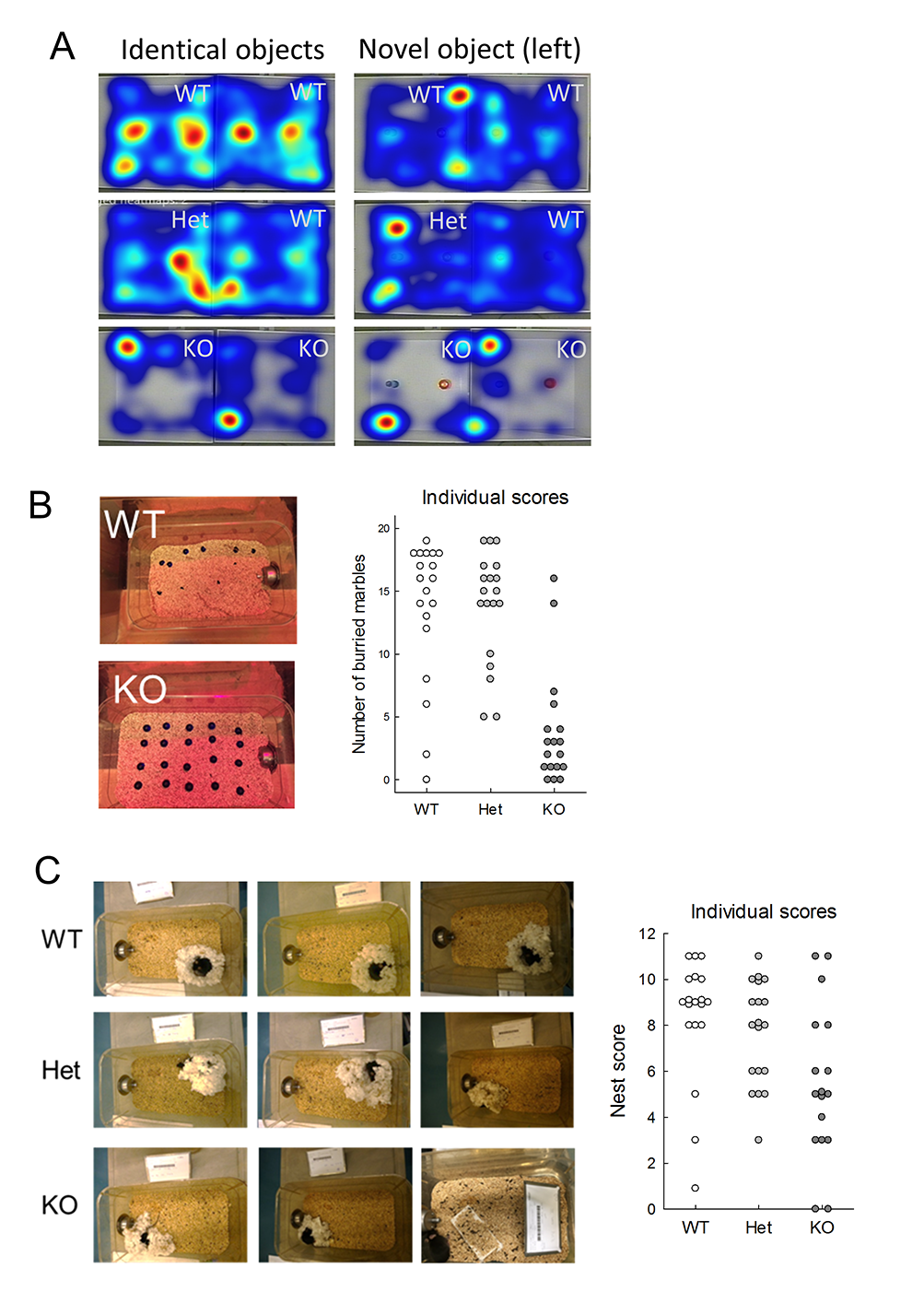

Supplement: Extended Data Figure 6-1 — Pictures and individual scores representative of object avoidance behavior. (A) Heatmaps of the novel object recognition task showing object interactions in wild-type and heterozygous Shank3Δ4-22 mice and object avoidance in homozygous Shank3Δ4-22 mice. (B) Representative picture of marble positions and individual scores in the marble burying test showing that in many instance Shank3Δ4-22 homozygous mice left the marbles completely undisturbed.(C) Representative picture of marble positions and individual scores in the nest building test showing a lower quality of nest building in Shank3Δ4-22 homozygous mice with some animals only coarsely shredding the nestlets without building a real nest and others even leaving the nestlets completely untouched. WT, wild-type mice; Het, heterozygous mice; KO, homozygous knockout mice. Download Figure 6-1, TIF file. [file sup_enu-eN-CFN-0046-18-s08.tif]
