# Supplementary material for: Behavioral Phenotyping of an Improved Mouse Model of Phelan–McDermid Syndrome with a Complete Deletion of the Shank3 Gene
Source: eNeuro. 2018 Oct 5;5(3):ENEURO.0046-18.2018. doi: 10.1523/ENEURO.0046-18.2018 (PMC6175061; doi:10.1523/ENEURO.0046-18.2018)
Supplement: Extended Data Table 5-1 — Individual results and statistical analyses for cohorts 1 and 2 related to general health, physical factors, gross appearance and spontaneous activity. WT, wild-type mice; Het, heterozygous mice; KO, homozygous knockout mice. Group values are reported as means ± s.e.m. Red font indicates significant results (p < 0.05), orange font indicates trends (0.1 < p < 0.05). Download Table 5-1, DOCX file. [file sup_enu-eN-CFN-0046-18-s02.docx]

# Extended Tables

Extended Table 5-1

| **Physical factors and gross appearance** | |  |  |  |  |  |  |  |  |  |  |  |  |  |  |  |  |  |  |  |  |  |  |
| --- | --- | --- | --- | --- | --- | --- | --- | --- | --- | --- | --- | --- | --- | --- | --- | --- | --- | --- | --- | --- | --- | --- | --- |
|  | Cohort 1 | | | | | | | | | | |  | Cohort 2 | | | | | | | | | | |
|  | test | data structure | WT | Het | KO | genotype | | | pairwise comparisons | | |  | test | data structure | WT | Het | KO | genotype | | | pairwise comparisons | | |
|  |  |  |  |  |  | F | p-value | power | WT vs Het | WT vs KO | Het vs KO |  |  |  |  |  |  | F | p-value | power | WT vs Het | WT vs KO | Het vs KO |
| Weight at 3 months (grams) | ANOVA | normal | 26.16 ± 1.36 | 26.73 ± 0.81 | 24.53 ± 0.66 | 2.515 | 0.100 | 0.460 | - | - | - |  | ANOVA | normal | 26.55 ± 1 | 27.7 ± 0.81 | 27.34 ± 0.6 | 0.505 | 0.610 | 0.123 | - | - | - |
| Weight at 15 months (grams) | ANOVA | normal | 31.02 ± 1.66 | 31.56 ± 1.99 | 29.06 ± 2.79 | 0.514 | 0.606 | 0.122 | - | - | - |  | ANOVA | normal | 35.52 ± 4.22 | 30.48 ± 1.42 | 29.8 ± 1.73 | 1.321 | 0.300 | 0.235 | - | - | - |
| Weight at 20 months (grams) | ANOVA | normal | 32.95 ± 2.11 | 31.2 ± 1.82 | 28.35 ± 2.84 | 0.663 | 0.532 | 0.138 |  |  |  |  | ANOVA | normal | 32.8 ± 4.04 | 31.76 ± 1.61 | 29.16 ± 2.17 | 0.381 | 0.694 | 0.094 | - | - | - |
| Length | Kruskal-Wallis | non normal | 15.81 ± 0.16 | 16.05 ± 0.22 | 15.72 ± 0.18 | 1.247 | 0.536 | NA | - | - | - |  | Kruskal-Wallis | non normal | 17.32 ± 0.36 | 17.72 ± 0.17 | 17.65 ± 0.17 | 0.513 | 0.774 | NA | - | - | - |
| Coat appearance | Kruskal-Wallis | non normal | 2.45 ± 0.2 | 2.9 ± 0.1 | 2.88 ± 0.11 | 4.809 | *0.090* | NA | - | - | - |  | Kruskal-Wallis | non normal | 2.87 ± 0.12 | 2.88 ± 0.11 | 3 ± 0 | 1.235 | 0.530 | NA | - | - | - |
| Skin color | - | - | 0 ± 0 | 0 ± 0 | 0 ± 0 | - | - | - | - | - | - |  |  |  | 0 ± 0 | 0 ± 0 | 0 ± 0 | - | - | - | - | - | - |
| Whisker barbering | - | - | 0 ± 0 | 0 ± 0 | 0 ± 0 | - | - | - | - | - | - |  |  |  | 0 ± 0 | 0 ± 0 | 0 ± 0 | - | - | - | - | - | - |
| Patches of missing fur on face | - | - | 0 ± 0 | 0 ± 0 | 0 ± 0 | - | - | - | - | - | - |  |  |  | 0 ± 0 | 0 ± 0 | 0 ± 0 | - | - | - | - | - | - |
| Patches of missing fur on body | - | - | 0 ± 0 | 0 ± 0 | 0 ± 0 | - | - | - | - | - | - |  |  |  | 0 ± 0 | 0 ± 0 | 0 ± 0 | - | - | - | - | - | - |
| Wounding | Kruskal-Wallis | non normal | 2.45 ± 0.2 | 2.9 ± 0.1 | 2.88 ± 0.11 | 1.727 | 0.422 | NA | - | - | - |  | Kruskal-Wallis | non normal | 2.87 ± 0.12 | 2.88 ± 0.11 | 3 ± 0 | 1.079 | 0.583 | NA | - | - | - |
| Body tone | Kruskal-Wallis | non normal | 1.18 ± 0.12 | 1.2 ± 0.13 | 1 ± 0.16 | 1.093 | 0.579 | NA | - | - | - |  |  |  | 1 ± 0 | 1 ± 0 | 1 ± 0 | - | - | - | - | - | - |
| Palpebral closure | - | - | 0 ± 0 | 0 ± 0 | 0 ± 0 | - | - | - | - | - | - |  |  |  | 0 ± 0 | 0 ± 0 | 0 ± 0 | - | - | - | - | - | - |
| Spontaneous piloerection | - | - | 0 ± 0 | 0 ± 0 | 0 ± 0 | - | - | - | - | - | - |  |  |  | 0 ± 0 | 0 ± 0 | 0 ± 0 | - | - | - | - | - | - |
|  |  |  |  |  |  |  |  |  |  |  |  |  |  |  |  |  |  |  |  |  |  |  |  |
| **Jar observation** |  |  |  |  |  |  |  |  |  |  |  |  |  |  |  |  |  |  |  |  |  |  |  |
|  | Cohort 1 | | | | | | | | | | |  | Cohort 2 | | | | | | | | | | |
|  | test | data structure | WT | Het | KO | genotype | | | pairwise comparisons | | |  | test | data structure | WT | Het | KO | genotype | | | pairwise comparisons | | |
|  |  |  |  |  |  | F | p-value | power | WT vs Het | WT vs KO | Het vs KO |  |  |  |  |  |  | F | p-value | power | WT vs Het | WT vs KO | Het vs KO |
| Body position | Kruskal-Wallis | non normal | 4.09 ± 0.09 | 4 ± 0 | 4.11 ± 0.11 | 1.067 | 0.587 | NA | - | - | - |  | Kruskal-Wallis | non normal | 4.25 ± 0.16 | 4.22 ± 0.14 | 4.4 ± 0.16 | 0.804 | 0.669 | NA | - | - | - |
| Spontaneous activity | Kruskal-Wallis | non normal | 1.63 ± 0.15 | 1.5 ± 0.16 | 1.66 ± 0.16 | 0.622 | 0.733 | NA | - | - | - |  | Kruskal-Wallis | non normal | 1.75 ± 0.16 | 1.66 ± 0.16 | 1.6 ± 0.16 | 0.433 | 0.805 | NA | - | - | - |
| Latency to sit/stand (seconds) | - | - | 0 ± 0 | 0 ± 0 | 0 ± 0 | - | - | - | - | - | - |  | - | - | 0 ± 0 | 0 ± 0 | 0 ± 0 | - | - | - | - | - | - |
| Latency to rear (seconds) | Kruskal-Wallis | non normal | 8.63 ± 1.96 | 7.9 ± 1.4 | 5.77 ± 1.97 | 2.390 | 0.303 | NA | - | - | - |  | Kruskal-Wallis | non normal | 9.37 ± 1.92 | 8.22 ± 1.59 | 5.5 ± 0.93 | 3.934 | 0.140 | NA | - | - | - |
| Repeated jumps (percentage of mice) | Kruskal-Wallis | non normal | 9.09 ± 9.09 | 0 ± 0 | 11.11 ± 11.11 | 1.067 | 0.587 | NA | - | - | - |  | Kruskal-Wallis | non normal | 25 ± 16.36 | 22.22 ± 14.69 | 40 ± 16.32 | 0.804 | 0.669 | NA | - | - | - |
| Circling (percentage of mice) | Kruskal-Wallis | non normal | 9.09 ± 9.09 | 20 ± 13.33 | 22.22 ± 14.69 | 0.710 | 0.701 | NA | - | - | - |  | - | - | 0 ± 0 | 0 ± 0 | 0 ± 0 | - | - | - | - | - | - |
| Urination | Kruskal-Wallis | non normal | 0.63 ± 0.27 | 0 ± 0 | 0.11 ± 0.11 | 5.419 | *0.067* | NA | - | - | - |  | - | - | 0 ± 0 | 0 ± 0 | 0 ± 0 | - | - | - | - | - | - |
| Defecation (number) | Kruskal-Wallis | non normal | 2 ± 0.48 | 0.5 ± 0.3 | 0.88 ± 0.45 | 5.738 | *0.057* | - | - | - | - |  | Kruskal-Wallis | non normal | 0.25 ± 0.25 | 0.22 ± 0.22 | 0.2 ± 0.2 | 0.027 | 0.987 | NA | - | - | - |
| respiration | - | - | 2 ± 0 | 2 ± 0 | 2 ± 0 | - | - | - | - | - | - |  | - | - | 2 ± 0 | 2 ± 0 | 2 ± 0 | - | - | - | - | - | - |
| tremor | - | - | 0 ± 0 | 0 ± 0 | 0 ± 0 | - | - | - | - | - | - |  | - | - | 0 ± 0 | 0 ± 0 | 0 ± 0 | - | - | - | - | - | - |
|  |  |  |  |  |  |  |  |  |  |  |  |  |  |  |  |  |  |  |  |  |  |  |  |
| **Cage transfer** |  |  |  |  |  |  |  |  |  |  |  |  |  |  |  |  |  |  |  |  |  |  |  |
|  | Cohort 1 | | | | | | | | | | |  | Cohort 2 | | | | | | | | | | |
|  | test | data structure | WT | Het | KO | genotype | | | pairwise comparisons | | |  | test | data structure | WT | Het | KO | genotype | | | pairwise comparisons | | |
|  |  |  |  |  |  | F | p-value | power | WT vs Het | WT vs KO | Het vs KO |  |  |  |  |  |  | F | p-value | power | WT vs Het | WT vs KO | Het vs KO |
| Transfer arousal | Kruskal-Wallis | non normal | 3.63 ± 0.24 | 3.2 ± 0.29 | 3.11 ± 0.35 | 2.095 | 0.351 | NA | - | - | - |  | Kruskal-Wallis | non normal | 2.62 ± 0.32 | 3.22 ± 0.36 | 3.2 ± 0.24 | 2.529 | 0.282 | NA | - | - | - |
| Gait | Kruskal-Wallis | non normal | 0 ± 0 | 0.2 ± 0.13 | 0 ± 0 | 4.142 | 0.126 | NA | - | - | - |  | Kruskal-Wallis | non normal | 0 ± 0 | 0.11 ± 0.11 | 0.1 ± 0.1 | 0.884 | 0.643 | NA | - | - | - |
| Pelvic elevation | Kruskal-Wallis | non normal | 2 ± 0 | 2.2 ± 0.13 | 2 ± 0 | 4.143 | 0.126 | NA | - | - | - |  | Kruskal-Wallis | non normal | 2 ± 0 | 2.11 ± 0.11 | 2 ± 0.14 | 0.643 | 0.725 | NA | - | - | - |
| Tail elevation | Kruskal-Wallis | non normal | 2 ± 0.23 | 2 ± 0.29 | 1.44 ± 0.24 | 3.175 | 0.204 | NA | - | - | - |  | Kruskal-Wallis | non normal | 1.75 ± 0.16 | 1.44 ± 0.17 | 1 ± 0.25 | 7.001 | **0.030** | NA | 0.591 | *0.051* | 0.300 |
