# Supplementary material for: Behavioral Phenotyping of an Improved Mouse Model of Phelan–McDermid Syndrome with a Complete Deletion of the Shank3 Gene
Source: eNeuro. 2018 Oct 5;5(3):ENEURO.0046-18.2018. doi: 10.1523/ENEURO.0046-18.2018 (PMC6175061; doi:10.1523/ENEURO.0046-18.2018)
Supplement: Extended Data Table 6-1 — Individual results and statistical analyses for cohorts 1 and 2 related to motor functions. WT, wild-type mice; Het, heterozygous mice; KO, homozygous knockout mice. Group values are reported as means ± s.e.m. Red font indicates significant results (p < 0.05), orange font indicates trends (0.1 < p < 0.05). Download Table 6-1, DOCX file. [file sup_enu-eN-CFN-0046-18-s03.docx]

# Extended Tables

Extended Table 6-1

| **Gait analysis** |  |  |  |  |  |  |  |  |  |  |  |  |  |  |  |  |  |  |  |  |  |  |  |
| --- | --- | --- | --- | --- | --- | --- | --- | --- | --- | --- | --- | --- | --- | --- | --- | --- | --- | --- | --- | --- | --- | --- | --- |
|  | Cohort 1 | | | | | | | | | | |  | Cohort 2 | | | | | | | | | | |
|  | test | data structure | WT | Het | KO | genotype | | | pairwise comparisons | | |  | test | data structure | WT | Het | KO | genotype | | | pairwise comparisons | | |
|  |  |  |  |  |  | F | p-value | power | WT vs Het | WT vs KO | Het vs KO |  |  |  |  |  |  | F | p-value | power | WT vs Het | WT vs KO | Het vs KO |
| Stance Mean (cm) | ANOVA | normal | 3.74 ± 0.1 | 3.81 ± 0.1 | 3.68 ± 0.22 | 0.171 | 0.844 | 0.074 | - | - | - |  | ANOVA | normal | 3.3 ± 0.15 | 2.96 ± 0.1 | 2.9 ± 0.07 | 3.397 | **0.050** | 0.583 | 0.120 | *0.054* | 0.929 |
| Stance Variance (cm) | Kruskal-Wallis | non normal | 0.21 ± 0.07 | 0.23 ± 0.06 | 0.31 ± 0.09 | 2.875 | 0.238 | NA | - | - | - |  | ANOVA | non normal | 0.08 ± 0.02 | 0.06 ± 0 | 0.05 ± 0.01 | 5.330 | *0.070* | NA | - | - | - |
| Stride Mean (cm) | ANOVA | normal | 5 ± 0.28 | 5.33 ± 0.31 | 5.77 ± 0.38 | 1.379 | 0.269 | 0.271 | - | - | - |  | ANOVA | normal | 5.94 ± 0.32 | 5.99 ± 0.26 | 7.02 ± 0.18 | 6.191 | **0.007** | 0.849 | 0.986 | **0.015** | **0.018** |
| Stride Variance (cm) | Kruskal-Wallis | non normal | 0.81 ± 0.15 | 1.08 ± 0.2 | 1.41 ± 0.27 | 4.054 | 0.132 | NA | - | - | - |  | ANOVA | non normal | 0.72 ± 0.26 | 0.61 ± 0.16 | 0.31 ± 0.04 | 3.198 | 0.202 | NA | - | - | - |
| Sway Mean (cm) | ANOVA | normal | 2.88 ± 0.08 | 2.94 ± 0.08 | 2.67 ± 0.11 | 2.088 | 0.144 | 0.391 | - | - | - |  | ANOVA | normal | 4.12 ± 0.23 | 4.23 ± 0.1 | 4.42 ± 0.12 | 0.931 | 0.408 | 0.192 | - | - | - |
| Sway Variance (cm) | Kruskal-Wallis | non normal | 0.07 ± 0.02 | 0.12 ± 0.03 | 0.11 ± 0.02 | 1.663 | 0.435 | NA | - | - | - |  | ANOVA | non normal | 0.1 ± 0.01 | 0.23 ± 0.08 | 0.17 ± 0.03 | 0.971 | 0.615 | MA | - | - | - |
|  |  |  |  |  |  |  |  |  |  |  |  |  |  |  |  |  |  |  |  |  |  |  |  |
| **Open field spontaneous activity (traveled distance)** | |  |  |  |  |  |  |  |  |  |  |  |  |  |  |  |  |  |  |  |  |  |  |
|  | Cohort 1 | | | | | | | | | | |  | Cohort 2 | | | | | | | | | | |
|  | test | data structure | WT | Het | KO | genotype | | | pairwise comparisons | | |  | test | data structure | WT | Het | KO | genotype | | | pairwise comparisons | | |
|  |  |  |  |  |  | F | p-value | power | WT vs Het | WT vs KO | Het vs KO |  |  |  |  |  |  | F | p-value | power | WT vs Het | WT vs KO | Het vs KO |
| Total Distance (cm) | ANOVA | normal | 14842.53 ± 842.39 | 12776.12 ± 854 | 11626.96 ± 949.7 | 3.477 | **0.045** | 0.600 | 0.223 | **0.041** | 0.645 |  | ANOVA | non normal | 12404.92 ± 1518 | 9603.21 ± 1095.11 | 8724.29 ± 549.97 | 5.343 | *0.069* | NA | - | - | - |
|  |  |  |  |  |  |  |  |  |  |  |  |  |  |  |  |  |  |  |  |  |  |  |  |
| Distance repeated measures | test | data structure |  | | | F | p-value | power | WT vs Het | WT vs KO | Het vs KO |  | test | data structure |  | | | F | p-value | power | WT vs Het | WT vs KO | Het vs KO |
| - time effect | repeated measures | sphericity violated |  | | | 21.880 | **0.000** | 1.000 | - | - | - |  | repeated measures | sphericity violated |  |  |  | 15.819 | **0.000** | 1.000 | - | - | - |
| - time x genotype effect | repeated measures | sphericity violated |  |  |  | 1.280 | 0.248 | 0.639 | - | - | - |  | repeated measures | sphericity violated |  |  |  | 1.902 | *0.051* | 0.839 | - | - | - |
| - genotype effect | repeated measures | sphericity violated |  |  |  | 3.477 | **0.045** | 0.600 | 0.223 | **0.041** | 0.645 |  | repeated measures | sphericity violated |  |  |  | 3.148 | *0.061* | 0.549 | 0.187 | *0.056* | 0.821 |
|  |  |  |  |  |  |  |  |  |  |  |  |  |  |  |  |  |  |  |  |  |  |  |  |
| Individual time bins | test | data structure | WT | Het | KO | genotype | | | pairwise comparisons | | |  | test | data structure | WT | Het | KO | genotype | | | pairwise comparisons | | |
|  |  |  |  |  |  | F | p-value | power | WT vs Het | WT vs KO | Het vs KO |  |  |  |  |  |  | F | p-value | power | WT vs Het | WT vs KO | Het vs KO |
| Distance 0-10 min | ANOVA | normal | 3021.13 ± 171.29 | 2566.9 ± 170.97 | 2714.18 ± 220.39 | 1.634 | 0.214 | 0.314 | - | - | - |  | ANOVA | normal | 2313.21 ± 332.53 | 2140.91 ± 288.17 | 2268.55 ± 230.97 | 0.099 | 0.907 | 0.063 | - | - | - |
| Distance 10-20 min | ANOVA | normal | 2756.41 ± 142.2 | 2347.03 ± 240.98 | 1954.99 ± 167.6 | 4.553 | **0.020** | 0.725 | 0.271 | **0.015** | 0.335 |  | ANOVA | normal | 2187.26 ± 266.6 | 1750.22 ± 202.12 | 1441.78 ± 90.86 | 3.872 | **0.035** | 0.643 | 0.269 | **0.027** | 0.472 |
| Distance 20-30 min | ANOVA | normal | 2511.25 ± 182.35 | 2244.49 ± 211.77 | 1834.94 ± 255.02 | 2.480 | 0.103 | 0.455 | - | - | - |  | ANOVA | non normal | 2127.14 ± 308.48 | 1557.91 ± 184.05 | 1135.83 ± 93.18 | 8.210 | **0.016** | NA | 0.199 | **0.004** | 0.114 |
| Distance 30-40 min | ANOVA | normal | 2413.69 ± 163.46 | 1923.41 ± 185.22 | 1824.79 ± 155.82 | 3.568 | **0.042** | 0.612 | 0.111 | *0.054* | 0.916 |  | ANOVA | normal | 1913.95 ± 214.95 | 1409.75 ± 193.11 | 1377.57 ± 139.5 | 2.620 | 0.093 | 0.471 | - | - | - |
| Distance 40-50 min | ANOVA | normal | 2237.14 ± 195.11 | 1865.45 ± 164.72 | 1565.98 ± 188.89 | 3.320 | *0.051* | 0.579 | 0.327 | **0.042** | 0.512 |  | ANOVA | normal | 1888.57 ± 255.25 | 1426.38 ± 214.91 | 1212.61 ± 126.95 | 2.957 | *0.071* | 0.522 | 0.263 | *0.061* | 0.715 |
| Distance 50-60 min | ANOVA | normal | 1902.88 ± 224.52 | 1828.84 ± 79.9 | 1732.04 ± 193.24 | 0.222 | 0.803 | 0.081 | - | - | - |  | ANOVA | non normal | 1974.76 ± 240.97 | 1318.02 ± 145.04 | 1287.9 ± 183.22 | 6.543 | **0.038** | 0.651 | **0.035** | **0.018** | 0.829 |
|  |  |  |  |  |  |  |  |  |  |  |  |  |  |  |  |  |  |  |  |  |  |  |  |
| **Rotarod** |  |  |  |  |  |  |  |  |  |  |  |  |  |  |  |  |  |  |  |  |  |  |  |
|  | Cohort 1 | | | | | | | | | | |  | Cohort 2 | | | | | | | | | | |
| Latency, repeated measures | test | data structure |  | | | F | p-value | power | WT vs Het | WT vs KO | Het vs KO |  | test | data structure |  | | | F | p-value | power | WT vs Het | WT vs KO | Het vs KO |
| - trial effect | repeated measures | sphericity violated |  | | | 9.765 | **0.000** | 1.000 | - | - | - |  | repeated measures | sphericity violated |  | | | 2.793 | **0.020** | 0.818 | - | - | - |
| - trial x genotype effect | repeated measures | sphericity violated |  |  |  | 0.940 | 0.499 | 0.479 | - | - | - |  | repeated measures | sphericity violated |  |  |  | 3.120 | **0.001** | 0.980 | - | - | - |
| - genotype effect | repeated measures | sphericity violated |  |  |  | 5.674 | **0.009** | 0.821 | 0.120 | **0.007** | 0.408 |  | repeated measures | sphericity violated |  |  |  | 4.210 | **0.027** | 0.682 | 0.897 | **0.036** | *0.080* |
|  |  |  |  |  |  |  |  |  |  |  |  |  |  |  |  |  |  |  |  |  |  |  |  |
| Individual trials | test | data structure | WT | Het | KO | genotype | | | pairwise comparisons | | |  | test | data structure | WT | Het | KO | genotype | | | pairwise comparisons | | |
|  |  |  |  |  |  | F | p-value | power | WT vs Het | WT vs KO | Het vs KO |  |  |  |  |  |  | F | p-value | power | WT vs Het | WT vs KO | Het vs KO |
| Latency trial 1 | ANOVA | normal | 189.29 ± 16.93 | 155.6 ± 16.77 | 155.2 ± 21.3 | 1.205 | 0.315 | 0.241 | - | - | - |  | ANOVA | normal | 170.27 ± 28.77 | 197.63 ± 23.28 | 143.74 ± 17.22 | 1.474 | 0.249 | 0.283 | - | - | - |
| Latency trial 2 | ANOVA | normal | 232.92 ± 27.64 | 166.34 ± 21 | 119.28 ± 19.03 | 5.901 | **0.007** | 0.836 | 0.121 | **0.006** | 0.368 |  | ANOVA | normal | 151.56 ± 17.15 | 217.88 ± 34.88 | 150.74 ± 24.69 | 2.020 | 0.155 | 0.375 | - | - | - |
| Latency trial 3 | ANOVA | normal | 245.65 ± 19.73 | 183.15 ± 20.73 | 126.5 ± 20.72 | 8.498 | **0.001** | 0.946 | 0.086 | **0.001** | 0.155 |  | Kruskal-Wallis | non normal | 191.26 ± 41.01 | 172.9 ± 21.1 | 131.13 ± 20.04 | 1.876 | 0.391 | NA | - | - | - |
| Latency trial 4 | ANOVA | normal | 277.17 ± 22.9 | 230.13 ± 25.94 | 186.03 ± 27.04 | 3.267 | *0.054* | 0.572 | 0.379 | **0.043** | 0.460 |  | ANOVA | normal | 238.58 ± 31.46 | 187.02 ± 13.57 | 151.8 ± 21.24 | 3.681 | **0.040** | 0.620 | 0.277 | **0.032** | 0.502 |
| Latency trial 5 | ANOVA | normal | 297.26 ± 31.49 | 219.55 ± 39.79 | 199.22 ± 36.15 | 2.151 | 0.136 | 0.402 | - | - | - |  | ANOVA | normal | 234.41 ± 24.11 | 239.41 ± 18.6 | 148.54 ± 24.3 | 5.363 | **0.012** | 0.791 | 0.987 | **0.035** | **0.020** |
| Latency trial 6 | Kruskal-Wallis | non normal | 294.34 ± 27.18 | 233.2 ± 41.05 | 169.32 ± 20.66 | 6.667 | **0.036** | NA | 0.135 | **0.011** | 0.280 |  | ANOVA | normal | 244.87 ± 24.24 | 146.83 ± 23.18 | 101.83 ± 18.52 | 10.887 | **0.000** | 0.981 | 0.013 | **0.000** | 0.309 |
| Latency day 1 | ANOVA | normal | 222.62 ± 17.14 | 168.36 ± 17.13 | 133.66 ± 16.31 | 7.020 | **0.004** | 0.897 | 0.071 | **0.003** | 0.352 |  | ANOVA | normal | 171.03 ± 24.52 | 196.14 ± 23.6 | 141.87 ± 17.93 | 1.637 | 0.216 | 0.311 | - | - | - |
| Latency day 2 | ANOVA | normal | 289.59 ± 22.22 | 227.62 ± 33.92 | 184.86 ± 21.91 | 3.911 | **0.032** | 0.655 | 0.231 | **0.027** | 0.521 |  | ANOVA | normal | 239.29 ± 22.83 | 191.09 ± 14.23 | 134.06 ± 16.84 | 8.629 | **0.002** | 0.946 | 0.176 | **0.001** | *0.074* |
|  |  |  |  |  |  |  |  |  |  |  |  |  |  |  |  |  |  |  |  |  |  |  |  |
| **Beam walking** |  |  |  |  |  |  |  |  |  |  |  |  |  |  |  |  |  |  |  |  |  |  |  |
|  | Cohort 1 | | | | | | | | | | |  | Cohort 2 | | | | | | | | | | |
|  | test | data structure | WT | Het | KO | genotype | | | pairwise comparisons | | |  | test | data structure | WT | Het | KO | genotype | | | pairwise comparisons | | |
|  |  |  |  |  |  | F | p-value | power | WT vs Het | WT vs KO | Het vs KO |  |  |  |  |  |  | F | p-value | power | WT vs Het | WT vs KO | Het vs KO |
| Percentage of mice falling of large beam | NA | NA | 0 ± 0 | 0 ± 0 | 0 ± 0 | NA | NA | NA |  |  |  |  |  | NA | 0 ± 0 | 0 ± 0 | 0 ± 0 | NA | NA | NA | - | - | - |
| Percentage of mice falling of medium beam | Kruskal-Wallis | non normal | 0 ± 0 | 0 ± 0 | 8.33 ± 4.16 | 7.519 | **0.023** | NA | 1.000 | **0.015** | **0.017** |  | Kruskal-Wallis | non normal | 0 ± 0 | 0 ± 0 | 5 ± 3.33 | 3.536 | 0.171 | NA | - | - | - |
| Percentage of mice falling of small beam | Kruskal-Wallis | non normal | 20.45 ± 5.65 | 25 ± 8.33 | 72.22 ± 11.36 | 10.854 | **0.004** | NA | 0.796 | **0.002** | **0.007** |  | Kruskal-Wallis | non normal | 50 ± 14.17 | 27.77 ± 12.8 | 85 ± 5.52 | 9.218 | **0.010** | NA | 0.287 | 0.070 | **0.003** |
|  |  |  |  |  |  |  |  |  |  |  |  |  |  |  |  |  |  |  |  |  |  |  |  |
| Distance crossed on large beam (cm) | Kruskal-Wallis | non normal | 98.27 ± 1.72 | 92.5 ± 5.33 | 100 ± 0 | 2.209 | 0.331 | NA | - | - | - |  | Kruskal-Wallis | non normal | 90.62 ± 4.57 | 89.17 ± 8.18 | 98.23 ± 1.76 | 2.134 | 0.344 | NA | - | - | - |
| Distance crossed on medium beam (cm) | Kruskal-Wallis | non normal | 83.61 ± 6.25 | 83.47 ± 7.6 | 87.09 ± 8.89 | 0.662 | 0.718 | NA | - | - | - |  | Kruskal-Wallis | non normal | 92.06 ± 5.28 | 98.12 ± 1.87 | 89.11 ± 5.74 | 1.392 | 0.499 | NA | - | - | - |
| Distance crossed on small beam (cm) | ANOVA | normal | 51.65 ± 7.22 | 51.91 ± 11.04 | 31.01 ± 9.69 | 1.562 | 0.228 | 0.302 | - | - | - |  | Kruskal-Wallis | non normal | 41.07 ± 8.36 | 55.83 ± 12.36 | 21.96 ± 7.98 | 7.273 | **0.026** | NA | 0.671 | **0.045** | **0.012** |
|  |  |  |  |  |  |  |  |  |  |  |  |  |  |  |  |  |  |  |  |  |  |  |  |
| Percentage of mice fully crossing large beam | Kruskal-Wallis | non normal | 97.72 ± 2.27 | 92.5 ± 5.33 | 100 ± 0 | 2.126 | 0.345 | NA | - | - | - |  | Kruskal-Wallis | non normal | 90.62 ± 4.57 | 86.11 ± 11.11 | 97.5 ± 2.5 | 1.741 | 0.419 | NA | - | - | - |
| Percentage of mice fully crossing medium beam | Kruskal-Wallis | non normal | 70.45 ± 9.42 | 65.83 ± 11.61 | 77.77 ± 11.36 | 0.687 | 0.709 | NA | - | - | - |  | Kruskal-Wallis | non normal | 84.37 ± 10.49 | 97.22 ± 2.77 | 82.5 ± 9.89 | 1.259 | 0.533 | NA | - | - | - |
| Percentage of mice fully crossing small beam | Kruskal-Wallis | non normal | 34.09 ± 10.26 | 37.5 ± 11.93 | 13.88 ± 11.11 | 3.244 | 0.197 | NA | - | - | - |  | Kruskal-Wallis | non normal | 18.75 ± 9.14 | 39.41 ± 16.04 | 5 ± 3.33 | 3.709 | 0.157 | NA | - | - | - |
|  |  |  |  |  |  |  |  |  |  |  |  |  |  |  |  |  |  |  |  |  |  |  |  |
| Number of paw misplacement on the large beam (all mice) | Kruskal-Wallis | non normal | 0.47 ± 0.23 | 0.7 ± 0.29 | 0.8 ± 0.18 | 4.278 | 0.118 | NA | - | - | - |  | ANOVA | normal | 0.46 ± 0.14 | 0.41 ± 0.13 | 1.29 ± 0.19 | 9.388 | **0.001** | 0.962 | 0.830 | **0.002** | **0.001** |
| Number of paw misplacement on the medium beam (all mice) | ANOVA | normal | 1.56 ± 0.39 | 1.45 ± 0.35 | 2.16 ± 0.27 | 1.098 | 0.348 | 0.222 | - | - | - |  | ANOVA | normal | 1.9 ± 0.47 | 1.55 ± 0.41 | 2.57 ± 0.75 | 0.804 | 0.459 | 0.171 | - | - | - |
| Number of paw misplacement on the small beam (all mice) | Kruskal-Wallis | non normal | 1.38 ± 0.16 | 1.92 ± 0.38 | 1.44 ± 0.23 | 1.161 | 0.560 | NA | - | - | - |  | Kruskal-Wallis | non normal | 1.53 ± 0.22 | 3.11 ± 0.88 | 2.1 ± 0.25 | 3.900 | 0.142 | NA | - | - | - |
|  |  |  |  |  |  |  |  |  |  |  |  |  |  |  |  |  |  |  |  |  |  |  |  |
| Number of paw misplacement on large beam (fully crossing mice) | Kruskal-Wallis | non normal | 0.52 ± 0.27 | 0.7 ± 0.29 | 0.8 ± 0.18 | 4.166 | 0.125 | NA | - | - | - |  | ANOVA | normal | 0.52 ± 0.16 | 0.46 ± 0.14 | 1.3 ± 0.2 | 7.245 | **0.004** | 0.900 | 0.979 | **0.013** | **0.008** |
| Number of paw misplacement on medium beam (fully crossing mice) | ANOVA | normal | 1.56 ± 0.38 | 1.11 ± 0.31 | 2.07 ± 0.3 | 1.073 | 0.373 | 0.321 | - | - | - |  | ANOVA | normal | 1.84 ± 0.61 | 1.64 ± 0.44 | 2.55 ± 0.95 | 0.440 | 0.649 | 0.113 | - | - | - |
| Number of paw misplacement on small beam (fully crossing mice) | Kruskal-Wallis | non normal | 1.55 ± 0.27 | 2.5 ± 0.63 | 2.12 ± 1.12 | 1.962 | 0.375 | NA | - | - | - |  | ANOVA | normal | 2.33 ± 0.33 | 3.18 ± 1 | 3.5 ± 0.5 | 0.447 | 0.659 | 0.095 | - | - | - |
|  |  |  |  |  |  |  |  |  |  |  |  |  |  |  |  |  |  |  |  |  |  |  |  |
| Time to cross large beam (fully crossing mice) | Kruskal-Wallis | non normal | 10.37 ± 2.01 | 11.76 ± 3.24 | 7.91 ± 1.84 | 1.214 | 0.545 | NA | - | - | - |  | Kruskal-Wallis | non normal | 9.6 ± 1.56 | 5.8 ± 0.57 | 6.5 ± 1.5 | 7.063 | **0.029** | NA | **0.019** | **0.020** | 0.939 |
| Time to cross large beam (fully crossing mice) | Kruskal-Wallis | non normal | 38.21 ± 6.71 | 19.5 ± 5.66 | 16.3 ± 6.38 | 4.729 | *0.094* | NA | - | - | - |  | ANOVA | normal | 15.25 ± 6.39 | 13.75 ± 3.4 | 19.61 ± 6.49 | 0.307 | 0.738 | 0.093 | - | - | - |
| Time to cross large beam (fully crossing mice) | ANOVA | normal | 43.76 ± 5.82 | 51.31 ± 3.98 | 24.37 ± 14.37 | 2.828 | *0.099* | 0.451 | 0.347 | 0.107 | **0.035** |  | ANOVA | normal | 67.16 ± 12.67 | 36.85 ± 9.96 | 20.5 ± 1.5 | 3.186 | *0.085* | 0.478 | *0.096* | **0.044** | 0.387 |
|  |  |  |  |  |  |  |  |  |  |  |  |  |  |  |  |  |  |  |  |  |  |  |  |
| Time to cross the large beam (all mice, 120 seconds cut-off) | Kruskal-Wallis | non normal | 12.7 ± 3.58 | 20 ± 6.46 | 7.91 ± 1.84 | 1.750 | 0.417 | NA | - | - | - |  | Kruskal-Wallis | non normal | 19.93 ± 5.29 | 21.58 ± 12.77 | 9.02 ± 3.97 | 6.707 | **0.035** | NA | *0.090* | **0.010** | 0.392 |
| Time to cross the large beam (all mice, 120 seconds cut-off) | Kruskal-Wallis | non normal | 58.7 ± 10.41 | 54 ± 13.51 | 37.33 ± 13.58 | 1.351 | 0.509 | NA | - | - | - |  | Kruskal-Wallis | non normal | 29.34 ± 13.51 | 16.25 ± 5.29 | 32.17 ± 12.43 | 0.486 | 0.784 | NA | - | - | - |
| Time to cross the large beam (all mice, 120 seconds cut-off) | ANOVA | normal | 95.56 ± 7.15 | 93.8 ± 8.76 | 107.91 ± 9.16 | 0.808 | 0.456 | 0.173 | - | - | - |  | ANOVA | normal | 105.91 ± 4.92 | 85.75 ± 13.49 | 115.02 ± 3.31 | 3.250 | *0.056* |  | 0.251 | 0.733 | **0.049** |
|  |  |  |  |  |  |  |  |  |  |  |  |  |  |  |  |  |  |  |  |  |  |  |  |
| **Motor reflexes** |  |  |  |  |  |  |  |  |  |  |  |  |  |  |  |  |  |  |  |  |  |  |  |
|  | Cohort 1 | | | | | | | | | | |  | Cohort 2 | | | | | | | | | | |
|  | test | data structure | WT | Het | KO | genotype | | | pairwise comparisons | | |  | test | data structure | WT | Het | KO | genotype | | | pairwise comparisons | | |
|  |  |  |  |  |  | F | p-value | power | WT vs Het | WT vs KO | Het vs KO |  |  |  |  |  |  | F | p-value | power | WT vs Het | WT vs KO | Het vs KO |
| Righting Reflex | Kruskal-Wallis | non normal | 0.09 ± 0.09 | 0 ± 0 | 0 ± 0 | 1.727 | 0.422 | NA | - | - | - |  | - | - | 0 ± 0 | 0 ± 0 | 0 ± 0 | - | - | NA | - | - | - |
| Hindlimb placing, score | Kruskal-Wallis | non normal | 5.63 ± 0.36 | 5.2 ± 0.53 | 4.44 ± 0.86 | 1.946 | 0.378 | NA | - | - | - |  | Kruskal-Wallis | non normal | 5.5 ± 0.32 | 5.33 ± 0.47 | 4 ± 0.78 | 2.635 | 0.268 | NA | - | - | - |
| Hindlimb placing, latency to climb | Kruskal-Wallis | non normal | 7.93 ± 2.13 | 7.06 ± 2.43 | 9.4 ± 4.01 | 1.375 | 0.503 | NA | - | - | - |  | Kruskal-Wallis | non normal | 8.79 ± 2.63 | 7.37 ± 2.4 | 12.36 ± 3.43 | 1.741 | 0.419 | NA | - | - | - |
| Hindlimb placing, failed attempts | Kruskal-Wallis | non normal | 0.18 ± 0.18 | 0.4 ± 0.26 | 0.77 ± 0.43 | 1.946 | 0.378 | NA | - | - | - |  | Kruskal-Wallis | non normal | 0.25 ± 0.16 | 0.33 ± 0.23 | 1 ± 0.39 | 2.635 | 0.268 | NA | - | - | - |
| Inverted screen, latency to fall | Kruskal-Wallis | non normal | 37.9 ± 7.82 | 35.2 ± 6.45 | 12.66 ± 6.44 | 8.321 | **0.016** | NA | 0.950 | **0.011** | **0.011** |  | Kruskal-Wallis | non normal | 28.12 ± 5.51 | 39 ± 7.27 | 5.7 ± 1.23 | 14.074 | **0.001** | NA | 0.536 | **0.006** | **0.000** |
| Hanging, score | Kruskal-Wallis | non normal | 6.27 ± 0.14 | 5.6 ± 0.26 | 4.55 ± 0.37 | 13.107 | **0.001** | NA | *0.079* | **0.000** | *0.062* |  | Kruskal-Wallis | non normal | 6.25 ± 0.41 | 6.44 ± 0.41 | 5.1 ± 0.4 | 5.419 | *0.075* | NA | - | - | - |
| Hanging, latency to fall | ANOVA | normal | 26.69 ± 2.22 | 21.46 ± 3.52 | 7.33 ± 1.48 | 17.076 | **0.000** | 0.997 | 0.240 | **0.000** | **0.004** |  | Kruskal-Wallis | non normal | 23.71 ± 4.6 | 26.11 ± 4.09 | 9.52 ± 1.49 | 10.011 | **0.007** | NA | 0.695 | **0.015** | **0.004** |
|  |  |  |  |  |  |  |  |  |  |  |  |  |  |  |  |  |  |  |  |  |  |  |  |
| **Grip strength** |  |  |  |  |  |  |  |  |  |  |  |  |  |  |  |  |  |  |  |  |  |  |  |
|  | Cohort 1 | | | | | | | | | | |  | Cohort 2 | | | | | | | | | | |
| Latency, repeated measure | test | data structure |  | | | F | p-value | power | WT vs Het | WT vs KO | Het vs KO |  | test | data structure |  | | | F | p-value | power | WT vs Het | WT vs KO | Het vs KO |
| - session effect | repeated measures | sphericity violated |  | | | 2.270 | 0.113 | 0.442 | - | - | - |  | repeated measures | sphericity violated |  | | | 1.737 | 0.187 | 0.347 | - | - | - |
| - session x genotype effect | repeated measures | sphericity violated |  |  |  | 1.208 | 0.318 | 0.353 | - | - | - |  | repeated measures | sphericity violated |  |  |  | 4.538 | **0.003** | 0.920 | - | - | - |
| - genotype effect | repeated measures | sphericity violated |  |  |  | 1.028 | 0.371 | 0.210 | - | - | - |  | repeated measures | sphericity violated |  |  |  | 0.161 | 0.853 | 0.072 | - | - | - |
|  |  |  |  |  |  |  |  |  |  |  |  |  |  |  |  |  |  |  |  |  |  |  |  |
| Individual trials | test | data structure | WT | Het | KO | genotype | | | pairwise comparisons | | |  | test | data structure | WT | Het | KO | genotype | | | pairwise comparisons | | |
|  |  |  |  |  |  | F | p-value | power | WT vs Het | WT vs KO | Het vs KO |  |  |  |  |  |  | F | p-value | power | WT vs Het | WT vs KO | Het vs KO |
| Session 1 |  | normal | 0.88 ± 0.07 | 0.81 ± 0.08 | 0.85 ± 0.06 | 0.203 | 0.817 | 0.078 | - | - | - |  |  | normal | 1.03 ± 0.04 | 1.2 ± 0.05 | 1.19 ± 0.06 | 2.567 | *0.098* | 0.463 | 0.124 | 0.151 | 0.986 |
| Session 2 |  | normal | 0.67 ± 0.07 | 0.66 ± 0.05 | 0.9 ± 0.07 | 3.387 | **0.049** | 0.588 | 0.994 | *0.079* | *0.072* |  |  | normal | 1.12 ± 0.07 | 1.06 ± 0.08 | 1.11 ± 0.05 | 0.182 | 0.835 | 0.075 | - | - | - |
| Session 3 |  | normal | 0.73 ± 0.06 | 0.75 ± 0.07 | 0.82 ± 0.11 | 0.250 | 0.781 | 0.085 | - | - | - |  |  | normal | 1.16 ± 0.07 | 1.11 ± 0.05 | 0.95 ± 0.06 | 2.923 | *0.073* | 0.517 | 0.847 | *0.079* | 0.202 |
| Mean strength |  | normal | 0.76 ± 0.05 | 0.74 ± 0.05 | 0.86 ± 0.06 | 1.028 | 0.371 | 0.210 | - | - | - |  |  | normal | 1.1 ± 0.04 | 1.12 ± 0.05 | 1.08 ± 0.04 | 0.161 | 0.853 | 0.072 | - | - | - |
| highest score |  | non normal | 0.94 ± 0.06 | 0.93 ± 0.06 | 1.04 ± 0.07 | 1.504 | 0.471 | NA | - | - | - |  |  | normal | 1.27 ± 0.05 | 1.24 ± 0.04 | 1.22 ± 0.06 | 0.210 | 0.812 | 0.079 | - | - | - |
