# Supplementary material for: Behavioral Phenotyping of an Improved Mouse Model of Phelan–McDermid Syndrome with a Complete Deletion of the Shank3 Gene
Source: eNeuro. 2018 Oct 5;5(3):ENEURO.0046-18.2018. doi: 10.1523/ENEURO.0046-18.2018 (PMC6175061; doi:10.1523/ENEURO.0046-18.2018)
Supplement: Extended Data Table 7-1 — Individual results and statistical analyses for cohorts 1 and 2 related to the sensory profile. WT, wild-type mice; Het, heterozygous mice; KO, homozygous knockout mice. Group values are reported as means ± s.e.m. Red font indicates significant results (p < 0.05), orange font indicates trends (0.1 < p < 0.05). Download Table 7-1, DOCX file. [file sup_enu-eN-CFN-0046-18-s04.docx]

# Extended Tables

Extended Table 7-1

| **Reflexes and reactions to simple stimuli** |  |  |  |  |  |  |  |  |  |  |  |  |  |  |  |  |  |  |  |  |  |  |  |
| --- | --- | --- | --- | --- | --- | --- | --- | --- | --- | --- | --- | --- | --- | --- | --- | --- | --- | --- | --- | --- | --- | --- | --- |
|  | Cohort 1 | | | | | | | | | | |  | Cohort 2 | | | | | | | | | | |
|  | test | data structure | WT | Het | KO | genotype | | | pairwise comparisons | | |  | test | data structure | WT | Het | KO | genotype | | | pairwise comparisons | | |
|  |  |  |  |  |  | F | p-value | power | WT vs Het | WT vs KO | Het vs KO |  |  |  |  |  |  | F | p-value | power | WT vs Het | WT vs KO | Het vs KO |
| Pinna reflex | Kruskal-Wallis | non normal | 0.81 ± 0.12 | 0.5 ± 0.16 | 0.44 ± 0.17 | 3.389 | 0.1840 | NA | - | - | - |  | Kruskal-Wallis | non normal | 1 ± 0 | 0.88 ± 0.11 | 1 ± 0 | 2.000 | 0.3680 | NA | - | - | - |
| Cornel reflex | NA | NA | 1 ± 0 | 1 ± 0 | 1 ± 0 | - | - | - | - | - | - |  | Kruskal-Wallis | non normal | 1.12 ± 0.12 | 0.88 ± 0.11 | 1.1 ± 0.1 | 2.594 | 0.2730 | NA | - | - | - |
| Toe pinch retractation | Kruskal-Wallis | non normal | 1.9 ± 0.31 | 2.2 ± 0.53 | 1.88 ± 0.51 | 0.094 | 0.9540 | NA | - | - | - |  | Kruskal-Wallis | non normal | 2.25 ± 0.79 | 2.55 ± 0.41 | 2.6 ± 0.7 | 0.696 | 0.7060 | NA | - | - | - |
| Preyer reflex | Kruskal-Wallis | non normal | 1.72 ± 0.23 | 1.6 ± 0.22 | 2 ± 0.23 | 1.580 | 0.4540 | NA | - | - | - |  | Kruskal-Wallis | non normal | 1.12 ± 0.12 | 1.11 ± 0.11 | 0.9 ± 0.1 | 2.601 | 0.2200 | NA | - | - | - |
| Visual Placing/Reaching reflex | NA | NA | 9 ± 0 | 9 ± 0 | 9 ± 0 | - | - | - | - | - | - |  | NA | NA | 9 ± 0 | 9 ± 0 | 9 ± 0 | - | - |  | - | - | - |
|  |  |  |  |  |  |  |  |  |  |  |  |  |  |  |  |  |  |  |  |  |  |  |  |
| **Tail flick** |  |  |  |  |  |  |  |  |  |  |  |  |  |  |  |  |  |  |  |  |  |  |  |
|  | Cohort 1 | | | | | | | | | | |  | Cohort 2 | | | | | | | | | | |
| Latency, repeated measures | test | data structure |  | | | F | p-value | power | WT vs Het | WT vs KO | Het vs KO |  | test | data structure |  | | | F | p-value | power | WT vs Het | WT vs KO | Het vs KO |
| - trial effect | repeated measures | sphericity violated |  | | | 1.263 | 0.2910 | 0.263 | - | - | - |  | repeated measures | sphericity violated |  | | | 10.642 | **0.0001** | 0.985 | - | - | - |
| - trial x genotype effect | repeated measures | sphericity violated |  |  |  | 0.450 | 0.7717 | 0.148 | - | - | - |  | repeated measures | sphericity violated |  |  |  | 0.207 | 0.9335 | 0.091 | - | - | - |
| - genotype effect | repeated measures | sphericity violated |  |  |  | 2.696 | *0.0856* | 0.488 | 0.716 | *0.073* | 0.304 |  | repeated measures | sphericity violated |  |  |  | 0.136 | 0.8736 | 0.069 | - | - | - |
|  |  |  |  |  |  |  |  |  |  |  |  |  |  |  |  |  |  |  |  |  |  |  |  |
| Individual trials | test | data structure | WT | Het | KO | genotype | | | pairwise comparisons | | |  | test | data structure | WT | Het | KO | genotype | | | pairwise comparisons | | |
|  |  |  |  |  |  | F | p-value | power | WT vs Het | WT vs KO | Het vs KO |  |  |  |  |  |  | F | p-value | power | WT vs Het | WT vs KO | Het vs KO |
| Latency to flick, trial 1 (seconds) | ANOVA | normal | 12.93 ± 1.26 | 13.13 ± 1.14 | 11.7 ± 1.36 | 0.356 | 0.7039 | 0.101 | - | - | - |  | Kruskal-Wallis | non normal | 9.27 ± 1.23 | 9.31 ± 0.59 | 9 ± 0.85 | 0.108 | 0.9480 | NA | - | - | - |
| Latency to flick, trial 2 (seconds) | ANOVA | normal | 14.11 ± 1.01 | 12.23 ± 0.99 | 10.38 ± 1.28 | 2.912 | *0.0716* | 0.521 | 0.437 | *0.058* | 0.480 |  | Kruskal-Wallis | non normal | 6.69 ± 0.82 | 6.82 ± 1.2 | 7.49 ± 0.91 | 0.744 | 0.6900 | NA | - | - | - |
| Latency to flick, trial 3 (seconds) | ANOVA | normal | 14.61 ± 1.21 | 13.82 ± 0.63 | 12.12 ± 1.02 | 1.557 | 0.2292 | 0.301 | - | - | - |  | ANOVA | normal | 6.16 ± 0.76 | 5.88 ± 0.49 | 6.75 ± 1 | 0.316 | 0.7320 | 0.095 | - | - | - |
| Shortest latency to flick (seconds) | ANOVA | normal | 10.33 ± 0.81 | 10.55 ± 1.04 | 9.18 ± 1.16 | 0.517 | 0.6020 | 0.126 | - | - | - |  | ANOVA | normal | 4.86 ± 0.59 | 5.13 ± 0.55 | 5.93 ± 0.82 | 0.653 | 0.5297 | 0.147 | - | - | - |
| Longest latency to flick (seconds) | ANOVA | normal | 16.95 ± 0.86 | 15.22 ± 0.53 | 13.89 ± 1.07 | 3.331 | *0.0509* | 0.581 | 0.314 | 0.042 | 0.528 |  | ANOVA | normal | 10.35 ± 0.64 | 10.16 ± 0.82 | 10.14 ± 0.55 | 0.028 | 0.9723 | 0.054 | - | - | - |
| Mean latency to flick (seconds) | ANOVA | normal | 13.88 ± 0.68 | 13.06 ± 0.61 | 11.4 ± 0.98 | 2.696 | *0.0856* | 0.488 | 0.716 | *0.073* | 0.304 |  | ANOVA | normal | 7.37 ± 0.61 | 7.34 ± 0.51 | 7.75 ± 0.71 | 0.136 | 0.8736 | 0.069 | - | - | - |
|  |  |  |  |  |  |  |  |  |  |  |  |  |  |  |  |  |  |  |  |  |  |  |  |
| **Startle response** |  |  |  |  |  |  |  |  |  |  |  |  |  |  |  |  |  |  |  |  |  |  |  |
|  | Cohort 1 | | | | | | | | | | |  | Cohort 2 | | | | | | | | | | |
|  | test | data structure | WT | Het | KO | genotype | | | pairwise comparisons | | |  | test | data structure | WT | Het | KO | genotype | | | pairwise comparisons | | |
|  |  |  |  |  |  | F | p-value | power | WT vs Het | WT vs KO | Het vs KO |  |  |  |  |  |  | F | p-value | power | WT vs Het | WT vs KO | Het vs KO |
| Startle response at 74 dB | Kruskal-Wallis | non normal | 154.36 ± 10.96 | 158.86 ± 13.26 | 147.29 ± 10.67 | 0.383 | 0.8260 | NA | - | - | - |  | ANOVA | normal | 273.83 ± 67.95 | 182.33 ± 55.23 | 182.12 ± 20.79 | 1.054 | 0.3925 | 0.175 | - | - | - |
| Startle response at 78 dB | Kruskal-Wallis | non normal | 155.34 ± 13.45 | 146.46 ± 13.73 | 164.92 ± 12.81 | 1.096 | 0.5780 | NA | - | - | - |  | ANOVA | normal | 286.66 ± 67.74 | 184.75 ± 50.84 | 151.16 ± 43.08 | 1.633 | 0.2543 | 0.250 | - | - | - |
| Startle response at 82 dB | Kruskal-Wallis | non normal | 170.72 ± 21.09 | 157.8 ± 12.77 | 173.48 ± 17.72 | 0.360 | 0.8350 | NA | - | - | - |  | ANOVA | normal | 295.44 ± 79.35 | 165.83 ± 42.02 | 180.33 ± 24.62 | 1.997 | 0.1979 | 0.298 | - | - | - |
| Startle response at 86 dB | Kruskal-Wallis | non normal | 237.51 ± 41.83 | 160.86 ± 12.04 | 181.79 ± 17.2 | 3.741 | 0.1760 | NA | - | - | - |  | ANOVA | normal | 278.66 ± 72.01 | 165.91 ± 51.03 | 163.45 ± 17.43 | 1.718 | 0.2395 | 0.261 | - | - | - |
| Startle response at 92 dB | Kruskal-Wallis | non normal | 243.34 ± 43.32 | 192.03 ± 18.24 | 229.03 ± 27.78 | 1.346 | 0.5100 | NA | - | - | - |  | Kruskal-Wallis | non normal | 308.22 ± 116.03 | 194.12 ± 54.75 | 139.25 ± 19.88 | 2.598 | 0.2730 | NA | - | - | - |
|  |  |  |  |  |  |  |  |  |  |  |  |  |  |  |  |  |  |  |  |  |  |  |  |
| Startle response, repeated measures | test | data structure |  | | | F | p-value | power | WT vs Het | WT vs KO | Het vs KO |  | test | data structure |  | | | F | p-value | power | WT vs Het | WT vs KO | Het vs KO |
| - sound intensity effect | repeated measures | sphericity violated |  | | | 9.550 | **0.0002** | 1.000 | - | - | - |  | repeated measures | sphericity assumed |  | | | 0.175 | 0.8957 | 0.076 | - | - | - |
| - sound intensity x genotype effect | repeated measures | sphericity violated |  |  |  | 1.392 | 0.2463 | 0.607 | - | - | - |  | repeated measures | sphericity assumed |  |  |  | 0.766 | 0.5928 | 0.230 | - | - | - |
| - genotype effect | repeated measures | sphericity violated |  |  |  | 0.730 | 0.4913 | 0.161 | - | - | - |  | repeated measures | sphericity assumed |  |  |  | 1.721 | 0.2389 | 0.262 | - | - | - |
|  |  |  |  |  |  |  |  |  |  |  |  |  |  |  |  |  |  |  |  |  |  |  |  |
|  | test | data structure | WT | Het | KO | genotype | | | pairwise comparisons | | |  | test | data structure | WT | Het | KO | genotype | | | pairwise comparisons | | |
|  |  |  |  |  |  | F | p-value | power | WT vs Het | WT vs KO | Het vs KO |  |  |  |  |  |  | F | p-value | power | WT vs Het | WT vs KO | Het vs KO |
| Startle response at 74 dB, normalized to weight | Kruskal-Wallis | non normal | 6.04 ± 0.51 | 6.02 ± 0.6 | 6.02 ± 0.41 | 0.236 | 0.8890 | NA | - | - | - |  | ANOVA | normal | 10.56 ± 2.75 | 6.59 ± 1.88 | 6.5 ± 0.55 | 1.538 | 0.2722 | 0.238 | - | - | - |
| Startle response at 78 dB, normalized to weight | Kruskal-Wallis | non normal | 6.21 ± 0.74 | 5.49 ± 0.51 | 6.76 ± 0.54 | 2.827 | 0.2430 | NA | - | - | - |  | ANOVA | normal | 10.79 ± 2.44 | 6.8 ± 1.96 | 5.43 ± 1.51 | 1.870 | 0.2156 | 0.281 | - | - | - |
| Startle response at 82 dB, normalized to weight | Kruskal-Wallis | non normal | 6.83 ± 1 | 5.86 ± 0.37 | 7.17 ± 0.8 | 0.990 | 0.6100 | NA | - | - | - |  | ANOVA | normal | 11.09 ± 2.89 | 5.96 ± 1.25 | 6.56 ± 1.09 | 2.471 | 0.1460 | 0.359 | - | - | - |
| Startle response at 86 dB, normalized to weight | Kruskal-Wallis | non normal | 9.64 ± 2.03 | 6.04 ± 0.44 | 7.54 ± 0.84 | 3.039 | 0.2190 | NA | - | - | - |  | ANOVA | normal | 10.67 ± 2.81 | 5.9 ± 1.5 | 5.89 ± 0.64 | 2.493 | 0.1440 | 0.362 | - | - | - |
| Startle response at 92 dB, normalized to weight | Kruskal-Wallis | non normal | 9.62 ± 2.02 | 7.15 ± 0.6 | 9.59 ± 1.41 | 2.113 | 0.3480 | NA | - | - | - |  | Kruskal-Wallis | non normal | 11.56 ± 4.36 | 6.93 ± 1.59 | 5.01 ± 0.72 | 2.962 | 0.2270 | NA | - | - | - |
|  |  |  |  |  |  |  |  |  |  |  |  |  |  |  |  |  |  |  |  |  |  |  |  |
| Startle response normalized, repeated measures | test | data structure |  | | | F | p-value | power | WT vs Het | WT vs KO | Het vs KO |  | test | data structure |  | | | F | p-value | power | WT vs Het | WT vs KO | Het vs KO |
| - sound intensity effect | repeated measures | sphericity violated |  | | | 8.496 | **0.0007** | 0.952 | - | - | - |  | repeated measures | sphericity violated |  | | | 0.147 | 0.9632 | 0.077 | - | - | - |
| - sound intensity x genotype effect | repeated measures | sphericity violated |  |  |  | 1.412 | 0.2441 | 0.400 | - | - | - |  | repeated measures | sphericity violated |  |  |  | 0.669 | 0.7148 | 0.254 | - | - | - |
| - genotype effect | repeated measures | sphericity violated |  |  |  | 0.974 | 0.3904 | 0.201 | - | - | - |  | repeated measures | sphericity violated |  |  |  | 2.253 | 0.1674 | 0.331 | - | - | - |
|  |  |  |  |  |  |  |  |  |  |  |  |  |  |  |  |  |  |  |  |  |  |  |  |
| **Pre-pulse inhibition** |  |  |  |  |  |  |  |  |  |  |  |  |  |  |  |  |  |  |  |  |  |  |  |
|  | Cohort 1 | | | | | | | | | | |  | Cohort 2 | | | | | | | | | | |
|  | test | data structure | WT | Het | KO | genotype | | | pairwise comparisons | | |  | test | data structure | WT | Het | KO | genotype | | | pairwise comparisons | | |
|  |  |  |  |  |  | F | p-value | power | WT vs Het | WT vs KO | Het vs KO |  |  |  |  |  |  | F | p-value | power | WT vs Het | WT vs KO | Het vs KO |
| Percentage of inhibition at 74 dB | ANOVA | normal | 25.99 ± 5.94 | 17.81 ± 8.11 | 23.3 ± 7.35 | 0.354 | 0.7048 | 0.101 | - | - | - |  | ANOVA | normal | 17.63 ± 11.11 | 12.09 ± 4.67 | -0.9 ± 8.01 | 1.399 | 0.2662 | 0.271 | - | - | - |
| Percentage of inhibition at 78 dB | ANOVA | normal | 32.3 ± 4.53 | 25.56 ± 7.83 | 35.13 ± 5.35 | 0.641 | 0.5348 | 0.146 | - | - | - |  | ANOVA | normal | 31.14 ± 9.08 | 2.93 ± 10.16 | 8.62 ± 6.04 | 2.916 | *0.0735* | 0.516 | *0.076* | 0.167 | 0.877 |
| Percentage of inhibition at 82 dB | ANOVA | normal | 28.62 ± 6.63 | 30.09 ± 6.09 | 26.19 ± 8.57 | 0.073 | 0.9293 | 0.060 | - | - | - |  | ANOVA | normal | 32.83 ± 9.89 | 12.02 ± 10.39 | 1.79 ± 6.8 | 3.001 | *0.0687* | 0.528 | 0.270 | *0.058* | 0.690 |
| Percentage of inhibition at 86 dB | ANOVA | normal | 35.85 ± 5.64 | 36.75 ± 7.74 | 42.82 ± 2.43 | 0.397 | 0.6762 | 0.107 | - | - | - |  | ANOVA | normal | 26.05 ± 17.78 | 3.58 ± 10.54 | 12.84 ± 5.42 | 0.909 | 0.4162 | 0.188 | - | - | - |
| Percentage of inhibition at 92 dB | ANOVA | normal | 48.93 ± 4.81 | 43.64 ± 6.59 | 49.37 ± 5.88 | 0.304 | 0.7406 | 0.093 | - | - | - |  | ANOVA | normal | 36.32 ± 15.53 | 24.02 ± 9.01 | 12.44 ± 9.24 | 1.127 | 0.3406 | 0.225 | - | - | - |
| Percentage of inhibition, mean | ANOVA | normal | 34.34 ± 4.57 | 30.77 ± 6.63 | 35.36 ± 4.91 | 0.191 | 0.8273 | 0.077 | - | - | - |  | ANOVA | normal | 28.79 ± 11.97 | 10.93 ± 7.85 | 6.96 ± 5.87 | 1.785 | 0.1893 | 0.336 | - | - | - |
|  |  |  |  |  |  |  |  |  |  |  |  |  |  |  |  |  |  |  |  |  |  |  |  |
| Percentage of inhibition, repeated measure | test | data structure |  | | | F | p-value | power | WT vs Het | WT vs KO | Het vs KO |  | test | data structure |  | | | F | p-value | power | WT vs Het | WT vs KO | Het vs KO |
| - sound intensity effect | repeated measures | sphericity violated |  | | | 19.656 | **0.0000** | 1.000 | - | - | - |  | repeated measures | sphericity assumed |  | | | 3.000 | **0.0222** | 0.701 | - | - | - |
| - sound intensity x genotype effect | repeated measures | sphericity violated |  |  |  | 0.716 | 0.6368 | 0.317 | - | - | - |  | repeated measures | sphericity assumed |  |  |  | 1.395 | 0.2085 | 0.526 | - | - | - |
| - genotype effect | repeated measures | sphericity violated |  |  |  | 0.191 | 0.8273 | 0.077 | - | - | - |  | repeated measures | sphericity assumed |  |  |  | 1.785 | 0.1893 | 0.336 | - | - | - |
|  |  |  |  |  |  |  |  |  |  |  |  |  |  |  |  |  |  |  |  |  |  |  |  |
| **Burried food test** |  |  |  |  |  |  |  |  |  |  |  |  |  |  |  |  |  |  |  |  |  |  |  |
|  | Cohort 1 | | | | | | | | | | |  | Cohort 2 | | | | | | | | | | |
|  | test | data structure | WT | Het | KO | genotype | | | pairwise comparisons | | |  | test | data structure | WT | Het | KO | genotype | | | pairwise comparisons | | |
|  |  |  |  |  |  | F | p-value | power | WT vs Het | WT vs KO | Het vs KO |  |  |  |  |  |  | F | p-value | power | WT vs Het | WT vs KO | Het vs KO |
| Latency to retrieve and eat food (seconds) | Kruskal-Wallis | non normal | 51.18 ± 12.98 | 95.8 ± 37.31 | 502.33 ± 141.31 | 6.100 | **0.0470** |  | 0.638 | **0.018** | *0.062* |  | Kruskal-Wallis | non normal | 51.02 ± 16.38 | 93.71 ± 30.41 | 498.44 ± 135.13 | 9.266 | **0.0100** |  | 0.252 | **0.003** | **0.061** |
|  |  |  |  |  |  |  |  |  |  |  |  |  |  |  |  |  |  |  |  |  |  |  |  |
| **Olfactory habituation/dishabituation, sniffing only** |  |  |  |  |  |  |  |  |  |  |  |  |  |  |  |  |  |  |  |  |  |  |  |
|  | Cohort 1 | | | | | | | | | | |  | Cohort 2 | | | | | | | | | | |
| Water repeated measure | test | data structure |  | | | F | p-value | power | WT vs Het | WT vs KO | Het vs KO |  | test | data structure |  | | | F | p-value | power | WT vs Het | WT vs KO | Het vs KO |
| - trial effect | repeated measures | sphericity violated |  | | | 6.222 | 0.0123 | 0.877 | - | - | - |  | repeated measures | sphericity assumed |  | | | 2.737 | *0.0753* | 0.475 | - | - | - |
| - trial x genotype effect | repeated measures | sphericity violated |  |  |  | 1.597 | 0.2128 | 0.460 | - | - | - |  | repeated measures | sphericity assumed |  |  |  | 1.262 | 0.2986 | 0.333 | - | - | - |
| - genotype effect | repeated measures | sphericity violated |  |  |  | 3.749 | **0.0366** | 0.635 | 0.379 | **0.012** | *0.083* |  | repeated measures | sphericity assumed |  |  |  | 0.386 | 0.6838 | 0.105 | - | - | - |
|  |  |  |  |  |  |  |  |  |  |  |  |  |  |  |  |  |  |  |  |  |  |  |  |
| Banana repeated measure | test | data structure |  | | | F | p-value | power | WT vs Het | WT vs KO | Het vs KO |  | test | data structure |  | | | F | p-value | power | WT vs Het | WT vs KO | Het vs KO |
| - trial effect | repeated measures | sphericity assumed |  | | | 6.088 | **0.0041** | 0.824 | - | - | - |  | repeated measures | sphericity assumed |  | | | 4.463 | **0.0169** | 0.726 | - | - | - |
| - trial x genotype effect | repeated measures | sphericity assumed |  |  |  | 2.544 | **0.0500** | 0.625 | - | - | - |  | repeated measures | sphericity assumed |  |  |  | 1.797 | 0.1456 | 0.494 | - | - | - |
| - genotype effect | repeated measures | sphericity assumed |  |  |  | 4.636 | **0.0186** | 0.734 | 0.770 | **0.009** | **0.020** |  | repeated measures | sphericity assumed |  |  |  | 1.885 | 0.1745 | 0.351 | - | - | - |
|  |  |  |  |  |  |  |  |  |  |  |  |  |  |  |  |  |  |  |  |  |  |  |  |
| Lemon repeated measure | test | data structure |  | | | F | p-value | power | WT vs Het | WT vs KO | Het vs KO |  | test | data structure |  | | | F | p-value | power | WT vs Het | WT vs KO | Het vs KO |
| - trial effect | repeated measures | sphericity assumed |  | | | 2.208 | 0.1197 | 0.412 | - | - | - |  | repeated measures | sphericity violated |  | | | 4.185 | **0.0332** | 0.708 | - | - | - |
| - trial x genotype effect | repeated measures | sphericity assumed |  |  |  | 0.714 | 0.5863 | 0.207 | - | - | - |  | repeated measures | sphericity violated |  |  |  | 0.241 | 0.8682 | 0.098 | - | - | - |
| - genotype effect | repeated measures | sphericity assumed |  |  |  | 2.692 | *0.0859* | 0.488 | 0.747 | *0.070* | **0.040** |  | repeated measures | sphericity violated |  |  |  | 0.932 | 0.4083 | 0.191 | - | - | - |
|  |  |  |  |  |  |  |  |  |  |  |  |  |  |  |  |  |  |  |  |  |  |  |  |
| Male repeated measure | test | data structure |  | | | F | p-value | power | WT vs Het | WT vs KO | Het vs KO |  | test | data structure |  | | | F | p-value | power | WT vs Het | WT vs KO | Het vs KO |
| - trial effect | repeated measures | sphericity violated |  | | | 15.578 | **0.0000** | 0.999 | - | - | - |  | repeated measures | sphericity violated |  | | | 13.289 | **0.0007** | 0.996 | - | - | - |
| - trial x genotype effect | repeated measures | sphericity violated |  |  |  | 1.894 | 0.1405 | 0.536 | - | - | - |  | repeated measures | sphericity violated |  |  |  | 0.651 | 0.5510 | 0.197 | - | - | - |
| - genotype effect | repeated measures | sphericity violated |  |  |  | 0.132 | 0.8767 | 0.068 | - | - | - |  | repeated measures | sphericity violated |  |  |  | 1.079 | 0.3567 | 0.216 | - | - | - |
|  |  |  |  |  |  |  |  |  |  |  |  |  |  |  |  |  |  |  |  |  |  |  |  |
| Female repeated measure | test | data structure |  | | | F | p-value | power | WT vs Het | WT vs KO | Het vs KO |  | test | data structure |  | | | F | p-value | power | WT vs Het | WT vs KO | Het vs KO |
| - trial effect | repeated measures | sphericity violated |  | | | 16.439 | **0.0000** | 0.999 | - | - | - |  | repeated measures | sphericity violated |  | | | 8.134 | **0.0066** | 0.947 | - | - | - |
| - trial x genotype effect | repeated measures | sphericity violated |  |  |  | 0.192 | 0.8943 | 0.088 | - | - | - |  | repeated measures | sphericity violated |  |  |  | 0.630 | 0.5596 | 0.191 | - | - | - |
| - genotype effect | repeated measures | sphericity violated |  |  |  | 1.354 | 0.2751 | 0.266 | - | - | - |  | repeated measures | sphericity violated |  |  |  | 0.591 | 0.5620 | 0.136 | - | - | - |
|  |  |  |  |  |  |  |  |  |  |  |  |  |  |  |  |  |  |  |  |  |  |  |  |
| Individual trials | test | data structure | WT | Het | KO | genotype | | | pairwise comparisons | | |  | test | data structure | WT | Het | KO | genotype | | | pairwise comparisons | | |
|  |  |  |  |  |  | F | p-value | power | WT vs Het | WT vs KO | Het vs KO |  |  |  |  |  |  | F | p-value | power | WT vs Het | WT vs KO | Het vs KO |
| Water 1 | Kruskal-Wallis | non normal | 2.28 ± 0.57 | 1.66 ± 0.59 | 0.57 ± 0.14 | 10.844 | **0.0040** | NA | 0.331 | **0.001** | **0.025** |  | Kruskal-Wallis | non normal | 1.1 ± 0.2 | 2 ± 0.64 | 1.24 ± 0.27 | 0.534 | 0.7760 | NA | - | - | - |
| Water 2 | Kruskal-Wallis | non normal | 1.08 ± 0.23 | 0.96 ± 0.17 | 0.48 ± 0.15 | 5.231 | 0.0730 | NA | - | - | - |  | Kruskal-Wallis | non normal | 0.94 ± 0.17 | 1.01 ± 0.27 | 0.92 ± 0.24 | 0.456 | 0.7960 | NA | - | - | - |
| Water 3 | Kruskal-Wallis | non normal | 0.79 ± 0.15 | 0.71 ± 0.21 | 0.54 ± 0.16 | 1.446 | 0.4850 | NA | - | - | - |  | ANOVA | normal | 1.26 ± 0.28 | 0.95 ± 0.19 | 0.86 ± 0.16 | 1.330 | 0.2841 | 0.258 | - | - | - |
| Banana 1 | Kruskal-Wallis | non normal | 1.06 ± 0.13 | 0.87 ± 0.34 | 0.13 ± 0.08 | 11.660 | **0.0030** | NA | 0.195 | **0.001** | **0.036** |  | ANOVA | normal | 1.77 ± 0.14 | 1.25 ± 0.33 | 0.74 ± 0.25 | 4.194 | **0.0280** | 0.678 | 0.232 | **0.021** | 0.433 |
| Banana 2 | Kruskal-Wallis | non normal | 0.55 ± 0.15 | 0.48 ± 0.09 | 0.16 ± 0.12 | 6.230 | **0.0440** | NA | 0.868 | **0.033** | **0.025** |  | ANOVA | normal | 0.81 ± 0.2 | 0.86 ± 0.19 | 0.71 ± 0.19 | 0.220 | 0.8045 | 0.080 | - | - | - |
| Banana 3 | Kruskal-Wallis | non normal | 0.34 ± 0.09 | 0.46 ± 0.12 | 0.22 ± 0.09 | 1.961 | 0.3750 | NA | - | - | - |  | Kruskal-Wallis | non normal | 0.81 ± 0.16 | 1.06 ± 0.37 | 0.66 ± 0.22 | 1.311 | 0.5190 | NA | - | - | - |
| Lemon 1 | Kruskal-Wallis | non normal | 0.34 ± 0.09 | 0.51 ± 0.17 | 0.19 ± 0.13 | 3.418 | 0.1810 | NA | - | - | - |  | Kruskal-Wallis | non normal | 0.94 ± 0.44 | 1.1 ± 0.22 | 0.84 ± 0.28 | 0.774 | 0.6790 | NA | - | - | - |
| Lemon 2 | Kruskal-Wallis | non normal | 0.26 ± 0.09 | 0.32 ± 0.11 | 0.07 ± 0.03 | 3.333 | 0.1890 | NA | - | - | - |  | Kruskal-Wallis | non normal | 0.59 ± 0.23 | 0.76 ± 0.15 | 0.4 ± 0.11 | 1.849 | 0.3970 | NA | - | - | - |
| Lemon 3 | Kruskal-Wallis | non normal | 0.37 ± 0.1 | 0.25 ± 0.07 | 0.06 ± 0.04 | 6.596 | **0.0370** | NA | 0.642 | **0.014** | **0.050** |  | Kruskal-Wallis | non normal | 0.33 ± 0.04 | 0.77 ± 0.15 | 0.44 ± 0.14 | 5.139 | 0.0770 | NA | - | - | - |
| Male 1 | Kruskal-Wallis | non normal | 6.35 ± 1.27 | 4.17 ± 1.07 | 6.57 ± 1.78 | 1.380 | 0.5030 | NA | - | - | - |  | Kruskal-Wallis | non normal | 5.71 ± 2.2 | 5.27 ± 1.54 | 3.32 ± 1.03 | 1.668 | 0.4340 | NA | - | - | - |
| Male 2 | Kruskal-Wallis | non normal | 2.09 ± 0.4 | 3.47 ± 0.69 | 0.98 ± 0.43 | 7.919 | **0.0190** | NA | 0.226 | *0.091* | **0.005** |  | Kruskal-Wallis | non normal | 2.5 ± 0.63 | 1.89 ± 0.43 | 1.55 ± 0.47 | 2.376 | 0.3050 | NA | - | - | - |
| Male 3 | Kruskal-Wallis | non normal | 1.8 ± 0.56 | 1.37 ± 0.39 | 2.33 ± 1.25 | 1.039 | 0.5950 | NA | - | - | - |  | Kruskal-Wallis | non normal | 1.13 ± 0.42 | 1.09 ± 0.27 | 1.1 ± 0.23 | 0.133 | 0.9360 | NA | - | - | - |
| Female 1 | Kruskal-Wallis | non normal | 7.1 ± 1.15 | 7.95 ± 2.04 | 10.25 ± 3.38 | 0.277 | 0.8710 | NA | - | - | - |  | Kruskal-Wallis | non normal | 3.44 ± 1.47 | 4.52 ± 1.46 | 2.4 ± 0.79 | 1.557 | 0.4590 | NA | - | - | - |
| Female 2 | Kruskal-Wallis | non normal | 2.45 ± 0.43 | 2.33 ± 0.72 | 3.3 ± 1.56 | 0.365 | 0.8300 | NA | - | - | - |  | ANOVA | normal | 1.76 ± 0.32 | 2.13 ± 0.4 | 1.94 ± 0.5 | 0.034 | 0.9664 | 0.055 | - | - | - |
| Female 3 | Kruskal-Wallis | non normal | 1.73 ± 0.39 | 2.24 ± 0.92 | 4.47 ± 1.61 | 1.757 | 0.4150 | NA | - | - | - |  | ANOVA | normal | 0.93 ± 0.25 | 1.29 ± 0.27 | 1.05 ± 0.16 | 0.273 | 0.7637 | 0.088 | - | - | - |
|  |  |  |  |  |  |  |  |  |  |  |  |  |  |  |  |  |  |  |  |  |  |  |  |
| **Olfactory habituation/dishabituation, all interactions** |  |  |  |  |  |  |  |  |  |  |  |  |  |  |  |  |  |  |  |  |  |  |  |
|  | Cohort 1 | | | | | | | | | | |  | Cohort 2 | | | | | | | | | | |
| Water repeated measure | test | data structure |  | | | F | p-value | power | WT vs Het | WT vs KO | Het vs KO |  | test | data structure |  | | | F | p-value | power | WT vs Het | WT vs KO | Het vs KO |
| - trial effect | repeated measures | sphericity violated |  | | | 3.221 | *0.0655* | 0.591 | - | - | - |  | repeated measures | sphericity assumed |  | | | 0.064 | 0.9383 | 0.059 | - | - | - |
| - trial x genotype effect | repeated measures | sphericity violated |  |  |  | 0.922 | 0.4362 | 0.273 | - | - | - |  | repeated measures | sphericity assumed |  |  |  | 0.700 | 0.5958 | 0.198 | - | - | - |
| - genotype effect | repeated measures | sphericity violated |  |  |  | 5.222 | **0.0121** | 0.786 | 0.106 | **0.049** | 0.367 |  | repeated measures | sphericity assumed |  |  |  | 2.210 | 0.1315 | 0.406 | - | - | - |
|  |  |  |  |  |  |  |  |  |  |  |  |  |  |  |  |  |  |  |  |  |  |  |  |
| Banana repeated measure | test | data structure |  | | | F | p-value | power | WT vs Het | WT vs KO | Het vs KO |  | test | data structure |  | | | F | p-value | power | WT vs Het | WT vs KO | Het vs KO |
| - trial effect | repeated measures | sphericity assumed |  | | | 2.634 | *0.0813* | 0.457 | - | - | - |  | repeated measures | sphericity violated |  | | | 3.155 | *0.0753* | 0.578 | - | - | - |
| - trial x genotype effect | repeated measures | sphericity assumed |  |  |  | 4.116 | **0.0057** | 0.846 | - | - | - |  | repeated measures | sphericity violated |  |  |  | 0.591 | 0.6034 | 0.182 | - | - | - |
| - genotype effect | repeated measures | sphericity assumed |  |  |  | 2.914 | *0.0722* | 0.519 | 0.372 | 0.143 | **0.024** |  | repeated measures | sphericity violated |  |  |  | 3.384 | *0.0508* | 0.581 | 0.787 | *0.054* | **0.026** |
|  |  |  |  |  |  |  |  |  |  |  |  |  |  |  |  |  |  |  |  |  |  |  |  |
| Lemon repeated measure | test | data structure |  | | | F | p-value | power | WT vs Het | WT vs KO | Het vs KO |  | test | data structure |  | | | F | p-value | power | WT vs Het | WT vs KO | Het vs KO |
| - trial effect | repeated measures | sphericity violated |  | | | 3.678 | **0.0438** | 0.652 | - | - | - |  | repeated measures | sphericity violated |  | | | 0.500 | 0.5537 | 0.127 | - | - | - |
| - trial x genotype effect | repeated measures | sphericity violated |  |  |  | 2.138 | 0.1075 | 0.593 | - | - | - |  | repeated measures | sphericity violated |  |  |  | 0.471 | 0.7002 | 0.152 | - | - | - |
| - genotype effect | repeated measures | sphericity violated |  |  |  | 2.207 | 0.1302 | 0.409 | - | - | - |  | repeated measures | sphericity violated |  |  |  | 3.855 | **0.0353** | 0.642 | 0.192 | 0.202 | **0.010** |
|  |  |  |  |  |  |  |  |  |  |  |  |  |  |  |  |  |  |  |  |  |  |  |  |
| Male repeated measure | test | data structure |  | | | F | p-value | power | WT vs Het | WT vs KO | Het vs KO |  | test | data structure |  | | | F | p-value | power | WT vs Het | WT vs KO | Het vs KO |
| - trial effect | repeated measures | sphericity assumed |  | | | 21.516 | **0.0000** | 1.000 | - | - | - |  | repeated measures | sphericity violated |  | | | 9.190 | **0.0043** | 0.969 | - | - | - |
| - trial x genotype effect | repeated measures | sphericity assumed |  |  |  | 0.244 | 0.9119 | 0.096 | - | - | - |  | repeated measures | sphericity violated |  |  |  | 0.828 | 0.4592 | 0.245 | - | - | - |
| - genotype effect | repeated measures | sphericity assumed |  |  |  | 2.225 | 0.1275 | 0.414 | - | - | - |  | repeated measures | sphericity violated |  |  |  | 4.701 | **0.0189** | 0.733 | 0.751 | **0.012** | **0.020** |
|  |  |  |  |  |  |  |  |  |  |  |  |  |  |  |  |  |  |  |  |  |  |  |  |
| Female repeated measure | test | data structure |  | | | F | p-value | power | WT vs Het | WT vs KO | Het vs KO |  | test | data structure |  | | | F | p-value | power | WT vs Het | WT vs KO | Het vs KO |
| - trial effect | repeated measures | sphericity assumed |  | | | 21.617 | **0.0000** | 1.000 | - | - | - |  | repeated measures | sphericity violated |  | | | 6.457 | **0.0082** | 0.886 | - | - | - |
| - trial x genotype effect | repeated measures | sphericity assumed |  |  |  | 2.706 | **0.0397** | 0.709 | - | - | - |  | repeated measures | sphericity violated |  |  |  | 0.177 | 0.9074 | 0.079 | - | - | - |
| - genotype effect | repeated measures | sphericity assumed |  |  |  | 0.964 | 0.3942 | 0.200 | - | - | - |  | repeated measures | sphericity violated |  |  |  | 0.446 | 0.6452 | 0.114 | - | - | - |
|  |  |  |  |  |  |  |  |  |  |  |  |  |  |  |  |  |  |  |  |  |  |  |  |
| Individual trials | test | data structure | WT | Het | KO | genotype | | | pairwise comparisons | | |  | test | data structure | WT | Het | KO | genotype | | | pairwise comparisons | | |
|  |  |  |  |  |  | F | p-value | power | WT vs Het | WT vs KO | Het vs KO |  |  |  |  |  |  | F | p-value | power | WT vs Het | WT vs KO | Het vs KO |
| Water 1 | Kruskal-Wallis | non normal | 4.78 ± 1.46 | 4.99 ± 1.56 | 1.35 ± 0.47 | 5.315 | *0.0700* | NA | - | - | - |  | Kruskal-Wallis | non normal | 1.78 ± 0.29 | 2.48 ± 1.07 | 1.35 ± 0.33 | 1.853 | 0.3960 | NA | - | - | - |
| Water 2 | Kruskal-Wallis | non normal | 4.13 ± 1.07 | 2.23 ± 0.84 | 0.54 ± 0.23 | 12.735 | **0.0020** | NA | 0.197 | **0.001** | **0.025** |  | Kruskal-Wallis | non normal | 2.51 ± 0.81 | 1.22 ± 0.26 | 1.32 ± 0.37 | 1.057 | 0.5890 | NA | - | - | - |
| Water 3 | Kruskal-Wallis | non normal | 2.21 ± 0.56 | 2.37 ± 0.85 | 1.01 ± 0.36 | 3.745 | 0.1540 | NA | - | - | - |  | Kruskal-Wallis | non normal | 2.97 ± 1.06 | 2.21 ± 0.91 | 0.9 ± 0.23 | 2.274 | 0.3210 | NA | - | - | - |
| Banana 1 | Kruskal-Wallis | non normal | 2 ± 0.18 | 1.64 ± 0.6 | 0.3 ± 0.2 | 11.729 | **0.0030** | NA | 0.200 | **0.001** | **0.034** |  | Kruskal-Wallis | non normal | 3.91 ± 0.85 | 5.55 ± 2.35 | 1.1 ± 0.8 | 10.192 | **0.0060** | NA | 0.506 | **0.003** | **0.017** |
| Banana 2 | Kruskal-Wallis | non normal | 0.78 ± 0.24 | 0.68 ± 0.2 | 0.52 ± 0.27 | 2.478 | 0.2900 | NA | - | - | - |  | Kruskal-Wallis | non normal | 2.3 ± 1.02 | 1.54 ± 0.75 | 0.62 ± 0.24 | 3.575 | 0.1670 | NA | - | - | - |
| Banana 3 | Kruskal-Wallis | non normal | 0.45 ± 0.14 | 2.26 ± 1.01 | 0.24 ± 0.15 | 7.021 | **0.0300** | NA | 0.187 | 0.154 | **0.008** |  | Kruskal-Wallis | non normal | 2.66 ± 1.2 | 1.67 ± 0.73 | 0.55 ± 0.12 | 6.081 | **0.0480** | NA | 0.924 | **0.044** | **0.029** |
| Lemon 1 | Kruskal-Wallis | non normal | 0.96 ± 0.26 | 1.52 ± 0.49 | 0.51 ± 0.34 | 4.258 | 0.1190 | NA | - | - | - |  | Kruskal-Wallis | non normal | 2.18 ± 0.78 | 3.07 ± 1.55 | 0.39 ± 0.22 | 11.769 | **0.0030** | NA | 0.759 | **0.007** | **0.002** |
| Lemon 2 | Kruskal-Wallis | non normal | 1.18 ± 0.5 | 0.56 ± 0.34 | 0.14 ± 0.09 | 4.733 | *0.0940* | NA | - | - | - |  | Kruskal-Wallis | non normal | 1.26 ± 0.5 | 1.51 ± 0.67 | 0.4 ± 0.15 | 3.844 | 0.1460 | NA | - | - | - |
| Lemon 3 | Kruskal-Wallis | non normal | 0.7 ± 0.22 | 0.38 ± 0.18 | 0.22 ± 0.16 | 4.858 | *0.0880* | NA | - | - | - |  | Kruskal-Wallis | non normal | 0.94 ± 0.31 | 0.88 ± 0.22 | 0.2 ± 0.05 | 11.373 | **0.0030** | NA | 0.322 | **0.030** | **0.001** |
| Male 1 | ANOVA | normal | 53.4 ± 7.5 | 48.9 ± 9.52 | 32.8 ± 10 | 1.411 | 0.2613 | 0.266 | - | - | - |  | Kruskal-Wallis | non normal | 59.48 ± 14.13 | 49.13 ± 12.04 | 20.81 ± 6.48 | 2.325 | 0.3130 | NA | - | - | - |
| Male 2 | Kruskal-Wallis | non normal | 29.44 ± 8.93 | 31.2 ± 7.74 | 10.69 ± 8.64 | 8.049 | **0.0180** | NA | 0.911 | **0.014** | **0.012** |  | Kruskal-Wallis | non normal | 23.86 ± 7.84 | 26.67 ± 7.58 | 4.28 ± 1.69 | 10.016 | **0.0070** | NA | 0.934 | **0.007** | **0.007** |
| Male 3 | Kruskal-Wallis | non normal | 23.09 ± 8.92 | 15.5 ± 6.75 | 5.04 ± 2.65 | 6.041 | **0.0490** | NA | 0.746 | **0.021** | *0.051* |  | Kruskal-Wallis | non normal | 9.97 ± 3.82 | 13 ± 5.81 | 5.48 ± 2.08 | 2.294 | 0.3180 | NA | - | - | - |
| Female 1 | ANOVA | normal | 58.56 ± 7.1 | 56.89 ± 8.12 | 29.62 ± 8.93 | 3.919 | **0.0320** | 0.611 | 0.987 | **0.043** | *0.066* |  | Kruskal-Wallis | non normal | 44.66 ± 10.62 | 40.1 ± 2.73 | 32.69 ± 11.87 | 0.349 | 0.8400 | NA | - | - | - |
| Female 2 | Kruskal-Wallis | non normal | 27.99 ± 8.59 | 26.36 ± 11.34 | 15.87 ± 7.3 | 2.302 | 0.3160 | NA | - | - | - |  | Kruskal-Wallis | non normal | 15.83 ± 9.33 | 9.47 ± 4.66 | 13.47 ± 5.23 | 0.976 | 0.9760 | NA | - | - | - |
| Female 3 | Kruskal-Wallis | non normal | 12.36 ± 4.44 | 11.78 ± 5.04 | 21.44 ± 7.48 | 0.448 | 0.7990 | NA | - | - | - |  | Kruskal-Wallis | non normal | 17.61 ± 7.04 | 11.15 ± 5.81 | 6.99 ± 4.61 | 6.743 | **0.0340** | NA | 0.172 | **0.009** | 0.216 |
