# Supplementary material for: Behavioral Phenotyping of an Improved Mouse Model of Phelan–McDermid Syndrome with a Complete Deletion of the Shank3 Gene
Source: eNeuro. 2018 Oct 5;5(3):ENEURO.0046-18.2018. doi: 10.1523/ENEURO.0046-18.2018 (PMC6175061; doi:10.1523/ENEURO.0046-18.2018)
Supplement: Extended Data Table 8-1 — Individual results and statistical analyses for cohorts 1 and 2 related to social behavior. WT, wild-type mice; Het, heterozygous mice; KO, homozygous knockout mice. C, center chamber; M, mouse chamber; O, object chamber. Group values are reported as means ± s.e.m. Red font indicates significant results (p < 0.05), orange font indicates trends (0.1 < p < 0.05). Download Table 8-1, DOCX file. [file sup_enu-eN-CFN-0046-18-s05.docx]

# Extended Tables

Extended table 8-1

| **3 chambered social interaction test - social preference** |  |  |  |  |  |  |  |  |  |  |  |  |  |  |  |  |  |  |  |  |  |  |  |
| --- | --- | --- | --- | --- | --- | --- | --- | --- | --- | --- | --- | --- | --- | --- | --- | --- | --- | --- | --- | --- | --- | --- | --- |
|  | Cohort 1 | | | | | | | | | | |  | Cohort 2 | | | | | | | | | | |
| Zone comparison, 3 zones, repeated measures | test | data structure |  | | | F | p-value | power | C vs M | C vs O | M vs O |  | test | data structure |  | | | F | p-value | power | C vs M | C vs O | M vs O |
| WT, time in chambers | repeated measures | sphericity assumed |  | | | 128.691 | **0.000** | 1.000 | **0.000** | **0.000** | **0.000** |  | repeated measures | sphericity assumed |  | | | 50.234 | **0.000** | 1.000 | **0.000** | **0.000** | **0.000** |
| WT, time in chambers | repeated measures | sphericity assumed |  | | | 82.545 | **0.000** | 1.000 | **0.000** | **0.007** | **0.000** |  | repeated measures | sphericity assumed |  | | | 18.929 | **0.000** | 0.997 | **0.001** | **0.035** | **0.001** |
| Het, time in chambers | repeated measures | sphericity assumed |  | | | 47.573 | **0.000** | 1.000 | **0.000** | 0.109 | **0.000** |  | repeated measures | sphericity assumed |  | | | 25.537 | **0.000** | 0.999 | **0.000** | **0.006** | **0.005** |
| KO, time in chambers | repeated measures | sphericity assumed |  | | | 21.063 | **0.001** | 1.000 | **0.000** | *0.063* | **0.000** |  | repeated measures | sphericity assumed |  | | | 11.839 | **0.001** | 0.981 | **0.001** | **0.015** | *0.066* |
|  |  |  |  |  |  |  |  |  |  |  |  |  |  |  |  |  |  |  |  |  |  |  |  |
| Zone comparison, 2 zones, repeated measures | Cohort 1 | | | | | | | |  |  |  |  | Cohort 2 | | | | | | | |  |  |  |
| Mouse A - Object, interaction time | test | data structure |  | | | F | p-value | power |  |  |  |  | test | data structure |  | | | All F | All p-value | power |  |  |  |
| - chamber effect, all mice | repeated measures | sphericity assumed |  |  |  | 18.844 | **0.000** | 0.987 |  |  |  |  | repeated measures | sphericity assumed |  |  |  | 20.988 | **0.000** | 0.993 |  |  |  |
| - chamber effect, WT | repeated measures | sphericity assumed |  |  |  | 6.942 | **0.027** | 0.651 |  |  |  |  | repeated measures | sphericity assumed |  |  |  | 4.063 | *0.084* | 0.413 |  |  |  |
| - chamber effect, Het | repeated measures | sphericity assumed |  |  |  | 3.768 | *0.084* | 0.411 |  |  |  |  | repeated measures | sphericity assumed |  |  |  | 9.615 | **0.015** | 0.775 |  |  |  |
| - chamber effect, KO | repeated measures | sphericity assumed |  |  |  | 7.681 | **0.024** | 0.681 |  |  |  |  | repeated measures | sphericity assumed |  |  |  | 15.938 | **0.003** | 0.943 |  |  |  |
|  |  |  |  |  |  |  |  |  |  |  |  |  |  |  |  |  |  |  |  |  |  |  |  |
| Group comparison | test | data structure | WT | Het | KO | genotype | | | pairwise comparisons | | |  | test | data structure | WT | Het | KO | genotype | | | pairwise comparisons | | |
|  |  |  |  |  |  | F | p-value | power | WT vs Het | WT vs KO | Het vs KO |  |  |  |  |  |  | F | p-value | power | WT vs Het | WT vs KO | Het vs KO |
| Mouse-Object, total time in mouse or object chamber | Kruskal-Wallis | non normal | 545.82 ± 8.08 | 531.81 ± 4.67 | 511.87 ± 12.25 | 7.793 | **0.020** | NA | **0.029** | **0.010** | 0.643 |  | Kruskal-Wallis | non normal | 506.67 ± 20.82 | 484.63 ± 58.17 | 491.57 ± 25.14 | 3.804 | 0.149 | NA | - | - | - |
| Mouse-Object, total time sniffing mouse or object | ANOVA | normal | 96.33 ± 8.85 | 121.31 ± 15.09 | 99.68 ± 21 | 0.798 | 0.461 | 0.171 | - | - | - |  | ANOVA | normal | 79.88 ± 9.6 | 83.56 ± 11.78 | 102.71 ± 11.03 | 1.286 | 0.295 | 0.252 | - | - | - |
| Mouse-Object, total time close to mouse or object | Kruskal-Wallis | non normal | 167.88 ± 5.67 | 171.93 ± 8.04 | 153.87 ± 18.6 | 0.648 | 0.531 | NA | - | - | - |  | ANOVA | normal | 154.89 ± 18.17 | 165.18 ± 22.2 | 139.85 ± 11.31 | 0.566 | 0.575 | 0.133 | - | - | - |
|  |  |  |  |  |  |  |  |  |  |  |  |  |  |  |  |  |  |  |  |  |  |  |  |
| **Male-female social interactions, sniffing** |  |  |  |  |  |  |  |  |  |  |  |  |  |  |  |  |  |  |  |  |  |  |  |
|  | Cohort 1 | | | | | | | | | | |  | Cohort 2 | | | | | | | | | | |
|  | test | data structure | WT | Het | KO | genotype | | | pairwise comparisons | | |  | test | data structure | WT | Het | KO | genotype | | | pairwise comparisons | | |
|  |  |  |  |  |  | F | p-value | power | WT vs Het | WT vs KO | Het vs KO |  |  |  |  |  |  | F | p-value | power | WT vs Het | WT vs KO | Het vs KO |
| Anogenital sniffing, total time (seconds) | Kruskal-Wallis | non normal | 11.27 ± 2.21 | 13 ± 2.85 | 7.62 ± 2.06 | 1.610 | 0.447 | NA | - | - | - |  | Kruskal-Wallis | non normal | 8.57 ± 2.35 | 11.66 ± 2.49 | 10.33 ± 2.01 | 1.656 | 0.437 | NA | - | - | - |
| Anogenital sniffing, number of interactions | Kruskal-Wallis | non normal | 12.64 ± 4.18 | 12.37 ± 3.05 | 7.24 ± 3.66 | 2.054 | 0.358 | NA | - | - | - |  | Kruskal-Wallis | non normal | 12.45 ± 6.37 | 16.4 ± 8.05 | 8.23 ± 3.26 | 0.653 | 0.721 | NA | - | - | - |
| Anogenital sniffing, latency to first exploration (seconds) | Kruskal-Wallis | non normal | 14.09 ± 5.14 | 23.91 ± 15.8 | 52.06 ± 27.39 | 3.632 | 0.163 | NA | - | - | - |  | Kruskal-Wallis | non normal | 47.05 ± 27.83 | 14.83 ± 6.02 | 110.83 ± 39.81 | 2.033 | *0.362* | NA | - | - | - |
| Nose to body sniffing, total time (seconds) | Kruskal-Wallis | non normal | 16.63 ± 2.89 | 18 ± 4.91 | 13 ± 2.47 | 1.075 | 0.584 | NA | - | - | - |  | Kruskal-Wallis | non normal | 11.83 ± 2.08 | 11.55 ± 1.39 | 19.77 ± 4.85 | 0.493 | 0.781 | NA | - | - | - |
| Nose to body sniffing, number of interactions | Kruskal-Wallis | non normal | 11.73 ± 2.43 | 11.16 ± 2.97 | 8.01 ± 2.22 | 0.327 | 0.849 | NA | - | - | - |  | Kruskal-Wallis | non normal | 9.67 ± 1.22 | 10.46 ± 1.55 | 19.15 ± 7.78 | 2.710 | 0.258 | NA | - | - | - |
| Nose to body sniffing, latency to first exploration (seconds) | Kruskal-Wallis | non normal | 20.91 ± 8 | 30.6 ± 15.07 | 11.63 ± 4.22 | 0.135 | 0.935 | NA | - | - | - |  | Kruskal-Wallis | non normal | 15.72 ± 6.37 | 10.21 ± 3.1 | 14.93 ± 6.73 | 0.232 | 0.890 | NA | - | - | - |
| Nose to nose sniffing, total time (seconds) | Kruskal-Wallis | non normal | 8.81 ± 1.19 | 11.3 ± 1.69 | 8.87 ± 1.43 | 2.326 | 0.313 | NA | - | - | - |  | Kruskal-Wallis | non normal | 8.14 ± 1.24 | 10.11 ± 1.35 | 10.22 ± 1.78 | 0.939 | 0.626 | NA | - | - | - |
| Nose to nose sniffing, number of interactions | Kruskal-Wallis | non normal | 6.45 ± 0.97 | 6.94 ± 0.97 | 4.99 ± 0.81 | 1.175 | 0.556 | NA | - | - | - |  | Kruskal-Wallis | non normal | 6.08 ± 1.42 | 6.87 ± 0.84 | 8.05 ± 2.06 | 0.577 | 0.577 | NA | - | - | - |
| Nose to nose sniffing, latency to first exploration (seconds) | Kruskal-Wallis | non normal | 38.62 ± 10.97 | 29.86 ± 11.36 | 19.56 ± 5.79 | 1.369 | 0.504 | NA | - | - | - |  | Kruskal-Wallis | non normal | 28.59 ± 11.11 | 38.78 ± 15.47 | 13.93 ± 5.62 | 1.284 | 0.526 | NA | - | - | - |
| All sniffing, total time (seconds) | Kruskal-Wallis | non normal | 36.72 ± 5.52 | 42.3 ± 8.64 | 29.5 ± 4.67 | 2.761 | 0.251 | NA | - | - | - |  | Kruskal-Wallis | non normal | 29.14 ± 3.72 | 33.33 ± 3.1 | 40.33 ± 7.57 | 0.839 | 0.657 | NA | - | - | - |
| All sniffing, number of interactions | Kruskal-Wallis | non normal | 30.83 ± 6.27 | 30.48 ± 6.17 | 20.25 ± 5.61 | 0.536 | 0.536 | NA | - | - | - |  | Kruskal-Wallis | non normal | 28.21 ± 7.07 | 33.74 ± 8.77 | 35.44 ± 10.91 | 1.216 | 0.544 | NA | - | - | - |
| All sniffing, latency to first exploration (seconds) | Kruskal-Wallis | non normal | 38.62 ± 10.97 | 29.86 ± 11.36 | 19.56 ± 5.79 | 0.588 | 0.588 | NA | - | - | - |  | Kruskal-Wallis | non normal | 38.62 ± 10.97 | 29.86 ± 11.36 | 19.56 ± 5.79 | 0.059 | 0.971 | NA | - | - | - |
|  |  |  |  |  |  |  |  |  |  |  |  |  |  |  |  |  |  |  |  |  |  |  |  |
| **Male-female social interactions,ultrasonic vocalization** |  |  |  |  |  |  |  |  |  |  |  |  |  |  |  |  |  |  |  |  |  |  |  |
|  | Cohort 1 | | | | | | | | | | |  | Cohort 2 | | | | | | | | | | |
|  | test | data structure | WT | Het | KO | genotype | | | pairwise comparisons | | |  | test | data structure | WT | Het | KO | genotype | | | pairwise comparisons | | |
|  |  |  |  |  |  | F | p-value | power | WT vs Het | WT vs KO | Het vs KO |  |  |  |  |  |  | F | p-value | power | WT vs Het | WT vs KO | Het vs KO |
| USV, all calls | Kruskal-Wallis | non normal | 321.63 ± 48.18 | 348.11 ± 65.79 | 228.75 ± 22.95 | 2.931 | 0.231 | NA | - | - | - |  | Kruskal-Wallis | non normal | 472 ± 100.48 | 408.55 ± 115.23 | 339.88 ± 51.98 | 1.332 | 0.514 | NA | - | - | - |
| USV, minute 1 | Kruskal-Wallis | non normal | 65.27 ± 10.76 | 98.55 ± 30.21 | 54.75 ± 6.34 | 1.423 | 0.491 | NA | - | - | - |  | Kruskal-Wallis | non normal | 113.85 ± 23.83 | 89.66 ± 32.85 | 73.55 ± 14.24 | 2.991 | 0.224 | NA | - | - | - |
| USV, minute 2 | Kruskal-Wallis | non normal | 60.9 ± 11.54 | 69.55 ± 15.26 | 50.12 ± 6 | 0.594 | 0.743 | NA | - | - | - |  | Kruskal-Wallis | non normal | 79.42 ± 14.44 | 76.88 ± 24.97 | 63.88 ± 8.79 | 1.181 | 0.554 | NA | - | - | - |
| USV, minute 3 | Kruskal-Wallis | non normal | 70.9 ± 10.17 | 69.33 ± 10.29 | 40.12 ± 3.45 | 7.696 | **0.021** | NA | 0.999 | **0.012** | **0.017** |  | Kruskal-Wallis | non normal | 88.14 ± 26.13 | 68.11 ± 14.42 | 73.33 ± 13.43 | 0.949 | 0.622 | NA | - | - | - |
| USV, minute 4 | Kruskal-Wallis | non normal | 58.9 ± 8.93 | 62.88 ± 9.55 | 44.62 ± 5.16 | 2.717 | 0.257 | NA | - | - | - |  | Kruskal-Wallis | non normal | 99 ± 34.01 | 90 ± 24.11 | 59.88 ± 7.7 | 0.780 | 0.800 | NA | - | - | - |
| USV, minute 5 | Kruskal-Wallis | non normal | 65.63 ± 17.35 | 47.77 ± 7.28 | 39.12 ± 5.67 | 1.570 | 0.456 | NA | - | - | - |  | Kruskal-Wallis | non normal | 91.57 ± 24.8 | 83.88 ± 21.23 | 69.22 ± 13.1 | 0.780 | 0.742 | NA | - | - | - |
|  |  |  |  |  |  |  |  |  |  |  |  |  |  |  |  |  |  |  |  |  |  |  |  |
| USV, repeated measure | test | data structure |  | | | F | p-value | power | WT vs Het | WT vs KO | Het vs KO |  | test | data structure |  | | | F | p-value | power | WT vs Het | WT vs KO | Het vs KO |
| - time effect | repeated measures | sphericity assumed |  | | | 2.645 | **0.038** | 0.720 | - | - | - |  | repeated measures | sphericity assumed |  | | | 1.382 | 0.262 | 0.414 | - | - | - |
| - time x genotype effect | repeated measures | sphericity assumed |  |  |  | 1.393 | 0.209 | 0.605 | - | - | - |  | repeated measures | sphericity assumed |  |  |  | 0.594 | 0.669 | 0.259 | - | - | - |
| - genotype effect | repeated measures | sphericity assumed |  |  |  | 1.388 | 0.268 | 0.270 | - | - | - |  | repeated measures | sphericity assumed |  |  |  | 0.485 | 0.622 | 0.119 | - | - | - |
|  |  |  |  |  |  |  |  |  |  |  |  |  |  |  |  |  |  |  |  |  |  |  |  |
|  |  |  |  |  |  |  |  |  |  |  |  |  |  |  |  |  |  |  |  |  |  |  |  |
|  |  |  |  |  |  |  |  |  |  |  |  |  |  |  |  |  |  |  |  |  |  |  |  |
|  |  |  |  |  |  |  |  |  |  |  |  |  |  |  |  |  |  |  |  |  |  |  |  |
| **Social transmission of food preference** |  |  |  |  |  |  |  |  |  |  |  |  |  |  |  |  |  |  |  |  |  |  |  |
|  | Cohort 1 | | | | | | | | | | |  | Cohort 2 | | | | | | | | | | |
|  | test | data structure | WT | Het | KO | genotype | | | pairwise comparisons | | |  | test | data structure | WT | Het | KO | genotype | | | pairwise comparisons | | |
|  |  |  |  |  |  | F | p-value | power | WT vs Het | WT vs KO | Het vs KO |  |  |  |  |  |  | F | p-value | power | WT vs Het | WT vs KO | Het vs KO |
| Time spent sniffing the demonstrator | Kruskal-Wallis | non normal | 39.72 ± 8.84 | 45.58 ± 10.02 | 28.97 ± 11.44 | 2.458 | 0.293 | NA | - | - | - |  | Kruskal-Wallis | non normal | 16.17 ± 4.6 | 28.39 ± 6.4 | 20.85 ± 6.85 | 1.666 | 0.435 | 0.225 | - | - | - |
| Number of sniffing bouts | ANOVA | normal | 11.81 ± 2.34 | 15 ± 2.27 | 8.44 ± 2.51 | 1.096 | 0.350 | 0.220 | - | - | - |  | ANOVA | normal | 6.75 ± 1.06 | 12 ± 1.85 | 6.2 ± 1.34 | 4.404 | **0.024** | 0.701 | *0.058* | 0.963 | **0.024** |
|  |  |  |  |  |  |  |  |  |  |  |  |  |  |  |  |  |  |  |  |  |  |  |  |
| Time spent exploring all food (sec) | ANOVA | normal | 1608.9 ± 148.84 | 1414.5 ± 124.79 | 1753.55 ± 199.94 | 0.909 | 0.416 | 0.188 | - | - | - |  | ANOVA | normal | 1429.5 ± 114.22 | 1503.55 ± 160.51 | 1680.8 ± 164.6 | 0.607 | 0.554 | 0.139 | - | - | - |
| Time pre-exposed/all food (%) | ANOVA | normal | 71.69 ± 4.18 | 57.16 ± 8.36 | 59.26 ± 8.04 | 1.295 | 0.292 | 0.253 | - | - | - |  | ANOVA | normal | 55.55 ± 4.6 | 61.5 ± 4.54 | 69.29 ± 5.22 | 1.341 | 0.281 | 0.260 | - | - | - |
| Time New/all food (%) | ANOVA | normal | 28.3 ± 4.18 | 42.83 ± 8.36 | 40.73 ± 8.04 | 1.295 | 0.292 | 0.253 | - | - | - |  | ANOVA | normal | 44.44 ± 4.6 | 38.49 ± 4.54 | 30.7 ± 5.22 | 1.341 | 0.281 | 0.260 | - | - | - |
| Ratio time pre-exposed/new | Kruskal-Wallis | non normal | 3.47 ± 0.67 | 2.56 ± 0.8 | 2.93 ± 1.2 | 1.870 | 0.393 | NA | - | - | - |  | Kruskal-Wallis | non normal | 1.41 ± 0.23 | 2.02 ± 0.48 | 3.18 ± 0.65 | 3.586 | 0.166 | NA | - | - | - |
| Time spent exploring cocoa / all food (%) | ANOVA | normal | 46.64 ± 7.96 | 55.44 ± 8.51 | 50.18 ± 8.69 | 0.343 | 0.713 | 0.099 | - | - | - |  | ANOVA | normal | 57.49 ± 4.19 | 45.32 ± 5.87 | 51.67 ± 8.26 | 0.587 | 0.564 | 0.136 | - | - | - |
| Time spent exploring cinnamon / all food (%) | ANOVA | normal | 53.35 ± 7.96 | 44.55 ± 8.51 | 49.81 ± 8.69 | 0.343 | 0.713 | 0.099 | - | - | - |  | ANOVA | normal | 42.5 ± 4.19 | 54.67 ± 5.87 | 48.32 ± 8.26 | 0.587 | 0.564 | 0.136 | - | - | - |
| Ratio time cocoa/cinnamon | Kruskal-Wallis | non normal | 1.59 ± 0.52 | 2.27 ± 0.71 | 2.38 ± 1.23 | 0.500 | 0.779 | NA | - | - | - |  | Kruskal-Wallis | non normal | 1.5 ± 0.22 | 1 ± 0.21 | 1.8 ± 0.56 | 1.859 | 0.395 | NA | - | - | - |
|  |  |  |  |  |  |  |  |  |  |  |  |  |  |  |  |  |  |  |  |  |  |  |  |
| Total amount of eaten food (g) | ANOVA | normal | 1.32 ± 0.2 | 1.05 ± 0.28 | 0.57 ± 0.09 | 2.924 | *0.073* | 0.517 | 0.657 | *0.061* | 0.302 |  | Kruskal-Wallis | non normal | 1.07 ± 0.37 | 0.58 ± 0.1 | 0.6 ± 0.09 | 1.319 | 0.517 | NA | - | - | - |
| Amount of eaten food, pre-exposed (g) | ANOVA | normal | 1.04 ± 0.16 | 0.85 ± 0.25 | 0.43 ± 0.09 | 2.591 | *0.096* | 0.467 | 0.760 | *0.083* | 0.299 |  | Kruskal-Wallis | non normal | 0.64 ± 0.24 | 0.48 ± 0.11 | 0.49 ± 0.11 | 0.021 | 0.989 | NA | - | - | - |
| Amount of eaten food, new (g) | Kruskal-Wallis | non normal | 0.28 ± 0.08 | 0.2 ± 0.08 | 0.13 ± 0.05 | 1.469 | 0,480 | NA | - | - | - |  | Kruskal-Wallis | non normal | 0.42 ± 0.17 | 0.1 ± 0.03 | 0.14 ± 0.04 | 2.895 | **0.235** | NA | **0.043** | *0.074* | 0.937 |
| Amount of eaten food, cocoa (g) | Kruskal-Wallis | non normal | 0.61 ± 0.21 | 0.5 ± 0.19 | 0.25 ± 0.11 | 1.352 | 0.509 | NA | - | - | - |  | Kruskal-Wallis | non normal | 0.57 ± 0.14 | 0.22 ± 0.1 | 0.34 ± 0.11 | 4.840 | 0.089 | NA | - | - | - |
| Amount of eaten food, cinnamon (g) | Kruskal-Wallis | non normal | 0.71 ± 0.14 | 0.55 ± 0.25 | 0.32 ± 0.07 | 3.733 | 0.155 | NA | - | - | - |  | Kruskal-Wallis | non normal | 0.5 ± 0.27 | 0.36 ± 0.1 | 0.29 ± 0.08 | 0.362 | 0.834 | NA | - | - | - |
|  |  |  |  |  |  |  |  |  |  |  |  |  |  |  |  |  |  |  |  |  |  |  |  |
|  | Cohort 1 | | | | | | | |  |  |  |  | Cohort 2 | | | | | | | |  |  |  |
| Pre-exposure preference, interaction time | test | data structure |  | | | All F | All p-value | power |  |  |  |  | test | data structure |  | | | All F | All p-value | power |  |  |  |
| - All mice, percentage pre-exposed vs new | repeated measures | sphericity assumed |  | | | 10.558 | **0.003** | 0.881 |  |  |  |  | repeated measures | sphericity assumed |  | | | 18.700 | **0.000** | 0.986 |  |  |  |
| - WT, percentage pre-exposed vs new | repeated measures | sphericity assumed |  |  |  | 26.897 | **0.000** | 0.996 |  |  |  |  | repeated measures | sphericity assumed |  |  |  | 1.456 | 0.267 | 0.182 |  |  |  |
| - Het, percentage pre-exposed vs new | repeated measures | sphericity assumed |  |  |  | 0.733 | 0.414 | 0.120 |  |  |  |  | repeated measures | sphericity assumed |  |  |  | 6.398 | **0.035** | 0.603 |  |  |  |
| - WTe, percentage pre-exposed vs new | repeated measures | sphericity assumed |  |  |  | 1.326 | 0.283 | 0.174 |  |  |  |  | repeated measures | sphericity assumed |  |  |  | 13.658 | **0.005** | 0.907 |  |  |  |
|  |  |  |  |  |  |  |  |  |  |  |  |  |  |  |  |  |  |  |  |  |  |  |  |
| Flavor preference, interaction time | test | data structure |  | | | All F | All p-value | power |  |  |  |  | test | data structure |  | | | All F | All p-value | power |  |  |  |
| - All mice, time cacao vs cinnamon | repeated measures | sphericity assumed |  | | | 0.000 | 0.997 | 0.050 |  |  |  |  | repeated measures | sphericity assumed |  | | | 0.003 | 0.955 | 0.050 |  |  |  |
| - WT, time cacao vs cinnamon | repeated measures | sphericity assumed |  |  |  | 0.434 | 0.525 | 0.092 |  |  |  |  | repeated measures | sphericity assumed |  |  |  | 2.519 | 0.157 | 0.279 |  |  |  |
| - Het, time cacao vs cinnamon | repeated measures | sphericity assumed |  |  |  | 0.242 | 0.634 | 0.073 |  |  |  |  | repeated measures | sphericity assumed |  |  |  | 1.093 | 0.326 | 0.152 |  |  |  |
| - KO, time cacao vs cinnamon | repeated measures | sphericity assumed |  |  |  | 0.039 | 0.849 | 0.053 |  |  |  |  | repeated measures | sphericity assumed |  |  |  | 0.019 | 0.894 | 0.052 |  |  |  |
|  |  |  |  |  |  |  |  |  |  |  |  |  |  |  |  |  |  |  |  |  |  |  |  |
| Pre-exposure preference, amount of eaten food (g) | test | data structure |  | | | All F | All p-value | power |  |  |  |  | test | data structure |  | | | All F | All p-value | power |  |  |  |
| - All mice, pre-expose vs new | repeated measures | sphericity assumed |  | | | 27.643 | **0.000** | 0.999 |  |  |  |  | repeated measures | sphericity assumed |  | | | 14.936 | **0.001** | 0.960 |  |  |  |
| - WT pre-expose vs new | repeated measures | sphericity assumed |  |  |  | 22.368 | **0.001** | 0.987 |  |  |  |  | repeated measures | sphericity assumed |  |  |  | 1.010 | 0.354 | 0.137 |  |  |  |
| - Het, pre-expose vs new | repeated measures | sphericity assumed |  |  |  | 6.525 | **0.034** | 0.611 |  |  |  |  | repeated measures | sphericity assumed |  |  |  | 9.646 | **0.015** | 0.776 |  |  |  |
| - KO, pre-expose vs new | repeated measures | sphericity assumed |  |  |  | 6.811 | **0.035** | 0.612 |  |  |  |  | repeated measures | sphericity assumed |  |  |  | 7.424 | **0.023** | 0.680 |  |  |  |
|  |  |  |  |  |  |  |  |  |  |  |  |  |  |  |  |  |  |  |  |  |  |  |  |
| Flavor preference, amount of eaten food (g) | test | data structure |  | | | All F | All p-value | power |  |  |  |  | test | data structure |  | | | All F | All p-value | power |  |  |  |
| - All mice, cacao vs cinnamon | repeated measures | sphericity assumed |  | | | 0.236 | 0.631 | 0.075 |  |  |  |  | repeated measures | sphericity assumed |  | | | 0.012 | 0.915 | 0.051 |  |  |  |
| - WT, cacao vs cinnamon | repeated measures | sphericity assumed |  |  |  | 0.113 | 0.745 | 0.060 |  |  |  |  | repeated measures | sphericity assumed |  |  |  | 0.098 | 0.765 | 0.058 |  |  |  |
| - Het, cacao vs cinnamon | repeated measures | sphericity assumed |  |  |  | 0.026 | 0.876 | 0.052 |  |  |  |  | repeated measures | sphericity assumed |  |  |  | 0.653 | 0.443 | 0.110 |  |  |  |
| - KO, cacao vs cinnamon | repeated measures | sphericity assumed |  |  |  | 0.223 | 0.651 | 0.070 |  |  |  |  | repeated measures | sphericity assumed |  |  |  | 0.084 | 0.779 | 0.058 |  |  |  |
