# Supplementary material for: Behavioral Phenotyping of an Improved Mouse Model of Phelan–McDermid Syndrome with a Complete Deletion of the Shank3 Gene
Source: eNeuro. 2018 Oct 5;5(3):ENEURO.0046-18.2018. doi: 10.1523/ENEURO.0046-18.2018 (PMC6175061; doi:10.1523/ENEURO.0046-18.2018)
Supplement: Extended Data Table 9-1 — Individual results and statistical analyses for cohorts 1 and 2 related to the avoidance behavior. WT, wild-type mice; Het, heterozygous mice; KO, homozygous knockout mice. Group values are reported as means ± s.e.m. Red font indicates significant results (p < 0.05), orange font indicates trends (0.1 < p < 0.05). Download Table 9-1, DOCX file. [file sup_enu-eN-CFN-0046-18-s06.docx]

# Extended Tables

Extended Table 9-1

| **Novel object habituation** |  |  |  |  |  |  |  |  |  |  |  |  |  |  |  |  |  |  |  |  |  |  |  |
| --- | --- | --- | --- | --- | --- | --- | --- | --- | --- | --- | --- | --- | --- | --- | --- | --- | --- | --- | --- | --- | --- | --- | --- |
|  | Cohort 1 | | | | | | | | | | |  | Cohort 2 | | | | | | | | | | |
|  | test | data structure | WT | Het | KO | genotype | | | pairwise comparisons | | |  | test | data structure | WT | Het | KO | genotype | | | pairwise comparisons | | |
|  |  |  |  |  |  | F | p-value | power | WT vs Het | WT vs KO | Het vs KO |  |  |  |  |  |  | F | p-value | power | WT vs Het | WT vs KO | Het vs KO |
| Habituation, total distance (cm) | ANOVA | normal | 4209.36 ± 500.47 | 3389.04 ± 365.92 | 3897.18 ± 453.39 | 0.884 | 0.425 | 0.186 | - | - | - |  | ANOVA | normal | 2802.11 ± 313.6 | 2803.81 ± 206.78 | 2417.96 ± 112.19 | 1.153 | 0.333 | 0.229 | - | - | - |
| Habituation, time in left side (sec) | ANOVA | normal | 320.3 ± 27 | 323.19 ± 23.96 | 239.9 ± 31.42 | 2.804 | *0.078* | 0.505 | 0.997 | 0.117 | 0.111 |  | ANOVA | normal | 307.09 ± 32.17 | 287.8 ± 17.5 | 310.03 ± 31.19 | 0.191 | 0.828 | 0.076 | - | - | - |
| Habituation, time in right side (sec) | ANOVA | normal | 279.37 ± 26.98 | 276.5 ± 23.9 | 359.86 ± 31.41 | 2.815 | *0.078* | 0.507 | 0.997 | 0.116 | 0.110 |  | ANOVA | normal | 292.11 ± 32.26 | 311.45 ± 17.53 | 289.36 ± 31.26 | 0.189 | 0.829 | 0.076 | - | - | - |
|  |  |  |  |  |  |  |  |  |  |  |  |  |  |  |  |  |  |  |  |  |  |  |  |
|  | Cohort 1 | | | | | | | |  |  |  |  | Cohort 2 | | | | | | | |  |  |  |
| Habituation, time spend in left vs right half side | test | data structure |  | | | F | p-value | power |  |  |  |  | test | data structure |  | | | F | p-value | power |  |  |  |
| - All mice | repeated measures | sphericity assumed |  | | | 0.026 | 0.873 | 0.053 |  |  |  |  | repeated measures | sphericity assumed |  | | | 0.018 | 0.894 | 0.052 |  |  |  |
| - WT | repeated measures | sphericity assumed |  |  |  | 0.575 | 0.466 | 0.106 |  |  |  |  | repeated measures | sphericity assumed |  |  |  | 0.054 | 0.823 | 0.055 |  |  |  |
| - Het | repeated measures | sphericity assumed |  |  |  | 0.951 | 0.355 | 0.141 |  |  |  |  | repeated measures | sphericity assumed |  |  |  | 0.456 | 0.519 | 0.092 |  |  |  |
| - KO | repeated measures | sphericity assumed |  |  |  | 3.643 | *0.093* | 0.390 |  |  |  |  | repeated measures | sphericity assumed |  |  |  | 0.110 | 0.748 | 0.060 |  |  |  |
|  |  |  |  |  |  |  |  |  |  |  |  |  |  |  |  |  |  |  |  |  |  |  |  |
| **Novel object recognition: training with 2 identical objects** |  |  |  |  |  |  |  |  |  |  |  |  |  |  |  |  |  |  |  |  |  |  |  |
|  | Cohort 1 | | | | | | | | | | |  | Cohort 2 | | | | | | | | | | |
|  | test | data structure | WT | Het | KO | genotype | | | pairwise comparisons | | |  | test | data structure | WT | Het | KO | genotype | | | pairwise comparisons | | |
|  |  |  |  |  |  | F | p-value | power | WT vs Het | WT vs KO | Het vs KO |  |  |  |  |  |  | F | p-value | power | WT vs Het | WT vs KO | Het vs KO |
| Identical objects, total distance (cm) | ANOVA | normal | 2686.26 ± 288.94 | 2325.75 ± 301.85 | 1650.77 ± 232.19 | 3.416 | **0.048** | 0.592 | 0.627 | **0.039** | 0.241 |  | ANOVA | normal | 2058.32 ± 200.69 | 2007.51 ± 150.68 | 1614.69 ± 99.18 | 2.775 | *0.082* | 0.495 | 0.970 | 0.112 | 0.156 |
| Identical objects, time in left side (sec) | ANOVA | normal | 155.81 ± 12.58 | 153.67 ± 15.55 | 160.56 ± 28.05 | 0.033 | 0.968 | 0.054 | - | - | - |  | ANOVA | normal | 144.05 ± 14.41 | 143.63 ± 13.8 | 191.02 ± 18.41 | 3.012 | *0.068* | 0.530 | 1.000 | 0.121 | 0.103 |
| Identical objects, time in right side (sec) | ANOVA | normal | 143.94 ± 12.63 | 146.17 ± 15.53 | 138.93 ± 28.04 | 0.036 | 0.964 | 0.055 | - | - | - |  | ANOVA | normal | 155.62 ± 14.47 | 155.98 ± 13.75 | 108.56 ± 18.33 | 3.034 | *0.067* | 0.533 | 1.000 | 0.119 | 0.102 |
| Identical objects, number of side switches | ANOVA | normal | 16.18 ± 1.56 | 14.2 ± 1.78 | 12.55 ± 1.97 | 1.062 | 0.360 | 0.216 | - | - | - |  | ANOVA | normal | 15.62 ± 2.28 | 14.33 ± 0.91 | 15.1 ± 1.47 | 0.158 | 0.855 | 0.072 | - | - | - |
| Identical objects, number of left object exploration | Kruskal-Wallis | non normal | 23.45 ± 4.55 | 18.3 ± 4.04 | 10.88 ± 2.37 | 4.149 | 0.126 | NA | 0.614 | *0.081* | 0.408 |  | ANOVA | normal | 19.62 ± 2.67 | 20.88 ± 3.14 | 17 ± 2.04 | 0.606 | 0.554 | 0.139 | - | - | - |
| Identical objects, number of right object exploration | Kruskal-Wallis | non normal | 40.63 ± 11.91 | 35.9 ± 14.72 | 17.55 ± 4.8 | 1.899 | 0.387 | NA | - | - | - |  | ANOVA | normal | 23.5 ± 3.88 | 21 ± 3.05 | 16.1 ± 2.88 | 1.371 | 0.273 | 0.266 | - | - | - |
| Identical objects, time exploring left object (sec) | Kruskal-Wallis | non normal | 23.67 ± 5.21 | 16.13 ± 3.64 | 9.19 ± 3.39 | 4.029 | 0.133 | NA | 0.426 | *0.064* | 0.517 |  | ANOVA | normal | 20.18 ± 2.53 | 24.06 ± 5.01 | 12 ± 2.71 | 3.059 | *0.066* | 0.536 | 0.746 | 0.271 | *0.059* |
| Identical objects, time exploring right object (sec) | Kruskal-Wallis | non normal | 19.99 ± 4.21 | 22.76 ± 7.04 | 7.81 ± 2.58 | 4.927 | *0.085* | NA | - | - | - |  | ANOVA | normal | 29.45 ± 4.85 | 25.24 ± 3.99 | 14.4 ± 3.72 | 3.586 | **0.043** | 0.608 | 0.768 | **0.045** | 0.161 |
| Identical objects, latency to observe left object (sec) | Kruskal-Wallis | non normal | 18.68 ± 6.63 | 21.56 ± 12.68 | 20.09 ± 4.37 | 1.570 | 0.456 | NA | - | - | - |  | Kruskal-Wallis | non normal | 12.06 ± 4.79 | 21.93 ± 8.67 | 21.19 ± 6.62 | 1.584 | 0.453 | NA | - | - | - |
| Identical objects, latency to observe right object (sec) | Kruskal-Wallis | non normal | 21.61 ± 5.29 | 18.42 ± 9.5 | 18.38 ± 3.73 | 1.865 | 0.393 | NA | - | - | - |  | Kruskal-Wallis | non normal | 5.98 ± 2.01 | 9.06 ± 4.61 | 17.51 ± 4.81 | 5.698 | *0.058* | NA | - | - | - |
| Identical objects, total number of object exploration | Kruskal-Wallis | non normal | 64.09 ± 16.02 | 54.2 ± 18.43 | 28.44 ± 7.09 | 2.901 | 0.235 | NA | - | - | - |  | ANOVA | normal | 43.12 ± 5.74 | 41.88 ± 5.25 | 33.1 ± 4.71 | 1.150 | 0.334 | 0.228 | - | - | - |
| Identical objects, total time exploring objects (sec) | Kruskal-Wallis | non normal | 43.67 ± 8.87 | 38.9 ± 10.46 | 17 ± 5.89 | 5.199 | *0.074* | NA | - | - | - |  | ANOVA | normal | 49.64 ± 7.05 | 49.3 ± 7.9 | 26.41 ± 6.05 | 3.795 | **0.037** | 0.634 | 0.999 | *0.070* | *0.065* |
| Identical objects, latency to observe any object (sec) | Kruskal-Wallis | non normal | 13.48 ± 5 | 14.48 ± 9.8 | 14.62 ± 3.69 | 2.387 | 0.303 | NA | - | - | - |  | Kruskal-Wallis | non normal | 2.78 ± 1.32 | 7.41 ± 4.78 | 15.15 ± 4.95 | 7.373 | **0.025** | NA | 0.883 | **0.028** | **0.015** |
|  |  |  |  |  |  |  |  |  |  |  |  |  |  |  |  |  |  |  |  |  |  |  |  |
| Repeated measure, Left vs right | Cohort 1 | | | | | | | |  |  |  |  | Cohort 2 | | | | | | | |  |  |  |
| AA: time spent sniffing left vs right object | test | data structure |  | | | F | p-value | power |  |  |  |  | test | data structure |  | | | F | p-value | power |  |  |  |
| - All mice | repeated measures | sphericity assumed |  | | | 0.052 | 0.822 | 0.056 |  |  |  |  | repeated measures | sphericity assumed |  | | | 4.013 | *0.056* | 0.488 |  |  |  |
| - WT | repeated measures | sphericity assumed |  |  |  | 1.216 | 0.296 | 0.170 |  |  |  |  | repeated measures | sphericity assumed |  |  |  | 8.413 | **0.023** | 0.702 |  |  |  |
| - Het | repeated measures | sphericity assumed |  |  |  | 2.694 | 0.135 | 0.312 |  |  |  |  | repeated measures | sphericity assumed |  |  |  | 0.071 | 0.797 | 0.056 |  |  |  |
| - KO | repeated measures | sphericity assumed |  |  |  | 1.075 | 0.330 | 0.150 |  |  |  |  | repeated measures | sphericity assumed |  |  |  | 0.982 | 0.348 | 0.144 |  |  |  |
|  |  |  |  |  |  |  |  |  |  |  |  |  |  |  |  |  |  |  |  |  |  |  |  |
| AA: number of left vs right object interactions | test | data structure |  | | | F | p-value | power |  |  |  |  | test | data structure |  | | | F | p-value | power |  |  |  |
| - All mice | repeated measures | sphericity assumed |  | | | 8.654 | **0.006** | 0.811 |  |  |  |  | repeated measures | sphericity assumed |  | | | 0.287 | 0.597 | 0.081 |  |  |  |
| - WT | repeated measures | sphericity assumed |  |  |  | 4.304 | *0.065* | 0.466 |  |  |  |  | repeated measures | sphericity assumed |  |  |  | 1.312 | 0.290 | 0.169 |  |  |  |
| - Het | repeated measures | sphericity assumed |  |  |  | 2.447 | 0.152 | 0.288 |  |  |  |  | repeated measures | sphericity assumed |  |  |  | 0.001 | 0.974 | 0.050 |  |  |  |
| - KO | repeated measures | sphericity assumed |  |  |  | 6.250 | **0.037** | 0.593 |  |  |  |  | repeated measures | sphericity assumed |  |  |  | 0.293 | 0.602 | 0.077 |  |  |  |
|  |  |  |  |  |  |  |  |  |  |  |  |  |  |  |  |  |  |  |  |  |  |  |  |
| AA: time spend in left vs right half | test | data structure |  | | | F | p-value | power |  |  |  |  | test | data structure |  | | | F | p-value | power |  |  |  |
| - All mice | repeated measures | sphericity assumed |  | | | 0.403 | 0.531 | 0.094 |  |  |  |  | repeated measures | sphericity assumed |  | | | 1.330 | 0.259 | 0.199 |  |  |  |
| - WT | repeated measures | sphericity assumed |  |  |  | 0.221 | 0.648 | 0.071 |  |  |  |  | repeated measures | sphericity assumed |  |  |  | 0.160 | 0.064 | 0.071 |  |  |  |
| - Het | repeated measures | sphericity assumed |  |  |  | 0.058 | 0.815 | 0.055 |  |  |  |  | repeated measures | sphericity assumed |  |  |  | 0.201 | 0.666 | 0.068 |  |  |  |
| - KO | repeated measures | sphericity assumed |  |  |  | 0.149 | 0.710 | 0.063 |  |  |  |  | repeated measures | sphericity assumed |  |  |  | 5.035 | *0.052* | 0.517 |  |  |  |
|  |  |  |  |  |  |  |  |  |  |  |  |  |  |  |  |  |  |  |  |  |  |  |  |
| **Novel object recognition: test with one new object** |  |  |  |  |  |  |  |  |  |  |  |  |  |  |  |  |  |  |  |  |  |  |  |
|  | Cohort 1 | | | | | | | | | | |  | Cohort 2 | | | | | | | | | | |
|  | test | data structure | WT | Het | KO | genotype | | | pairwise comparisons | | |  | test | data structure | WT | Het | KO | genotype | | | pairwise comparisons | | |
|  |  |  |  |  |  | F | p-value | power | WT vs Het | WT vs KO | Het vs KO |  |  |  |  |  |  | F | p-value | power | WT vs Het | WT vs KO | Het vs KO |
| Novel object, total distance (cm) | ANOVA | normal | 2099.44 ± 251.03 | 1493.85 ± 300.17 | 891.21 ± 189.6 | 5.553 | **0.010** | 0.812 | 0.218 | **0.007** | 0.253 |  | ANOVA | normal | 1800.47 ± 138.78 | 1469.05 ± 143.3 | 1207.8 ± 112.83 | 5.059 | **0.015** | 0.766 | 0.213 | **0.011** | 0.333 |
| Novel object, time in new object side (sec) | ANOVA | normal | 152.37 ± 15.38 | 153.3 ± 24.59 | 160.48 ± 37.51 | 0.028 | 0.973 | 0.054 | 1.000 | 0.974 | 0.980 |  | ANOVA | normal | 150.51 ± 10.9 | 132.9 ± 17.64 | 186.43 ± 18.32 | 2.874 | *0.076* | 0.510 | 0.749 | 0.298 | *0.069* |
| Novel object, time in pre-exposed object side (sec) | ANOVA | normal | 146.83 ± 15.41 | 145.93 ± 24.75 | 138.47 ± 37.87 | 0.029 | 0.971 | 0.054 | 1.000 | 0.972 | 0.979 |  | ANOVA | normal | 149.1 ± 10.95 | 166.89 ± 17.67 | 113.3 ± 18.32 | 2.870 | *0.076* | 0.509 | 0.745 | 0.301 | *0.069* |
| Novel object, number of side switches | ANOVA | normal | 14.45 ± 1.13 | 10.4 ± 2.4 | 6.77 ± 1.61 | 4.668 | **0.018** | 0.737 | 0.241 | **0.014** | 0.353 |  | ANOVA | normal | 14.75 ± 1.06 | 9.22 ± 1.01 | 10.1 ± 1.24 | 6.441 | **0.006** | 0.863 | 0.007 | **0.021** | 0.841 |
| Novel object, number of new object exploration | Kruskal-Wallis | non normal | 22.18 ± 4.16 | 16.8 ± 4.65 | 3.33 ± 0.89 | 12.974 | **0.002** | NA | 0.292 | **0.001** | **0.014** |  | ANOVA | normal | 23.5 ± 3.01 | 19 ± 2.59 | 8.9 ± 1.03 | 11.261 | **0.000** | 0.984 | 0.371 | **0.000** | **0.009** |
| Novel object, number of pre-exposed object exploration | Kruskal-Wallis | non normal | 23.36 ± 7.24 | 15.2 ± 5.21 | 3.88 ± 0.91 | 6.591 | **0.037** | NA | 0.758 | **0.012** | *0.080* |  | ANOVA | normal | 11.87 ± 1.43 | 10.66 ± 2.3 | 7.5 ± 0.93 | 1.954 | 0.164 | 0.364 | 0.869 | 0.166 | 0.354 |
| Novel object, time exploring new object (sec) | Kruskal-Wallis | non normal | 22.81 ± 4.67 | 16.62 ± 4.08 | 1.99 ± 0.82 | 14.303 | **0.001** | NA | 0.404 | **0.001** | **0.005** |  | ANOVA | normal | 34.76 ± 4.88 | 27.43 ± 3.33 | 9.68 ± 1.92 | 14.840 | **0.000** | 0.997 | 0.315 | **0.000** | **0.002** |
| Novel object, time exploring pre-exposed object (sec) | Kruskal-Wallis | non normal | 12.79 ± 3 | 12.51 ± 4.33 | 2.11 ± 1.01 | 10.323 | **0.006** | NA | 0.707 | **0.003** | **0.010** |  | Kruskal-Wallis | non normal | 12.07 ± 2.83 | 11.5 ± 2.73 | 5.33 ± 0.97 | 5.602 | *0.061* | NA | - | - | - |
| Novel object, latency to observe new object (sec) | Kruskal-Wallis | non normal | 19.54 ± 7.57 | 68.32 ± 31.56 | 95.13 ± 18.8 | 2.708 | 0.258 | NA | 0.246 | 0.195 | 0.982 |  | Kruskal-Wallis | non normal | 12.75 ± 6.85 | 31.7 ± 15.38 | 56.92 ± 20.92 | 2.636 | 0.268 | NA | - | - | - |
| Novel object, latency to observe pre-exposed object (sec) | Kruskal-Wallis | non normal | 31.51 ± 13.61 | 47.5 ± 29.39 | 80.95 ± 19.46 | 6.208 | **0.045** | NA | 0.730 | **0.019** | **0.049** |  | Kruskal-Wallis | non normal | 29.67 ± 11.37 | 51.96 ± 23.17 | 57.7 ± 20.03 | 0.504 | 0.777 | NA | - | - | - |
| Novel object, total number of object exploration | Kruskal-Wallis | non normal | 45.54 ± 11.08 | 32 ± 9.73 | 7.22 ± 1.68 | 11.947 | **0.003** | NA | 0.314 | **0.001** | **0.018** |  | ANOVA | normal | 35.37 ± 3.91 | 29.66 ± 4.29 | 16.4 ± 1.49 | 8.752 | **0.001** | 0.949 | 0.477 | **0.001** | **0.021** |
| Novel object, total time exploring objects (sec) | Kruskal-Wallis | non normal | 35.61 ± 7.26 | 29.13 ± 7.44 | 4.1 ± 1.73 | 13.948 | **0.001** | NA | 0.558 | **0.001** | **0.004** |  | ANOVA | normal | 46.84 ± 6.45 | 38.93 ± 4.8 | 15.02 ± 2.19 | 13.748 | **0.000** | 0.995 | 0.466 | **0.000** | **0.002** |
| Novel object, latency to observe any object (sec) | Kruskal-Wallis | non normal | 11.15 ± 6.67 | 41.52 ± 28.12 | 66.67 ± 17.69 | 9.609 | **0.008** | NA | 0.466 | **0.003** | **0.024** |  | Kruskal-Wallis | non normal | 12.16 ± 6.85 | 11.66 ± 7.96 | 28.03 ± 13.03 | 1.730 | 0.412 | NA | - | - | - |
|  |  |  |  |  |  |  |  |  |  |  |  |  |  |  |  |  |  |  |  |  |  |  |  |
| Repeated measure, new vs pre-exposed | Cohort 1 | | | | | | | |  |  |  |  | Cohort 2 | | | | | | | |  |  |  |
| AB: time spent sniffing new vs pre-exposed object | test | data structure |  | | | F | p-value | power |  |  |  |  | test | data structure |  | | | F | p-value | power |  |  |  |
| - All mice | repeated measures | sphericity assumed |  | | | 7.385 | **0.011** | 0.747 |  |  |  |  | repeated measures | sphericity assumed |  | | | 30.865 | **0.000** | 1.000 |  |  |  |
| - WT | repeated measures | sphericity assumed |  |  |  | 11.085 | **0.008** | 0.850 |  |  |  |  | repeated measures | sphericity assumed |  |  |  | 23.202 | **0.002** | 0.983 |  |  |  |
| - Het | repeated measures | sphericity assumed |  |  |  | 1.078 | 0.326 | 0.154 |  |  |  |  | repeated measures | sphericity assumed |  |  |  | 17.948 | **0.003** | 0.958 |  |  |  |
| - KO | repeated measures | sphericity assumed |  |  |  | 0.030 | 0.867 | 0.053 |  |  |  |  | repeated measures | sphericity assumed |  |  |  | 4.175 | *0.071* | 0.446 |  |  |  |
|  |  |  |  |  |  |  |  |  |  |  |  |  |  |  |  |  |  |  |  |  |  |  |  |
| AB: number of new vs pre-exposed object interactions | test | data structure |  | | | F | p-value | power |  |  |  |  | test | data structure |  | | | F | p-value | power |  |  |  |
| - All mice | repeated measures | sphericity assumed |  | | | 0.002 | 0.967 | 0.050 |  |  |  |  | repeated measures | sphericity assumed |  | | | 21.950 | **0.000** | 0.995 |  |  |  |
| - WT | repeated measures | sphericity assumed |  |  |  | 0.083 | 0.779 | 0.058 |  |  |  |  | repeated measures | sphericity assumed |  |  |  | 19.312 | **0.003** | 0.962 |  |  |  |
| - Het | repeated measures | sphericity assumed |  |  |  | 0.822 | 0.388 | 0.128 |  |  |  |  | repeated measures | sphericity assumed |  |  |  | 12.077 | **0.008** | 0.859 |  |  |  |
| - KO | repeated measures | sphericity assumed |  |  |  | 0.649 | 0.444 | 0.110 |  |  |  |  | repeated measures | sphericity assumed |  |  |  | 1.189 | 0.304 | 0.165 |  |  |  |
|  |  |  |  |  |  |  |  |  |  |  |  |  |  |  |  |  |  |  |  |  |  |  |  |
| AB: time spend in new vs pre-exposed half | test | data structure |  | | | F | p-value | power |  |  |  |  | test | data structure |  | | | F | p-value | power |  |  |  |
| - All mice | repeated measures | sphericity assumed |  | | | 0.146 | 0.705 | 0.066 |  |  |  |  | repeated measures | sphericity assumed |  | | | 0.624 | 0.437 | 0.119 |  |  |  |
| - WT | repeated measures | sphericity assumed |  |  |  | 0.032 | 0.861 | 0.053 |  |  |  |  | repeated measures | sphericity assumed |  |  |  | 0.004 | 0.951 | 0.050 |  |  |  |
| - Het | repeated measures | sphericity assumed |  |  |  | 0.022 | 0.885 | 0.052 |  |  |  |  | repeated measures | sphericity assumed |  |  |  | 0.926 | 0.364 | 0.136 |  |  |  |
| - KO | repeated measures | sphericity assumed |  |  |  | 0.085 | 0.778 | 0.058 |  |  |  |  | repeated measures | sphericity assumed |  |  |  | 3.981 | *0.077* | 0.430 |  |  |  |
|  |  |  |  |  |  |  |  |  |  |  |  |  |  |  |  |  |  |  |  |  |  |  |  |
| **Marble burying** |  |  |  |  |  |  |  |  |  |  |  |  |  |  |  |  |  |  |  |  |  |  |  |
|  | Cohort 1 | | | | | | | | | | |  | Cohort 2 | | | | | | | | | | |
|  | test | data structure | WT | Het | KO | genotype | | | pairwise comparisons | | |  | test | data structure | WT | Het | KO | genotype | | | pairwise comparisons | | |
|  |  |  |  |  |  | F | p-value | power | WT vs Het | WT vs KO | Het vs KO |  |  |  |  |  |  | F | p-value | power | WT vs Het | WT vs KO | Het vs KO |
| Number of burried marbles (over 20) | Kruskal-Wallis | non normal | 13.9 ± 1.62 | 14.6 ± 1.55 | 2.25 ± 0.52 | 12.074 | **0.000** | 1.000 | 0.949404203324744 | **0.001** | **0.000** |  | Kruskal-Wallis | non normal | 13.25 ± 2.25 | 12.88 ± 1.26 | 5 ± 1.83 | 7.015 | **0.004** | 0.892 | 0.990 | **0.010** | **0.011** |
|  |  |  |  |  |  |  |  |  |  |  |  |  |  |  |  |  |  |  |  |  |  |  |  |
| **4-object preference test, exploration** |  |  |  |  |  |  |  |  |  |  |  |  |  |  |  |  |  |  |  |  |  |  |  |
|  | Cohort 1 | | | | | | | | | | |  | Cohort 2 | | | | | | | | | | |
|  | test | data structure | WT | Het | KO | genotype | | | pairwise comparisons | | |  | test | data structure | WT | Het | KO | genotype | | | pairwise comparisons | | |
|  |  |  |  |  |  | F | p-value | power | WT vs Het | WT vs KO | Het vs KO |  |  |  |  |  |  | F | p-value | power | WT vs Het | WT vs KO | Het vs KO |
| Time exploring all the objects | Kruskal-Wallis | non normal | 62.97 ± 6.27 | 64.55 ± 7.23 | 39.01 ± 3.68 | 9.158 | **0.010** | NA | 0.940 | **0.008** | **0.007** |  | Kruskal-Wallis | non normal | 112.11 ± 24.05 | 107.3 ± 6.9 | 66.15 ± 6.41 | 9.047 | **0.011** | NA | 0.399 | *0.056* | **0.003** |
| Total number of object interactions | ANOVA | normal | 61.81 ± 4.92 | 76.4 ± 6.05 | 62.12 ± 3.74 | 2.635 | *0.091* | 0.477 | 0.116 | 0.999 | 0.168 |  | ANOVA | normal | 117 ± 9.13 | 114 ± 6.7 | 102.44 ± 6.97 | 1.063 | 0.363 | 0.212 | - | - | - |
|  |  |  |  |  |  |  |  |  |  |  |  |  |  |  |  |  |  |  |  |  |  |  |  |
| **Nest building** |  |  |  |  |  |  |  |  |  |  |  |  |  |  |  |  |  |  |  |  |  |  |  |
|  | Cohort 1 | | | | | | | | | | |  | Cohort 2 | | | | | | | | | | |
|  | test | data structure | WT | Het | KO | genotype | | | pairwise comparisons | | |  | test | data structure | WT | Het | KO | genotype | | | pairwise comparisons | | |
|  |  |  |  |  |  | F | p-value | power | WT vs Het | WT vs KO | Het vs KO |  |  |  |  |  |  | F | p-value | power | WT vs Het | WT vs KO | Het vs KO |
| Nest shredded | Kruskal-Wallis | non normal | 1.9 ± 0.09 | 2 ± 0 | 1.55 ± 0.17 | 7.206 | **0.027** | NA | 0.583 | *0.038* | **0.011** |  | Kruskal-Wallis | non normal | 1.75 ± 0.16 | 1.88 ± 0.11 | 1.2 ± 0.24 | 3.800 | **0.037** | NA | 0.874 | 0.132 | **0.040** |
| Nest dispersion | Kruskal-Wallis | non normal | 1.9 ± 0.09 | 1.9 ± 0.09 | 2 ± 0 | 0.894 | 0.639 | NA | 0.996 | 0.717 | 0.681 |  | Kruskal-Wallis | non normal | 2 ± 0 | 2 ± 0 | 1.5 ± 0.26 | 5.508 | *0.064* | NA | - | - | - |
| Nest density | Kruskal-Wallis | non normal | 1.27 ± 0.19 | 0.7 ± 0.21 | 0.55 ± 0.29 | 5.233 | *0.073* | NA | - | - | - |  | Kruskal-Wallis | non normal | 1.25 ± 0.25 | 0.77 ± 0.27 | 0.9 ± 0.31 | 1.422 | 0.491 | NA | - | - | - |
| Nest shape | Kruskal-Wallis | non normal | 2.81 ± 0.18 | 2.2 ± 0.29 | 1.66 ± 0.37 | 7.122 | **0.028** | NA | 0.105 | **0.009** | 0.301 |  | Kruskal-Wallis | non normal | 2.25 ± 0.36 | 1.88 ± 0.38 | 1.1 ± 0.4 | 3.938 | 0.140 | NA | - | - | - |
| Nest walls | Kruskal-Wallis | non normal | 1.18 ± 0.18 | 1.2 ± 0.29 | 0.33 ± 0.23 | 6.980 | **0.031** | NA | 0.992 | **0.019** | **0.022** |  | Kruskal-Wallis | non normal | 1.25 ± 0.25 | 0.77 ± 0.22 | 0.5 ± 0.26 | 4.470 | 0.106 | NA | - | - | - |
| Nest total score | Kruskal-Wallis | non normal | 9.09 ± 0.47 | 8 ± 0.64 | 6.11 ± 0.82 | 7.960 | **0.019** | NA | 0.228 | **0.005** | 0.107 |  | Kruskal-Wallis | non normal | 8.5 ± 0.88 | 7.33 ± 0.83 | 5.2 ± 1.2 | 4.137 | *0.126* | NA | - | - | - |
