# Supplementary material for: Behavioral Phenotyping of an Improved Mouse Model of Phelan–McDermid Syndrome with a Complete Deletion of the Shank3 Gene
Source: eNeuro. 2018 Oct 5;5(3):ENEURO.0046-18.2018. doi: 10.1523/ENEURO.0046-18.2018 (PMC6175061; doi:10.1523/ENEURO.0046-18.2018)
Supplement: Extended Data Table 10-1 — Individual results and statistical analyses for cohorts 1 and 2 related to the hyper-reactivity and escape behavior. WT, wild-type mice; Het, heterozygous mice; KO, homozygous knockout mice. Group values are reported as means ± s.e.m. Red font indicates significant results (p < 0.05), orange font indicates trends (0.1 < p < 0.05). Download Table 10-1, DOCX file. [file sup_enu-eN-CFN-0046-18-s07.docx]

# Extended Tables

Extended Table 10-1

| **Reflexes and reactions to simple stimuli** |  |  |  |  |  |  |  |  |  |  |  |  |  |  |  |  |  |  |  |  |  |  |  |
| --- | --- | --- | --- | --- | --- | --- | --- | --- | --- | --- | --- | --- | --- | --- | --- | --- | --- | --- | --- | --- | --- | --- | --- |
|  | Cohort 1 | | | | | | | | | | |  | Cohort 2 | | | | | | | | | | |
|  | test | data structure | WT | Het | KO | genotype | | | pairwise comparisons | | |  | test | data structure | WT | Het | KO | genotype | | | pairwise comparisons | | |
|  |  |  |  |  |  | F | p-value | power | WT vs Het | WT vs KO | Het vs KO |  |  |  |  |  |  | F | p-value | power | WT vs Het | WT vs KO | Het vs KO |
| Touch escape | Kruskal-Wallis | non normal | 1.36 ± 0.15 | 1.1 ± 0.17 | 1.88 ± 0.2 | 7.502 | **0.023** | NA | 0.357 | 0.063 | **0.007** |  | Kruskal-Wallis | non normal | 1.12 ± 0.12 | 1.22 ± 0.14 | 2.1 ± 0.23 | 11.007 | **0.004** | NA | 0.730 | **0.003** | **0.007** |
| Positional passivity (sum) | Kruskal-Wallis | non normal | 1.36 ± 0.15 | 1 ± 0 | 2.22 ± 0.27 | 13.351 | **0.001** | NA | 0.151 | **0.021** | **0.000** |  | Kruskal-Wallis | non normal | 3.25 ± 0.25 | 2.77 ± 0.22 | 3.4 ± 0.16 | 4.241 | 0.120 | NA | - | - | - |
| Positional passivity (score) | Kruskal-Wallis | non normal | 2.45 ± 0.24 | 3 ± 0 | 1.22 ± 0.4 | 13.199 | **0.001** | NA | 0.153 | **0.000** | **0.021** |  | Kruskal-Wallis | non normal | 0.87 ± 0.22 | 1.33 ± 0.16 | 0.7 ± 0.15 | 5.738 | *0.057* | NA | - | - | - |
| Catalepsy (4 trials) | Kruskal-Wallis | non normal | 3.75 ± 0.84 | 3.52 ± 0.87 | 0.58 ± 0.49 | 11.431 | **0.003** | NA | 0.813 | **0.002** | **0.005** |  | Kruskal-Wallis | non normal | 1.93 ± 0.57 | 1.88 ± 0.5 | 0.55 ± 0.21 | 6.806 | **0.033** | NA | 0.913 | **0.025** | **0.027** |
| Trunk curl | Kruskal-Wallis | non normal | 1 ± 0 | 1 ± 0 | 1 ± 0 | NA | NA | NA | - | - | - |  | Kruskal-Wallis | non normal | 1 ± 0 | 0.77 ± 0.14 | 1 ± 0 | NA | NA | NA | - | - | - |
| Negative geotaxis, latency to turn | Kruskal-Wallis | non normal | 5.03 ± 0.65 | 6.5 ± 1.15 | 3.18 ± 0.98 | 5.840 | *0.054* | NA | - | - | - |  | Kruskal-Wallis | non normal | 9.08 ± 2.18 | 10.11 ± 3.83 | 3.26 ± 0.73 | 7.759 | 0.210 | NA | 0.380 | **0.007** | *0.063* |
|  |  |  |  |  |  |  |  |  |  |  |  |  |  |  |  |  |  |  |  |  |  |  |  |
| **Beam walking** |  |  |  |  |  |  |  |  |  |  |  |  |  |  |  |  |  |  |  |  |  |  |  |
|  | Cohort 1 | | | | | | | | | | |  | Cohort 2 | | | | | | | | | | |
|  | test | data structure | WT | Het | KO | genotype | | | pairwise comparisons | | |  | test | data structure | WT | Het | KO | genotype | | | pairwise comparisons | | |
|  |  |  |  |  |  | F | p-value | power | WT vs Het | WT vs KO | Het vs KO |  |  |  |  |  |  | F | p-value | power | WT vs Het | WT vs KO | Het vs KO |
| Latency to start crossing the large beam | Kruskal-Wallis | non normal | 4.11 ± 1.51 | 10.72 ± 6.38 | 6.36 ± 3.5 | 1.187 | 0.911 | NA | - | - | - |  | Kruskal-Wallis | non normal | 14.15 ± 5.9 | 10.88 ± 6.81 | 1.8 ± 0.87 | 5.388 | 0.068 | NA | - | - | - |
| Latency to start crossing the medium beam | Kruskal-Wallis | non normal | 18.81 ± 6.52 | 12.62 ± 7.67 | 14.97 ± 12.88 | 1.735 | 0.420 | NA | - | - | - |  | Kruskal-Wallis | non normal | 11.9 ± 7.29 | 1.11 ± 0.22 | 1.07 ± 0.32 | 3.086 | 0.214 | NA | - | - | - |
| Latency to start crossing the small beam | Kruskal-Wallis | non normal | 52.86 ± 5.55 | 48.85 ± 10.79 | 26.55 ± 13.45 | 4.909 | *0.086* | NA | - | - | - |  | Kruskal-Wallis | non normal | 57.25 ± 12.24 | 43.16 ± 11.22 | 40.17 ± 8.91 | 1.140 | 0.566 | NA | - | - | - |
|  |  |  |  |  |  |  |  |  |  |  |  |  |  |  |  |  |  |  |  |  |  |  |  |
| **Escape behavior** |  |  |  |  |  |  |  |  |  |  |  |  |  |  |  |  |  |  |  |  |  |  |  |
|  | Cohort 1 | | | | | | | | | | |  | Cohort 2 | | | | | | | | | | |
|  | test | data structure | WT | Het | KO | genotype | | | pairwise comparisons | | |  | test | data structure | WT | Het | KO | genotype | | | pairwise comparisons | | |
|  |  |  |  |  |  | F | p-value | power | WT vs Het | WT vs KO | Het vs KO |  |  |  |  |  |  | F | p-value | power | WT vs Het | WT vs KO | Het vs KO |
| Buried food, number of escape attempts | Kruskal-Wallis | non normal | 0.36 ± 0.27 | 0.8 ± 0.38 | 0.77 ± 0.66 | 1.230 | 0.541 | NA | - | - | - |  | Kruskal-Wallis | non normal | 1 ± 0.75 | 0.55 ± 0.55 | 0.1 ± 0.1 | 1.087 | 0.581 | NA | - | - | - |
| Buried food, percentage of mice escaping | Kruskal-Wallis | non normal | 18.18 ± 12.19 | 40 ± 16.32 | 22.22 ± 14.69 | 1.358 | 0.500 | NA | - | - | - |  | Kruskal-Wallis | non normal | 25 ± 16.36 | 11.11 ± 11.11 | 10 ± 10 | 0.904 | 0.636 | NA | - | - | - |
| 4-object exploration, number of escape attempts | Kruskal-Wallis | non normal | 0.18 ± 0.12 | 2.9 ± 1.26 | 6.75 ± 1.82 | 12.629 | **0.002** | NA | *0.065* | **0.000** | *0.077* |  | Kruskal-Wallis | non normal | 0.83 ± 0.54 | 1.11 ± 0.58 | 1.6 ± 1.28 | 0.091 | 0.955 | NA | - | - | - |
| 4-object exploration, percentage of mice escaping | Kruskal-Wallis | non normal | 18.18 ± 12.19 | 60 ± 16.32 | 75 ± 16.36 | 6.593 | **0.037** | NA | **0.060** | **0.016** | 0.534 |  | Kruskal-Wallis | non normal | 33.33 ± 21.08 | 11.11 ± 11.11 | 30 ± 15.27 | 1.251 | 0.535 | NA | - | - | - |
| Marble burying, number of escape attempts | Kruskal-Wallis | non normal | 1.81 ± 1.24 | 6.2 ± 2.29 | 15.37 ± 2 | 15.225 | **0.000** | NA | **0.042** | **0.000** | *0.053* |  | ANOVA | normal | 7.75 ± 2.03 | 15.55 ± 2.51 | 16.6 ± 4.1 | 4.514 | 0.105 | 0.289 | - | - | - |
| Marble burying, percentage of mice escaping | Kruskal-Wallis | non normal | 27.27 ± 14.08 | 90 ± 10 | 100 ± 0 | 14.098 | **0.001** | NA | **0.002** | **0.001** | 0.654 |  | Kruskal-Wallis | non normal | 75 ± 16.36 | 100 ± 0 | 100 ± 0 | 4.940 | *0.085* | NA | - | - | - |
