# Supplementary material for: Behavioral Phenotyping of an Improved Mouse Model of Phelan–McDermid Syndrome with a Complete Deletion of the Shank3 Gene
Source: eNeuro. 2018 Oct 5;5(3):ENEURO.0046-18.2018. doi: 10.1523/ENEURO.0046-18.2018 (PMC6175061; doi:10.1523/ENEURO.0046-18.2018)
Supplement: Extended Data Table 11-1 — Individual results and statistical analyses for cohorts 1 and 2 related to stereotypies, repetitive behavior, perseveration and cognitive flexibility. WT, wild-type mice; Het, heterozygous mice; KO, homozygous knockout mice. Group values are reported as means ± s.e.m. Red font indicates significant results (p < 0.05), orange font indicates trends (0.1 < p < 0.05). Download Table 11-1, DOCX file. [file sup_enu-eN-CFN-0046-18-s08.docx]

# Extended Tables

Extended Table 11-1

| **Stereotypies in open field** |  |  |  |  |  |  |  |  |  |  |  |  |  |  |  |  |  |  |  |  |  |  |  |  |  |  |  |  |  |
| --- | --- | --- | --- | --- | --- | --- | --- | --- | --- | --- | --- | --- | --- | --- | --- | --- | --- | --- | --- | --- | --- | --- | --- | --- | --- | --- | --- | --- | --- |
|  | Cohort 1 | | | | | | | | | | |  |  |  |  | Cohort 2 | | | | | | | | | | |  |  |  |
|  | test | data structure | WT | Het | KO | genotype | | | pairwise comparisons | | |  |  |  |  | test | data structure | WT | Het | KO | genotype | | | pairwise comparisons | | |  |  |  |
|  |  |  |  |  |  | F | p-value | power | WT vs Het | WT vs KO | Het vs KO |  |  |  |  |  |  |  |  |  | F | p-value | power | WT vs Het | WT vs KO | Het vs KO |  |  |  |
| Grooming, total duration (sec) | Kruskal-Wallis | non normal | 58.56 ± 9.57 | 48.81 ± 6.62 | 60.84 ± 8.18 | 0.572 | 0.530 | NA | - | - | - |  |  |  |  | Kruskal-Wallis | non normal | 78.64 ± 9.34 | 77.91 ± 6.13 | 120.99 ± 13.09 | 7.262 | **0.026** | NA | 0.671 | **0.045** | **0.012** |  |  |  |
| Grooming, number of bouts | ANOVA | normal | 26.54 ± 2.17 | 19.6 ± 2.14 | 25.11 ± 2.34 | 2.789 | *0.079* | 0.503 | *0.078* | 0.893 | 0.220 |  |  |  |  | ANOVA | normal | 23.87 ± 2.02 | 25.22 ± 1.73 | 28.9 ± 3 | 1.189 | 0.322 | 0.235 | - | - | - |  |  |  |
| Jumping, total duration (sec) | Kruskal-Wallis | non normal | 0.13 ± 0.09 | 0 ± 0 | 0.09 ± 0.06 | 0.921 | 0.325 | NA | - | - | - |  |  |  |  | Kruskal-Wallis | non normal | 0 ± 0 | 0 ± 0 | 0.27 ± 0.2 | 3.531 | 0.171 | NA | - | - | - |  |  |  |
| Jumping, number | Kruskal-Wallis | non normal | 0.63 ± 0.38 | 0 ± 0 | 0.22 ± 0.14 | 1.640 | 0.214 | NA | - | - | - |  |  |  |  | Kruskal-Wallis | non normal | 0 ± 0 | 0 ± 0 | 0.6 ± 0.42 | 3.531 | 0.171 | NA | - | - | - |  |  |  |
| Rotation, total duration (sec) | Kruskal-Wallis | non normal | 0.37 ± 0.09 | 2.35 ± 1.53 | 7.63 ± 5.76 | 1.409 | 0.013 | NA | - | - | - |  |  |  |  | Kruskal-Wallis | non normal | 0.42 ± 0.21 | 0.55 ± 0.17 | 1.14 ± 0.41 | 2.739 | 0.254 | NA | - | - | - |  |  |  |
| Rotation, number | Kruskal-Wallis | non normal | 1.63 ± 0.41 | 2.3 ± 0.61 | 9.55 ± 3.47 | 5.373 | **0.024** | NA | 0.963 | **0.015** | **0.030** |  |  |  |  | Kruskal-Wallis | non normal | 1.62 ± 0.8 | 2.11 ± 0.73 | 3.1 ± 0.92 | 2.273 | 0.321 | NA | - | - | - |  |  |  |
| Twitching/shaking, total duration (sec) | Kruskal-Wallis | non normal | 0.33 ± 0.1 | 0.98 ± 0.62 | 0.54 ± 0.12 | 0.809 | 0.548 | NA | - | - | - |  |  |  |  | Kruskal-Wallis | non normal | 0.22 ± 0.11 | 0.38 ± 0.09 | 0.71 ± 0.17 | 7.522 | *0.023* | NA | 0.263 | **0.006** | 0.106 |  |  |  |
| Twitching/shaking, number | Kruskal-Wallis | non normal | 2.27 ± 0.61 | 3.4 ± 1.64 | 2.88 ± 0.65 | 0.287 | 0.707 | NA | - | - | - |  |  |  |  | Kruskal-Wallis | non normal | 1 ± 0.46 | 1.77 ± 0.46 | 3.1 ± 0.7 | 5.766 | *0.056* | NA | - | - | - |  |  |  |
|  |  |  |  |  |  |  |  |  |  |  |  |  |  |  |  |  |  |  |  |  |  |  |  |  |  |  |  |  |  |
| **Repetitive novel object contact task, object preference, time** | | |  |  |  |  |  |  |  |  |  |  |  |  |  |  |  |  |  |  |  |  |  |  |  |  |  |  |  |
|  | Cohort 1 | | | | | | | | | | |  |  |  |  | Cohort 2 | | | | | | | | | | |  |  |  |
| Time exploring the different objects | test | data structure |  | | | F | p-value | power | WT vs Het | WT vs KO | Het vs KO |  |  |  |  | test | data structure |  | | | F | p-value | power | WT vs Het | WT vs KO | Het vs KO |  |  |  |
| - object effect | repeated measures | sphericity violated |  | | | 1.836 | 0.147 | 0.460 | - | - | - |  |  |  |  | repeated measures | sphericity assumed |  | | | 9.106 | **0.000** | 0.980 | - | - | - |  |  |  |
| - object x genotype effect | repeated measures | sphericity violated |  |  |  | 1.478 | 0.197 | 0.542 | - | - | - |  |  |  |  | repeated measures | sphericity assumed |  |  |  | 1.166 | 0.335 | 0.362 | - | - | - |  |  |  |
| - genotype effect | repeated measures | sphericity violated |  |  |  | 4.714 | **0.018** | 0.739 | 0.854 | **0.013** | **0.010** |  |  |  |  | repeated measures | sphericity assumed |  |  |  | 3.894 | **0.036** | 0.641 | 0.801 | **0.023** | **0.029** |  |  |  |
|  |  |  |  |  |  |  |  |  |  |  |  |  |  |  |  |  |  |  |  |  |  |  |  |  |  |  |  |  |  |
| Individual objects | test | data structure | WT | Het | KO | genotype | | | pairwise comparisons | | |  |  |  |  | test | data structure | WT | Het | KO | genotype | | | pairwise comparisons | | |  |  |  |
|  |  |  |  |  |  | F | p-value | power | WT vs Het | WT vs KO | Het vs KO |  |  |  |  |  |  |  |  |  | F | p-value | power | WT vs Het | WT vs KO | Het vs KO |  |  |  |
| Time exploring the dice (sec) | ANOVA | normal | 13.05 ± 1.74 | 12.63 ± 1.39 | 11.42 ± 1.96 | 0.227 | 0.799 | 0.082 | - | - | - |  |  |  |  | Kruskal-Wallis | non normal | 21.63 ± 5.46 | 17.25 ± 2.84 | 12.77 ± 3.01 | 2.308 | 0.315 |  | - | - | - |  |  |  |
| Time exploring the jack (sec) | Kruskal-Wallis | non normal | 14.31 ± 2.33 | 20.07 ± 5.35 | 10.62 ± 2.65 | 1.762 | 0.414 | NA | - | - | - |  |  |  |  | Kruskal-Wallis | non normal | 32.03 ± 5.26 | 36.8 ± 7.72 | 19.62 ± 2.23 | 4.630 | *0.099* |  | - | - | - |  |  |  |
| Time exploring the Lego (sec) | ANOVA | normal | 17.69 ± 2.91 | 21.39 ± 3.23 | 10.54 ± 1.93 | 3.314 | *0.052* | 0.576 | 0.617 | 0.218 | **0.043** |  |  |  |  | ANOVA | normal | 34.86 ± 6.47 | 35.88 ± 3.89 | 18.91 ± 2.48 | 5.253 | **0.014** | 0.777 | 0.985 | **0.043** | **0.020** |  |  |  |
| Time exploring the pin (sec) | Kruskal-Wallis | non normal | 17.91 ± 4.33 | 10.45 ± 1.83 | 6.4 ± 1.27 | 5.854 | *0.054* | NA | - | - | - |  |  |  |  | Kruskal-Wallis | non normal | 23.57 ± 9.06 | 17.35 ± 2.72 | 14.83 ± 2.35 | 0.394 | 0.821 |  |  |  |  |  |  |  |
|  |  |  |  |  |  |  |  |  |  |  |  |  |  |  |  |  |  |  |  |  |  |  |  |  |  |  |  |  |  |
| Percentage of time exploring the different objects | test | data structure |  | | | F | p-value | power | WT vs Het | WT vs KO | Het vs KO |  |  |  |  | test | data structure |  | | | F | p-value | power | WT vs Het | WT vs KO | Het vs KO |  |  |  |
| - object effect | repeated measures | sphericity assumed |  | | | 1.651 | 0.184 | 0.382 | - | - | - |  |  |  |  | repeated measures | sphericity assumed |  | | | 11.573 | **0.000** | 0.998 | - | - | - |  |  |  |
| - object x genotype effect | repeated measures | sphericity assumed |  |  |  | 0.900 | 0.500 | 0.306 | - | - | - |  |  |  |  | repeated measures | sphericity assumed |  |  |  | 0.444 | 0.847 | 0.160 | - | - | - |  |  |  |
| - genotype effect | repeated measures | sphericity assumed |  |  |  | 0.048 | 0.954 | NA | - | - | - |  |  |  |  | repeated measures | sphericity assumed |  |  |  | 0.000 | 1.000 | 0.050 | - | - | - |  |  |  |
|  |  |  |  |  |  |  |  |  |  |  |  |  |  |  |  |  |  |  |  |  |  |  |  |  |  |  |  |  |  |
| Individual objects | test | data structure | WT | Het | KO | genotype | | | pairwise comparisons | | |  |  |  |  | test | data structure | WT | Het | KO | genotype | | | pairwise comparisons | | |  |  |  |
|  |  |  |  |  |  | F | p-value | power | WT vs Het | WT vs KO | Het vs KO |  |  |  |  |  |  |  |  |  | F | p-value | power | WT vs Het | WT vs KO | Het vs KO |  |  |  |
| Percentage of time exploring the dice | Kruskal-Wallis | non normal | 21.32 ± 2.62 | 21.23 ± 3 | 30.77 ± 6.58 | 1.874 | 0.392 | NA | - | - | - |  |  |  |  | ANOVA | normal | 19.43 ± 2.17 | 16.8 ± 2.77 | 18.2 ± 3.07 | 0.211 | 0.811 | 0.079 | - | - | - |  |  |  |
| Percentage of time exploring the jack | Kruskal-Wallis | non normal | 24.33 ± 4.91 | 28.41 ± 5.09 | 25.87 ± 6.12 | 0.506 | 0.776 | NA | - | - | - |  |  |  |  | ANOVA | normal | 30.29 ± 3.55 | 32.38 ± 4.7 | 30.61 ± 3.12 | 0.084 | 0.920 | 0.061 | - | - | - |  |  |  |
| Percentage of time exploring the Lego | ANOVA | normal | 28.26 ± 3.37 | 32.07 ± 2.51 | 26.89 ± 4.71 | 0.570 | 0.572 | 0.134 | - | - | - |  |  |  |  | ANOVA | normal | 32.47 ± 1.84 | 34 ± 3.97 | 28.95 ± 2.47 | 0.763 | 0.478 | 0.163 | - | - | - |  |  |  |
| Percentage of time exploring the pin | ANOVA | normal | 26.07 ± 4.19 | 18.27 ± 4.07 | 16.45 ± 3.56 | 1.634 | 0.214 | 0.313 | - | - | - |  |  |  |  | Kruskal-Wallis | non normal | 17.79 ± 3.69 | 16.8 ± 3.01 | 22.23 ± 2.94 | 2.343 | 0.310 | NA | - | - | - |  |  |  |
|  |  |  |  |  |  |  |  |  |  |  |  |  |  |  |  |  |  |  |  |  |  |  |  |  |  |  |  |  |  |
| Time exploring the objects, objects ranked by preference | test | data structure |  | | | F | p-value | power | WT vs Het | WT vs KO | Het vs KO |  |  |  |  | test | data structure |  | | | F | p-value | power | WT vs Het | WT vs KO | Het vs KO |  |  |  |
| - object effect | repeated measures | sphericity violated |  | | | 48.443 | **0.000** | 1.000 | - | - | - |  |  |  |  | repeated measures | sphericity violated |  | | | 60.058 | **0.000** | 1.000 | - | - | - |  |  |  |
| - object x genotype effect | repeated measures | sphericity violated |  |  |  | 1.469 | 0.238 | 0.539 | - | - | - |  |  |  |  | repeated measures | sphericity violated |  |  |  | 4.434 | 0.010 | 0.976 | - | - | - |  |  |  |
| - genotype effect | repeated measures | sphericity violated |  |  |  | 4.904 | **0.016** | 0.757 | 0.810 | **0.013** | **0.008** |  |  |  |  | repeated measures | sphericity violated |  |  |  | 3.894 | **0.036** | 0.641 | 0.801 | **0.023** | **0.029** |  |  |  |
|  |  |  |  |  |  |  |  |  |  |  |  |  |  |  |  |  |  |  |  |  |  |  |  |  |  |  |  |  |  |
| Individual objects | test | data structure | WT | Het | KO | genotype | | | pairwise comparisons | | |  |  |  |  | test | data structure | WT | Het | KO | genotype | | | pairwise comparisons | | |  |  |  |
|  |  |  |  |  |  | F | p-value | power | WT vs Het | WT vs KO | Het vs KO |  |  |  |  |  |  |  |  |  | F | p-value | power | WT vs Het | WT vs KO | Het vs KO |  |  |  |
| Time exploring object #1 (sec) | Kruskal-Wallis | non normal | 25.73 ± 3.53 | 28.3 ± 4.28 | 16.39 ± 1.5 | 6.541 | *0.038* | NA | 0.749 | **0.034** | **0.017** |  |  |  |  | Kruskal-Wallis | non normal | 39.02 ± 6.58 | 47.62 ± 5.66 | 24.2 ± 2.19 | 10.906 | **0.004** | NA | 0.355 | **0.036** | **0.001** |  |  |  |
| Time exploring object #2 (sec) | ANOVA | normal | 18.09 ± 2.1 | 18.14 ± 2.19 | 10.04 ± 1.62 | 4.599 | **0.020** | 0.728 | 1.000 | **0.031** | **0.034** |  |  |  |  | Kruskal-Wallis | non normal | 33.09 ± 6.6 | 28.31 ± 2.92 | 19.85 ± 2.08 | 5.388 | *0.068* | NA | - | - | - |  |  |  |
| Time exploring object #3 (sec) | ANOVA | normal | 10.68 ± 1.14 | 11.25 ± 1.18 | 8.03 ± 1.39 | 1.750 | 0.194 | 0.333 | - | - | - |  |  |  |  | Kruskal-Wallis | non normal | 22.92 ± 5.85 | 19.77 ± 1.99 | 12.66 ± 1.49 | 4.482 | 0.106 | NA | - | - | - |  |  |  |
| Time exploring object #4 (sec) | ANOVA | normal | 8.46 ± 0.98 | 7.31 ± 0.98 | 4.53 ± 1.01 | 3.782 | 0.036 | 0.637 | 0.680 | 0.030 | 0.163 |  |  |  |  | Kruskal-Wallis | non normal | 17.07 ± 5.68 | 11.59 ± 1.25 | 9.42 ± 1.24 | 1.282 | 0.527 | NA | - | - | - |  |  |  |
|  |  |  |  |  |  |  |  |  |  |  |  |  |  |  |  |  |  |  |  |  |  |  |  |  |  |  |  |  |  |
| Percentage of time exploring the objects, objects ranked by preference | test | data structure |  | | | F | p-value | power | WT vs Het | WT vs KO | Het vs KO |  |  |  |  | test | data structure |  | | | F | p-value | power | WT vs Het | WT vs KO | Het vs KO |  |  |  |
| - object effect | repeated measures | sphericity violated |  | | | 66.388 | **0.000** | 1.000 | - | - | - |  |  |  |  | repeated measures | sphericity violated |  | | | 104.77 | **0.000** | 1.000 | - | - | - |  |  |  |
| - object x genotype effect | repeated measures | sphericity violated |  |  |  | 0.437 | 0.737 | 0.171 | - | - | - |  |  |  |  | repeated measures | sphericity violated |  |  |  | 2.197 | *0.095* | 0.738 | - | - | - |  |  |  |
| - genotype effect | repeated measures | sphericity violated |  |  |  | 0.946 | 0.401 | 0.196 | - | - | - |  |  |  |  | repeated measures | sphericity violated |  |  |  | 0.599 | 0.447 | 0.115 | - | - | - |  |  |  |
|  |  |  |  |  |  |  |  |  |  |  |  |  |  |  |  |  |  |  |  |  |  |  |  |  |  |  |  |  |  |
| Individual objects | test | data structure | WT | Het | KO | genotype | | | pairwise comparisons | | |  |  |  |  | test | data structure | WT | Het | KO | genotype | | | pairwise comparisons | | |  |  |  |
|  |  |  |  |  |  | F | p-value | power | WT vs Het | WT vs KO | Het vs KO |  |  |  |  |  |  |  |  |  | F | p-value | power | WT vs Het | WT vs KO | Het vs KO |  |  |  |
| Percentage of time exploring object #1 | Kruskal-Wallis | non normal | 40.64 ± 3.29 | 42.4 ± 2.03 | 44.44 ± 5.51 | 1.058 | 0.589 | NA | - | - | - |  |  |  |  | Kruskal-Wallis | non normal | 36.44 ± 2.21 | 43.66 ± 2.95 | 37.14 ± 1.32 | 4.015 | 0.134 | NA | - | - | - |  |  |  |
| Percentage of time exploring object #2 | ANOVA | normal | 28.36 ± 1.77 | 28.24 ± 1.77 | 24.8 ± 2.45 | 0.956 | 0.397 | 0.198 | - | - | - |  |  |  |  | ANOVA | normal | 29.95 ± 1.15 | 26.06 ± 1.38 | 30.05 ± 1.02 | 3.673 | **0.042** | 0.614 | 0.092 | 0.998 | *0.060* |  |  |  |
| Percentage of time exploring object #3 | ANOVA | normal | 17.36 ± 1.63 | 18.47 ± 2.01 | 19.68 ± 2.05 | 0.362 | 0.700 | 0.102 | - | - | - |  |  |  |  | ANOVA | normal | 19.7 ± 1.64 | 19.02 ± 2.02 | 18.93 ± 1.2 | 0.057 | 0.945 | 0.058 | - | - | - |  |  |  |
| Percentage of time exploring object #4 | ANOVA | normal | 13.62 ± 1.25 | 11.96 ± 1.46 | 11.06 ± 2.26 | 0.647 | 0.532 | 0.147 | - | - | - |  |  |  |  | ANOVA | normal | 13.89 ± 1.44 | 11.24 ± 1.38 | 13.86 ± 0.81 | 1.607 | 0.223 | 0.303 | - | - | - |  |  |  |
|  |  |  |  |  |  |  |  |  |  |  |  |  |  |  |  |  |  |  |  |  |  |  |  |  |  |  |  |  |  |
| **Repetitive novel object contact task, object preference, number** | | |  |  |  |  |  |  |  |  |  |  |  |  |  |  |  |  |  |  |  |  |  |  |  |  |  |  |  |
|  | Cohort 1 | | | | | | | | | | |  |  |  |  | Cohort 2 | | | | | | | | | | |  |  |  |
| Number of object interactions | test | data structure |  | | | F | p-value | power | WT vs Het | WT vs KO | Het vs KO |  |  |  |  | test | data structure |  | | | F | p-value | power | WT vs Het | WT vs KO | Het vs KO |  |  |  |
| - object effect | repeated measures | sphericity assumed |  | | | 6.371 | **0.004** | 0.860 | - | - | - |  |  |  |  | repeated measures | sphericity assumed |  | | | 4.402 | **0.025** | 0.854 | - | - | - |  |  |  |
| - object x genotype effect | repeated measures | sphericity assumed |  |  |  | 1.282 | 0.291 | 0.353 | - | - | - |  |  |  |  | repeated measures | sphericity assumed |  |  |  | 0.215 | 0.902 | 0.102 | - | - | - |  |  |  |
| - genotype effect | repeated measures | sphericity assumed |  |  |  | 2.635 | *0.091* | 0.477 | **0.048** | 0.968 | *0.073* |  |  |  |  | repeated measures | sphericity assumed |  |  |  | 1.063 | 0.363 | 0.212 | - | - | - |  |  |  |
|  |  |  |  |  |  |  |  |  |  |  |  |  |  |  |  |  |  |  |  |  |  |  |  |  |  |  |  |  |  |
| Individual objects | test | data structure | WT | Het | KO | genotype | | | pairwise comparisons | | |  |  |  |  | test | data structure | WT | Het | KO | genotype | | | pairwise comparisons | | |  |  |  |
|  |  |  |  |  |  | F | p-value | power | WT vs Het | WT vs KO | Het vs KO |  |  |  |  |  |  |  |  |  | F | p-value | power | WT vs Het | WT vs KO | Het vs KO |  |  |  |
| Number of dice exploration | ANOVA | normal | 16.63 ± 1.55 | 18.7 ± 1.78 | 17.87 ± 1.02 | 0.482 | 0.623 | 0.120 | - | - | - |  |  |  |  | ANOVA | normal | 25.71 ± 2.53 | 23.66 ± 1.79 | 23.33 ± 2.87 | 0.253 | 0.779 | 0.085 | - | - | - |  |  |  |
| Number of jack exploration | ANOVA | normal | 12.72 ± 1.35 | 17.2 ± 1.87 | 14.5 ± 1.21 | 2.280 | 0.122 | 0.421 | - | - | - |  |  |  |  | ANOVA | normal | 31 ± 2.3 | 30.33 ± 3.11 | 26.66 ± 2.14 | 0.807 | 0.459 | 0.170 | - | - | - |  |  |  |
| Number of Lego exploration | Kruskal-Wallis | non normal | 19.72 ± 3.01 | 23.3 ± 2.21 | 15.25 ± 2.12 | 5.067 | 0.079 | NA | - | - | - |  |  |  |  | Kruskal-Wallis | non normal | 29.28 ± 3.33 | 29.66 ± 1.97 | 25.77 ± 2.27 | 1.442 | 0.486 | NA | - | - | - |  |  |  |
| Number of pin exploration | ANOVA | normal | 12.72 ± 1.35 | 17.2 ± 1.87 | 14.5 ± 1.21 | 2.280 | 0.122 | 0.421 | - | - | - |  |  |  |  | ANOVA | normal | 31 ± 2.3 | 30.33 ± 3.11 | 26.66 ± 2.14 | 0.807 | 0.459 | 0.170 | - | - | - |  |  |  |
|  |  |  |  |  |  |  |  |  |  |  |  |  |  |  |  |  |  |  |  |  |  |  |  |  |  |  |  |  |  |
| Percentage of number of object interactions | test | data structure |  | | | F | p-value | power | WT vs Het | WT vs KO | Het vs KO |  |  |  |  | test | data structure |  | | | F | p-value | power | WT vs Het | WT vs KO | Het vs KO |  |  |  |
| - object effect | repeated measures | sphericity assumed |  | | | 6.283 | **0.006** | 0.842 | - | - | - |  |  |  |  | repeated measures | sphericity assumed |  | | | 6.968 | **0.012** | 0.746 | - | - | - |  |  |  |
| - object x genotype effect | repeated measures | sphericity assumed |  |  |  | 1.264 | 0.299 | 0.339 | - | - | - |  |  |  |  | repeated measures | sphericity assumed |  |  |  | 0.294 | 0.770 | 0.093 | - | - | - |  |  |  |
| - genotype effect | repeated measures | sphericity assumed |  |  |  | 0.935 | 0.405 | 0.194 | - | - | - |  |  |  |  | repeated measures | sphericity assumed |  |  |  | 0.279 | 0.760 | 0.089 | - | - | - |  |  |  |
|  |  |  |  |  |  |  |  |  |  |  |  |  |  |  |  |  |  |  |  |  |  |  |  |  |  |  |  |  |  |
| Individual objects | test | data structure | WT | Het | KO | genotype | | | pairwise comparisons | | |  |  |  |  | test | data structure | WT | Het | KO | genotype | | | pairwise comparisons | | |  |  |  |
|  |  |  |  |  |  | F | p-value | power | WT vs Het | WT vs KO | Het vs KO |  |  |  |  |  |  |  |  |  | F | p-value | power | WT vs Het | WT vs KO | Het vs KO |  |  |  |
| Percentage of dice exploration | Kruskal-Wallis | non normal | 27.39 ± 2.03 | 24.8 ± 1.98 | 29.27 ± 1.87 | 2.315 | 0.314 | NA | - | - | - |  |  |  |  | Kruskal-Wallis | non normal | 21.88 ± 1.09 | 21.27 ± 1.74 | 22.74 ± 2.4 | 0.025 | 0.987 | NA | - | - | - |  |  |  |
| Percentage of jack exploration | ANOVA | normal | 20.79 ± 1.82 | 22.22 ± 1.58 | 23.23 ± 1.09 | 0.563 | 0.576 | 0.133 | - | - | - |  |  |  |  | ANOVA | normal | 26.69 ± 1.08 | 26.18 ± 1.2 | 25.99 ± 1.32 | 0.079 | 0.925 | 0.060 | - | - | - |  |  |  |
| Percentage of Lego interaction | ANOVA | normal | 31 ± 2.97 | 30.74 ± 2.19 | 24.25 ± 2.67 | 1.825 | 0.181 | 0.346 | - | - | - |  |  |  |  | ANOVA | normal | 24.72 ± 1.46 | 26.35 ± 1.81 | 25.25 ± 1.37 | 0.270 | 0.766 | 0.087 | - | - | - |  |  |  |
| percentage of pin exploration | ANOVA | normal | 20.79 ± 1.82 | 22.22 ± 1.58 | 23.23 ± 1.09 | 0.563 | 0.576 | 0.133 | - | - | - |  |  |  |  | ANOVA | normal | 51.97 ± 17.97 | 44.35 ± 12.46 | 37.63 ± 8.67 | 0.295 | 0.747 | 0.091 | - | - | - |  |  |  |
|  |  |  |  |  |  |  |  |  |  |  |  |  |  |  |  |  |  |  |  |  |  |  |  |  |  |  |  |  |  |
| Number of object interactions, object ranked by preference | test | data structure |  | | | F | p-value | power | WT vs Het | WT vs KO | Het vs KO |  |  |  |  | test | data structure |  | | | F | p-value | power | WT vs Het | WT vs KO | Het vs KO |  |  |  |
| - object effect | repeated measures | sphericity violated |  | | | 55.245 | **0.000** | 1.000 | - | - | - |  |  |  |  | repeated measures | sphericity violated |  | | | 27.266 | **0.000** | 1.000 | - | - | - |  |  |  |
| - object x genotype effect | repeated measures | sphericity violated |  |  |  | 1.078 | 0.374 | 0.401 | - | - | - |  |  |  |  | repeated measures | sphericity violated |  |  |  | 0.564 | 0.687 | 0.211 | - | - | - |  |  |  |
| - genotype effect | repeated measures | sphericity violated |  |  |  | 2.635 | *0.091* | 0.477 | **0.048** | 0.968 | *0.073* |  |  |  |  | repeated measures | sphericity violated |  |  |  | 1.180 | 0.326 | 0.231 | - | - | - |  |  |  |
|  |  |  |  |  |  |  |  |  |  |  |  |  |  |  |  |  |  |  |  |  |  |  |  |  |  |  |  |  |  |
| Individual objects | test | data structure | WT | Het | KO | genotype | | | pairwise comparisons | | |  |  |  |  | test | data structure | WT | Het | KO | genotype | | | pairwise comparisons | | |  |  |  |
|  |  |  |  |  |  | F | p-value | power | WT vs Het | WT vs KO | Het vs KO |  |  |  |  |  |  |  |  |  | F | p-value | power | WT vs Het | WT vs KO | Het vs KO |  |  |  |
| Number of object #1 explorations | Kruskal-Wallis | non normal | 22.09 ± 2.65 | 25 ± 1.81 | 20 ± 1.25 | 3.733 | 0.155 | NA | - | - | - |  |  |  |  | Kruskal-Wallis | non normal | 33.42 ± 2.68 | 33.77 ± 2.43 | 30.55 ± 1.97 | 0.808 | 0.668 | NA | - | - | - |  |  |  |
| Number of object #2 explorations | ANOVA | normal | 17 ± 1.07 | 20.2 ± 1.31 | 15.75 ± 1.12 | 3.616 | **0.041** | 0.616 | 0.135 | 0.748 | **0.044** |  |  |  |  | ANOVA | normal | 31.85 ± 2.28 | 30.66 ± 3.01 | 26.88 ± 2.17 | 1.015 | 0.379 | 0.204 | - | - | - |  |  |  |
| Number of object #3 explorations | ANOVA | normal | 11.72 ± 1.17 | 16.5 ± 1.89 | 14 ± 1.1 | 2.848 | *0.076* | 0.510 | *0.062* | 0.542 | 0.492 |  |  |  |  | ANOVA | normal | 27.14 ± 2.27 | 28 ± 1.87 | 23.22 ± 2.24 | 1.514 | 0.242 | 0.287 | - | - | - |  |  |  |
| Number of object #4 explorations | ANOVA | normal | 11 ± 1 | 14.7 ± 1.55 | 12.37 ± 0.96 | 2.483 | 0.103 | 0.454 | - | - | - |  |  |  |  | ANOVA | normal | 24.57 ± 2.3 | 21.55 ± 1.33 | 21 ± 2.2 | 0.879 | 0.429 | 0.182 | - | - | - |  |  |  |
|  |  |  |  |  |  |  |  |  |  |  |  |  |  |  |  |  |  |  |  |  |  |  |  |  |  |  |  |  |  |
| Percentage of number of object interactions, object ranked by preference | test | data structure |  | | | F | p-value | power | WT vs Het | WT vs KO | Het vs KO |  |  |  |  | test | data structure |  | | | F | p-value | power | WT vs Het | WT vs KO | Het vs KO |  |  |  |
| - object effect | repeated measures | sphericity violated |  | | | 62.505 | **0.000** | 1.000 | - | - | - |  |  |  |  | repeated measures | sphericity violated |  | | | 29.730 | **0.000** | 1.000 | - | - | - |  |  |  |
| - object x genotype effect | repeated measures | sphericity violated |  |  |  | 1.246 | 0.304 | 0.462 | - | - | - |  |  |  |  | repeated measures | sphericity violated |  |  |  | 0.519 | 0.747 | 0.196 | - | - | - |  |  |  |
| - genotype effect | repeated measures | sphericity violated |  |  |  | 0.000 | 1.000 | 0.050 | - | - | - |  |  |  |  | repeated measures | sphericity violated |  |  |  | 0.880 | 0.429 | 0.182 | - | - | - |  |  |  |
|  |  |  |  |  |  |  |  |  |  |  |  |  |  |  |  |  |  |  |  |  |  |  |  |  |  |  |  |  |  |
| Individual objects | test | data structure | WT | Het | KO | genotype | | | pairwise comparisons | | |  |  |  |  | test | data structure | WT | Het | KO | genotype | | | pairwise comparisons | | |  |  |  |
|  |  |  |  |  |  | F | p-value | power | WT vs Het | WT vs KO | Het vs KO |  |  |  |  |  |  |  |  |  | F | p-value | power | WT vs Het | WT vs KO | Het vs KO |  |  |  |
| Percentage of object #1 explorations | Kruskal-Wallis | non normal | 35.02 ± 1.95 | 33.17 ± 1.51 | 32.43 ± 1.46 | 1.356 | 0.508 | NA | - | - | - |  |  |  |  | Kruskal-Wallis | non normal | 28.56 ± 0.57 | 29.6 ± 0.94 | 30.09 ± 1.31 | 0.593 | 0.743 | NA | - | - | - |  |  |  |
| Percentage of object #2 explorations | ANOVA | normal | 28.16 ± 1.34 | 26.84 ± 1.03 | 25.32 ± 0.97 | 1.392 | 0.267 | 0.272 | - | - | - |  |  |  |  | ANOVA | normal | 27.37 ± 0.79 | 26.51 ± 1.07 | 26.18 ± 1.29 | 0.277 | 0.761 | 0.088 | - | - | - |  |  |  |
| Percentage of object #3 explorations | ANOVA | normal | 18.93 ± 1.01 | 21.08 ± 1.19 | 22.44 ± 0.78 | 2.815 | *0.078* | 0.505 | 0.305 | *0.072* | 0.657 |  |  |  |  | ANOVA | normal | 23.19 ± 0.74 | 24.52 ± 0.74 | 22.58 ± 1.36 | 0.968 | 0.395 | 0.196 | - | - | - |  |  |  |
| Percentage of object #4 explorations | ANOVA | normal | 17.87 ± 0.95 | 18.89 ± 0.88 | 19.79 ± 0.5 | 1.218 | 0.312 | 0.242 | - | - | - |  |  |  |  | ANOVA | normal | 20.86 ± 0.57 | 19.36 ± 1.41 | 20.33 ± 1.15 | 0.408 | 0.670 | 0.108 | - | - | - |  |  |  |
|  |  |  |  |  |  |  |  |  |  |  |  |  |  |  |  |  |  |  |  |  |  |  |  |  |  |  |  |  |  |
| **Repetitive novel object contact task, pattern of object investigation** | | |  |  |  |  |  |  |  |  |  |  |  |  |  |  |  |  |  |  |  |  |  |  |  |  |  |  |  |
|  | Cohort 1 | | | | | | | | | | |  |  |  |  | Cohort 2 | | | | | | | | | | |  |  |  |
| 3-object sequences | test | data structure | WT | Het | KO | genotype | | | pairwise comparisons | | |  |  |  |  | test | data structure | WT | Het | KO | genotype | | | pairwise comparisons | | |  |  |  |
|  |  |  |  |  |  | F | p-value | power | WT vs Het | WT vs KO | Het vs KO |  |  |  |  |  |  |  |  |  | F | p-value | power | WT vs Het | WT vs KO | Het vs KO |  |  |  |
| Total number of 3-object choices | ANOVA | normal | 60.18 ± 4.16 | 63.3 ± 7.14 | 59.62 ± 4.59 | 0.127 | 0.881 | 0.067 | - | - | - |  |  |  |  | ANOVA | normal | 49.71 ± 4.23 | 42.77 ± 4.39 | 47.33 ± 3.95 | 0.682 | 0.516 | 0.150 | - | - | - |  |  |  |
| # of different 3-object sequences | ANOVA | normal | 26.9 ± 0.75 | 27.4 ± 1.62 | 27.25 ± 1.3 | 0.044 | 0.957 | 0.056 | - | - | - |  |  |  |  | ANOVA | normal | 25.85 ± 0.91 | 23.11 ± 1.59 | 23.88 ± 1.16 | 1.074 | 0.359 | 0.214 | - | - | - |  |  |  |
| # of repetition of top preferred sequence | ANOVA | normal | 5.45 ± 0.56 | 5.4 ± 0.54 | 5.25 ± 0.36 | 0.038 | 0.963 | 0.055 | - | - | - |  |  |  |  | Kruskal-Wallis | non normal | 4 ± 0.37 | 4.11 ± 0.42 | 4.11 ± 0.38 | 0.206 | 0.902 | NA | - | - | - |  |  |  |
| # of repetition of 2nd preferred sequence | ANOVA | normal | 4.72 ± 0.38 | 4.7 ± 0.51 | 4.37 ± 0.32 | 0.189 | 0.829 | 0.076 | - | - | - |  |  |  |  | Kruskal-Wallis | non normal | 3.57 ± 0.2 | 3.55 ± 0.33 | 3.77 ± 0.4 | 0.154 | 0.926 | NA | - | - | - |  |  |  |
| # of repetition of 3rd preferred sequence | ANOVA | normal | 4.09 ± 0.34 | 4.2 ± 0.48 | 4 ± 0.42 | 0.053 | 0.949 | 0.057 | - | - | - |  |  |  |  | Kruskal-Wallis | non normal | 3.42 ± 0.2 | 3.11 ± 0.26 | 3.44 ± 0.29 | 0.857 | 0.651 | NA | - | - | - |  |  |  |
| # of repetition of top 3 preferred sequences | ANOVA | normal | 14.27 ± 1.24 | 14.3 ± 1.52 | 13.62 ± 1.06 | 0.074 | 0.928 | 0.060 | - | - | - |  |  |  |  | ANOVA | normal | 11 ± 0.69 | 10.77 ± 0.96 | 11.33 ± 1.05 | 0.092 | 0.912 | 0.062 | - | - | - |  |  |  |
| % of top preferred sequence choice | ANOVA | normal | 8.91 ± 0.48 | 8.87 ± 0.58 | 8.89 ± 0.33 | 0.002 | 0.998 | 0.050 | - | - | - |  |  |  |  | Kruskal-Wallis | non normal | 8.04 ± 0.29 | 10.01 ± 1.12 | 8.62 ± 0.22 | 4.905 | *0.086* | NA | - | - | - |  |  |  |
| % of top 2 preferred sequence choice | ANOVA | normal | 16.77 ± 0.77 | 16.48 ± 0.99 | 16.29 ± 0.56 | 0.084 | 0.920 | 0.061 | - | - | - |  |  |  |  | Kruskal-Wallis | non normal | 15.36 ± 0.44 | 18.59 ± 1.83 | 16.54 ± 0.39 | 0.409 | *0.086* | NA | - | - | - |  |  |  |
| % of top 3 preferred sequence choice | ANOVA | normal | 23.61 ± 1.04 | 23.3 ± 1.46 | 22.95 ± 0.85 | 0.073 | 0.930 | 0.060 | - | - | - |  |  |  |  | Kruskal-Wallis | non normal | 22.41 ± 0.82 | 26.23 ± 2.56 | 23.88 ± 0.6 | 2.762 | 0.251 | NA | - | - | - |  |  |  |
|  |  |  |  |  |  |  |  |  |  |  |  |  |  |  |  |  |  |  |  |  |  |  |  |  |  |  |  |  |  |
| 4-object sequences | test | data structure | WT | Het | KO | genotype | | | pairwise comparisons | | |  |  |  |  | test | data structure | p-value | Het | KO | genotype | | | pairwise comparisons | | |  |  |  |
|  |  |  |  |  |  | F | p-value | power | WT vs Het | WT vs KO | Het vs KO |  |  |  |  |  |  |  |  |  | F | p-value | power | WT vs Het | WT vs KO | Het vs KO |  |  |  |
| Total number of 4-object choices | ANOVA | normal | 59.36 ± 4.29 | 62.5 ± 7.15 | 58.62 ± 4.49 | 0.135 | 0.875 | 0.069 | - | - | - |  |  |  |  | ANOVA | normal | 49.57 ± 4.4 | 43 ± 4.52 | 47.22 ± 4.03 | 0.574 | 0.572 | 0.133 | - | - | - |  |  |  |
| #of different 4-object sequences | ANOVA | normal | 42 ± 2.17 | 44.7 ± 3.85 | 43.62 ± 2.85 | 0.219 | 0.805 | 0.081 | - | - | - |  |  |  |  | ANOVA | normal | 38.85 ± 2.28 | 34 ± 3.26 | 34.88 ± 2.2 | 0.837 | 0.446 | 0.175 | - | - | - |  |  |  |
| #of repetition of top preferred sequence | Kruskal-Wallis | non normal | 3.27 ± 0.27 | 3.3 ± 0.3 | 3.62 ± 0.26 | 0.887 | 0.643 | NA | - | - | - |  |  |  |  | Kruskal-Wallis | non normal | 2.71 ± 0.28 | 2.88 ± 0.26 | 3.22 ± 0.22 | 2.037 | 0.361 | NA | - | - | - |  |  |  |
| #of repetition of 2nd preferred sequence | Kruskal-Wallis | non normal | 3.09 ± 0.28 | 3.2 ± 0.32 | 2.75 ± 0.25 | 0.979 | 0.613 | NA | - | - | - |  |  |  |  | Kruskal-Wallis | non normal | 2.42 ± 0.2 | 2.44 ± 0.17 | 2.77 ± 0.27 | 0.970 | 0.616 | NA | - | - | - |  |  |  |
| #of repetition of 3rd preferred sequence | Kruskal-Wallis | non normal | 2.63 ± 0.24 | 2.8 ± 0.32 | 2.25 ± 0.16 | 2.012 | 0.366 | NA | - | - | - |  |  |  |  | Kruskal-Wallis | non normal | 2.14 ± 0.14 | 2.11 ± 0.11 | 2.22 ± 0.22 | 0.389 | 0.823 | NA | - | - | - |  |  |  |
| #of repetition of top 3 preferred sequences | ANOVA | normal | 9 ± 0.76 | 9.3 ± 0.89 | 8.62 ± 0.59 | 0.169 | 0.846 | 0.073 | - | - | - |  |  |  |  | Kruskal-Wallis | non normal | 7.28 ± 0.47 | 7.44 ± 0.47 | 8.22 ± 0.64 | 1.296 | 0.523 | NA | - | - | - |  |  |  |
| % of top preferred sequence choice | ANOVA | normal | 5.6 ± 0.37 | 5.62 ± 0.45 | 6.28 ± 0.35 | 0.815 | 0.453 | 0.174 | - | - | - |  |  |  |  | Kruskal-Wallis | non normal | 5.55 ± 0.54 | 7.07 ± 0.82 | 7 ± 0.4 | 6.246 | **0.044** | NA | *0.075* | **0.014** | 0.471 |  |  |  |
| % of top 2 preferred sequence choice | ANOVA | normal | 10.84 ± 0.65 | 11.08 ± 0.94 | 11.09 ± 0.72 | 0.035 | 0.966 | 0.055 | - | - | - |  |  |  |  | Kruskal-Wallis | non normal | 10.47 ± 0.53 | 13.01 ± 1.23 | 12.97 ± 0.61 | 7.092 | **0.029** | NA | *0.070* | **0.008** | 0.378 |  |  |  |
| % of top 3 preferred sequence choice | ANOVA | normal | 15.3 ± 0.84 | 15.71 ± 1.17 | 15.02 ± 0.96 | 0.111 | 0.896 | 0.065 | - | - | - |  |  |  |  | Kruskal-Wallis | non normal | 14.88 ± 0.54 | 18.28 ± 1.68 | 17.68 ± 0.75 | 7.638 | **0.022** | NA | **0.019** | **0.011** | 0.835 |  |  |  |
|  |  |  |  |  |  |  |  |  |  |  |  |  |  |  |  |  |  |  |  |  |  |  |  |  |  |  |  |  |  |
| **Barnes maze initial training - Distance** |  |  |  |  |  |  |  |  |  |  |  |  |  |  |  |  |  |  |  |  |  |  |  |  |  |  |  |  |  |
| Distance 4 days, repeated measures | Cohort 1 | | | | | | | | | | |  |  |  |  | Cohort 2 | | | | | | | | | | |  |  |  |
|  | test | data structure |  | | | F | p-value | power | WT vs Het | WT vs KO | Het vs KO |  |  |  |  | test | data structure |  | | | F | p-value | power | WT vs Het | WT vs KO | Het vs KO |  |  |  |
| - day effect | repeated measures | sphericity assumed |  | | | 9.739 | **0.000** | 0.994 | - | - | - |  |  |  |  | repeated measures | sphericity assumed |  | | | 4.702 | **0.009** | 0.821 | - | - | - |  |  |  |
| - day x genotype effect | repeated measures | sphericity assumed |  |  |  | 2.535 | **0.033** | 0.778 | - | - | - |  |  |  |  | repeated measures | sphericity assumed |  |  |  | 0.703 | 0.621 | 0.231 | - | - | - |  |  |  |
| - genotype effect | repeated measures | sphericity assumed |  |  |  | 2.445 | 0.107 | 0.446 | - | - | - |  |  |  |  | repeated measures | sphericity assumed |  |  |  | 0.953 | 0.402 | 0.193 | - | - | - |  |  |  |
|  |  |  |  |  |  |  |  |  |  |  |  |  |  |  |  |  |  |  |  |  |  |  |  |  |  |  |  |  |  |
| Individual days | test | data structure | WT | Het | KO | genotype | | | pairwise comparisons | | |  |  |  |  | test | data structure | WT | Het | KO | genotype | | | pairwise comparisons | | |  |  |  |
|  |  |  |  |  |  | F | p-value | power | WT vs Het | WT vs KO | Het vs KO |  |  |  |  |  |  |  |  |  | F | p-value | power | WT vs Het | WT vs KO | Het vs KO |  |  |  |
| Day 1 | ANOVA | normal | 557.96 ± 67.38 | 543.1 ± 47.41 | 530.86 ± 91.25 | 0.039 | 0.962 | 0.055 | - | - | - |  |  |  |  | ANOVA | normal | 412.11 ± 54.12 | 421.32 ± 83.83 | 443.91 ± 61.76 | 0.051 | 0.950 | 0.057 | - | - | - |  |  |  |
| Day 2 | ANOVA | normal | 450.51 ± 62 | 512.19 ± 60.61 | 650.27 ± 29.46 | 2.727 | *0.085* | 0.490 | 0.710 | *0.071* | 0.274 |  |  |  |  | ANOVA | normal | 391.61 ± 58.01 | 420.14 ± 50.5 | 376.34 ± 51.87 | 0.186 | 0.831 | 0.075 | - | - | - |  |  |  |
| Day 3 | ANOVA | normal | 325.27 ± 42.98 | 335.36 ± 48.15 | 597.53 ± 38.51 | 9.912 | **0.001** | 0.971 | 0.985 | **0.001** | **0.002** |  |  |  |  | ANOVA | normal | 240.63 ± 24.63 | 345.7 ± 41.59 | 387.92 ± 48.08 | 3.190 | **0.062** | 0.547 | 0.192 | 0.056 | 0.735 |  |  |  |
| Day 4 | ANOVA | normal | 353.27 ± 50.87 | 385.64 ± 49.07 | 440.99 ± 54.45 | 0.658 | 0.527 | 0.148 | - | - | - |  |  |  |  | ANOVA | normal | 244.6 ± 28.63 | 354.44 ± 33.02 | 302.84 ± 56.16 | 1.725 | 0.202 | 0.321 | - | - | - |  |  |  |
|  |  |  |  |  |  |  |  |  |  |  |  |  |  |  |  |  |  |  |  |  |  |  |  |  |  |  |  |  |  |
| **Barnes maze reversal - Distance** |  |  |  |  |  |  |  |  |  |  |  |  |  |  |  |  |  |  |  |  |  |  |  |  |  |  |  |  |  |
| Distance 4 days, repeated measures | Cohort 1 | | | | | | | | | | |  |  |  |  | Cohort 2 | | | | | | | | | | |  |  |  |
|  | test | data structure |  | | | F | p-value | power | WT vs Het | WT vs KO | Het vs KO |  |  |  |  | test | data structure |  | | | F | p-value | power | WT vs Het | WT vs KO | Het vs KO |  |  |  |
| - day effect | repeated measures | sphericity assumed |  | | | 4.838 | **0.007** | 0.837 | - | - | - |  |  |  |  | repeated measures | sphericity assumed |  | | | 28.027 | **0.000** | 1.000 | - | - | - |  |  |  |
| - day x genotype effect | repeated measures | sphericity assumed |  |  |  | 2.987 | **0.018** | 0.822 | - | - | - |  |  |  |  | repeated measures | sphericity assumed |  |  |  | 2.891 | **0.016** | 0.855 | - | - | - |  |  |  |
| - genotype effect | repeated measures | sphericity assumed |  |  |  | 1.314 | 0.287 | 0.257 | - | - | - |  |  |  |  | repeated measures | sphericity assumed |  |  |  | 0.732 | 0.493 | 0.157 | - | - | - |  |  |  |
|  |  |  |  |  |  |  |  |  |  |  |  |  |  |  |  |  |  |  |  |  |  |  |  |  |  |  |  |  |  |
| Individual days | test | data structure | WT | Het | KO | genotype | | | pairwise comparisons | | |  |  |  |  | test | data structure | WT | Het | KO | genotype | | | pairwise comparisons | | |  |  |  |
|  |  |  |  |  |  | F | p-value | power | WT vs Het | WT vs KO | Het vs KO |  |  |  |  |  |  |  |  |  | F | p-value | power | WT vs Het | WT vs KO | Het vs KO |  |  |  |
| Day 1 | Kruskal-Wallis | non normal | 417.66 ± 49.85 | 369.63 ± 45.09 | 608.9 ± 72.22 | 6.717 | **0.035** | NA | 0.605 | **0.044** | **0.012** |  |  |  |  | Kruskal-Wallis | non normal | 426.06 ± 62.18 | 511.91 ± 54.18 | 576.44 ± 39.63 | 3.628 | 0.163 | NA | - | - | - |  |  |  |
| Day 2 | ANOVA | normal | 335.39 ± 44.14 | 378.02 ± 46.9 | 474.56 ± 63.9 | 1.792 | 0.187 | 0.339 | - | - | - |  |  |  |  | ANOVA | normal | 339.05 ± 63.32 | 453.22 ± 43.19 | 317.72 ± 53.77 | 2.042 | 0.155 | 0.373 | - | - | - |  |  |  |
| Day 3 | Kruskal-Wallis | non normal | 380.4 ± 46.23 | 472.87 ± 63.15 | 452.97 ± 63.57 | 1.561 | 0.458 | NA | - | - | - |  |  |  |  | Kruskal-Wallis | non normal | 322.61 ± 59.04 | 363.46 ± 59.79 | 345.88 ± 69.77 | 0.406 | 0.816 | NA | - | - | - |  |  |  |
| Day 4 | ANOVA | normal | 359.88 ± 47.34 | 366.33 ± 45.42 | 349.84 ± 41.4 | 0.028 | 0.973 | 0.054 | - | - | - |  |  |  |  | ANOVA | normal | 176.44 ± 47.66 | 202.34 ± 47.96 | 326.22 ± 65.29 | 2.104 | 0.147 | 0.383 | - | - | - |  |  |  |
|  |  |  |  |  |  |  |  |  |  |  |  |  |  |  |  |  |  |  |  |  |  |  |  |  |  |  |  |  |  |
| **Barnes maze initial training probe** |  |  |  |  |  |  |  |  |  |  |  |  |  |  |  |  |  |  |  |  |  |  |  |  |  |  |  |  |  |
|  | Cohort 1 | | | | | | | | | | | | | |  | Cohort 2 | | | | | | | | | | | | | |
| Probe test by genotypes, quadrant effect | test | data structure |  | | | genotype | | | Quadrant pairwise comparisons | | | | | |  | test | data structure |  | | | genotype | | | Quadrant pairwise comparisons | | | | | |
|  |  |  |  |  |  | F | p-value | power | T vs L | T vs R | T vs O | L vs R | L vs O | R vs O |  |  |  |  |  |  | F | p-value | power | T vs L | T vs R | T vs O | L vs R | L vs O | R vs O |
| - All animals | repeated measures | sphericity violated |  | | | 75.488 | **0.000** | 1.000 | **0.000** | **0.000** | **0.000** | 0.932 | 0.783 | 0.896 |  | repeated measures | sphericity violated |  | | | 352.951 | **0.000** | 1.000 | **0.000** | **0.000** | **0.000** | 0.428 | *0.091* | 0.285 |
| - WT | repeated measures | sphericity assumed |  |  |  | 19.287 | **0.000** | 1.000 | **0.000** | **0.000** | **0.001** | 0.523 | 0.948 | 0.457 |  | repeated measures | sphericity violated |  |  |  | 42.623 | **0.000** | 1.000 | **0.002** | **0.000** | **0.000** | *0.059* | *0.084* | *0.055* |
| - Het | repeated measures | sphericity violated |  |  |  | 22.408 | **0.000** | 0.998 | **0.000** | **0.002** | **0.000** | 0.358 | 0.899 | 0.465 |  | repeated measures | sphericity violated |  |  |  | 195.818 | **0.000** | 1.000 | **0.000** | **0.000** | **0.000** | 0.360 | 0.961 | 0.217 |
| - KO | repeated measures | sphericity assumed |  |  |  | 89.420 | **0.000** | 1.000 | **0.000** | **0.000** | **0.000** | 0.741 | 0.372 | 0.771 |  | repeated measures | sphericity assumed |  |  |  | 624.907 | **0.000** | 1.000 | **0.000** | **0.000** | **0.000** | 0.315 | 0.637 | 0.218 |
|  |  |  |  |  |  |  |  |  |  |  |  |  |  |  |  |  |  |  |  |  |  |  |  |  |  |  |  |  |  |
| Probe test by quadrants | test | data structure | WT | Het | KO | genotype | | | pairwise comparisons | | |  |  |  |  | test | data structure | WT | Het | KO | genotype | | | pairwise comparisons | | |  |  |  |
|  |  |  |  |  |  | F | p-value | power | WT vs Het | WT vs KO | Het vs KO |  |  |  |  |  |  |  |  |  | F | p-value | power | WT vs Het | WT vs KO | Het vs KO |  |  |  |
| Target | ANOVA | normal | 94.51 ± 9.07 | 108.73 ± 11.85 | 136.69 ± 8.67 | 3.841 | **0.035** | 0.642 | 0.564 | **0.027** | 0.191 |  |  |  |  | ANOVA | normal | 127.37 ± 11.65 | 144.51 ± 6.43 | 148.29 ± 3.42 | 2.060 | 0.152 | 0.376 | 0.263 | 0.159 | 0.928 |  |  |  |
| Left | ANOVA | normal | 29.55 ± 5.39 | 21.01 ± 2.76 | 15.72 ± 4.63 | 2.301 | 0.121 | 0.423 | - | - | - |  |  |  |  | ANOVA | normal | 27.03 ± 8.81 | 8.04 ± 2.23 | 7.57 ± 1.65 | 5.087 | 0.016 | 0.760 | 0.027 | 0.028 | 0.997 |  |  |  |
| Right | Kruskal-Wallis | non normal | 23.83 ± 5.17 | 27.7 ± 8.24 | 13.62 ± 3.93 | 1.306 | 0.521 | NA | - | - | - |  |  |  |  | Kruskal-Wallis | non normal | 6.07 ± 2.57 | 12.64 ± 3.98 | 11.1 ± 2.31 | 4.325 | 0.115 | NA | 0.338 | 0.539 | 0.935 |  |  |  |
| Opposite | Kruskal-Wallis | non normal | 30.05 ± 6 | 20.2 ± 5.62 | 12 ± 3.81 | 4.239 | 0.120 | NA | - | - | - |  |  |  |  | Kruskal-Wallis | non normal | 10.07 ± 3.52 | 7.9 ± 2.76 | 6.75 ± 1.92 | 0.368 | 0.832 | NA | 0.847 | 0.695 | 0.951 |  |  |  |
|  |  |  |  |  |  |  |  |  |  |  |  |  |  |  |  |  |  |  |  |  |  |  |  |  |  |  |  |  |  |
| **Barnes maze reversal probe (time in quadrant)** | | |  |  |  |  |  |  |  |  |  |  |  |  |  |  |  |  |  |  |  |  |  |  |  |  |  |  |  |
|  | Cohort 1 | | | | | | | | | | | | | |  | Cohort 2 | | | | | | | | | | | | | |
| Probe test by genotypes, quadrant effect | test | data structure |  | | | genotype | | | Quadrant pairwise comparisons | | | | | |  | test | data structure |  | | | genotype | | | Quadrant pairwise comparisons | | | | | |
|  |  |  |  |  |  | F | p-value | power | T vs L | T vs R | T vs O | L vs R | L vs O | R vs O |  |  |  |  |  |  | F | p-value | power | T vs L | T vs R | T vs O | L vs R | L vs O | R vs O |
| - All animals | repeated measures | sphericity violated |  | | | 15.066 | **0.000** | 1.000 | **0.000** | **0.000** | 0.192 | 0.331 | **0.000** | **0.001** |  | repeated measures | sphericity violated |  | | | 45.909 | **0.000** | 1.000 | **0.000** | **0.000** | **0.000** | 0.483 | **0.001** | **0.002** |
| - WT | repeated measures | sphericity violated |  |  |  | 18.948 | **0.001** | 0.562 | **0.001** | **0.000** | **0.006** | **0.020** | *0.066* | **0.013** |  | repeated measures | sphericity violated |  |  |  | 15.916 | **0.004** | 1.000 | **0.002** | **0.002** | **0.025** | **0.002** | 0.100 | *0.090* |
| - Het | repeated measures | sphericity violated |  |  |  | 3.397 | *0.056* | 0.562 | **0.006** | 0.167 | 0.869 | 0.175 | **0.006** | 0.222 |  | repeated measures | sphericity violated |  |  |  | 130.91 | **0.000** | 1.000 | **0.000** | **0.000** | **0.000** | 0.364 | *0.063* | **0.027** |
| - KO | repeated measures | sphericity violated |  |  |  | 11.904 | **0.008** | 0.885 | **0.009** | *0.056* | 0.120 | 0.298 | **0.002** | **0.002** |  | repeated measures | sphericity violated |  |  |  | 5.354 | **0.032** | 0.645 | **0.006** | *0.052* | 0.466 | 0.131 | **0.008** | *0.088* |
|  |  |  |  |  |  |  |  |  |  |  |  |  |  |  |  |  |  |  |  |  |  |  |  |  |  |  |  |  |  |
|  | test | data structure | WT | Het | KO | genotype | | | pairwise comparisons | | |  |  |  |  | test | data structure | WT | Het | KO | genotype | | | pairwise comparisons | | |  |  |  |
| Probe test by quadrants |  |  |  |  |  | F | p-value | power | WT vs Het | WT vs KO | Het vs KO |  |  |  |  |  |  |  |  |  | F | p-value | power | WT vs Het | WT vs KO | Het vs KO |  |  |  |
| Target | Kruskal-Wallis | non normal | 116.1 ± 15.95 | 69.56 ± 15.76 | 50.04 ± 11.12 | 6.620 | **0.037** | NA | *0.090* | **0.013** | 0.353 |  |  |  |  | Kruskal-Wallis | non normal | 114.12 ± 16.12 | 145.13 ± 7.88 | 83.37 ± 18.51 | 6.908 | **0.032** | NA | 0.183 | 0.244 | **0.009** |  |  |  |
| Left | Kruskal-Wallis | non normal | 19.29 ± 5.74 | 10.91 ± 3.33 | 13.4 ± 3.67 | 1.039 | 0.595 | NA | - | - | - |  |  |  |  | Kruskal-Wallis | non normal | 17.11 ± 4.03 | 4.84 ± 2.4 | 6.43 ± 3.98 | 5.153 | *0.076* | NA | - | - | - |  |  |  |
| Right | Kruskal-Wallis | non normal | 9.96 ± 2.98 | 32.9 ± 13.48 | 20.86 ± 4.28 | 3.988 | 0.135 | NA | - | - | - |  |  |  |  | Kruskal-Wallis | non normal | 9.56 ± 4.57 | 2.46 ± 1.17 | 25.19 ± 9.56 | 5.960 | *0.051* | NA | - | - | - |  |  |  |
| Opposite | ANOVA | normal | 32.38 ± 8.85 | 64.73 ± 15.34 | 93.75 ± 13.37 | 5.549 | **0.010** | 0.808 | 0.155 | **0.008** | 0.297 |  |  |  |  | ANOVA | normal | 33.11 ± 11.78 | 18.47 ± 6.38 | 58.87 ± 14.5 | 3.579 | **0.046** | 0.599 | 0.629 | 0.272 | **0.038** |  |  |  |
