# Supplementary material for: Behavioral Phenotyping of an Improved Mouse Model of Phelan–McDermid Syndrome with a Complete Deletion of the Shank3 Gene
Source: eNeuro. 2018 Oct 5;5(3):ENEURO.0046-18.2018. doi: 10.1523/ENEURO.0046-18.2018 (PMC6175061; doi:10.1523/ENEURO.0046-18.2018)
Supplement: Extended Data Table 12-1 — Individual results and statistical analyses for cohorts 1 and 2 related to learning and memory. WT, wild-type mice; Het, heterozygous mice; KO, homozygous knockout mice. Group values are reported as means ± s.e.m. Red font indicates significant results (p < 0.05), orange font indicates trends (0.1 < p < 0.05). Download Table 12-1, DOCX file. [file sup_enu-eN-CFN-0046-18-s09.docx]

# Extended Tables

Extended Table 12-1

| **Y-maze, spontaneous alternation behavior** |  |  |  |  |  |  |  |  |  |  |  |  |  |  |  |  |  |  |  |  |  |  |  |
| --- | --- | --- | --- | --- | --- | --- | --- | --- | --- | --- | --- | --- | --- | --- | --- | --- | --- | --- | --- | --- | --- | --- | --- |
|  | Cohort 1 | | | | | | | | | | |  | Cohort 2 | | | | | | | | | | |
|  | test | data structure | WT | Het | KO | genotype | | | pairwise comparisons | | |  | test | data structure | WT | Het | KO | genotype | | | pairwise comparisons | | |
|  |  |  |  |  |  | F | p-value | power | WT vs Het | WT vs KO | Het vs KO |  |  |  |  |  |  | F | p-value | power | WT vs Het | WT vs KO | Het vs KO |
| Percentage of arm 1 choices | ANOVA | normal | 31.82 ± 1.28 | 34.51 ± 1.39 | 33.48 ± 1.37 | 1.067 | 0.358 | 0.217 | - | - | - |  | ANOVA | normal | 33.06 ± 1.15 | 33.94 ± 1.28 | 32.14 ± 1.91 | 0.356 | 0.704 | 0.100 | - | - | - |
| Percentage of arm 2 choices | Kruskal-Wallis | non normal | 33.43 ± 1.51 | 31.11 ± 1.16 | 32.54 ± 1.53 | 1.278 | 0.528 | NA | - | - | - |  | ANOVA | normal | 37.58 ± 1.56 | 34.54 ± 1.81 | 37.55 ± 1.97 | 0.905 | 0.418 | 0.188 | - | - | - |
| Percentage of arm 3 choices | ANOVA | normal | 34.74 ± 1.86 | 34.37 ± 1.64 | 33.97 ± 1.05 | 0.057 | 0.944 | 0.058 | - | - | - |  | ANOVA | normal | 28.68 ± 1.83 | 31.45 ± 1.33 | 30.3 ± 1.53 | 0.738 | 0.489 | 0.160 | - | - | - |
|  |  |  |  |  |  |  |  |  |  |  |  |  |  |  |  |  |  |  |  |  |  |  |  |
| Arm preference, t-test comparison to chance level | test | data structure |  | | | All t | All p-value | power |  |  |  |  | test | data structure |  | | | All t | All p-value | power |  |  |  |
| Arm 1, All mice | One sample T-test | normal |  | | | -0.144 | 0.887 | NA |  |  |  |  | One sample T-test | normal |  | | | -0.358 | 0.723 | NA |  |  |  |
| Arm 1, WT | One sample T-test | normal |  |  |  | -1.172 | 0.268 | NA |  |  |  |  | One sample T-test | normal |  |  |  | -0.233 | 0.822 | NA |  |  |  |
| Arm 1, Het | One sample T-test | normal |  |  |  | 0.850 | 0.417 | NA |  |  |  |  | One sample T-test | normal |  |  |  | 0.483 | 0.642 | NA |  |  |  |
| Arm 1, KO | One sample T-test | normal |  |  |  | 0.113 | 0.913 | NA |  |  |  |  | One sample T-test | normal |  |  |  | -0.621 | 0.550 | NA |  |  |  |
| Arm 2, All mice | One sample Wilcoxon | non normal |  | | | -1.450 | 0.147 | NA |  |  |  |  | One sample T-test | normal |  | | | 3.067 | **0.005** | NA |  |  |  |
| Arm 2, WT | One sample Wilcoxon | non normal |  |  |  | 0.067 | 0.948 | NA |  |  |  |  | One sample T-test | normal |  |  |  | 2.720 | **0.030** | NA |  |  |  |
| Arm 2, Het | One sample Wilcoxon | non normal |  |  |  | -1.894 | *0.091* | NA |  |  |  |  | One sample T-test | normal |  |  |  | 0.673 | 0.520 | NA |  |  |  |
| Arm 2, KO | One sample Wilcoxon | non normal |  |  |  | -0.511 | 0.623 | NA |  |  |  |  | One sample T-test | normal |  |  |  | 2.138 | *0.061* | NA |  |  |  |
| Arm 3, All mice | One sample T-test | normal |  | | | 1.174 | 0.250 | NA |  |  |  |  | One sample T-test | normal |  | | | -3.492 | **0.002** | NA |  |  |  |
| Arm 3, WT | One sample T-test | normal |  |  |  | 0.762 | 0.464 | NA |  |  |  |  | One sample T-test | normal |  |  |  | -2.538 | **0.039** | NA |  |  |  |
| Arm 3, Het | One sample T-test | normal |  |  |  | 0.633 | 0.542 | NA |  |  |  |  | One sample T-test | normal |  |  |  | -1.407 | 0.197 | NA |  |  |  |
| Arm 3, KO | One sample T-test | normal |  |  |  | 0.606 | 0.562 | NA |  |  |  |  | One sample T-test | normal |  |  |  | -1.967 | *0.081* | NA |  |  |  |
|  |  |  |  |  |  |  |  |  |  |  |  |  |  |  |  |  |  |  |  |  |  |  |  |
|  | test | data structure | WT | Het | KO | genotype | | | pairwise comparisons | | |  | test | data structure | WT | Het | KO | genotype | | | pairwise comparisons | | |
|  |  |  |  |  |  | F | p-value | power | WT vs Het | WT vs KO | Het vs KO |  |  |  |  |  |  | F | p-value | power | WT vs Het | WT vs KO | Het vs KO |
| 0-15 min, total number of choices | ANOVA | normal | 46.09 ± 3.45 | 35.3 ± 2.99 | 38.55 ± 4.23 | 2.553 | *0.097* | 0.466 | *0.090* | 0.311 | 0.805 |  | ANOVA | normal | 39.75 ± 6.14 | 45.77 ± 3.51 | 38.4 ± 3.79 | 0.792 | 0.465 | 0.169 | - | - | - |
| 0-15 min, number of correct choice | ANOVA | normal | 59.5 ± 2.22 | 63.38 ± 2.19 | 57.33 ± 2.48 | 1.709 | 0.200 | 0.327 | - | - | - |  | ANOVA | normal | 54.66 ± 1.97 | 57.68 ± 3.09 | 57.68 ± 2.22 | 0.456 | 0.639 | 0.116 | - | - | - |
| 0-15 min, number of type 1 errors | ANOVA | normal | 36.69 ± 1.93 | 30.84 ± 1.38 | 41.77 ± 2.79 | 6.686 | **0.004** | 0.881 | 0.119 | 0.212 | **0.003** |  | Kruskal-Wallis | non normal | 39.33 ± 2.15 | 37.7 ± 2.57 | 35.15 ± 1.92 | 2.903 | 0.234 | NA | - | - | - |
| 0-15 min, number of type 2 errors | Kruskal-Wallis | non normal | 2.62 ± 0.79 | 6.26 ± 1.74 | 1.58 ± 0.72 | 5.886 | **0.053** | NA | - | - | - |  | ANOVA | normal | 5.99 ± 2.32 | 4.6 ± 0.97 | 7.15 ± 1.52 | 0.640 | 0.536 | 0.145 | - | - | - |
|  |  |  |  |  |  |  |  |  |  |  |  |  |  |  |  |  |  |  |  |  |  |  |  |
|  |  |  |  |  |  |  |  |  |  |  |  |  |  |  |  |  |  |  |  |  |  |  |  |
| **Fear conditioning** |  |  |  |  |  |  |  |  |  |  |  |  |  |  |  |  |  |  |  |  |  |  |  |
|  | Cohort 1 | | | | | | | | | | |  | Cohort 2 | | | | | | | | | | |
| Training, repeated measures | test | data structure |  | | | F | p-value | power | WT vs Het | WT vs KO | Het vs KO |  | test | data structure |  | | | F | p-value | power | WT vs Het | WT vs KO | Het vs KO |
| - time effect | repeated measures | sphericity violated |  | | | 45.872 | **0.000** | 1.000 | - | - | - |  | repeated measures | sphericity violated |  | | | 7.804 | **0.000** | 0.998 | - | - | - |
| - time x genotype effect | repeated measures | sphericity violated |  |  |  | 45.872 | **0.000** | 0.869 | - | - | - |  | repeated measures | sphericity violated |  |  |  | 1.221 | 0.292 | 0.562 | - | - | - |
| - genotype effect | repeated measures | sphericity violated |  |  |  | 11.218 | **0.000** | 0.985 | 0.796 | 0.000 | 0.002 |  | repeated measures | sphericity violated |  |  |  | 4.376 | **0.024** | 0.700 | 0.915 | **0.034** | 0.069 |
|  |  |  |  |  |  |  |  |  |  |  |  |  |  |  |  |  |  |  |  |  |  |  |  |
|  | Cohort 1 | | | | | | | | | | |  | Cohort 2 | | | | | | | | | | |
| Training, individual time bins | test | data structure | WT | Het | KO | genotype | | | pairwise comparisons | | |  | test | data structure | WT | Het | KO | genotype | | | pairwise comparisons | | |
|  |  |  |  |  |  | F | p-value | power | WT vs Het | WT vs KO | Het vs KO |  |  |  |  |  |  | F | p-value | power | WT vs Het | WT vs KO | Het vs KO |
| Training habituation | Kruskal-Wallis | non normal | 17.96 ± 4.72 | 10.55 ± 3.49 | 20.97 ± 5.75 | 3.608 | 0.165 | NA | - | - | - |  | Kruskal-Wallis | non normal | 15.34 ± 2.85 | 10.33 ± 3.37 | 22.17 ± 8.96 | 1.101 | 0.577 | NA | - | - | - |
| Training Pre-tone 0-120 | Kruskal-Wallis | non normal | 10.79 ± 2.51 | 8.91 ± 2.51 | 22.58 ± 4.96 | 5.731 | *0.057* | NA | 0.913 | *0.051* | **0.024** |  | Kruskal-Wallis | non normal | 9.98 ± 2.38 | 7.31 ± 1.32 | 19.59 ± 6.31 | 3.821 | 0.148 | NA | - | - | - |
| Training Tone/shock120-140 | Kruskal-Wallis | non normal | 9.08 ± 3.93 | 8.85 ± 4.59 | 20.76 ± 5.17 | 5.357 | *0.069* | NA | - | - | - |  | Kruskal-Wallis | non normal | 12.65 ± 5.78 | 4.63 ± 3.21 | 19.21 ± 10.87 | 0.836 | 0.658 | NA | - | - | - |
| Training Post-tone140-260 | Kruskal-Wallis | non normal | 22.7 ± 4.99 | 20.02 ± 6.33 | 62.76 ± 7.91 | 12.505 | **0.002** | NA | 0.582 | **0.004** | **0.001** |  | Kruskal-Wallis | non normal | 15.67 ± 5.67 | 17.51 ± 4.52 | 34.1 ± 8.83 | 2.577 | 0.276 | NA | - | - | - |
| Training Tone/shock 260-280 | Kruskal-Wallis | non normal | 14.95 ± 5.9 | 20.52 ± 8.33 | 53.7 ± 11.2 | 6.394 | **0.041** | NA | 0.872 | **0.021** | **0.035** |  | Kruskal-Wallis | non normal | 15.24 ± 8.17 | 29.1 ± 6.15 | 41.42 ± 10.2 | 5..382 | 0.068 | NA | - | - | - |
| Training Tone/shock 260-280 | Kruskal-Wallis | non normal | 38.75 ± 7.07 | 50.3 ± 10.27 | 84.04 ± 6.09 | 12.469 | **0.002** | NA | 0.373 | **0.001** | **0.012** |  | Kruskal-Wallis | non normal | 20.48 ± 5.96 | 24.08 ± 5.46 | 48.3 ± 8.66 | 4.714 | **0.019** | NA | 0.645 | **0.015** | *0.042* |
| Training Tone/shock 400-420 | Kruskal-Wallis | non normal | 40.11 ± 8.9 | 52.46 ± 10.99 | 79.68 ± 5.54 | 9.976 | **0.007** | NA | 0.423 | **0.002** | **0.024** |  | ANOVA | normal | 18.55 ± 3.96 | 27.39 ± 9.11 | 52.18 ± 8.34 | 5.119 | **0.014** | 0.771 | 0.720 | **0.015** | *0.074* |
| Training Post-tone 420-540 | Kruskal-Wallis | non normal | 52.22 ± 7.89 | 66.4 ± 9.83 | 85.1 ± 4.6 | 9.859 | **0.007** | NA | 0.146 | **0.002** | *0.091* |  | ANOVA | normal | 15.38 ± 5.09 | 26.74 ± 5.76 | 40.71 ± 7.36 | 3.977 | **0.032** | 0.656 | 0.451 | **0.026** | 0.267 |
|  |  |  |  |  |  |  |  |  |  |  |  |  |  |  |  |  |  |  |  |  |  |  |  |
| Context, repeated measures | test | data structure |  | | | F | p-value | power | WT vs Het | WT vs KO | Het vs KO |  | test | data structure |  | | | F | p-value | power | WT vs Het | WT vs KO | Het vs KO |
| - time effect | repeated measures | sphericity assumed |  | | | 2.655 | *0.054* | 0.628 | - | - | - |  | repeated measures | sphericity assumed |  | | | 2.055 | 0.114 | 0.506 | - | - | - |
| - time x genotype effect | repeated measures | sphericity assumed |  |  |  | 0.712 | 0.641 | 0.267 | - | - | - |  | repeated measures | sphericity assumed |  |  |  | 0.904 | 0.497 | 0.335 | - | - | - |
| - genotype effect | repeated measures | sphericity assumed |  |  |  | 0.450 | 0.642 | 0.116 | - | - | - |  | repeated measures | sphericity assumed |  |  |  | 1.951 | 0.164 | 0.364 | - | - | - |
|  |  |  |  |  |  |  |  |  |  |  |  |  |  |  |  |  |  |  |  |  |  |  |  |
| Context, individual time bins | test | data structure | WT | Het | KO | genotype | | | pairwise comparisons | | |  | test | data structure | WT | Het | KO | genotype | | | pairwise comparisons | | |
|  |  |  |  |  |  | F | p-value | power | WT vs Het | WT vs KO | Het vs KO |  |  |  |  |  |  | F | p-value | power | WT vs Het | WT vs KO | Het vs KO |
| Context 0-60 | ANOVA | normal | 62.94 ± 5.29 | 57.47 ± 5.02 | 52.21 ± 6.69 | 0.900 | 0.418 | 0.189 | - | - | - |  | Kruskal-Wallis | non normal | 63.29 ± 10.3 | 56.88 ± 10.92 | 37.18 ± 9.96 | 4.270 | 0.118 | NA | - | - | - |
| Context 60-120 | Kruskal-Wallis | non normal | 69.66 ± 6.57 | 59.01 ± 9.45 | 73.65 ± 10.48 | 1.959 | 0.375 | NA | - | - | - |  | Kruskal-Wallis | non normal | 61.78 ± 13.97 | 73.75 ± 10.15 | 46.96 ± 9.11 | 4.180 | 0.124 | NA | - | - | - |
| Context 120-180 | ANOVA | normal | 67.22 ± 8.34 | 56.96 ± 9.83 | 51.1 ± 11.37 | 0.709 | 0.501 | 0.157 | - | - | - |  | Kruskal-Wallis | non normal | 56.36 ± 12.86 | 67.83 ± 8.4 | 35.35 ± 10.8 | 3.787 | 0.151 | NA | - | - | - |
| Context 180-240 | ANOVA | normal | 56.12 ± 8.68 | 54.02 ± 12.25 | 47.29 ± 8.98 | 0.200 | 0.820 | 0.078 | - | - | - |  | Kruskal-Wallis | non normal | 56.13 ± 10.71 | 54.93 ± 9.23 | 39.77 ± 9.11 | 2.274 | 0.321 | NA | - | - | - |
| Context mean | ANOVA | normal | 63.99 ± 5.68 | 56.87 ± 7.15 | 56.07 ± 7.13 | 0.450 | 0.642 | 0.116 | - | - | - |  | Kruskal-Wallis | non normal | 59.39 ± 11.17 | 63.35 ± 8.2 | 39.81 ± 8.66 | 3.885 | 0.145 | NA | - | - | - |
|  |  |  |  |  |  |  |  |  |  |  |  |  |  |  |  |  |  |  |  |  |  |  |  |
| Cued, repeated measures | test | data structure |  | | | F | p-value | power | WT vs Het | WT vs KO | Het vs KO |  | test | data structure |  | | | F | p-value | power | WT vs Het | WT vs KO | Het vs KO |
| - time effect | repeated measures | sphericity violated |  | | | 29.784 | **0.000** | 1.000 | - | - | - |  | repeated measures | sphericity violated |  | | | 2.688 | *0.054* | 0.626 | - | - | - |
| - time x genotype effect | repeated measures | sphericity violated |  |  |  | 5.525 | **0.000** | 0.999 | - | - | - |  | repeated measures | sphericity violated |  |  |  | 1.011 | 0.424 | 0.621 | - | - | - |
| - genotype effect | repeated measures | sphericity violated |  |  |  | 10.587 | **0.000** | 0.980 | 0.094 | **0.000** | **0.055** |  | repeated measures | sphericity violated |  |  |  | 0.908 | 0.417 | 0.188 | - | - | - |
|  |  |  |  |  |  |  |  |  |  |  |  |  |  |  |  |  |  |  |  |  |  |  |  |
| Cued, individual time bins | test | data structure | WT | Het | KO | genotype | | | pairwise comparisons | | |  | test | data structure | WT | Het | KO | genotype | | | pairwise comparisons | | |
|  |  |  |  |  |  | F | p-value | power | WT vs Het | WT vs KO | Het vs KO |  |  |  |  |  |  | F | p-value | power | WT vs Het | WT vs KO | Het vs KO |
| Cued Pre-tone 0-60 | Kruskal-Wallis | non normal | 0 ± 0 | 0 ± 0 | 0.41 ± 0.41 | 2.333 | 0.311 | NA | - | - | - |  | Kruskal-Wallis | non normal | 3.91 ± 2.59 | 0.85 ± 0.57 | 8.94 ± 4.64 | 0.914 | 0.633 | NA | - | - | - |
| Cued Pre-tone 60-120 | Kruskal-Wallis | non normal | 0 ± 0 | 0 ± 0 | 0.37 ± 0.37 | 2.333 | 0.311 | NA | - | - | - |  | Kruskal-Wallis | non normal | 1.99 ± 0.75 | 4.13 ± 1.64 | 4.18 ± 1.44 | 1.237 | 0.539 | NA | - | - | - |
| Cued Tone 120-140 | Kruskal-Wallis | non normal | 12.11 ± 6.96 | 20.67 ± 7.59 | 38.2 ± 7.09 | 6.111 | **0.047** | NA | 0.316 | **0.014** | 0.144 |  | Kruskal-Wallis | non normal | 9.34 ± 4.57 | 2.66 ± 2.02 | 13.56 ± 9.09 | 1.166 | 0.558 | NA | - | - | - |
| Cued Post-tone 140-200 | Kruskal-Wallis | non normal | 9.82 ± 3.03 | 8.71 ± 2.13 | 16.32 ± 5.57 | 1.517 | 0.468 | NA | - | - | - |  | Kruskal-Wallis | non normal | 6.73 ± 2.89 | 5.97 ± 2.91 | 10.18 ± 5.13 | 0.240 | 0.887 | NA | - | - | - |
| Cued Post-tone 200-260 | Kruskal-Wallis | non normal | 1.96 ± 1.33 | 6.42 ± 2.02 | 3.82 ± 1.37 | 3.410 | 0.182 | NA | - | - | - |  | Kruskal-Wallis | non normal | 2.75 ± 1.72 | 6.79 ± 2.47 | 15.62 ± 8.87 | 3.587 | 0.166 | NA | - | - | - |
| Cued Tone 260-280 | Kruskal-Wallis | non normal | 10.57 ± 5.5 | 32.74 ± 9 | 65.37 ± 4.16 | 15.451 | **0.000** | NA | *0.063* | **0.000** | **0.038** |  | Kruskal-Wallis | non normal | 18.41 ± 9.87 | 4.5 ± 3.59 | 15.41 ± 9.12 | 0.361 | 0.361 | NA | - | - | - |
| Cued Post-tone 280-340 | Kruskal-Wallis | non normal | 9.29 ± 3.54 | 28.67 ± 8.28 | 35.91 ± 6.82 | 7.307 | **0.026** | NA | 0.057 | **0.010** | 0.474 |  | Kruskal-Wallis | non normal | 8.43 ± 4.5 | 9.54 ± 3.73 | 18.4 ± 9.17 | 0.766 | 0.682 | NA | - | - | - |
| Cued Post-tone 340-400 | Kruskal-Wallis | non normal | 3.78 ± 1.39 | 13.94 ± 4.85 | 25.74 ± 7.06 | 7.223 | **0.027** | NA | 0.167 | **0.007** | 0.190 |  | Kruskal-Wallis | non normal | 6.9 ± 2.53 | 5.54 ± 1.63 | 16.71 ± 6.61 | 0.520 | 0.420 | NA | - | - | - |
