# Supplementary material for: Behavioral Phenotyping of an Improved Mouse Model of Phelan–McDermid Syndrome with a Complete Deletion of the Shank3 Gene
Source: eNeuro. 2018 Oct 5;5(3):ENEURO.0046-18.2018. doi: 10.1523/ENEURO.0046-18.2018 (PMC6175061; doi:10.1523/ENEURO.0046-18.2018)
Supplement: Extended Data Table 13-1 — Individual results and statistical analyses for cohorts 1 and 2 related to anxiety-like behaviors. WT, wild-type mice; Het, heterozygous mice; KO, homozygous knockout mice. Group values are reported as means ± s.e.m. Red font indicates significant results (p < 0.05), orange font indicates trends (0.1 < p < 0.05). Download Table 13-1, DOCX file. [file sup_enu-eN-CFN-0046-18-s10.docx]

# Extended Tables

Extended Table 13-1

| **Open field thigmotaxis** |  |  |  |  |  |  |  |  |  |  |  |  |  |  |  |  |  |  |  |  |  |  |  |
| --- | --- | --- | --- | --- | --- | --- | --- | --- | --- | --- | --- | --- | --- | --- | --- | --- | --- | --- | --- | --- | --- | --- | --- |
|  | Cohort 1 | | | | | | | | | | |  | Cohort 2 | | | | | | | | | | |
|  | test | data structure | WT | Het | KO | genotype | | | pairwise comparisons | | |  | test | data structure | WT | Het | KO | genotype | | | pairwise comparisons | | |
|  |  |  |  |  |  | F | p-value | power | WT vs Het | WT vs KO | Het vs KO |  |  |  |  |  |  | F | p-value | power | WT vs Het | WT vs KO | Het vs KO |
| Distance in border (cm) | ANOVA | normal | 11144.32 ± 576.73 | 9820.44 ± 608.93 | 8870.49 ± 572.36 | 3.780 | **0.036** | 0.639 | 0.252 | **0.030** | 0.516 |  | Kruskal-Wallis | non normal | 9630.85 ± 1029.53 | 7768 ± 828.74 | 7106.68 ± 408.73 | 5.343 | *0.069* | NA | - | - | - |
|  |  |  |  |  |  |  |  |  |  |  |  |  |  |  |  |  |  |  |  |  |  |  |  |
| Distance, repeated measures | test | data structure |  | | | F | p-value | power | WT vs Het | WT vs KO | Het vs KO |  | test | data structure |  | | | F | p-value | power | WT vs Het | WT vs KO | Het vs KO |
| - time effect | repeated measures | sphericity assumed |  |  |  | 36.204 | **0.000** | 1.000 | - | - | - |  | repeated measures | sphericity assumed |  |  |  | 19.444 | **0.000** | 1.000 |  |  |  |
| - time x genotype effect | repeated measures | sphericity assumed |  |  |  | 1.944 | **0.044** | 0.852 | - | - | - |  | repeated measures | sphericity assumed |  |  |  | 1.535 | 0.135 | 0.733 | - | - | - |
| - genotype effect | repeated measures | sphericity assumed |  |  |  | 3.780 | **0.036** | 0.639 | 0.252 | **0.030** | 0.516 |  | repeated measures | sphericity assumed |  |  |  | 2.867 | *0.076* | 0.508 | 0.230 | *0.069* | 0.803 |
|  |  |  |  |  |  |  |  |  |  |  |  |  |  |  |  |  |  |  |  |  |  |  |  |
|  | test | data structure | WT | Het | KO | genotype | | | pairwise comparisons | | |  | test | data structure | WT | Het | KO | genotype | | | pairwise comparisons | | |
|  |  |  |  |  |  | F | p-value | power | WT vs Het | WT vs KO | Het vs KO |  |  |  |  |  |  | F | p-value | power | WT vs Het | WT vs KO | Het vs KO |
| Distance in center (cm) | ANOVA | normal | 3642.07 ± 385.94 | 2890.37 ± 403.9 | 2719.81 ± 421.01 | 1.530 | 0.235 | 0.296 | - | - | - |  | Kruskal-Wallis | non normal | 2774.04 ± 577.24 | 1835.21 ± 356.24 | 1617.59 ± 161.2 | 2.303 | 0.316 | NA | - | - | - |
|  |  |  |  |  |  |  |  |  |  |  |  |  |  |  |  |  |  |  |  |  |  |  |  |
| Distance in center, repeated measures | test | data structure |  | | | F | p-value | power | WT vs Het | WT vs KO | Het vs KO |  | test | data structure |  | | | F | p-value | power | WT vs Het | WT vs KO | Het vs KO |
| - time effect | repeated measures | sphericity assumed |  |  |  | 0.777 | 0.568 | 0.273 | - | - | - |  | repeated measures | sphericity assumed |  |  |  | 2.367 | **0.044** | 0.739 | - | - | - |
| - time x genotype effect | repeated measures | sphericity assumed |  |  |  | 0.651 | 0.768 | 0.328 | - | - | - |  | repeated measures | sphericity assumed |  |  |  | 1.808 | 0.066 | 0.816 | - | - | - |
| - genotype effect | repeated measures | sphericity assumed |  |  |  | 1.530 | 0.235 | 0.296 | - | - | - |  | repeated measures | sphericity assumed |  |  |  | 2.577 | *0.097* | 0.464 | 0.217 | *0.096* | 0.907 |
|  |  |  |  |  |  |  |  |  |  |  |  |  |  |  |  |  |  |  |  |  |  |  |  |
|  | test | data structure | WT | Het | KO | genotype | | | pairwise comparisons | | |  | test | data structure | WT | Het | KO | genotype | | | pairwise comparisons | | |
|  |  |  |  |  |  | F | p-value | power | WT vs Het | WT vs KO | Het vs KO |  |  |  |  |  |  | F | p-value | power | WT vs Het | WT vs KO | Het vs KO |
| Distance border/total distance | ANOVA | normal | 75.49 ± 1.55 | 77.44 ± 2.37 | 77.43 ± 2.33 | 0.305 | 0.740 | 0.093 | - | - | - |  | ANOVA | normal | 78.77 ± 2.87 | 81.95 ± 2.27 | 81.72 ± 1 | 0.683 | 0.515 | 0.151 | - | - | - |
|  |  |  |  |  |  |  |  |  |  |  |  |  |  |  |  |  |  |  |  |  |  |  |  |
| Distance border/total distance, repeated measures | test | data structure |  | | | F | p-value | power | WT vs Het | WT vs KO | Het vs KO |  | test | data structure |  | | | F | p-value | power | WT vs Het | WT vs KO | Het vs KO |
| - time effect | repeated measures | sphericity assumed |  |  |  | 5.007 | **0.000** | 0.981 | - | - | - |  | repeated measures | sphericity assumed |  |  |  | 3.128 | **0.011** | 0.865 | - | - | - |
| - time x genotype effect | repeated measures | sphericity assumed |  |  |  | 0.910 | 0.526 | 0.463 | - | - | - |  | repeated measures | sphericity assumed |  |  |  | 0.960 | 0.481 | 0.485 | - | - | - |
| - genotype effect | repeated measures | sphericity assumed |  |  |  | 0.279 | 0.759 | 0.090 | - | - | - |  | repeated measures | sphericity assumed |  |  |  | 0.834 | 0.447 | 0.176 | - | - | - |
|  |  |  |  |  |  |  |  |  |  |  |  |  |  |  |  |  |  |  |  |  |  |  |  |
|  | test | data structure | WT | Het | KO | genotype | | | pairwise comparisons | | |  | test | data structure | WT | Het | KO | genotype | | | pairwise comparisons | | |
|  |  |  |  |  |  | F | p-value | power | WT vs Het | WT vs KO | Het vs KO |  |  |  |  |  |  | F | p-value | power | WT vs Het | WT vs KO | Het vs KO |
| Distance center/total distance | ANOVA | normal | 24.08 ± 1.59 | 22.06 ± 2.4 | 22.26 ± 2.31 | 0.298 | 0.745 | 0.092 | - | - | - |  | ANOVA | normal | 21.22 ± 2.87 | 18.04 ± 2.27 | 18.27 ± 1 | 0.683 | 0.515 | 0.151 | - | - | - |
|  |  |  |  |  |  |  |  |  |  |  |  |  |  |  |  |  |  |  |  |  |  |  |  |
| Distance center/total distance, repeated measures | test | data structure |  | | | F | p-value | power | WT vs Het | WT vs KO | Het vs KO |  | test | data structure |  | | | F | p-value | power | WT vs Het | WT vs KO | Het vs KO |
| - time effect | repeated measures | sphericity assumed |  |  |  | 5.222 | **0.000** | 0.985 | - | - | - |  | repeated measures | sphericity assumed |  |  |  | 3.128 | **0.011** | 0.865 | - | - | - |
| - time x genotype effect | repeated measures | sphericity assumed |  |  |  | 0.874 | 0.559 | 0.445 | - | - | - |  | repeated measures | sphericity assumed |  |  |  | 0.960 | 0.481 | 0.485 | - | - | - |
| - genotype effect | repeated measures | sphericity assumed |  |  |  | 0.264 | 0.770 | 0.087 | - | - | - |  | repeated measures | sphericity assumed |  |  |  | 0.834 | 0.447 | 0.176 | - | - | - |
|  |  |  |  |  |  |  |  |  |  |  |  |  |  |  |  |  |  |  |  |  |  |  |  |
|  | test | data structure | WT | Het | KO | genotype | | | pairwise comparisons | | |  | test | data structure | WT | Het | KO | genotype | | | pairwise comparisons | | |
|  |  |  |  |  |  | F | p-value | power | WT vs Het | WT vs KO | Het vs KO |  |  |  |  |  |  | F | p-value | power | WT vs Het | WT vs KO | Het vs KO |
| Distance border/center | Kruskal-Wallis | non normal | 3.3 ± 0.26 | 4.1 ± 0.64 | 4 ± 0.65 | 0.312 | 0.856 | NA | - | - | - |  | Kruskal-Wallis | non normal | 4.44 ± 0.81 | 5.72 ± 1.38 | 4.65 ± 0.36 | 0.787 | 0.675 | NA | - | - | - |
|  |  |  |  |  |  |  |  |  |  |  |  |  |  |  |  |  |  |  |  |  |  |  |  |
| Distance border/center, repeated measures | test | data structure |  | | | F | p-value | power | WT vs Het | WT vs KO | Het vs KO |  | test | data structure |  | | | F | p-value | power | WT vs Het | WT vs KO | Het vs KO |
| - time effect | repeated measures | sphericity violated |  |  |  | 1.615 | 0.208 | 0.329 | - | - | - |  | repeated measures | sphericity violated |  |  |  | 0.548 | 0.611 | 0.144 | - | - | - |
| - time x genotype effect | repeated measures | sphericity violated |  |  |  | 1.012 | 0.410 | 0.300 | - | - | - |  | repeated measures | sphericity violated |  |  |  | 0.800 | 0.549 | 0.260 | - | - | - |
| - genotype effect | repeated measures | sphericity violated |  |  |  | 0.767 | 0.474 | 0.167 | - | - | - |  | repeated measures | sphericity violated |  |  |  | 1.294 | 0.293 | 0.253 | - | - | - |
|  |  |  |  |  |  |  |  |  |  |  |  |  |  |  |  |  |  |  |  |  |  |  |  |
|  | test | data structure | WT | Het | KO | genotype | | | pairwise comparisons | | |  | test | data structure | WT | Het | KO | genotype | | | pairwise comparisons | | |
|  |  |  |  |  |  | F | p-value | power | WT vs Het | WT vs KO | Het vs KO |  |  |  |  |  |  | F | p-value | power | WT vs Het | WT vs KO | Het vs KO |
| Time in border (seconds) | ANOVA | normal | 2986.77 ± 73.6 | 2924.17 ± 115.44 | 2987.27 ± 100.75 | 0.141 | 0.869 | 0.069 | - | - | - |  | ANOVA | normal | 2946.65 ± 141.1 | 3069.69 ± 102.22 | 3139.51 ± 73.24 | 0.852 | 0.439 | 0.179 | - | - | - |
|  |  |  |  |  |  |  |  |  |  |  |  |  |  |  |  |  |  |  |  |  |  |  |  |
| Time in border, repeated measures | test | data structure |  | | | F | p-value | power | WT vs Het | WT vs KO | Het vs KO |  | test | data structure |  | | | F | p-value | power | WT vs Het | WT vs KO | Het vs KO |
| - time effect | repeated measures | sphericity violated |  |  |  | 2.546 | *0.056* | 0.638 | - | - | - |  | repeated measures | sphericity assumed |  |  |  | 1.962 | *0.089* | 0.644 | - | - | - |
| - time x genotype effect | repeated measures | sphericity violated |  |  |  | 0.829 | 0.560 | 0.327 | - | - | - |  | repeated measures | sphericity assumed |  |  |  | 0.770 | 0.657 | 0.388 | - | - | - |
| - genotype effect | repeated measures | sphericity violated |  |  |  | 0.141 | 0.869 | 0.069 | - | - | - |  | repeated measures | sphericity assumed |  |  |  | 0.852 | 0.439 | 0.179 | - | - | - |
|  |  |  |  |  |  |  |  |  |  |  |  |  |  |  |  |  |  |  |  |  |  |  |  |
|  | test | data structure | WT | Het | KO | genotype | | | pairwise comparisons | | |  | test | data structure | WT | Het | KO | genotype | | | pairwise comparisons | | |
|  |  |  |  |  |  | F | p-value | power | WT vs Het | WT vs KO | Het vs KO |  |  |  |  |  |  | F | p-value | power | WT vs Het | WT vs KO | Het vs KO |
| Time in center (seconds) | ANOVA | normal | 591.22 ± 75.09 | 649.04 ± 116.54 | 597.4 ± 100.64 | 0.107 | 0.899 | 0.065 | - | - | - |  | ANOVA | normal | 640.75 ± 140.19 | 519.8 ± 102.24 | 445.76 ± 73.41 | 0.872 | 0.431 | 0.182 | - | - | - |
|  |  |  |  |  |  |  |  |  |  |  |  |  |  |  |  |  |  |  |  |  |  |  |  |
| Time in center, repeated measures | test | data structure |  | | | F | p-value | power | WT vs Het | WT vs KO | Het vs KO |  | test | data structure |  | | | F | p-value | power | WT vs Het | WT vs KO | Het vs KO |
| - time effect | repeated measures | sphericity violated |  |  |  | 2.464 | *0.063* | 0.620 | - | - | - |  | repeated measures | sphericity assumed |  |  |  | 2.360 | **0.044** | 0.738 | - | - | - |
| - time x genotype effect | repeated measures | sphericity violated |  |  |  | 0.814 | 0.616 | 0.320 | - | - | - |  | repeated measures | sphericity assumed |  |  |  | 0.705 | 0.718 | 0.354 | - | - | - |
| - genotype effect | repeated measures | sphericity violated |  |  |  | 0.107 | 0.899 | 0.065 | - | - | - |  | repeated measures | sphericity assumed |  |  |  | 0.872 | 0.431 | 0.182 | - | - | - |
|  |  |  |  |  |  |  |  |  |  |  |  |  |  |  |  |  |  |  |  |  |  |  |  |
|  | test | data structure | WT | Het | KO | genotype | | | pairwise comparisons | | |  | test | data structure | WT | Het | KO | genotype | | | pairwise comparisons | | |
|  |  |  |  |  |  | F | p-value | power | WT vs Het | WT vs KO | Het vs KO |  |  |  |  |  |  | F | p-value | power | WT vs Het | WT vs KO | Het vs KO |
| Time border/center | Kruskal-Wallis | non normal | 6.18 ± 0.96 | 7 ± 1.79 | 9.61 ± 4.32 | 0.107 | 0.948 | NA | - | - | - |  | Kruskal-Wallis | non normal | 8.01 ± 2.94 | 9.75 ± 3.51 | 9.88 ± 2.15 | 0.867 | 0.648 | NA | - | - | - |
|  |  |  |  |  |  |  |  |  |  |  |  |  |  |  |  |  |  |  |  |  |  |  |  |
| Time border/center, repeated measures | test | data structure |  | | | F | p-value | power | WT vs Het | WT vs KO | Het vs KO |  | test | data structure |  | | | F | p-value | power | WT vs Het | WT vs KO | Het vs KO |
| - time effect | repeated measures | sphericity violated |  |  |  | 0.760 | 0.440 | 0.155 | - | - | - |  | repeated measures | sphericity violated |  |  |  | 0.460 | 0.639 | 0.122 | - | - | - |
| - time x genotype effect | repeated measures | sphericity violated |  |  |  | 1.641 | 0.194 | 0.400 | - | - | - |  | repeated measures | sphericity violated |  |  |  | 0.785 | 0.544 | 0.235 | - | - | - |
| - genotype effect | repeated measures | sphericity violated |  |  |  | 1.104 | 0.346 | 0.223 | - | - | - |  | repeated measures | sphericity violated |  |  |  | 0.866 | 0.434 | 0.180 | - | - | - |
|  |  |  |  |  |  |  |  |  |  |  |  |  |  |  |  |  |  |  |  |  |  |  |  |
| **Vertical activity in openfield** |  |  |  |  |  |  |  |  |  |  |  |  |  |  |  |  |  |  |  |  |  |  |  |
|  | Cohort 1 | | | | | | | | | | |  | Cohort 2 | | | | | | | | | | |
|  | test | data structure | WT | Het | KO | genotype | | | pairwise comparisons | | |  | test | data structure | WT | Het | KO | genotype | | | pairwise comparisons | | |
|  |  |  |  |  |  | F | p-value | power | WT vs Het | WT vs KO | Het vs KO |  |  |  |  |  |  | F | p-value | power | WT vs Het | WT vs KO | Het vs KO |
| Free rears, total duration (sec) | Kruskal-Wallis | non normal | 4.49 ± 1.76 | 9.48 ± 2.56 | 4.16 ± 1.02 | 5.773 | *0.056* | NA | - | - | - |  | Kruskal-Wallis | non normal | 48.88 ± 18.43 | 62.16 ± 18.79 | 80.09 ± 26.01 | 0.826 | 0.662 | NA | - | - | - |
| Free rears, number | Kruskal-Wallis | non normal | 9.9 ± 3.47 | 13.6 ± 2.45 | 6.77 ± 1.11 | 4.858 | *0.088* | NA | - | - | - |  | Kruskal-Wallis | non normal | 6.37 ± 1.74 | 7.33 ± 1.73 | 8.3 ± 2.12 | 0.380 | 0.827 | NA | - | - | - |
| Wall rears, total duration (sec) | Kruskal-Wallis | non normal | 16.16 ± 2.21 | 18.08 ± 3.36 | 9.16 ± 1.54 | 7.305 | **0.026** | NA | 0.817 | **0.023** | **0.014** |  | ANOVA | normal | 12.66 ± 29.26 | 13.97 ± 20.64 | 88.87 ± 8.74 | 1.868 | 0.176 | 0.350 | - | - | - |
| Wall rears, number | ANOVA | normal | 32.45 ± 2.88 | 33.8 ± 4.39 | 23.55 ± 3.36 | 2.264 | 0.123 | 0.420 | - | - | - |  | ANOVA | normal | 20.12 ± 3.86 | 20.55 ± 1.84 | 15.6 ± 1.05 | 1.455 | 0.253 | 0.280 | - | - | - |
| All rears, total duration (sec) | Kruskal-Wallis | non normal | 20.66 ± 2.9 | 27.57 ± 4.67 | 13.32 ± 1.88 | 5.850 | *0.054* | NA | - | - | - |  | Kruskal-Wallis | non normal | 17.55 ± 38.81 | 20.19 ± 35.24 | 16.89 ± 34.13 | 1.849 | 0.397 | NA | - | - | - |
| All rears, number | Kruskal-Wallis | non normal | 42.36 ± 4.83 | 47.4 ± 5.73 | 30.33 ± 3.58 | 6.463 | **0.039** | NA | 0.707 | **0.040** | **0.018** |  | ANOVA | normal | 26.5 ± 4.79 | 27.88 ± 2.79 | 23.9 ± 2.01 | 0.424 | 0.659 | 0.111 | - | - | - |
|  |  |  |  |  |  |  |  |  |  |  |  |  |  |  |  |  |  |  |  |  |  |  |  |
| **Zero-maze** |  |  |  |  |  |  |  |  |  |  |  |  |  |  |  |  |  |  |  |  |  |  |  |
|  | Cohort 1 | | | | | | | | | | |  | Cohort 2 | | | | | | | | | | |
|  | test | data structure | WT | Het | KO | genotype | | | pairwise comparisons | | |  | test | data structure | WT | Het | KO | genotype | | | pairwise comparisons | | |
|  |  |  |  |  |  | F | p-value | power | WT vs Het | WT vs KO | Het vs KO |  |  |  |  |  |  | F | p-value | power | WT vs Het | WT vs KO | Het vs KO |
| Time in closed arc, day 1 | Kruskal-Wallis | non normal | 391.14 ± 20.41 | 418.82 ± 13.71 | 425.98 ± 21.31 | 1.446 | 0.485 | NA | - | - | - |  | Kruskal-Wallis | non normal | 480.26 ± 25.29 | 467.09 ± 22.78 | 484.66 ± 27.01 | 0.943 | 0.624 | NA | - | - | - |
| Time in closed arc, day 2 | ANOVA | normal | 400.76 ± 23.56 | 444.65 ± 19.38 | 464.33 ± 23.28 | 2.173 | 0.133 | 0.405 | - | - | - |  | Kruskal-Wallis | non normal | 513.74 ± 20.1 | 545.23 ± 13.05 | 508.53 ± 52.91 | 3.092 | 0.213 | NA | - | - | - |
| Time in closed arc, mean | ANOVA | normal | 395.95 ± 20.9 | 431.73 ± 14.29 | 445.15 ± 19.66 | 1.893 | 0.170 | 0.358 | - | - | - |  | Kruskal-Wallis | non normal | 497 ± 18.32 | 506.16 ± 15.03 | 496.59 ± 33.09 | 0.863 | 0.649 | NA | - | - | - |
| Time in open arc, day 1 | Kruskal-Wallis | non normal | 201.39 ± 20.23 | 173.33 ± 13.1 | 157.78 ± 21.87 | 3.048 | 0.218 | NA | - | - | - |  | ANOVA | normal | 117.41 ± 25.44 | 130.6 ± 23.34 | 113.96 ± 27.32 | 0.120 | 0.888 | 0.054 | - | - | - |
| Time in open arc, day 2 | ANOVA | normal | 192.65 ± 24.72 | 151.43 ± 19.54 | 130.61 ± 23.41 | 1.936 | 0.164 | 0.366 | - | - | - |  | Kruskal-Wallis | non normal | 74.91 ± 20.52 | 47.37 ± 11.91 | 31.4 ± 15.87 | 5.516 | *0.063* | NA | - | - | - |
| Time in open arc, mean | ANOVA | normal | 197.02 ± 21.28 | 162.38 ± 14.16 | 144.2 ± 19.72 | 2.055 | 0.148 | 0.386 | - | - | - |  | Kruskal-Wallis | non normal | 96.16 ± 19.12 | 88.99 ± 15.14 | 72.68 ± 21.03 | 2.300 | 0.317 | NA | - | - | - |
| Ratio time close/open, day 1 | ANOVA | normal | 2.28 ± 0.35 | 2.61 ± 0.31 | 3.15 ± 0.43 | 1.419 | 0.259 | 0.277 | - | - | - |  | Kruskal-Wallis | non normal | 6.56 ± 1.65 | 5.14 ± 2.14 | 8.36 ± 3.01 | 1.504 | 0.410 | NA | - | - | - |
| Ratio time close/open, day 2 | Kruskal-Wallis | non normal | 2.84 ± 0.67 | 3.86 ± 0.84 | 6.52 ± 2.33 | 2.980 | 0.225 | NA | - | - | - |  | Kruskal-Wallis | non normal | 12.61 ± 3.82 | 52.23 ± 41.04 | 51.45 ± 19.6 | 3.414 | 0.181 | NA | - | - | - |
| Ratio time close/open, mean | ANOVA | normal | 2.43 ± 0.41 | 2.92 ± 0.34 | 3.73 ± 0.64 | 1.916 | 0.167 | 0.362 | - | - | - |  | Kruskal-Wallis | non normal | 7.61 ± 1.9 | 8.85 ± 4.23 | 13.42 ± 4.31 | 2.144 | 0.342 | NA | - | - | - |
| Number of open arc entries, day 1 | Kruskal-Wallis | non normal | 58.27 ± 4.5 | 57.3 ± 4.25 | 77.11 ± 12.17 | 0.870 | 0.647 | NA | - | - | - |  | ANOVA | normal | 35 ± 7.82 | 43 ± 7.64 | 43.4 ± 6.98 | 0.379 | 0.688 | 0.099 | - | - | - |
| Number of open arc entries, day 2 | ANOVA | normal | 50.63 ± 7.28 | 47.2 ± 5.42 | 44 ± 7.32 | 0.240 | 0.789 | 0.084 | - | - | - |  | ANOVA | normal | 31.12 ± 5.31 | 24.66 ± 6.39 | 16.1 ± 3.71 | 2.131 | 0.141 | 0.284 | - | - | - |
| Number of open arc entries, mean | ANOVA | normal | 54.45 ± 4.6 | 52.25 ± 3.65 | 60.55 ± 8.46 | 0.540 | 0.589 | 0.130 | - | - | - |  | ANOVA | normal | 33.06 ± 5.13 | 33.83 ± 6.2 | 29.75 ± 4.59 | 0.175 | 0.841 | 0.056 | - | - | - |
| Latency to enter in an open arc for the first time | Kruskal-Wallis | non normal | 27.83 ± 6.75 | 14.17 ± 3.86 | 39.5 ± 20.68 | 2.970 | 0.227 | NA | - | - | - |  | Kruskal-Wallis | non normal | 55.05 ± 30.13 | 83.65 ± 65.82 | 8.13 ± 4 | 0.483 | *0.083* | NA | - | - | - |
| Latency to fully cross an open arc for the first time | Kruskal-Wallis | non normal | 125.88 ± 30.26 | 90.06 ± 26.62 | 90.43 ± 30.27 | 1.657 | 0.437 | NA | - | - | - |  | Kruskal-Wallis | non normal | 181.59 ± 73.28 | 194.85 ± 65.42 | 99.5 ± 58.3 | 3.618 | 0.202 | NA | - | - | - |
| Dipping from close arc, frequency, day 1 | ANOVA | normal | 69.45 ± 7.33 | 76.4 ± 6.98 | 73.55 ± 11.12 | 0.180 | 0.836 | 0.075 | - | - | - |  | ANOVA | normal | 27.37 ± 5.57 | 32.11 ± 3.13 | 31.6 ± 3.42 | 0.389 | 0.682 | 0.086 | - | - | - |
| Dipping from close arc, frequency, day 2 | ANOVA | normal | 39.18 ± 2.99 | 39.2 ± 3.25 | 35.44 ± 4.95 | 0.322 | 0.727 | 0.096 | - | - | - |  | Kruskal-Wallis | non normal | 22 ± 3.64 | 16.12 ± 3.9 | 10.3 ± 2.02 | 6.412 | *0.041* | NA | 0.096 | **0.012** | 0.407 |
| Dipping from close arc, frequency, mean | ANOVA | normal | 54.31 ± 3.69 | 57.8 ± 3.49 | 54.5 ± 6.48 | 0.185 | 0.832 | 0.076 | - | - | - |  | ANOVA | normal | 24.68 ± 3.79 | 25.5 ± 3.41 | 20.95 ± 2.01 | 0.671 | 0.521 | 0.124 | - | - | - |
| Dipping from close arc, duration, day 1 | ANOVA | normal | 172.14 ± 9.16 | 189.92 ± 10.4 | 156.02 ± 13.5 | 2.297 | 0.120 | 0.425 | - | - | - |  | ANOVA | normal | 74.8 ± 18.66 | 96.82 ± 16.08 | 83.55 ± 17.17 | 0.391 | 0.681 | 0.166 | - | - | - |
| Dipping from close arc, duration, day 2 | ANOVA | normal | 119.23 ± 10.28 | 132.55 ± 13.54 | 92.2 ± 15.35 | 2.377 | 0.112 | 0.438 | - | - | - |  | Kruskal-Wallis | non normal | 60.53 ± 10.9 | 59.49 ± 18.71 | 39.24 ± 15.95 | 3.265 | 0.195 | NA | - | - | - |
| Dipping from close arc, duration, mean | ANOVA | normal | 145.68 ± 7.89 | 161.24 ± 9.41 | 124.11 ± 11.28 | 3.689 | **0.038** | 0.628 | 0.467 | 0.259 | **0.030** |  | ANOVA | normal | 67.66 ± 13.85 | 83.65 ± 14.85 | 61.4 ± 9.22 | 0.857 | 0.437 | 0.345 | - | - | - |
| Dipping from open arc, frequency, day 1 | Kruskal-Wallis | non normal | 34.09 ± 4.18 | 22.4 ± 2.66 | 23.77 ± 5.73 | 3.844 | 0.146 | NA | - | - | - |  | Kruskal-Wallis | non normal | 17.62 ± 4.88 | 17.88 ± 4.32 | 15 ± 5.3 | 0.883 | 0.543 | NA | - | - | - |
| Dipping from open arc, frequency, day 2 | Kruskal-Wallis | non normal | 22.45 ± 4.45 | 15.9 ± 3.1 | 15.88 ± 4 | 1.018 | 0.601 | NA | - | - | - |  | Kruskal-Wallis | non normal | 9.37 ± 1.84 | 7.62 ± 3.04 | 2.5 ± 1.56 | 7.090 | **0.029** | NA | 0.281 | **0.008** | 0.133 |
| Dipping from open arc, frequency, mean | Kruskal-Wallis | non normal | 28.27 ± 4.16 | 19.15 ± 1.9 | 19.83 ± 4.22 | 3.146 | 0.207 | NA | - | - | - |  | Kruskal-Wallis | non normal | 13.5 ± 2.95 | 14.55 ± 4.02 | 8.75 ± 3.34 | 2.847 | 0.241 | NA | - | - | - |
| Dipping from open arc, duration, day 1 | Kruskal-Wallis | non normal | 83.93 ± 14.51 | 53.37 ± 6.12 | 32.96 ± 6.02 | 8.463 | **0.016** | NA | 0.983 | **0.004** | 0.060 |  | Kruskal-Wallis | non normal | 33.86 ± 9.8 | 29.96 ± 6.59 | 18.93 ± 8.13 | 2.585 | 0.275 | NA | - | - | - |
| Dipping from open arc, duration, day 2 | Kruskal-Wallis | non normal | 71.41 ± 14.33 | 52.36 ± 12.31 | 33.14 ± 8.24 | 4.140 | 0.126 | NA | - | - | - |  | Kruskal-Wallis | non normal | 23.64 ± 9.16 | 13.98 ± 5.37 | 4.13 ± 3.17 | 7.036 | **0.030** | NA | 0.138 | **0.008** | 0.242 |
| Dipping from open arc, duration, mean | Kruskal-Wallis | non normal | 77.67 ± 12.62 | 52.87 ± 7.88 | 33.05 ± 6.03 | 8.411 | **0.015** | NA | 0.236 | **0.004** | 0.088 |  | Kruskal-Wallis | non normal | 28.75 ± 7.49 | 23.94 ± 5.51 | 11.53 ± 5.56 | 26.000 | *0.077* | NA | - | - | - |
|  |  |  |  |  |  |  |  |  |  |  |  |  |  |  |  |  |  |  |  |  |  |  |  |
| Open vs close arc time, day 1 | test | data structure |  | | | All t | All p-value | power |  |  |  |  | test | data structure |  | | | All t | All p-value | power |  |  |  |
| - All mice | repeated measures | sphericity assumed |  | | | 114.550 | **0.000** | 1.000 |  |  |  |  | repeated measures | sphericity assumed |  | | | 157.246 | **0.000** | 1.000 |  |  |  |
| - WT | repeated measures | sphericity assumed |  |  |  | 21.826 | **0.001** | 0.987 |  |  |  |  | repeated measures | sphericity assumed |  |  |  | 51.177 | **0.000** | 1.000 |  |  |  |
| - Het | repeated measures | sphericity assumed |  |  |  | 83.979 | **0.000** | 1.000 |  |  |  |  | repeated measures | sphericity assumed |  |  |  | 53.253 | **0.000** | 1.000 |  |  |  |
| - KO | repeated measures | sphericity assumed |  |  |  | 40.141 | **0.000** | 1.000 |  |  |  |  | repeated measures | sphericity assumed |  |  |  | 46.532 | **0.000** | 1.000 |  |  |  |
|  |  |  |  |  |  |  |  |  |  |  |  |  |  |  |  |  |  |  |  |  |  |  |  |
| Open vs close arc time, day 2 | test | data structure |  | | | All t | All p-value | power |  |  |  |  | test | data structure |  | | | All t | All p-value | power |  |  |  |
| - All mice | repeated measures | sphericity assumed |  | | | 103.335 | **0.000** | 1.000 |  |  |  |  | repeated measures | sphericity assumed |  | | | 359.443 | **0.000** | 1.000 |  |  |  |
| - WT | repeated measures | sphericity assumed |  |  |  | 18.608 | **0.002** | 0.972 |  |  |  |  | repeated measures | sphericity assumed |  |  |  | 117.574 | **0.000** | 1.000 |  |  |  |
| - Het | repeated measures | sphericity assumed |  |  |  | 56.747 | **0.000** | 1.000 |  |  |  |  | repeated measures | sphericity assumed |  |  |  | 399.191 | **0.000** | 1.000 |  |  |  |
| - KO | repeated measures | sphericity assumed |  |  |  | 51.107 | **0.000** | 1.000 |  |  |  |  | repeated measures | sphericity assumed |  |  |  | 71.920 | **0.000** | 1.000 |  |  |  |
|  |  |  |  |  |  |  |  |  |  |  |  |  |  |  |  |  |  |  |  |  |  |  |  |
| Open vs close arc time, mean | test | data structure |  | | | All t | All p-value | power |  |  |  |  | test | data structure |  | | | All t | All p-value | power |  |  |  |
| - All mice | repeated measures | sphericity assumed |  | | | 128.205 | **0.000** | 1.000 |  |  |  |  | repeated measures | sphericity assumed |  | | | 338.054 | **0.000** | 1.000 |  |  |  |
| - WT | repeated measures | sphericity assumed |  |  |  | 22.258 | **0.001** | 0.988 |  |  |  |  | repeated measures | sphericity assumed |  |  |  | 114.874 | **0.000** | 1.000 |  |  |  |
| - Het | repeated measures | sphericity assumed |  |  |  | 89.624 | 0.000 | 1.000 |  |  |  |  | repeated measures | sphericity assumed |  |  |  | 191.414 | **0.000** | 1.000 |  |  |  |
| - KO | repeated measures | sphericity assumed |  |  |  | 59.048 | **0.000** | 1.000 |  |  |  |  | repeated measures | sphericity assumed |  |  |  | 76.608 | **0.000** | 1.000 |  |  |  |
